# Evaluation of Antibacterial Functionalized Dihydropyrimidine Photoaffinity Probes Toward Mechanism of Action Studies

**DOI:** 10.1021/acsmedchemlett.4c00173

**Published:** 2024-06-20

**Authors:** Christopher M. Russo, Zachary W. Boyer, Kaitlyn Scheunemann, Jonathan Farren, Alexandra Minich, Cody J. Wenthur, Matthew C. O’Reilly

**Affiliations:** † Department of Chemistry, Villanova University, Villanova, Pennsylvania 19085, United States; ‡ Department of Chemistry and Biotechnology, University of Wisconsin−River Falls, River Falls, Wisconsin 54022, United States; § School of Pharmacy, University of Wisconsin−Madison, Madison, Wisconsin 53705, United States

**Keywords:** antibiotics, structure−activity relationship, MRSA, drug discovery, dihydropyrimidine, NMR, photoaffinity probes, diazirine

## Abstract

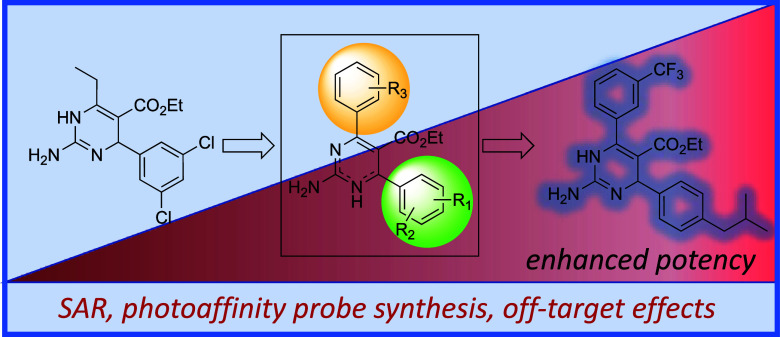

Antibiotic-resistant bacteria are a global health concern, necessitating the development of antibiotics working through new or underutilized mechanisms. Functionalized amino dihydropyrimidines have previously demonstrated potential as antibacterial agents, but they had limited potency, and their biological mechanism was not understood. To further evaluate their potential, focused libraries were prepared and screened for bacterial growth inhibition, and these compounds provided additional insights into the structure–activity relationships, allowing for the preparation of compounds that inhibited all strains of *Staphylococcus aureus* with an MIC of 2 μg/mL. After eliminating the proposed mechanism of dihydrofolate reductase inhibition, trifluoromethyl diazirine photoaffinity probes were synthesized to investigate their mechanism, and these were tested to ensure the photolabile group did not impact the antibacterial activity. Finally, the compounds were screened for hemolysis and mammalian cytotoxicity. While they lacked nonspecific membrane rupturing activity, many of the compounds showed significant mammalian cytotoxicity, indicating further development will be required to render them selective for bacteria.

Antibiotic-resistant (AR) bacteria pose a significant infection threat to the world community.^1,2^ A systematic analysis indicated that AR bacteria were directly responsible for the deaths of 1.27 million people worldwide in 2019 alone, and these infections contributed to the deaths of an additional 4.95 million people.^1,2^ Methicillin-resistant *Staphylococcus aureus* (MRSA) caused more than 100000 of these fatalities, making it the leading cause of death by AR bacteria.^1,2^ The World Bank estimates that antimicrobial resistance is likely to decrease global gross domestic product by $1–3.4 trillion within the decade and is expected to increase healthcare costs by $1 trillion by 2050.^3^ Recommendations to mitigate these threats include the development of novel antibiotics that work through new or underexploited mechanisms.

Trimethoprim **1** is the only FDA approved antibacterial working via inhibition of dihydrofolate reductase (DHFR), and its pyrimidine ring resembles the substrate dihydrofolate. Emmacin **2**, discovered in 2008, was found to inhibit MRSA growth, and its dihydropyrimidine (DHP) structure along with enzyme kinetics assays suggested it may work via DHFR inhibition (Figure 1a).^4,5^ Recent work synthesized focused libraries of DHPs that systematically (1) omitted the chloro- and hydroxy-substituents from the benzene ring, (2) substituted larger and smaller groups in place of the core ethyl substituent, and (3) replaced the guanidine with a urea or thiourea; these modifications provided structure activity relationship (SAR) insights (Figure 1b).^6^ Importantly, the most significant potency increases occurred by way of modifying the arene substituents, and the core ethyl group could be larger (phenyl) or smaller (methyl) with minimal impacts to bioactivity. Herein, we further evaluate these trends, demonstrating that alkyl substitution can significantly improve antibacterial potency, and compounds were discovered that inhibit MRSA growth at concentrations as low as 2 μg/mL. Additionally, the question of whether DHPs are active DHFR inhibitors was investigated. On the hypothesis that this inconsistency could be due to the errant presence of pyrimidines, a selection of DHPs were intentionally oxidized to their corresponding pyrimidines, which were found to lack both DHFR inhibition and antibacterial activity. Toward fully evaluating the mechanism of action, DHPs were synthesized with photoaffinity labels; they were validated to maintain potent antibacterial activity, and they will be used in future studies to explore the biological target of these compounds. Finally, compounds were screened for hemolytic activity and cytotoxicity toward a kidney cell line (HEK 293). While the compounds lacked nonspecific hemolysis, mammalian cytotoxicity was observed for many compounds.

**Figure 1:**
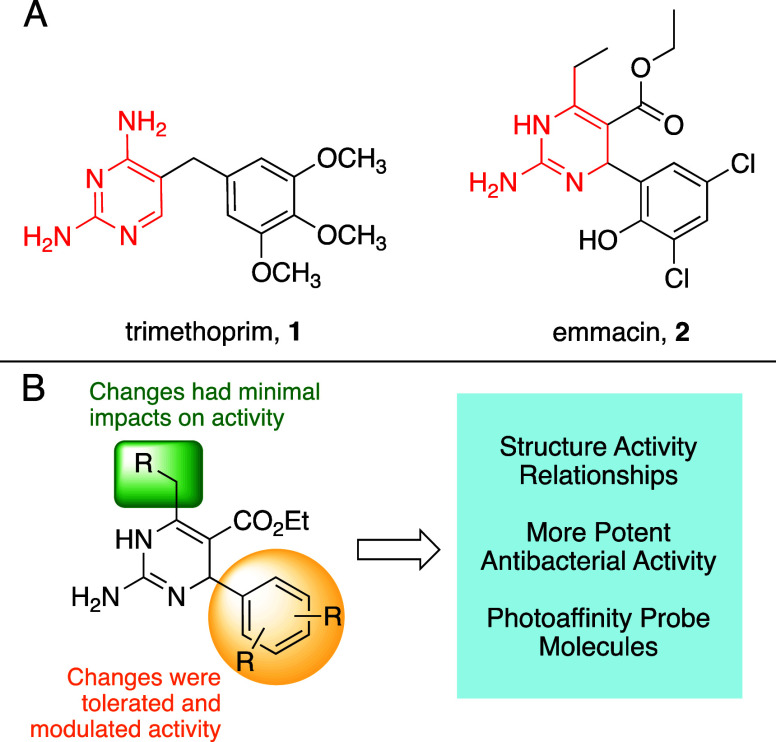
(A) Structural similarities between FDA-approved trimethoprim 1 and DHP emmacin 2. (B) SAR trend providing a basis for this study.

The three-component modified aza-Biginelli reaction was utilized to expeditiously synthesize DHPs to evaluate their SAR, which involves heating an aryl aldehyde (**3**), β-keto ester (**4**), and guanidine (**5**) in DMF under mildly basic conditions (Figure 2).^7,8^ Former work from our group and others demonstrated that the aza-Biginelli chemistry was significantly more successful and operationally facile when the β-keto ester **4**’s R-group was a phenyl ring.^6,9,10^ Further, a phenyl ring, compared to smaller alkyl groups, had limited impacts on the antibacterial activity of the DHP products, indicating that keeping the β-keto ester standard as ethyl benzoylacetate would facilitate rapid synthesis while allowing variations of the southeast arene’s substitution using a variety of substituted benzaldehydes **3**.^6^ Previous analogues included chlorination of the 3- and 5-positions and oxygenation of the 2-position, but no systematic study examining diverse substituents in other positions has occurred. Therefore, mono- and disubstituted benzaldehydes were used in the aza-Biginelli reaction to provide DHP products containing strong electron-withdrawing groups (−NO_2_, **6a**–**c**; −CF_3_, **6d**,**e**), modestly electron-withdrawing halogens (−F/Cl, **6f**–**j**), modestly electron-donating groups (alkyl, **6k**–**r**), and strongly electron-donating groups (−OCH_3_/N(CH_3_)_2_, **6s**–**w**), which could then be compared to previously synthesized nonsubstituted benzaldehyde product **6** and dichlorobenzaldehyde product **6j** (Table 1). All compounds were produced using the aza-Biginelli reaction, as our former study described,^6^ and synthesis and characterization details are in the Supporting Information.

**Figure 2:**
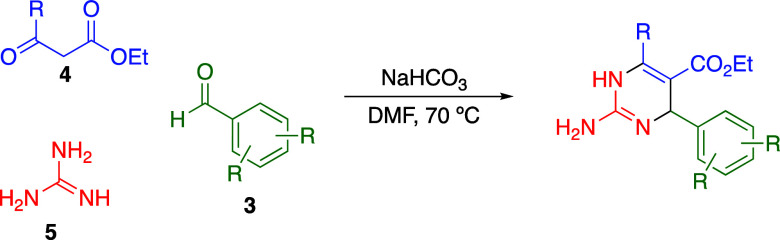
Aza-Biginelli three-component reaction used to synthesize focused libraries of dihydropyrimidines.

**Table 1:**
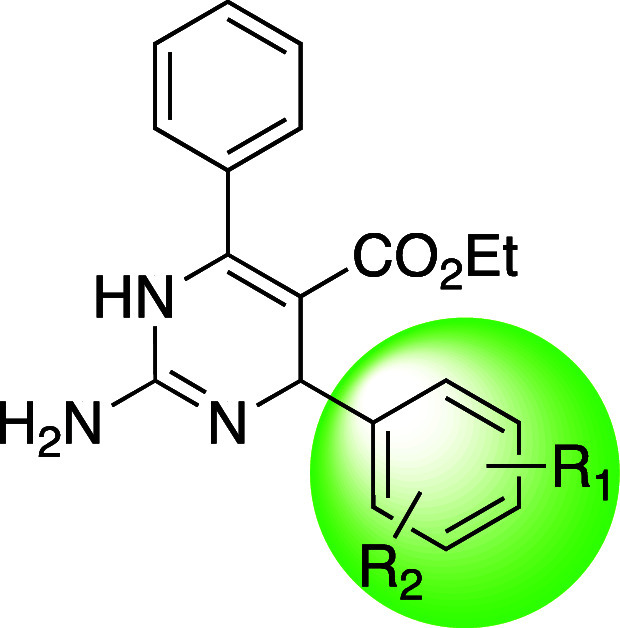
Structures and Antibacterial Activity of Focused Library **1** Measured As Minimum Inhibitory Concentrations (MIC) in *Staphylococcus aureus* Strains^a^

	R_1_	R_2_	ATCC 12600	ATCC 33591	ATCC 43300
**6**	H	H	>64^b^	>64^b^	>64^b^
**6a**	2-NO_2_	H	>64^b^	>64^b^	>64^b^
**6b**	3-NO_2_	H	64	64-I^c^	64-I^c^
**6c**	4-NO_2_	H	64	64	64
**6d**	3-CF_3_	H	32	32	32
**6e**	4-CF_3_	H	16	16	16
**6f**	2-F	H	>64^b^	>64^b^	>64^b^
**6g**	3-F	H	64-I^c^	64-I^c^	64-I^c^
**6h**	4-F	H	64-I^c^	64-I^c^	64-I^c^
**6i**	3-Cl	H	32	32	32
**6j**	3-Cl	5-Cl	8-I^c^	8-I^c^	8-I^c^
**6k**	2-CH_3_	H	64-I^c^	64-I^c^	64-I^c^
**6l**	3-CH_3_	H	64-I^c^	64-I^c^	64-I^c^
**6m**	4-CH_3_	H	>64^b^	>64^b^	>64^b^
**6n**	2-CH_3_	6-CH_3_	32-I^c^	64	32-I^c^
**6o**	2-CH_3_	4-CH_3_	32-I^c^	32-I^c^	32-I^c^
**6p**	3-CH_3_	5-CH_3_	32-I^c^	32-I^c^	32
**6q**	3-CH_3_	5-CH_3_	>64^b^	>64^b^	>64^b^
**6r**	4-CH(CH_3_)_2_	H	8-I^c^	8-I^c^	8-I^c^
**6s**	2-OCH_3_	H	>64^b^	>64^b^	>64^b^
**6t**	3-OCH_3_	H	>64^b^	>64^b^	>64^b^
**6u**	4-OCH_3_	H	64-I^c^	>64	>64
**6v**	3-OCH_3_	5-OCH_3_	>64^b^	>64^b^	>64^b^
**6w**	4-N(CH_3_)_2_	H	64-I^c^	64-I^c^	64-I^c^
carbenicillin			2	128	32
erythromycin			0.5	>512	>512
gentamycin			4	4	128
trimethoprim			32-I^c^	8-I^c^	8-I^c^
vancomycin			2	2	2

a Minimum inhibitory concentrations are defined as μg/mL amounts inhibiting greater than 95% of bacterial growth based on absorbance at 600 nm.

b Testing concentrations higher than the listed value was not performed due to compound solubility limitations.

c The “I” designation indicates that partial growth inhibition occurred at the value indicated, and >95% inhibition occurred at higher concentrations.

These 24 DHP analogues were then screened for inhibition of *Staphylococcus aureus* growth using microdilution assays. Growth inhibition was examined in methicillin-sensitive *S. aureus* (ATCC 12600) and two strains of MRSA (ATCC 33591 and ATCC 43300). Antibiotic controls were included in the screen, including carbenicillin, erythromycin, gentamycin, trimethoprim, and vancomycin, and minimum inhibitory concentrations (MICs) were determined for each compound, which were defined as the concentration required to inhibit >95% of bacterial growth (Table 1). The differences in antibiotic resistance properties of the three *S. aureus* strains were remarkable, and only vancomycin could inhibit the growth of all three strains at a low MIC (2 μg/mL). Our initial focused library had limited potency for growth inhibition, but trends did emerge from the data allowing for rational design of a second library. Specifically, analogues with strongly electron donating substituents were typically fully inactive (**6s**–**w**). Strongly electron withdrawing nitro groups also lacked potency, but a 4-NO_2_ substituent (**6c**) showed more growth inhibition than either of the other regioisomers (**6a**,**b**). That trend continued with trifluoromethyl substituents, where a 4-CF_3_ substituted compound provided enhanced potency (**6e**) over the 3-substituted analogue (**6d**). Fluorinated analogues (**6f**–**h**) lacked strong growth inhibition, while 3-chloro (**6i**) and 3,5-dichloro (**6j**) analogues showed stronger growth inhibition. Alkyl substituted compounds (**6k**–**r**) generally displayed modest growth inhibition, but a 4-isopropyl analogue (**6r**) was among the most potent in this focused library, providing inhibition at a concentration of 8 μg/mL. In all, the trends showed that weakly electron donating or withdrawing substituents were best for bacterial growth inhibition, with 3,5-dichloro (**6j**) and 4-isopropyl (**6r**) substitutions being the most promising options. Further, as both the 4-isopropyl and 4-CF_3_ analogues were rather bulky at the 4-position, there seemed to be a slight trend where activity levels may be enhanced if bulky groups were placed in that position.

With these data in mind, a second-generation library was designed to evaluate more steric bulk in the 4-position of the aldehyde-sourced arene. Therefore, 4-isobutylbenzaldehyde (**7a**) and 4-biphenylcarboxaldehyde (**7b**) adducts were produced (Table 2), and they were found to have enhanced potency compared to all compounds formerly described, inhibiting *S. aureus* growth at 4 μg/mL (Table 2). Those two benzaldehydes and 3,5-dichlorobenzaldehyde were then combined with various other β-keto esters that decreased the steric size of the R_1_ position (**7c**,**d**) or substituted the R_1_ naked phenyl ring with a trifluoromethyl-, fluoro-, or methyl-substituted arene (**7e**–**j**). In concert, these changes produced our most potent analogue yet, which combined a 3-trifluoromethylphenyl group in the R_1_ position with a 4-isobutylphenyl group in the R_2_ position (**7f**), and this compound inhibited the growth of all three *S. aureus* strains at 2 μg/mL, the same concentration required of vancomycin to elicit this effect. All compounds in this focused library inhibited the growth of *S. aureus* at concentrations less than or equal to 8 μg/mL (Table 2).

**Table 2:**
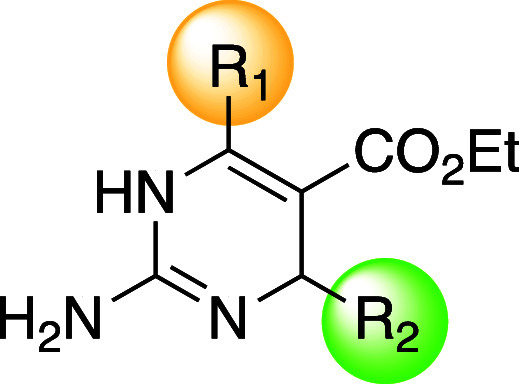
Structures and Antibacterial Activity of Focused Library 2 Measured as Minimum Inhibitory Concentrations (MIC) in *Staphylococcus aureus* Strains^a^

	R_1_	R_2_	ATCC 12600	ATCC 33591	ATCC 43300
**7a**	Ph	4-isobutyl-Ph	4	4-I^b^	4
**7b**	Ph	4-Ph-Ph	4	4	4
**7c**	Et	4-isobutyl-Ph	4-I^b^	8	4-I^b^
**7d**	Et	4-Ph-Ph	4-I^b^	8	4-I^b^
**7e**	3-CF_3_-Ph	3,5-Cl_2_-Ph	4	4	4
**7f**	3-CF_3_-Ph	4-isobutyl-Ph	2	2	2
**7g**	3-F-Ph	3,5-Cl_2_-Ph	8	8	8
**7h**	3-F-Ph	4-isobutyl-Ph	4	4	4
**7i**	3-CH_3_-Ph	3,5-Cl_2_-Ph	8	8	8
**7j**	3-CH_3_-Ph	4-isobutyl-Ph	4	4	4

a Minimum inhibitory concentrations are defined as μg/mL amounts inhibiting greater than 95% of bacterial growth based on absorbance at 600 nm.

b The “I” designation indicates that partial growth inhibition occurred at the value indicated, and >95% inhibition occurred at higher concentrations.

Former DHPs have been reported to inhibit dihydrofolate reductase (DHFR),^4^ but a secondary study was unable to link the antibacterial activity to DHFR inhibition.^6^ As our new DHPs were significantly more potent for antibacterial growth inhibition than those disclosed in the prior study (>4-fold more potent), we felt called to investigate whether the increased potency could be associated with DHFR inhibition. Toward this end, recombinant *Staphylococcus aureus* DHFR (*Sa*DHFR) was expressed, purified, and validated,^6,11^ and our new compounds had no discernible DHFR inhibition. As pyrimidine rings are the chief chemical motif known to produce DHFR inhibition, and literature accounts of dihydropyrimidine inhibition of DHFR exist, we considered that a DHP could be oxidized by air or under assay conditions to a pyrimidine, which may have caused the previously reported DHFR inhibition. To explore this, oxidization of DHP **7a** to the pyrimidine **8a** was performed using phenyliodine diacetate (PIDA) (Figure 3a),^12−14^ as this oxidation state mimics FDA approved antimicrobial DHFR inhibitors trimethoprim **1** (Figure 1a) and pyrimethamine **9** (Figure 3b). When **8a** was screened for antibacterial activity, however, it was found to have no impact on the growth of *S. aureus* at concentrations up to 64 μg/mL. While this demonstrates that pyrimidine **8a** lacks antibacterial activity, it could still inhibit DHFR *in vitro*. This hypothesis was subsequently explored, but no inhibition was observed. As the initial report of DHFR inhibition by a DHP concerned a compound that lacked the phenyl ring derived from the β-keto ester (Figure 1a, compound **2**), we next considered that the phenyl ring of **8a** may be too large for the DHFR pocket, where inhibitors bind. Therefore, we oxidized a previously reported antibacterial DHP that replaced that phenyl ring with a methyl substituent to pyrimidine **10** to explore the sterics in question. Compound **10** also lost all antibacterial activity and lacked DHFR inhibition, further suggesting that our DHPs likely generate their antibacterial effects through a separate mechanism. These compounds provided deeper insights into the antibacterial SAR, demonstrating that unsaturation in the dihydropyrimidine is essential for antibacterial activity.

**Figure 3:**
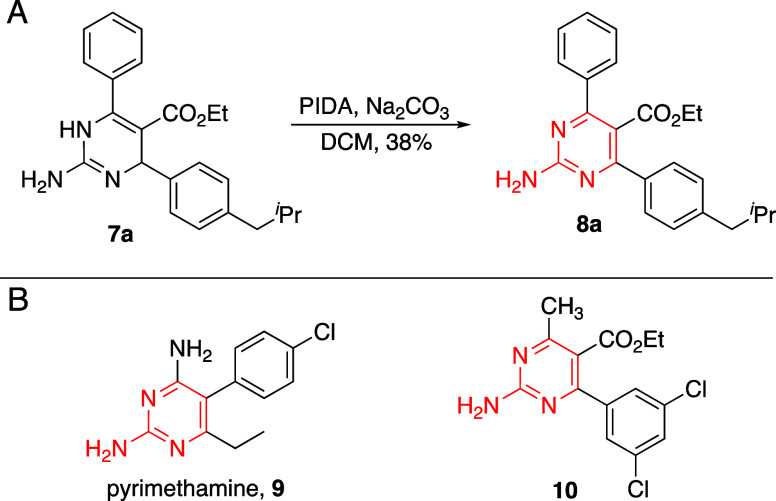
(A) Oxidation of antibacterial DHPs to pyrimidine analogues. (B) FDA-approved trimethoprim **1** and pyrimethamine **9** contain a pyrimidine motif important for DHFR inhibition, and DHP **2** was previously reported to inhibit DHFR. Oxidized pyrimidine products **8a** and **10** lacked antibacterial activity and DHFR inhibition.

To explore the antibacterial mechanism, we considered that photoaffinity labeling (PAL) may allow us to identify specific biological targets.^15−17^ Trifluoromethyl aryl diazirines can be ideal photolabile functional groups,^18,19^ which could be used with the cellular lysate, or in pulse-chase experiments with live cells to label targets in their appropriate biological context.^19,20^ As many of our most successful antibacterial compounds had multiple arenes as components (**7a**–**j**), we considered that a trifluoromethyl diazirine could be placed on either aryl position, with our initial emphasis on the β-keto ester, as this arene appeared to have less impact on the antibacterial activity. We envisioned accessing 4-trifluoromethyldiaziryl substituted β-keto ester **11** from precursor acetophenone **12** via base-promoted Claisen-type condensation with diethyl carbonate (Figure 4). Compound **12**’s diazirine would be constructed from the trifluoromethyl ketal **13**, which is available in two steps from commercially available and inexpensive 4-bromoacetophenone **14**.

**Figure 4:**
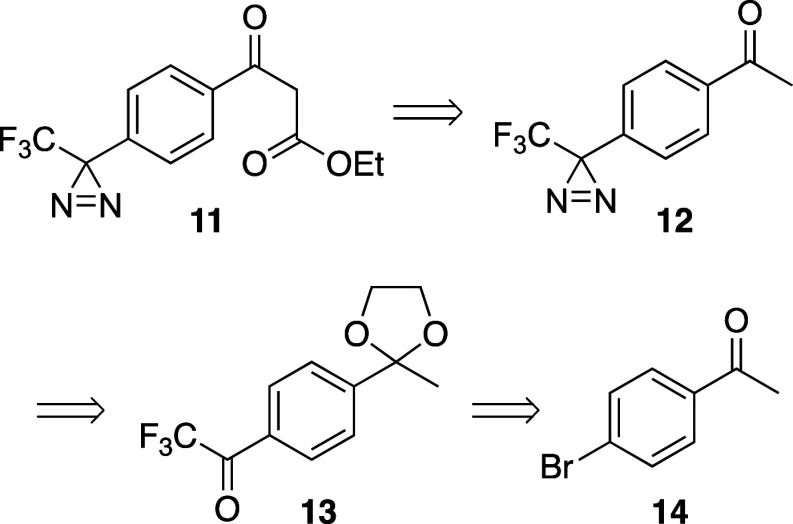
Retrosynthesis of trifluoromethyl diazirine functionalized β-keto ester.

Trifluoromethyl diazirines are known to be stable to acidic and basic conditions,^18,19^ and aryl ketone **14** was first protected as the acid labile ketal **15** to provide a stable functional group for installation of the trifluoromethyl ketone (Scheme 1). Toward this end, *n*-butyllithium promoted lithium-halogen exchange on bromo ketal **15**, and ethyl trifluoroacetate was added to the reaction to promote acyl transfer to the arene, forming **13**.^21,22^ Initial efforts used 2 equiv of *n*-butyllithium and produced the product in low yield (<20%). Instead, the reduced product alcohol was isolated as the major product (50–60%). Literature examples did not mention this secondary product, but they described variable yields of the trifluoromethyl ketone (∼80–30%).^21,23^ Yield of the trifluoromethyl ketone **13** improved to 75% by decreasing *n*-butyllithium equivalents to 1.5 (from 2.0 initially) and quenching the reaction at 0 °C. Oxime formation occurred smoothly to produce **16** in a 76% isolated yield, and tosylation, diaziridine formation, oxidation to diazirine, and ketal deprotection were performed without intermediate purification in 55% over four steps, providing this penultimate intermediate in 31% overall yield from 4-bromoacetophenone. Treatment of **12** with sodium hydride was expected to form the enolate, which we anticipated would promote acyl transfer from diethyl carbonate to provide the desired β-keto ester **11**. Unfortunately, this instead led to the complete decomposition of our starting material. This surprised us, as trifluoromethyldiaziryl acetophenones have precedented stability under strongly basic conditions (NaNH_2_ and NaH),^24,25^ but no alternative conditions promoted this reaction in our laboratory. Further, the reaction with diethyl carbonate was reproduced with a diazirine-lacking model substrate, and the reaction progressed as expected, indicating that the diazirine functional group could not tolerate those basic conditions.

**Scheme 1:**
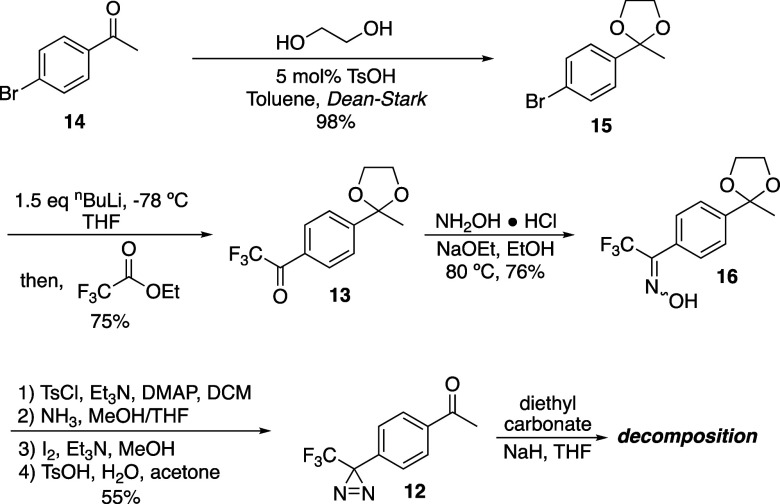
Synthesis of Diazirine Aza-Biginelli Precursors

An alternate route beginning with a halogenated benzyl alcohol was pursued, with the purpose of forming both the diazirine-containing benzaldehyde **25** and β-keto ester **11** products from a common intermediate (Scheme 2).^22^ Toward this end, 4-bromobenzyl alcohol **17** was silyl protected in high yield to produce **18**, and diazirine synthesis occurred analogously to Scheme 1 (**18** → **23**). The only significant change occurred during oxime formation, where a mildly acidic procedure, not tolerated by ketal **13**, provided oxime **21** in good yield with a significant reduction in reaction time. Overall, all reactions were more efficient when the silyl protecting group was employed, providing the functionalized product **23** in 77% overall yield through five steps (∼94% per step). Silyl ether cleavage followed by a Dess–Martin Periodinane (DMP) oxidation provided aldehyde **25**. While this aldehyde could already be used for aza-Biginelli dihydropyrimidine synthesis, further functionalization to the β-keto ester **11** was pursued, as that would enable addition of the diazirine to either arene of the dihydropyrimidine products. Toward this goal, chemistry from Holmquist and Roskamp produced β-keto esters directly via addition of diazoesters to aldehydes.^26−28^ While this reaction had a moderate scope,^27^ we were concerned that the Lewis acidic conditions could promote ring opening of the diazirine to a diazoalkane, potentially leading to decomposition.^29^ To optimize the reaction prior to involving the trifluoromethyl diazirine, we used 4-bromobenzaldehyde as a model substrate (Table S1, compound S1). Consistent with previously reported behavior for aromatic aldehydes,^27^ initial attempts to convert 4-bromobenzaldehyde to a β-keto ester **S2** using 5 mol % tin(II) chloride were mostly unsuccessful. Increasing catalyst loading to 50 mol % improved the reaction and gave **S2** with a typical yield of 53%. The use of other reportedly successful catalysts niobium(V) chloride or molybdenum(VI) dichloride dioxide were unsuccessful in our hands,^30,31^ while the use of more reactive catalysts such as tin(IV) chloride or boron trifluoride resulted in complicated mixtures.^32,33^ Tin(II) chloride was the most successful, and it was applied to diazirine benzaldehyde **25**. Gratifyingly, this converted aldehyde **25** to β-keto ester **11** in 59% average yield, demonstrating that the diazirine has significant stability under the Lewis acidic conditions. Increasing the amount of tin(II) chloride to a full equivalent improved the yield to 68%, which has been effective toward producing **11** on a gram-scale.

**Scheme 2:**
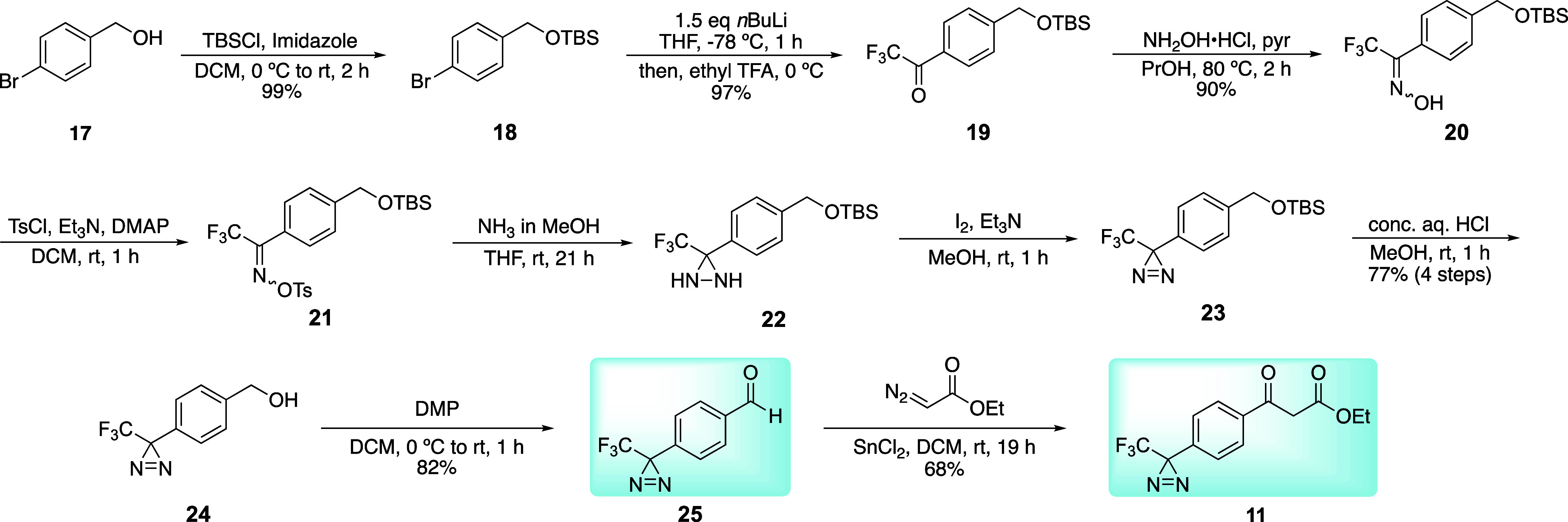
Unified Approach to Diazirine Aza-Biginelli Precursors

With the trifluoromethyl diazirine reactants **11** and **25** in hand, we produced a focused library of DHPs containing the trifluoromethyl diazirine, and it was found to maintain much of the antibacterial potency of the parent compounds, with various probes maintaining MIC values of 4 μg/mL or better (**26a**–**c**, Table 3). To evaluate whether the trifluoromethyl diazirine moiety affords any intrinsic bioactivity, precursors **11** and **25** were also assayed and showed no antibacterial activity at the highest concentrations tested (64 μg/mL). Additionally, we sought to synthesize probes with both alkyne and diazirine functional groups, as alkynes can be used as capture handles after probe photoconjugation. We suspected that the ethyl ester was well suited for replacement with a propargyl ester with a minimal change in the structure. Unfortunately, the synthesis of the propargyl esters of any DHPs was problematic due to competing hydrolysis. Thus, terminal butynyl and terminal pentynyl esters were selected. While the butynyl esters (**26d**,**e**) showed 2-fold decreased potency, the pentynyl esters (**26f**–**h**) showed either 2-fold increased potency (**26f**) or equal potency (**26g**,**h**) compared to their ethyl variants. Satisfyingly, compound **26h** provided the best MIC value of 2 μg/mL while incorporating both a trifluoromethyl diazirine and a terminal alkyne. These probes provide a pathway toward further investigations of the mechanism of antibacterial activity.

**Table 3:**
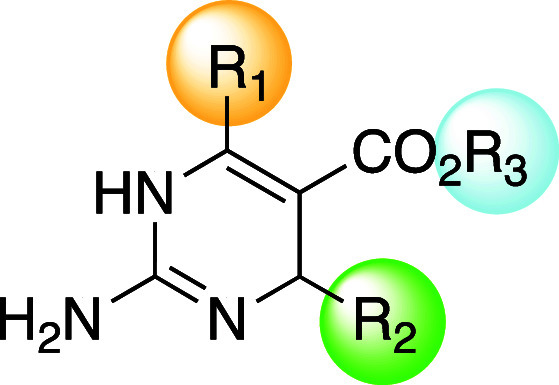
Antibacterial Activity of Diazirine-Functionalized Dihydropyrimidines Measured as Minimum Inhibitory Concentrations (MIC)^a^

	R_1_ (BKE)	R_2_ (RCHO)	R_3_	ATCC 12600	ATCC 43300
**6j**	Ph	3,5-Cl_2_Ph	Et	8	8
**7a**	Ph	4-isobutyl-Ph	Et	4	4
**26a**	Ph	4-CF_3_CN_2_Ph	Et	4	4
**26b**	4-CF_3_CN_2_Ph	3,5-Cl_2_Ph	Et	4	4
**26c**	4-CF_3_CN_2_Ph	4-isobutyl-Ph	Et	2	2
**26d**	Ph	3,5-Cl_2_Ph	(CH_2_)_2_CCH	16	16
**26e**	Ph	4-CF_3_CN_2_Ph	(CH_2_)_2_CCH	8	8
**26f**	Ph	3,5-Cl_2_Ph	(CH_2_)_3_CCH	4	4
**26g**	Ph	4-CF_3_CN_2_Ph	(CH_2_)_3_CCH	4	4
**26h**	4-CF_3_CN_2_Ph	4-isobutyl-Ph	(CH_2_)_3_CCH	2	2
**11**				>64	>64
**25**				>64	>64

a Minimum inhibitory concentrations are defined as μg/mL amounts inhibiting greater than 95% of bacterial growth based on absorbance at 600 nm.

After determining that some of our compounds were quite potent for the inhibition of bacterial growth, we next turned toward assessing broader cytotoxicity trends. The initial lead compound **2** of our prior effort was formerly reported to lack mammalian toxicity.^4^ Further, our previously reported derivatives were also screened for red blood cell (RBC) hemolysis, and the assay indicated a lack of RBC lysis at the highest concentrations tested for all compounds (64 μg/mL).^6^ As the compounds in this report were significantly more potent, we considered that they may generate these undesired off-target effects; therefore, initial efforts examined their hemolytic properties. All but one compound showed no RBC lysis at 64 μg/mL; our most potent compound for bacterial growth inhibition, **7f**, produced >20% hemolysis at 64 μg/mL (Table 4). This indicates it may have secondary mechanisms of cytotoxicity via nonspecific membrane rupturing activity, albeit, at a much higher concentration than its 2 μg/mL MIC. Next, we examined the compounds’ ability to decrease the viability of an immortalized mammalian cell line (HEK293) using a colorimetric MTT assay.^34^ To our surprise, all of the compounds screened produced relatively potent cytotoxicity against this cell line. Indeed, potency trends for bacterial inhibition seemed to be closely matched with the mammalian cytotoxicity trends, where the 2 μg/mL bacterial inhibitor **7f** had the lowest concentration for a cytotoxicity IC_50_ of 0.61 μg/mL, 4 μg/mL bacterial inhibitors (**7a**–**c**, **7e**, and **7h**) averaged a cytotoxicity IC_50_ of 2.7 μg/mL, and 8 μg/mL bacterial inhibitors (**6j**, **6r**, **7g**, and **7i**) averaged a cytotoxicity IC_50_ of 4.1 μg/mL. The trend between bacterial and mammalian growth inhibition breaks down a bit when **6** and **6u** are considered, as neither compound shows significant bacterial inhibition at 64 μg/mL, but both have similar mammalian cytotoxicity potency (average IC_50_ = 5.1 μg/mL) when compared to the more potent bacterial inhibitors. Of further note, our prior study demonstrated that the guanidine was essential for antibacterial activity of the previously reported DHPs.^6^ We screened a dihydropyrimidine where the guanidine was substituted with a urea (traditional Biginelli reaction derived), and this compound had no bacterial inhibition but continued to have significant mammalian cytotoxicity, further decoupling the antibacterial activity from mammalian cytotoxicity in these cases. Future studies involving the photoaffinity probes will examine the mechanism by which our compounds exert their growth-inhibiting effects in both bacterial and mammalian cells.

**Table 4:**
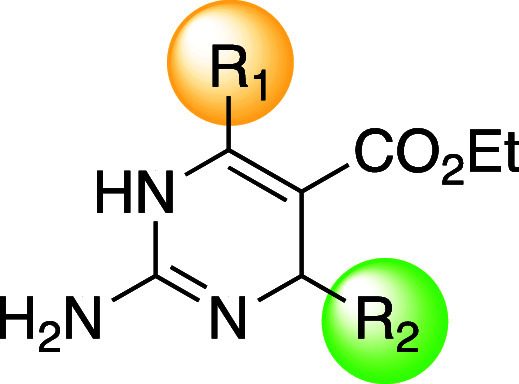
Comparison of the Compounds’ Hemolytic Activity Probed via Lysis of Red Blood Cells, Mammalian Toxicity of Compounds in MTT Assay Examining HEK293 Cell Viability, and Bacterial Inhibition of MRSA

			lysis_20_	HEK293 viability		ATCC 43300 MIC
	R_1_	R_2_	μg/mL	μM	μg/mL	μg/mL
**6**	Ph	Ph	>64	18	5.8	>64
**6i**	Ph	3-Cl-Ph	>64	18	5.8	32
**6j**	Ph	3,5-Cl_2_-Ph	>64	11	3.5	8
**6r**	Ph	4-iPr-Ph	>64	9.5	3.1	8
**6s**	Ph	2-OCH_3_-Ph	>64	87	28	>64
**6u**	Ph	4-OCH_3_-Ph	>64	14	4.5	>64
**7a**	Ph	4-isobutyl-Ph	>64	7.9	2.5	4
**7b**	Ph	4-Ph-Ph	>64	9.4	3.0	4
**7c**	Et	4-isobutyl-Ph	>64	11	3.5	4
**7e**	3-CF_3_-Ph	3,5-Cl_2_-Ph	>64	9.9	3.2	4
**7f**	3-CF_3_-Ph	4-isobutyl-Ph	64	1.9	0.61	2
**7g**	3-F-Ph	3,5-Cl_2_-Ph	>64	14	4.5	8
**7h**	3-F-Ph	4-isobutyl-Ph	>64	4.4	1.4	4
**7i**	3-CH_3_-Ph	3,5-Cl_2_-Ph	>64	16	5.1	8

We explored the utility of a class of functionalized dihydropyrimidines (DHPs) as antibacterial agents. This involved systematically evaluating the SAR, leading to compound **7f**, which inhibited the growth of all *S. aureus* strains at 2 μg/mL. Oxidation of DHPs to pyrimidines, which have the same core ring structure as FDA approved dihydrofolate reductase (DHFR) inhibitors, was completed to analyze whether DHPs or their related pyrimidines could exert their antibacterial effect via DHFR inhibition, as this mechanism had been previously proposed. After this was found to be inoperative, DHP photoaffinity probes were synthesized and were found to retain their strong antibacterial activity. Despite the promising antibacterial effects and lack of hemolytic activity, the DHPs also displayed broad mammalian cytotoxicity at relevant concentrations. Future efforts to explore the mechanism will additionally attempt to uncouple the antibacterial effect from mammalian cytotoxicity.

## Supporting Information Available

Some supporting tables involved in the chemical synthesis optimization. Full details of chemical synthesis including full characterization. Descriptions of the biological assays. NMR spectra of all final compounds and important intermediates (PDF)

## Abbreviations

AR: antibiotic resistant
MRSA: methicillin-resistant *Staphylococcus aureus*
DHFR: dihydrofolate reductase
DHP: dihydropyrimidine
SAR: structure–activity relationships
FDA: Food and Drug Administration
MIC: minimum inhibitory concentration
PIDA: phenyliodine diacetate
SaDHFR: *Staphylococcus aureus* dihydrofolate reductase
PAL: photoaffinity labeling
DMP: Dess–Martin periodinane
