# Supplementary material for: Evaluation of Antibacterial Functionalized Dihydropyrimidine Photoaffinity Probes Toward Mechanism of Action Studies
Source: ACS Med Chem Lett. 2024 Jun 20;15(7):1094–101. doi: 10.1021/acsmedchemlett.4c00173 (PMC11247627; doi:10.1021/acsmedchemlett.4c00173)
Supplement: Supplementary file 1 — ml4c00173_si_001.pdf [file ml4c00173_si_001.pdf]

Evaluation of Antibacterial Functionalized Dihydropyrimidine Photoaffinity Probes Toward Mechanism of Action Studies

Christopher M. Russo,[a] Zachary W. Boyer,[a] Kaitlyn Scheunemann,[b] Jonathan Farren,[b] Alexandra Minich,[c] Cody J. Wenthur,[c] and Matthew C. O'Reilly[a]*

[a]Department of Chemistry, Villanova University, Villanova, Pennsylvania 19085, United States

[b]Department of Chemistry and Biotechnology, University of Wisconsin–River Falls, River Falls, Wisconsin, 54022, United States

[c]School of Pharmacy, University of Wisconsin–Madison, Madison, Wisconsin, 53705, United States

Corresponding Author Information

*Corresponding author: Matthew C. O'Reilly – Department of Chemistry, Villanova University. ORCID: 0000-0002-9175-4179. Email: matthew.oreilly@villanova.edu

Table of Contents:

1. Supporting tables in order of their appearance in the article—*page 2*
2. Details of chemical synthesis and characterization—*page 3*
3. Details of biological assays
    a. Antibacterial microdilution assay (MIC determination)—*page 64*
    b. Hemolysis assay—*page 65*
    c. Cytotoxicity MTT assay—*page 66*
4. NMR spectra—*page 67*
5. References—*page 192*

Supporting Information Figures:

Table S1: Optimization of the Roskamp Reaction. EDA = Ethyl Diazoacetate

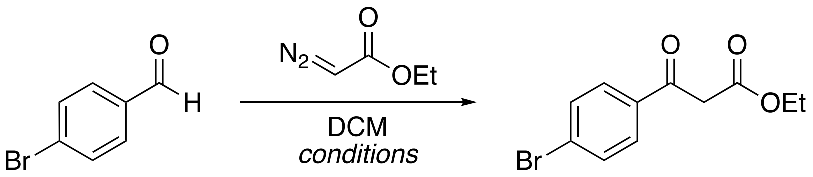

S1 **S2**

Entry	Catalyst	Loading (mol%)	Eq. EDA	Time (h)	Temp (°C)	Prod:SM	Notes
1	$SnCl_2$	5	1.2	17	rt	15:85	-
2	$NbCl_5$	5	1.2	17	rt	0:100	-
3	$MoCl_2O_2$	5	1.2	17	rt	14:86	-
4	$SnCl_2$	50	1.2	19	rt	60:40	-
5	$NbCl_5$	50	1.2	19	rt	7:93	-
6	$MoCl_2O_2$	50	1.2	19	rt	10:90	-
7	$SnCl_2$	100	1.2	21	rt	68:32	-
8	$ZnCl_2$	100	1.2	21	rt	31:69	Side-product(s)
9	BF_3	100	1.2	18	0 to rt	-	Complicated mixture
10	$SnCl_2$	100	1.2	21	rt	70:30	Slow-addition of EDA
**11**	**$SnCl_2$**	**50**	**2.4**	**21**	**rt**	**100:0**	**52% isolated**
12	BF_3	10	2.4	4	0 to rt	-	Complicated mixture
13	BF_3	10	1.2	1	0	-	Complicated mixture
14	$SnCl_4$	10	2.4	17	-78 to rt	-	Complicated mixture
**15**	**$SnCl_2$**	**100**	**2.4**	**23**	**rt**	**92:8**	**52% isolated**

Table S2: Results of the reactions of aldehyde **25** with 2.4 eq EDA and $SnCl_2$ at room temperature.

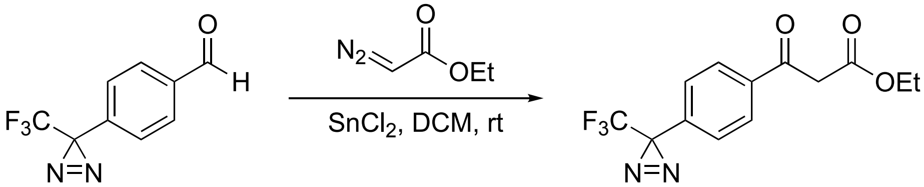

Entry	Catalyst Loading (mol%)	Scale (mmol)	Time (h)	Product Yield
1	50	1.00	22	59%
2	50	0.88	24	53%
3	50	1.00	28	66%
4	100	10.0	19	68%
5	100	1.00	21	63%
6	100	1.00	23	77%
7	100	1.00	25	72%

General Synthesis Considerations

All reagents were purchased from commercial suppliers and were used without additional purification. Thin-layer chromatography (TLC) was performed on TLC Silica gel 60 F_{254} from Supelco. Visualization was accomplished via UV light or iodine, *p*-anisaldehyde, or potassium permanganate staining. Flash chromatography was performed using normal phase Silica RediSep Silver Rf flash columns on a CombiFlash Rf automated flash chromatography system. All ^{1}H, ^{19}F, and ^{13}C NMR spectra were recorded on a 400 MHz or 500 MHz JEOL spectrometer. Chemical shifts for ^{1}H and ^{13}C spectra are reported in ppm relative to residual solvent peaks as an internal standard set to δ 7.26 and δ 77.16 ($CDCl_3$), δ 3.31 and δ 49.00 (Methanol-D_4), or δ 2.50 and δ 39.52 (DMSO-D_6). Data are reported as follows: chemical shift, multiplicity (s = singlet, d = doublet, t = triplet, q = quartet, p = pentet, sx = sextet, sp = septet, br = broad, dd = doublet of doublets, dq = doublet of quartets, td = triplet of doublets, pd = pentet of doublets, m = multiplet), coupling constant (Hz), integration. High resolution mass spectra (HRMS) were recorded on an AB SciEx 5600+ TripleTOF using electrospray ionization in positive mode. Analytical high performance liquid chromatography (HPLC) was performed on a Shimadzu analytical HPLC with UV detection at 221 nm and 254 nm along with full spectrum UV detection. No unexpected or unusually high safety hazards were encountered during the work.

NMR Solvents found in spectra:

- Functionalized dihydropyrimidine products were analyzed in DMSO-D_6, and this includes compounds **6a-w**, **7a-j**, and **26a-h**. These NMR spectra had the DMSO signal at δ 2.50 and a water signal around δ 3.33 ppm.
- Other compounds were analyzed in $CDCl_3$, and this includes compounds **8a**, **10**, **11**, **18**-**24**, **S3**, **S4**, **S5**, **S6**, **S7**, and **S8**. These NMR spectra had the $CDCl_3$ signal at δ 7.26 ppm.
- Compound **S4** was analyzed in MeOD-D_4. This NMR spectrum had the MeOD signal at δ 3.31 and a water signal at δ 4.87 ppm.

Compound Synthesis

General aza-Biginelli reaction procedure:

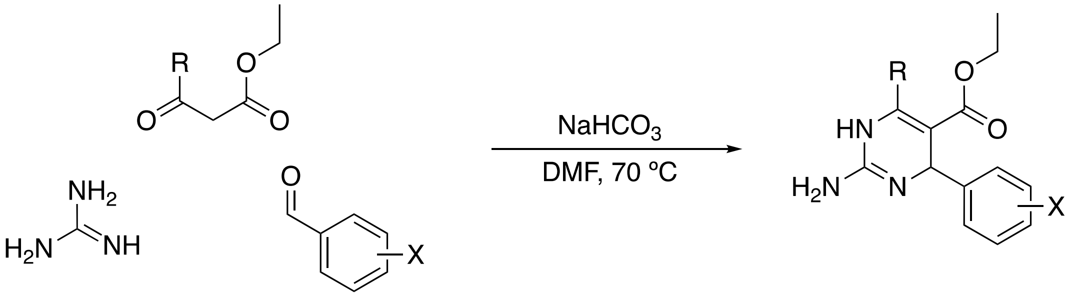

A flame- or oven-dried round-bottomed flask equipped with a magnetic stir bar was charged with all solid reagents, usually including a substituted benzaldehyde[1] (2.62 mmol, 1.00 eq), guanidine carbonate (3.14 mmol, 1.2 eq), and sodium bicarbonate (10.48 mmol, 4.0 eq). It was then sealed with a rubber septum and purged with argon. Anhydrous DMF (5.24 mL, 0.5 M with respect to the limiting reagent) was added via syringe, and the reaction was placed in an oil bath heated to 70 °C, and the β-keto ester (2.88 mmol, 1.10 eq) was added via syringe. The reaction was allowed to stir for 12-18 hours at 70 °C. The reaction was removed from the oil bath and was allowed to cool to room temperature, at which point it was poured into a beaker of crushed ice. The round-bottomed flask was washed with water to ensure all solids were transferred to the beaker, and the beaker was swirled or stirred to ensure the reaction components were successfully mixed, which caused a large amount of yellow/orange material to precipitate from solution. The solid was isolated using vacuum filtration, the filtercake was washed with 100 mL of additional water, and it was dried. In cases where the R-group of the β-keto ester was an arene (most analogues), DCM was added to the filtercake material, which had been transferred to an Erlenmeyer flask. The suspension was swirled to allow any soluble material to go into solution, and the remaining solid was isolated using vacuum filtration. In cases when this did not produce pure desired product, column chromatography (ethyl acetate/methanol) was performed to complete purification, and those details are described in the specific reactions below. After drying under reduced pressure, characterization was performed.[2]

[1]In cases where the benzaldehyde was a liquid, it was added via syringe after the addition of the anhydrous DMF.

[2]In all cases, high resolution mass spectrometry confirmed the molecular formula with less than 5 ppm of error. ^{1}H NMR and ^{13}C NMR provided proof of identity.

Aza-Biginelli Reaction Products (pages 5-38):

Preparation of **6.**

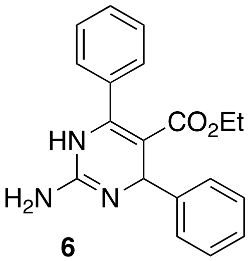

6

6 was prepared according to the general aza-Biginelli reaction procedure on a 9.43 mmol scale. After precipitation on ice water according to the general procedure, the dried crude product was triturated with dichloromethane to afford the desired product (1.267 g, 42% yield). ^{1}H NMR (500 MHz, room temperature, DMSO-D_6): 7.60 (s, 1H), 7.40-7.37 (m, 2H), 7.36-7.32 (m, 2H), 7.28-7.22 (m, 6H), 6.37 (s, br, 2H), 5.31 (s, 1H), 3.68 (q, J = 7.1 Hz, 2H), 0.74 (t, J = 7.1 Hz, 3H). ^{13}C NMR (101 MHz, room temperature, DMSO-D6): 166.22, 161.34, 155.53, 146.30, 142.67, 128.31, 128.10, 127.06, 126.99, 126.83, 126.32, 97.41, 58.06, 52.83, 13.66. HRMS (ESI^{+}), $C_{19}H_{19}N_3O_2^{+}$ [M+H]$^{+}$: calc. mass 322.1550, found 322.1552.

Preparation of **6a**.

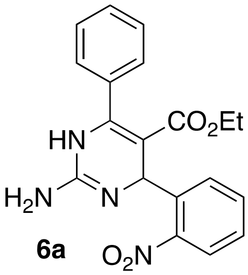

6a

6a was prepared according to the general aza-Biginelli reaction procedure on a 10.37 mmol scale. After precipitation on ice water according to the general procedure, the dried crude product was triturated with dichloromethane to afford the desired product as a light yellow solid (1.894 g, 55% yield). ^{1}H NMR (400 MHz, DMSO-D_6, room temperature) δ 7.96 (dd, J = 8.2, 1.3 Hz, 1H), 7.85 – 7.70 (m, 2H), 7.73 (dd, J = 8.2, 1.3 Hz, 1H); 7.55 (td, J = 7.4, 1.5 Hz, 1H), 7.50 (s, 1H), 7.32-7.28 (m, 5H), 6.45 (s, 2H), 5.70 (s, 1H), 3.57 (qd, J = 7.1, 1.7 Hz, 2H), 0.64 (t, J = 7.1 Hz, 3H). ^{13}C NMR (101 MHz, room temperature, DMSO-D6): δ 165.83, 163.08, 155.90, 147.95, 142.48, 140.11, 135.04, 129.82, 129.35, 128.44, 127.80, 127.52, 124.46, 96.49, 58.72, 49.31, 13.98. HRMS (ESI$^+$), $C_{19}H_{19}N_4O_4^+$ $[M+H]^+$: calc. mass 367.1401, found 367.1402.

Preparation of **6b**.

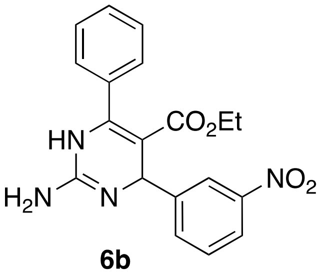

6b

6b was prepared according to the general aza-Biginelli reaction procedure on a 10.37 mmol scale. After precipitation on ice water according to the general procedure, the dried crude product was triturated with dichloromethane to afford the desired product as a pale-yellow solid (1.172 g, 34% yield). ^{1}H NMR (400 MHz, DMSO-D_6, room temperature) δ 8.24-8.22 (m, 1H), 8.14 (dd, J = 8.2, 1.8 Hz, 1H), 7.83 (d, J = 8.0 Hz, 1H), 7.78-7.60 (m, 2H), 7.30-7.22 (m, 5H), 6.52 (s, br, 2H), 5.46 (s, 1H), 3.70 (q, J = 7.0 Hz, 2H), 0.75 (t, J = 7.1 Hz, 3H). ^{13}C NMR (101 MHz, room temperature, DMSO-D6): δ 166.09, 162.29, 155.55, 148.35, 147.83, 142.27, 133.08, 130.14, 128.17, 127.29, 126.91, 122.16, 120.95, 96.35, 58.29, 52.13, 13.67. HRMS (ESI^{+}), $C_{19}H_{19}N_4O_4^{+}$ [M+H]$^{+}$: calc. mass 367.1401, found 367.1402.

Preparation of **6c**.

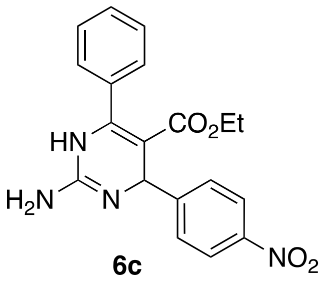

6c

6c was prepared according to the general aza-Biginelli reaction procedure on a 10.37 mmol scale. After precipitation on ice water according to the general procedure, the dried crude product was triturated with dichloromethane to afford the desired product as a pale-yellow solid (0.412 g, 12% yield). ^{1}H NMR (400 MHz, DMSO-D_6, room temperature) δ 8.25 (d, J = 8.7 Hz, 2H), 7.78 – 7.52 (m, 3H), 7.31-7.21 (m, 5H), 6.51 (s, 2H), 5.44 (s, 1H), 3.69 (q, J = 7.1 Hz, 2H), 0.74 (t, J = 7.1 Hz, 3H). ^{13}C NMR (101 MHz, room temperature, DMSO-D6): δ 166.58, 162.70, 156.09, 153.99, 147.16, 142.81, 128.69, 128.06, 127.78, 127.39, 124.32, 96.89, 58.80, 52.93, 14.18. HRMS (ESI$^+$), $C_{19}H_{19}N_4O_4^+$ [M+H]$^+$: calc. mass 367.1401, found 367.1395.

Preparation of **6d**.

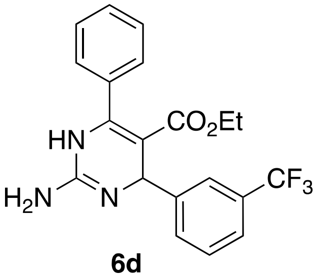

6d

6d was prepared according to the general aza-Biginelli reaction procedure on a 10.37 mmol scale. After precipitation on ice water according to the general procedure, the dried crude product was triturated with dichloromethane to afford the desired product as a pale-yellow solid (1.729 g, 50% yield). ^{1}H NMR (400 MHz, DMSO-D_6, room temperature) δ 7.72 – 7.57 (m, 5H), 7.30 – 7.19 (m, 5H), 6.47 (s, br, 2H), 5.40 (s, 1H), 3.69 (qd, J = 7.1, 1.8 Hz, 2H), 0.74 (t, J = 7.1 Hz, 3H). ^{13}C NMR (101 MHz, room temperature, DMSO-D6): δ 166.11, 162.02, 155.58, 147.59, 142.39, 130.44, 129.65, 128.98 (q, J = 31.3 Hz), 128.11, 127.22, 126.92, 124.38 (q, J = 272.3 Hz), 123.87 (q, J = 4.1 Hz), 122.87 (q, J = 3.8 Hz), 96.69, 58.22, 52.41, 13.63. HRMS (ESI$^+$), $C_{20}H_{19}F_3N_3O_2^+$ $[M+H]^+$: calc. mass 390.1424, found 390.1433.

Preparation of **6e**.

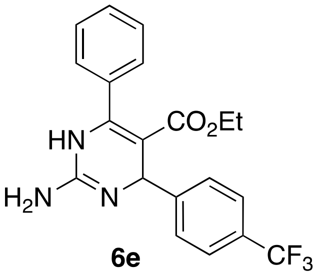

6e

6e was prepared according to the general aza-Biginelli reaction procedure on a 9.35 mmol scale. After precipitation on ice water according to the general procedure, the dried crude product was triturated with dichloromethane to afford the desired product as a pale-yellow solid (1.455 g, 44% yield). ^{1}H NMR (400 MHz, DMSO-D_6, room temperature) δ 7.73 (d, J = 8.1 Hz, 2H), 7.69-7.51 (m, 3H), 7.30-7.21 (m, 5H), 6.42 (s, br, 2H), 5.39 (s, 1H), 3.69 (q, J = 7.1 Hz, 2H), 0.74 (t, J = 7.1 Hz, 3H). ^{13}C NMR (101 MHz, room temperature, DMSO-D6): δ 166.12, 161.94, 155.52, 150.69, 142.41, 128.15, 127.72 (q, J = 31.8 Hz), 127.16, 127.07, 126.83, 125.37 (q, J = 3.8 Hz), 124.37 (q, J = 271.9 Hz) 96.72, 58.19, 52.49, 13.65. HRMS (ESI$^+$), $C_{20}H_{19}F_3N_3O_2^+$ $[M+H]^+$: calc. mass 390.1424, found 390.1418.

Preparation of **6f**.

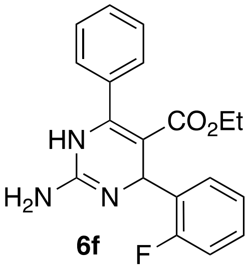

6f

6f was prepared according to the general aza-Biginelli reaction procedure on a 10.37 mmol scale. After precipitation on ice water according to the general procedure, the dried crude product was triturated with dichloromethane to afford the desired product as a pale-yellow solid (1.850 g, 48% yield). ^{1}H NMR (400 MHz, DMSO-D_6, room temperature) δ 7.48 (s, br, 1H), 7.41 (td, J = 7.5, 1.7 Hz, 1H), 7.36-7.26 (m, 6H), 7.23-7.16 (m, 2H), 6.25 (s, br, 2H), 5.63 (s, 1H), 3.64 (q, J = 7.2 Hz, 2H), 0.71 (t, J = 7.1 Hz, 3H). ^{13}C NMR (101 MHz, room temperature, DMSO-D6): δ 165.72, 162.11, 159.22 (d, J = 241.3 Hz), 155.23, 142.38, 132.24 (d, J = 14.4 Hz), 129.28 (d, J = 10.3 Hz), 128.58 (d, J = 4.6 Hz), 128.02, 127.13, 126.95, 124.74 (d, J = 3.3 Hz), 115.35 (d, J = 22.0 Hz), 95.22, 58.12, 46.71, 13.60. HRMS (ESI$^+$), $C_{19}H_{19}FN_3O_2^+$ [M+H]$^+$: calc. mass 340.1456, found 340.1449.

Preparation of **6g**.

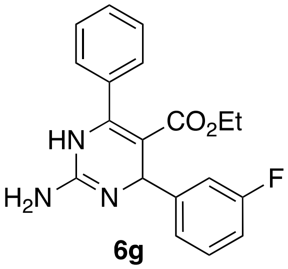

6g

6g was prepared according to the general aza-Biginelli reaction procedure on a 10.37 mmol scale. After precipitation on ice water according to the general procedure, the dried crude product was triturated with dichloromethane to afford the desired product as a pale-yellow solid (1.612 g, 50% yield). ^{1}H NMR (400 MHz, DMSO-D_6, room temperature) δ 7.57 (s, br, 1H), 7.43-7.36 (m, 1H), 7.31 – 7.19 (m, 6H), 7.16 – 7.04 (m, 2H), 6.41 (s, 2H), 5.33 (s, 1H), 3.70 (q, *J* = 7.1 Hz, 2H), 0.74 (t, *J* = 7.1 Hz, 3H). ^{13}C NMR (101 MHz, room temperature, DMSO-D6): δ 166.70, 162.74 (d, J = 245.9 Hz), 161.96 155.94, 149.66 (d, J = 5.5 Hz), 142.79, 130.90 (d, J = 8.0 Hz), 128.65, 127.72, 127.43, 122.76 (d, J = 2.9 Hz) 114.36 (d, J = 21.3 Hz), 113.38 (d, J = 19.7 Hz), 97.52, 58.74, 52.87, 14.18. HRMS (ESI$^+$), $C_{19}H_{19}FN_3O_2^+$ [M+H]$^+$: calc. mass 340.1456, found 340.1467.

Preparation of **6h**.

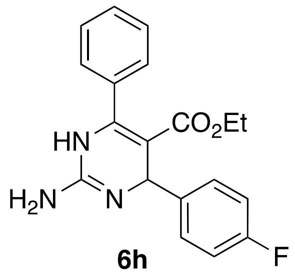

6h

6h was prepared according to the general aza-Biginelli reaction procedure on a 10.37 mmol scale. After precipitation on ice water according to the general procedure, the dried crude product was triturated with dichloromethane to afford the desired product as a pale-yellow solid (1.503 g, 47% yield). ^{1}H NMR (400 MHz, DMSO-D_6, room temperature) δ 7.50 (s, br, 1H), 7.43 – 7.34 (m, 2H), 7.30 – 7.20 (m, 5H), 7.20 – 7.13 (m, 2H), 6.33 (s, br, 2H), 5.31 (s, 1H), 3.68 (q, J = 7.1 Hz, 2H), 0.73 (t, J = 7.1 Hz, 3H). ^{13}C NMR (126 MHz, room temperature, DMSO-D6): δ 166.17, 161.39, 161.34 (d, J = 239.2 Hz), 155.35, 142.54, 128.24 (d, J = 7.5 Hz), 128.12, 127.10, 126.88, 115.05 (d, J = 21.7 Hz), 97.40, 58.14, 52.21, 13.67 (one set of coincident signals). HRMS (ESI$^+$), $C_{19}H_{19}FN_3O_2^+$ [M+H]$^+$: calc. mass 340.1456, found 340.1451.

Preparation of **6i**.

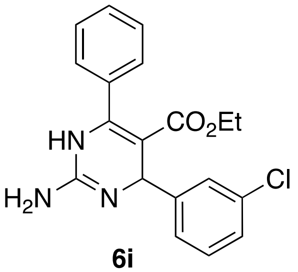

6i

6i was prepared according to the general aza-Biginelli reaction procedure on a 9.425 mmol scale. After precipitation on ice water according to the general procedure, the dried crude product was triturated with dichloromethane to afford the desired product as a pale-yellow solid (1.486 g, 44% yield). ^{1}H NMR (400 MHz, room temperature, DMSO-*D*$_{6}$): 7.45-7.26 (m, 5H), 7.25-7.18 (m, 5H), 6.59 (s, br, 2H), 5.29 (s, 1H), 3.67 (q, J = 7.1 Hz, 2H), 0.71 (t, 7.1 Hz, 3H). ^{13}C NMR (101 MHz, room temperature, DMSO-D6): 166.13, 161.75, 155.45, 148.65, 142.34, 132.90, 130.39, 128.11, 127.19, 126.99, 126.88, 126.17, 124.93, 96.79, 58.19, 52.36, 13.65. HRMS (ESI^{+}), $C_{19}H_{19}ClN_3O_2^{+}$ [M+H]$^{+}$: calc. mass 356.1160, found 356.1157.

Preparation of **6j**.

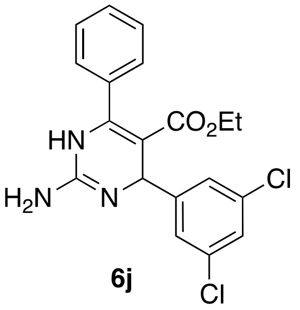

6j

6j was prepared according to the general aza-Biginelli reaction procedure on a 10.37 mmol scale. After precipitation on ice water according to the general procedure, the dried crude product was triturated with dichloromethane to afford the desired product as a pale-yellow solid (1.041 g, 28% yield). ^{1}H NMR (400 MHz, DMSO-D_6, room temperature) δ δ 7.65 (s, br, 1H), 7.51 (t, J = 2.0 Hz, 1H), 7.43-7.30 (m, 3H), 7.29-2.19 (m, 5H), 6.52 (s, br, 2H), 5.33 (s, 1H), 3.72 (qd, J = 7.1, 1.5 Hz, 2H), 0.76 (t, J = 7.1 Hz, 3H). ^{13}C NMR (101 MHz, room temperature, DMSO-D6): δ 166.09, 162.22, 155.51, 150.21, 141.13, 134.00, 128.14, 127.36, 126.93, 126.73, 125.04, 96.07, 58.32, 51.99, 13.65. HRMS (ESI$^+$), $C_{19}H_{18}Cl_2N_3O_2^+$ [M+H]$^+$: calc. mass 390.0771, found 390.0769.

Preparation of **6k**.

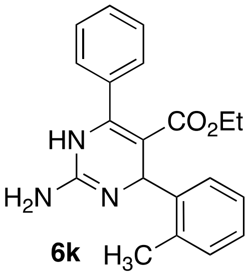

6k

6k was prepared according to the general aza-Biginelli reaction procedure on a 9.16 mmol scale. After precipitation on ice water according to the general procedure, the dried crude product was triturated with dichloromethane to afford the desired product as a pale-yellow solid (1.746 g, 62% yield). ^{1}H NMR (400 MHz, DMSO-D_6, room temperature) δ 7.41 (d, J = 7.4, 1H), 7.34 (s, br, 1H), 7.31-7.24 (m, 5H), 7.22 – 7.10 (m, 3H), 6.12 (s, 2H), 5.54 (s, 1H), 3.61 (q, J = 7.1 Hz, 2H), 2.47 (s, 3H), 0.68 (t, J = 7.1 Hz, 3H). ^{13}C NMR (101 MHz, room temperature, DMSO-D6): δ 165.98, 161.48, 155.05, 144.20, 142.81, 134.20, 130.08, 127.94, 127.20, 127.04, 126.85, 126.52, 97.47, 57.98, 49.50, 38.89, 18.74, 13.58. HRMS (ESI$^+$), $C_{20}H_{22}N_3O_2^+$ [M+H]$^+$: calc. mass 336.1707, found 336.1709.

Preparation of **6l**.

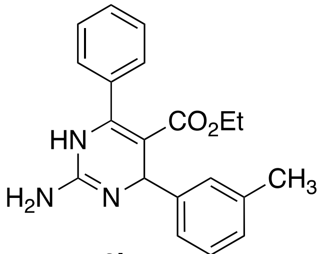

6l

6l was prepared according to the general aza-Biginelli reaction procedure on a 9.16 mmol scale. After precipitation on ice water according to the general procedure, the dried crude product was triturated with dichloromethane to afford the desired product as a pale-yellow solid (1.096 g, 39% yield). ^{1}H NMR (400 MHz, DMSO-D_6, room temperature) δ 7.44 (s, br, 1H), 7.29 – 7.12 (m, 8H), 7.08 – 7.03 (m, 1H), 6.26 (s, br, 2H), 5.26 (s, 1H), 3.67 (q, J = 7.1 Hz, 2H), 2.30 (s, 3H), 0.74 (t, J = 7.1 Hz, 3H). ^{13}C NMR (101 MHz, room temperature, DMSO-D6): δ 166.20, 161.26, 155.39, 146.24, 142.70, 137.19, 128.27, 128.08, 127.70, 126.95, 126.93, 126.81, 123.45, 97.40, 58.03, 52.86, 21.23, 13.66. HRMS (ESI$^+$), $C_{20}H_{22}N_3O_2^+$ [M+H]$^+$: calc. mass 336.1707, found 336.1706.

Preparation of **6m**.

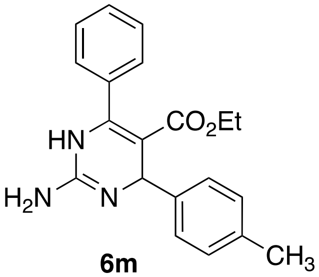

6m

6m was prepared according to the general aza-Biginelli reaction procedure on a 9.35 mmol scale. After precipitation on ice water according to the general procedure, the dried crude product was triturated with dichloromethane to afford the desired product as a pale-yellow solid (1.096 g, 38% yield). ^{1}H NMR (400 MHz, DMSO-D_6, room temperature) δ 7.43 (s, br, 1H), 7.29-7.20 (m, 7H), 7.13 (d, J = 7.9 Hz, 2H), 6.26 (s, br, 2H), 5.25 (s, 1H), 3.67 (q, J = 7.1 Hz, 2H), 2.27 (s, 3H), 0.74 (t, J = 7.1 Hz, 3H). ^{13}C NMR (101 MHz, room temperature, DMSO-D6): δ 166.23, 161.13, 155.43, 143.40, 142.71, 136.14, 128.84, 128.10, 126.95, 126.81, 126.23, 97.64, 58.04, 52.56, 20.71, 13.68. HRMS (ESI$^+$), $C_{20}H_{22}N_3O_2^+$ [M+H]$^+$: calc. mass 336.1707, found 336.1709.

Preparation of **6n**.

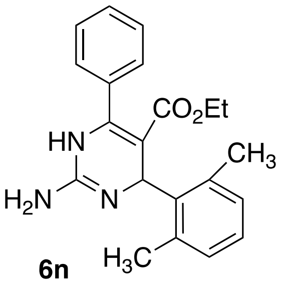

6n

6n was prepared according to the general aza-Biginelli reaction procedure on a 9.35 mmol scale. After precipitation on ice water according to the general procedure, the dried crude product was triturated with dichloromethane to afford the desired product as a pale-yellow solid (0.312 g, 10.5% yield). ^{1}H NMR (400 MHz, DMSO-D_6, room temperature) δ 7.28 – 7.21 (m, 3H), 7.21 – 7.17 (m, 2H), 7.13 (s, br, 1H), 7.04-6.94 (m, 3H), 6.05 (s, 1H), 5.96 (s, br, 2H), 3.51 (qd, J = 7.1, 4.6 Hz, 2H), 2.48 (s, 6H), 0.56 (t, J = 7.1 Hz, 3H). ^{13}C NMR (101 MHz, room temperature, DMSO-D6): δ 166.93, 160.30, 155.34, 143.62, 141.68, 137.54, 127.89, 127.38, 127.19, 127.10, 96.11, 58.32, 50.81, 20.07, 13.80 (one set of coincident signals). HRMS (ESI$^+$), $C_{21}H_{24}N_3O_2^+$ [M+H]$^+$: calc. mass 350.1863, found 350.1862.

Preparation of **6o**.

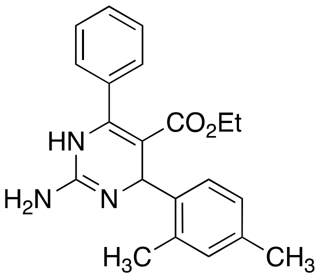

6o

6o was prepared according to the general aza-Biginelli reaction procedure on a 9.35 mmol scale. After precipitation on ice water according to the general procedure, the dried crude product was triturated with dichloromethane to afford the desired product as a pale-yellow solid (1.456 g, 49% yield). ^{1}H NMR (400 MHz, DMSO-D_6, room temperature) δ 7.31-7.22 (m, 7H), 7.01 – 6.97 (m, 1H), 6.95 (s, br, 1H), 6.08 (s, br, 2H), 5.49 (s, 1H), 3.60 (q, J = 7.1 Hz, 2H), 2.42 (s, 3H), 2.23 (s, 3H), 0.69 (t, J = 7.1 Hz, 3H). ^{13}C NMR (101 MHz, room temperature, DMSO-D6): δ 166.03, 161.27, 155.04, 142.85, 141.34, 136.02, 134.01, 130.70, 127.96, 127.23, 127.06, 126.86, 97.63, 57.99, 49.24, 20.61, 18.69, 13.62 (one set of coincident signals). HRMS (ESI$^+$), $C_{21}H_{24}N_3O_2^+$ [M+H]$^+$: calc. mass 350.1863, found 350.1868.

Preparation of **6p**.

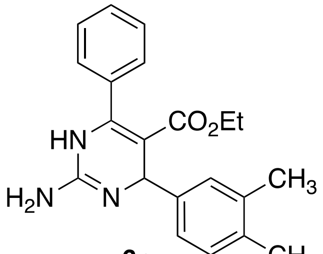

6p

6p was prepared according to the general aza-Biginelli reaction procedure on a 9.35 mmol scale. After precipitation on ice water according to the general procedure, the dried crude product was triturated with dichloromethane to afford the desired product as a pale-yellow solid (1.225 g, 41% yield). ^{1}H NMR (400 MHz, DMSO-D_6, room temperature) δ 7.45 (s, br, 1H), 7.28-7.20 (m, 5H), 7.13 – 7.04 (m, 3H), 6.23 (s, br, 2H), 5.22 (s, 1H), 3.66 (q, J = 7.1 Hz, 2H), 2.20 (s, 3H), 2.18 (s, 3H), 0.74 (t, J = 7.1 Hz, 3H). ^{13}C NMR (101 MHz, room temperature, DMSO-D6): δ 166.25, 161.11, 155.40, 143.77, 142.78, 135.76, 134.87, 129.42, 128.10, 127.54, 126.94, 126.83, 123.72, 58.04, 52.61, 19.69, 19.07, 13.70 (one set of coincident signals). HRMS (ESI$^+$), $C_{21}H_{24}N_3O_2^+$ [M+H]$^+$: calc. mass 350.1863, found 350.1866.

Preparation of **6q**.

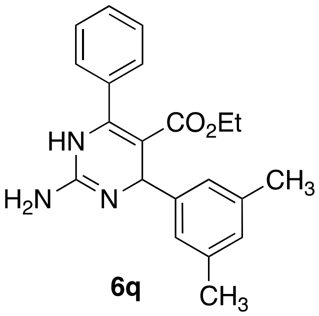

6q

6q was prepared according to the general aza-Biginelli reaction procedure on a 2.05 mmol scale. After precipitation on ice water according to the general procedure, the dried crude product was triturated with dichloromethane to afford the desired product as a pale-yellow solid (0.371g, 57% yield). ^{1}H NMR (400 MHz, DMSO-D_6, room temperature) δ 7.41 (s, br, 1H), 7.30 – 7.20 (m, 5H), 6.96 (s, 2H), 6.88 (s, 1H), 6.22 (s, br, 2H), 5.23 (s, 1H), 3.67 (q, J = 7.1 Hz, 2H), 2.25 (s, 6H), 0.74 (t, J = 7.1 Hz, 3H). ^{13}C NMR (101 MHz, room temperature, DMSO-D6): δ 166.20, 161.14, 155.30, 146.24, 142.71, 137.11, 128.49, 128.07, 126.96, 126.84, 124.15, 97.41, 58.03, 52.94, 21.14, 13.67. HRMS (ESI$^+$), $C_{21}H_{24}N_3O_2^+$ $[M+H]^+$: calc. mass 350.1863, found 350.1868.

Preparation of **6r**.

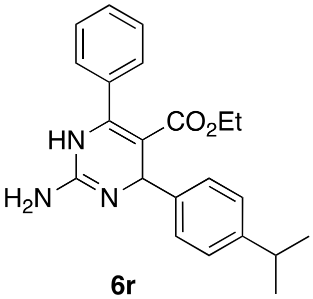

6r

6r was prepared according to the general aza-Biginelli reaction procedure on a 9.35 mmol scale. After precipitation on ice water according to the general procedure, the dried crude product was triturated with dichloromethane to afford the desired product as a pale-yellow solid (1.196 g, 39% yield). ^{1}H NMR (400 MHz, DMSO-D_6, room temperature) δ 7.45 (s, br, 1H), 7.34 – 7.15 (m, 9H), 6.25 (s, br, 2H), 5.25 (s, 1H), 3.67 (q, J = 7.1 Hz, 2H), 2.86 (sp, J = 6.9 Hz, 1H), 1.19 (d, J = 6.9 Hz, 6H), 0.73 (t, J = 7.1 Hz, 3H). ^{13}C NMR (101 MHz, room temperature, DMSO-D6): δ 166.25, 161.19, 155.43, 147.19, 143.81, 142.73, 128.09, 126.93, 126.79, 126.29, 126.20, 97.58, 58.04, 52.60, 33.15, 23.95, 13.65. HRMS (ESI$^+$), $C_{22}H_{26}N_3O_2^+$ [M+H]$^+$: calc. mass 364.2020, found 364.2015.

Preparation of **6s**.

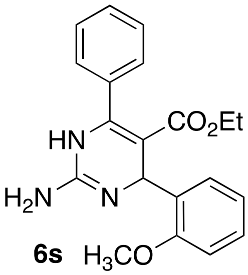

6s

6s was prepared according to the general aza-Biginelli reaction procedure on a 10.37 mmol scale. After precipitation on ice water according to the general procedure, the dried crude product was triturated with dichloromethane to afford the desired product as a pale-yellow solid (1.817 g, 55% yield). ^{1}H NMR (400 MHz, DMSO-D_6, room temperature) δ 7.35 – 7.21 (m, 7H), 7.08 – 7.01 (m, 2H), 6.93 (td, J = 7.5, 1.1 Hz, 1H), 6.20 (s, br, 2H), 5.61 (d, J = 2.3 Hz, 1H), 3.88 (s, 3H), 3.64 (q, J = 7.1 Hz, 2H), 0.69 (t, J = 7.1 Hz, 3H). ^{13}C NMR (101 MHz, room temperature, DMSO-D6): δ 166.08, 162.21, 156.27, 155.76, 142.71, 131.93, 128.56, 128.04, 127.03, 126.93, 126.84, 120.42, 110.97, 95.00, 58.02, 55.55, 46.87, 13.64. HRMS (ESI$^+$), $C_{20}H_{22}N_3O_3^+$ [M+H]$^+$: calc. mass 352.1656, found 352.1647.

Preparation of **6t**.

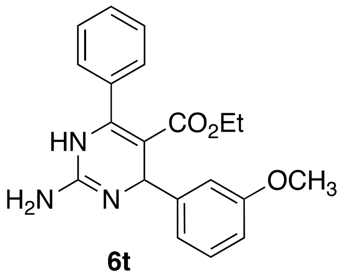

6t

6t was prepared according to the general aza-Biginelli reaction procedure on a 10.37 mmol scale. After precipitation on ice water according to the general procedure, the dried crude product was triturated with dichloromethane to afford the desired product as a pale-yellow solid (1.588 g, 48% yield). ^{1}H NMR (400 MHz, DMSO-D_6, room temperature) δ 7.52 (s, br, 1H), 7.25 (m, 7.30-7.20, 6H), 6.96 (d, J = 7.9 Hz, 1H), 6.94-6.91 (m, 1H), 6.82 (dd, J = 8.1, 2.5, 1H), 6.33 (s, br, 2H), 5.27 (s, 1H), 3.73 (s, 3H), 3.69 (q, J = 7.1 Hz, 2H), 0.75 (t, J = 7.1 Hz, 3H). ^{13}C NMR (101 MHz, room temperature, DMSO-D6): δ 166.26, 161.40, 159.28, 155.56, 147.80, 142.64, 129.46, 128.10, 127.04, 126.87, 118.50, 112.32, 112.01, 97.29, 58.12, 54.94, 52.66, 13.71. HRMS (ESI$^+$), $C_{20}H_{22}N_3O_3^+$ [M+H]$^+$: calc. mass 352.1656, found 352.1652.

Preparation of **6u**.

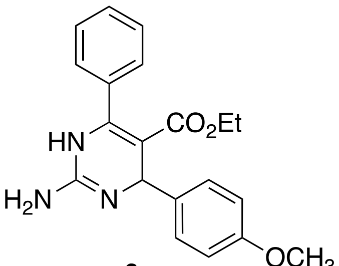

6u

6u was prepared according to the general aza-Biginelli reaction procedure on a 10.37 mmol scale. After precipitation on ice water according to the general procedure, the dried crude product was triturated with dichloromethane to afford the desired product as a pale-yellow solid (1.637 g, 49% yield). ^{1}H NMR (400 MHz, DMSO-D_6, room temperature) δ 7.43 (s, br, 1H), 7.30-7.20 (m, 7H), 6.90 (d, J = 8.7 Hz, 2H), 6.26 (s, br, 2H), 5.24 (s, 1H), 3.73 (s, 3H), 3.67 (q, J = 7.1 Hz, 2H), 0.74 (t, J = 7.1 Hz, 3H). ^{13}C NMR (101 MHz, room temperature, DMSO-D6): δ 166.25, 161.02, 158.42, 155.40, 142.74, 138.57, 128.10, 127.49, 126.96, 126.85, 113.68, 97.79, 58.06, 55.07, 52.28, 13.69. HRMS (ESI$^+$), $C_{20}H_{22}N_3O_3^+$ [M+H]$^+$: calc. mass 352.1656, found 352.1654.

Preparation of **6v**.

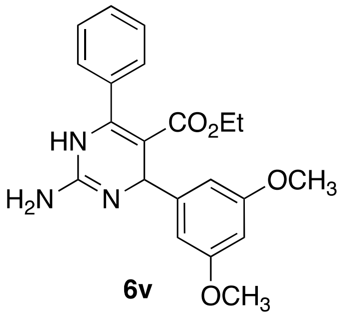

6v

6v was prepared according to the general aza-Biginelli reaction procedure on a 10.37 mmol scale. After precipitation on ice water according to the general procedure, the dried crude product was triturated with dichloromethane to afford the desired product as a pale-yellow solid (1.681 g, 47% yield). ^{1}H NMR (400 MHz, DMSO-D_6, room temperature) δ 7.54 (s, br, 1H), 7.31 – 7.20 (m, 5H), 6.54 (d, J = 2.3 Hz, 2H), 6.40 (t, J = 2.3 Hz, 1H), 6.34 (s, br, 1H), 5.24 (s, 1H), 3.75 – 3.66 (m, 8H), 0.76 (t, J = 7.1 Hz, 3H). ^{13}C NMR (101 MHz, room temperature, DMSO-D6): δ 166.78, 161.96, 160.99, 156.11, 148.99, 143.14, 128.59, 127.57, 127.41, 104.98, 98.82, 97.62, 58.65, 55.59, 53.15, 14.25. HRMS (ESI$^+$), $C_{21}H_{24}N_3O_4^+$ [M+H]$^+$: calc. mass 382.1761, found 382.1754.

Preparation of **6w**.

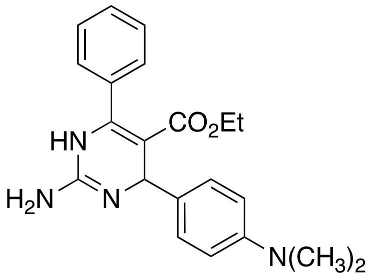

6w

6w was prepared according to the general aza-Biginelli reaction procedure on a 10.37 mmol scale. After precipitation on ice water according to the general procedure, the dried crude product was triturated with dichloromethane to afford the desired product as a pale-yellow solid (0.670 g, 19% yield). ^{1}H NMR (400 MHz, DMSO-D_6, room temperature) δ 7.33 (s, br, 1H), 7.28 – 7.20 (m, 5H), 7.17 (d, J = 8.7 Hz, 2H), 6.68 (d, J = 8.7 Hz, 2H), 6.19 (s, br, 2H), 5.17 (s, 1H), 3.66 (q, J = 7.1 Hz, 2H), 2.86 (s, 6H), 0.74 (t, J = 7.1 Hz, 3H). ^{13}C NMR (101 MHz, room temperature, DMSO-D6): δ 166.29, 160.64, 155.32, 149.77, 142.89, 134.33, 128.08, 127.02, 126.84, 126.79, 112.32, 98.09, 57.97, 52.35, 40.30, 13.70. HRMS (ESI$^+$), $C_{21}H_{25}N_4O_2^+$ [M+H]$^+$: calc. mass 365.1972, found 365.1975.

Preparation of **7a**.

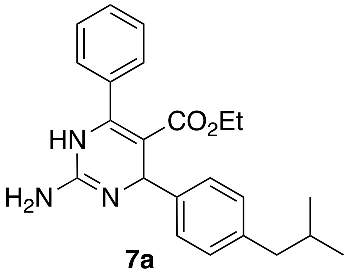

7a

7a was prepared according to the general aza-Biginelli reaction procedure on a 10.37 mmol scale. After precipitation on ice water according to the general procedure, the dried crude product was triturated with dichloromethane to afford the desired product as a pale-yellow solid (1.959 g, 55% yield). ^{1}H NMR (400 MHz, DMSO-D_6, room temperature) δ 7.32-7.20 (m, 7H); 7.12 (d, J = 7.8 Hz, 2H); 6.36 (s, br, 2H); 5.27 (s, 1H); 3.67 (q, J = 6.8 Hz, 2H); 2.42 (d, J = 7.1 Hz, 2H); 1.82 (sp, J = 6.7 Hz, 1H); 0.86 (d, J = 6.7 Hz, 6H); 0.73 (t, J = 6.7 Hz, 3H). ^{13}C NMR (101 MHz, room temperature, DMSO-D6): δ 166.24, 161.16, 155.47, 143.74, 142.71, 139.91, 128.87, 128.10, 126.96, 126.83, 126.14, 97.59, 58.03, 52.64, 44.34, 29.67, 22.21, 13.66. HRMS (ESI$^+$), $C_{23}H_{28}N_3O_2^+$ [M+H]$^+$: calc. mass 378.2176, found 378.2175.

Preparation of **7b**.

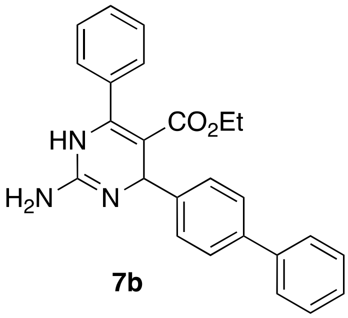

7b [23]

7b was prepared according to the general aza-Biginelli reaction procedure on a 9.35 mmol scale. After precipitation on ice water according to the general procedure, the dried crude product was triturated with dichloromethane to afford the desired product as a pale-yellow solid (1.256 g, 37% yield). ^{1}H NMR (400 MHz, DMSO-D_6, room temperature) δ 7.70 – 7.60 (m, 4H), 7.50 – 7.43 (m, 4H), 7.38-7.32 (m, 1H), 7.27 (s, 5H), 6.38 (s, br, 2H), 5.37 (s, 1H), 3.70 (q, J = 7.1 Hz, 2H), 0.76 (t, J = 7.1 Hz, 3H) (one broad N-H singlet buried underneath the aromatic signals is unreported). ^{13}C NMR (101 MHz, room temperature, DMSO-D6): δ 166.28, 161.38, 155.54, 145.41, 142.63, 140.08, 139.09, 128.92, 128.13, 127.33, 127.05, 126.93, 126.86, 126.75, 126.65, 97.41, 58.15, 52.59, 13.69. HRMS (ESI$^+$), $C_{25}H_{24}N_3O_2^+$ [M+H]$^+$: calc. mass 389.1863, found 389.1850.

Preparation of **7c**.

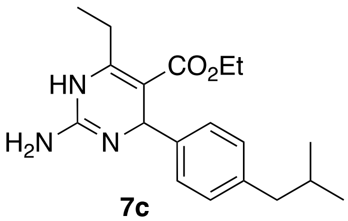

7c

7c was prepared according to the general aza-Biginelli reaction procedure on a 8.5 mmol scale. After precipitation on ice water according to the general procedure, the dried crude product isolated by column chromatography (gradient of 0-80% methanol in ethyl acetate) (0.311 g, 11% yield). ^{1}H NMR (400 MHz, DMSO-D_6, room temperature) δ 9.25 (s, br, 1H), 8.38 (s, br, 1H), 7.17-7.08 (m, 4H), 5.23 (s, 1H), 4.05-3.94 (m, 2H), 2.80-2.62 (m, 2H), 2.40 (d, J = 7.1 Hz, 2H), 1.81 (sp, J = 6.7 Hz, 1H), 1.12 (t, J = 7.3 Hz, 3H), 1.07 (t, J = 7.1 Hz, 3H), 0.84 (d, J = 6.7 Hz, 6H) (one broad N-H singlet buried underneath the aromatic signals is unreported). ^{13}C NMR (101 MHz, room temperature, DMSO-D6): δ 167.70, 164.80, 153.24, 141.22, 140.56, 129.15, 126.10, 99.93, 59.37, 51.63, 44.27, 29.59, 24.55, 22.23, 22.19, 13.99, 12.93. HRMS (ESI$^+$), $C_{19}H_{28}N_3O_2^+$ [M+H]$^+$: calc. mass 330.2176, found 330.2177.

Preparation of **7d**.

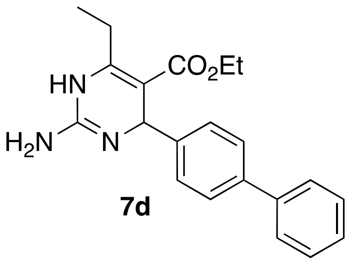

7d

7d was prepared according to the general aza-Biginelli reaction procedure on a 4.25 mmol scale. After precipitation on ice water according to the general procedure, the dried crude product was purified by column chromatography using a gradient of methanol in ethyl acetate, beginning at 100% ethyl acetate and finishing at 75% methanol/25% ethyl acetate (0.134 g, 9% yield). ^{1}H NMR (400 MHz, DMSO-D_6, room temperature) δ 7.65-7.61 (m, 2H), 7.59 (d, J = 8.3 Hz, 2H), 7.47-7.41 (m, 2H), 7.36-7.30 (m, 3H), 6.27 (s, br, 2H), 5.23 (s, 1H), 3.94 (q, J = 7.1 Hz, 2H), 2.64 (q, J = 7.5 Hz, 2H), 1.12-1.06 (m, 6H) (one broad N-H singlet buried underneath the aromatic signals is unreported). ^{13}C NMR (101 MHz, room temperature, DMSO-D6): δ 165.77, 155.51, 145.78, 140.06, 138.92, 128.90, 127.30, 126.88, 126.63, 96.38, 58.23, 52.38, 28.80, 14.32, 13.21 (two sets of coincident signals). HRMS (ESI$^+$), $C_{21}H_{24}N_3O_2^+$ [M+H]$^+$: calc. mass 350.1863, found 350.1861.

Preparation of **7e**.

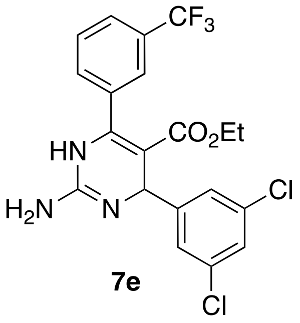

7e

7e was prepared according to the general aza-Biginelli reaction procedure on a 1.102 mmol scale. After precipitation on ice water according to the general procedure, the dried crude product was triturated with dichloromethane to afford the desired product as a pale-yellow solid (0.176 g, 46% yield). ^{1}H NMR (400 MHz, DMSO-D_6, room temperature) δ 7.68-7.62 (m, 1H), 7.56-7.49 (m, 4H), 7.35 (d, J = 2.0 Hz, 2H), 5.37 (s, 1H), 3.73 (qd, J = 7.1, 3.1 Hz, 2H), 0.75 (t, J = 7.1 Hz, 3H) (all NH signals were too broad and were not visible in this spectrum). ^{13}C NMR (101 MHz, room temperature, DMSO-D6): δ 165.64, 160.58, 155.69, 149.99, 143.10, 134.07, 132.37, 128.18, 127.96 (q, J = 31.6 Hz), 126.87, 125.05, 124.46 (q, J = 4.1 Hz), 124.39 (q, J = 271.8 Hz) 124.01 (q, J = 3.6 Hz), 96.61, 58.53, 52.01, 13.51. HRMS (ESI$^+$), $C_{20}H_{17}Cl_2F_3N_3O_2^+$ [M+H]$^+$: calc. mass 458.0644, found 458.0640.

Preparation of **7f**.

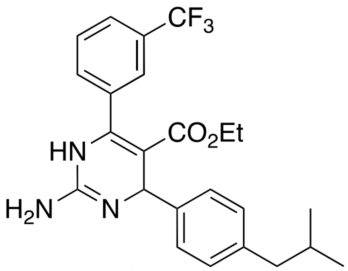

7f

7f was prepared according to the general aza-Biginelli reaction procedure on a 1.212 mmol scale. After precipitation on ice water according to the general procedure, the dried crude product was triturated with dichloromethane to afford the desired product as a pale-yellow solid (0.144 g, 30% yield). ^{1}H NMR (400 MHz, DMSO-D_6, room temperature) δ 7.66 – 7.61 (m, 1H), 7.60 – 7.47 (m, 4H), 7.28 (d, J = 8.1 Hz, 2H), 7.13 (d, J = 8.1 Hz, 2H), 6.38 (s, br, 2H), 5.29 (s, 1H), 3.68 (qd, J = 7.1, 2.2 Hz, 2H), 2.42 (d, J = 7.1 Hz, 2H), 1.81 (sp, J = 6.7 Hz, 1H), 0.86 (d, J = 6.6 Hz, 6H), 0.72 (t, J = 7.1 Hz, 3H). ^{13}C NMR (101 MHz, room temperature, DMSO-D6): δ 165.79, 159.60, 155.61, 143.68, 143.53, 140.05, 132.31, 128.94, 128.02, 127.67, 126.11, 124.46 (q, J = 4.1 Hz), 124.45 (q, J = 272.1 Hz), 123.63 (q, J = 4.3 Hz), 98.05, 58.21, 52.61, 44.31, 29.63, 22.19, 13.51. HRMS (ESI$^+$), $C_{24}H_{27}F_3N_3O_2^+$ [M+H]$^+$: calc. mass 446.2050, found 446.2048.

Preparation of **7g**.

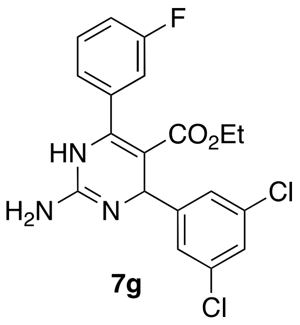

7g

7g was prepared according to the general aza-Biginelli reaction procedure on a 1.212 mmol scale. After precipitation on ice water according to the general procedure, the dried crude product was triturated with dichloromethane to afford the desired product as a pale-yellow solid (0.126 g, 28% yield). ^{1}H NMR (400 MHz, DMSO-D_6, room temperature) δ 7.68 (s, br, 1H), 7.52 (t, J = 1.9 Hz, 1H), 7.34 (d, J = 1.9 Hz, 2H), 7.15-6.95 (m, 4H), 6.56 (s, br, 2H), 5.35 (s, 1H), 3.74 (q, J = 7.1, 2H), 0.79 (t, J = 7.1 Hz, 3H). ^{13}C NMR (101 MHz, room temperature, DMSO-D6): δ 166.19, 161.87 (d, J = 241.5 Hz), 161.19, 156.03, 150.49, 145.02, 134.50, 129.34 (d, J = 8.9 Hz), 127.28, 125.51, 124.80 (d, J = 2.8 Hz), 115.21 (d, J = 21.5 Hz), 114.44 (d, J = 21.5 Hz), 96.85, 58.91, 52.45, 14.10. HRMS (ESI$^+$), $C_{19}H_{17}Cl_2FN_3O_2^+$ $[M+H]^+$: calc. mass 408.0676, found 408.0668.

Preparation of **7h**.

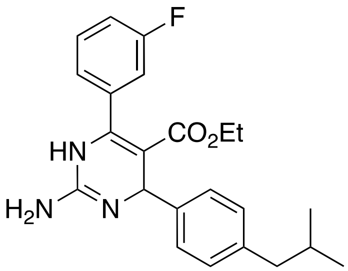

7h

7h was prepared according to the general aza-Biginelli reaction procedure on a 1.212 mmol scale. After precipitation on ice water according to the general procedure, the dried crude product was triturated with dichloromethane to afford the desired product as a pale-yellow solid (0.057 g, 13% yield). ^{1}H NMR (400 MHz, DMSO-D_6, room temperature) δ 7.52 (s, br, 1H), 7.33 – 7.28 (m, 1H), 7.26 (d, J = 8.0 Hz, 2H), 7.12 (d, J = 8.0 Hz, 2H), 7.10 – 7.03 (m, 2H), 6.99 (ddd, J = 10.2, 2.7, 1.5 Hz, 1H), 6.31 (s, br, 2H), 5.27 (s, 1H), 3.69 (qd, J = 7.1, 1.4 Hz, 2H), 2.42 (d, J = 7.1 Hz, 2H), 1.81 (sp, J = 6.7 Hz, 1H), 0.85 (d, J = 6.7 Hz, 6H), 0.76 (t, J = 7.1 Hz, 3H). ^{13}C NMR (101 MHz, room temperature, DMSO-D6): δ 165.90, 161.40 (d, J = 242.2 Hz), 159.75 (d, J = 2.4 Hz), 155.51, 145.21 (d, J = 8.0 Hz), 143.54, 140.01, 128.92, 128.75 (d, J = 8.3 Hz), 126.13, 124.29 (d, J = 2.9 Hz), 114.71 (d, J = 21.4 Hz), 113.55 (d, J = 20.9 Hz), 97.84, 58.15, 52.60, 44.33, 29.65, 22.23, 13.65. HRMS (ESI$^+$), $C_{23}H_{27}FN_3O_2^+$ [M+H]$^+$: calc. mass 396.2082, found 396.2073.

Preparation of **7i**.

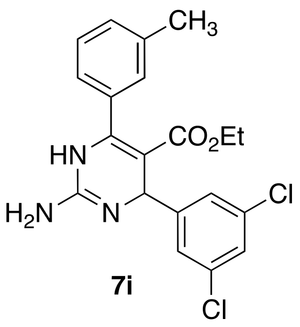

7i

7i was prepared according to the general aza-Biginelli reaction procedure on a 1.102 mmol scale. After precipitation on ice water according to the general procedure, the dried crude product was triturated with dichloromethane to afford the desired product as a pale-yellow solid (0.202 g, 45% yield). ^{1}H NMR (400 MHz, DMSO-D_6, room temperature) δ 7.47 (t, J = 1.9 Hz, 1H), 7.31 (d, J = 1.9 Hz, 2H), 7.19 (s, br, 1H), 7.15-7.10 (m, 1H), 7.08-7.03 (m, 1H), 7.02-6.96 (m, 2H), 6.49 (s, br, 2H), 5.29 (s, 1H), 3.69 (q, J = 7.1 Hz, 2H), 2.25 (s, 3H), 0.73 (t, J = 7.1 Hz, 3H). ^{13}C NMR (101 MHz, room temperature, DMSO-D6): δ 166.18, 161.75, 155.33, 150.29, 141.82, 135.79, 134.00, 128.74, 128.03, 126.86, 126.71, 125.45, 125.07, 96.13, 58.33, 52.16, 20.99, 13.67. HRMS (ESI^{+}), $C_{20}H_{20}Cl_2N_3O_2^{+}$ [M+H]$^{+}$: calc. mass 404.0927, found 404.0919.

Preparation of **7j**.

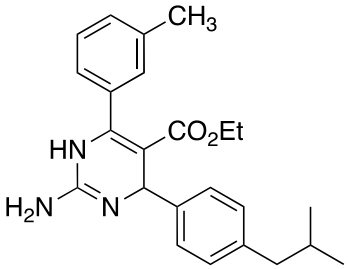

7j

7j was prepared according to the general aza-Biginelli reaction procedure on a 1.102 mmol scale. After precipitation on ice water according to the general procedure, the dried crude product was triturated with dichloromethane to afford the desired product as a pale-yellow solid (0.167 g, 39% yield). ^{1}H NMR (400 MHz, DMSO-D_6, room temperature) δ 7.71 (s, br, 1H), 7.27 (d, J = 8.0 Hz, 2H), 7.17-7.10 (m, 3H), 7.09-7.00 (m, 3H), 6.37 (s, br, 2H), 5.26 (s, 1H), 3.68 (q, J = 7.1 Hz, 2H), 2.42 (d, J = 7.1 Hz, 2H), 2.29 (s, 3H), 1.81 (sp, J = 6.8 Hz, 1H), 0.86 (d, J = 6.7 Hz), 0.74 (t, J = 7.1 Hz, 3H). ^{13}C NMR (101 MHz, room temperature, DMSO-D6): δ 166.26, 160.51, 155.24, 143.65, 142.23, 139.93, 135.64, 128.88, 128.75, 127.66, 126.76, 126.14, 125.39, 97.76, 58.06, 52.70, 44.34, 29.67, 22.24, 22.21, 21.00, 13.66. HRMS (ESI$^+$), $C_{24}H_{30}N_3O_2^+$ [M+H]$^+$: calc. mass 392.2333, found 392.2328.

PIDA Oxidation to Pyrimidines Reaction:

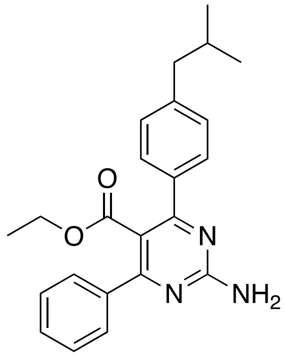

8a

Dihydropyrimidine **7a** (189 mg, 0.50 mmol, 1.00 eq) was stirred with phenyliodine(III) diacetate (193 mg, 0.60 mmol, 1.20 eq) in dichloromethane (4 mL) overnight at room temperature. After 13 hours, the reaction mixture was directly chromatographed using 0 to 100% ethyl acetate in hexanes to provide pyrimidine **8a** (71 mg, 0.20 mmol, 38%) as a yellow solid. ^{1}H NMR (500 MHz, $CDCl_3$) δ 7.62 – 7.56 (m, 2H), 7.52 (d, *J* = 8.1 Hz, 2H), 7.43 (dd, *J* = 5.3, 2.0 Hz, 3H), 7.21 (d, *J* = 8.0 Hz, 2H), 5.47 (s, 2H), 3.95 (q, *J* = 7.2 Hz, 2H), 2.51 (d, *J* = 7.2 Hz, 2H), 1.88 (dp, *J* = 13.5, 6.9 Hz, 1H), 0.91 (d, *J* = 6.6 Hz, 6H), 0.88 (t, *J* = 7.2 Hz, 3H). ^{13}C NMR (126 MHz, $CDCl_3$) δ 168.82, 166.86, 166.76, 162.26, 143.82, 138.29, 135.63, 129.79, 129.33, 128.53, 128.04, 127.89, 116.61, 61.48, 45.39, 30.34, 22.49, 13.56. HRMS (ESI^+), $C_{23}H_{26}N_3O_2^+$ $[M+H]^+$: calc. mass 376.2020, found 376.2010.

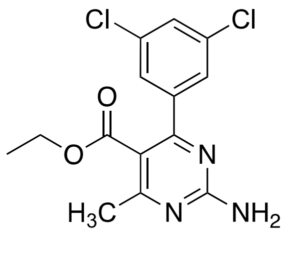

10

The dihydropyrimidine previously reported as **12b** by Boyer *et. al.*,[1] (57 mg, 0.17 mmol, 1.00 eq), was stirred with phenyliodine(III) diacetate (64 mg, 0.20 mmol, 1.20 eq) in dichloromethane (1 mL) overnight at room temperature. After 18 hours, the reaction mixture was directly chromatographed using 0 to 100% ethyl acetate in hexanes to provide pyrimidine **10** (18 mg, 0.06 mmol, 33%) as a yellow oil. ^{1}H NMR (400 MHz, $CDCl_3$) δ 7.43 (s, 3H), 5.32 (s, 2H), 4.20 – 4.08 (m, 2H), 2.50 (s, 3H), 1.41 – 0.91 (m, 3H). HRMS (ESI$^+$), $C_{14}H_{14}Cl_2N_3O_2^+$ [M+H]$^+$: calc. mass 326.0458, found 326.0456. Due to limited sample availability, a total-ion chromatogram is provided In lieu of ^{13}C NMR data.

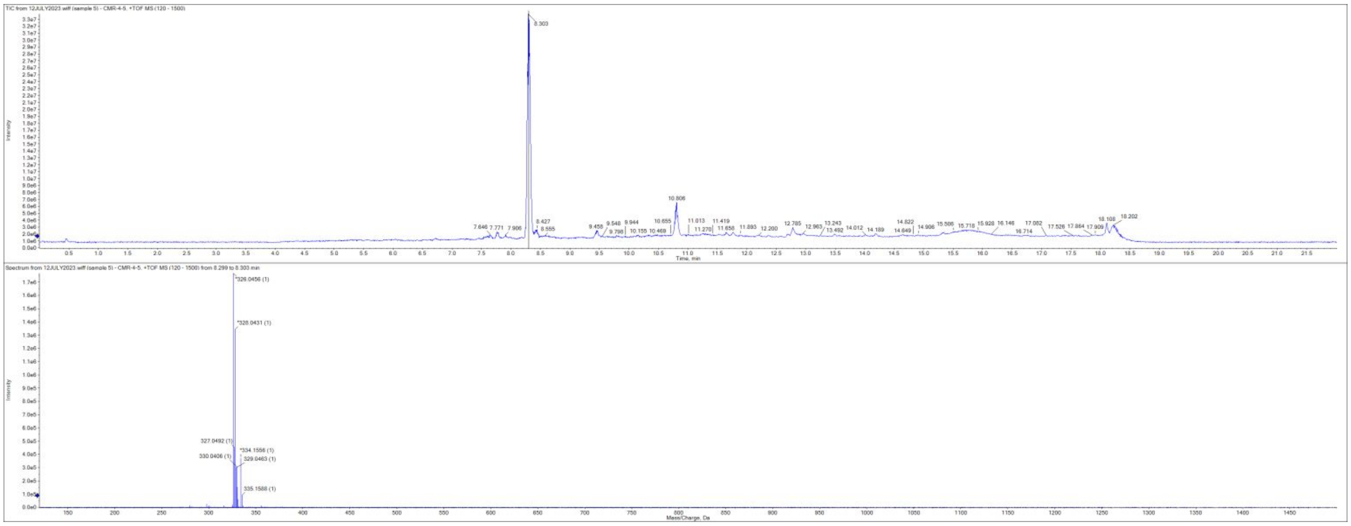

Photoaffinity Probe Synthesis:

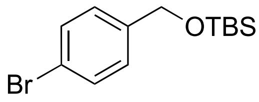

18

4-Bromobenzylalcohol (9.35 g, 50.0 mmol, 1.00 eq) was dissolved in dichloromethane (100 mL) and cooled in an ice bath. Imidazole (6.13 g, 90.0 mmol, 1.80 eq) and trimethylsilyl chloride (9.04 g, 60.0 mmol, 1.20 eq) were added and the reaction was stirred for 5 minutes before the ice bath was removed and the reaction was allowed to warm to room temperature. After 2 hours, the reaction was confirmed complete by TLC, concentrated to approximately half-volume under vacuum, diluted with diethyl ether (100 mL), washed with saturated ammonium chloride solution (1x 100 mL) and saturated sodium chloride solution (1x 100 mL) and concentrated under vacuum. The crude material was filtered through a plug of silica gel using 10% diethyl ether in hexanes and concentrated under vacuum to provide silyl ether **18** as a colorless oil (15.0 g, 49.7 mmol, 99%). ^{1}H NMR (500 MHz, $CDCl_3$) δ 7.45 (d, J = 8.4 Hz, 2H), 7.20 (d, J = 8.2 Hz, 2H), 4.68 (s, 2H), 0.94 (s, 9H), 0.10 (s, 6H). Spectral data is consistent with previously published data.[2]

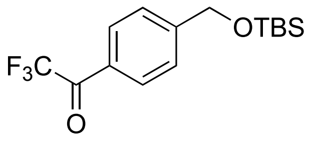

19

Bromide **18** (12.1 g, 40.0 mmol, 1.00 eq) was dissolved in THF (100 mL) and cooled in a dry-ice/ethanol bath (-78 °C) before *n*-butyl lithium (2.5 M in hexanes, 24 mL, 60.0 mmol, 1.50 eq) was added dropwise. The reaction was stirred while maintaining the dry-ice bath. After 1 hour, ethyl trifluoroacetate (8.57 mL, 72.0 mmol, 1.80 eq) was added. The dry-ice bath was replaced with a regular ice bath (0 °C) and stirring was continued. After 1 hour, the reaction was diluted with diethyl ether (100 mL), washed with saturated ammonium chloride solution (1x 100 mL) and saturated sodium chloride solution (1x 100 mL), dried with magnesium sulfate, filtered, and concentrated to provide a cloudy oil. The crude material was chromatographed using silica gel and 20% diethyl ether in hexanes to provide ketone **19** as a clear, pale-yellow oil (12.3 g, 34.7 mmol, 97%). ^{1}H NMR (500 MHz, $CDCl_3$) δ 8.05 (d, J = 7.7 Hz, 2H), 7.51 (d, J = 7.8 Hz, 2H), 4.83 (s, 2H), 0.96 (s, 9H), 0.13 (s, 6H). ^{19}F NMR (471 MHz, $CDCl_3$) δ -71.21. Spectral data is consistent with previously published data.[2]

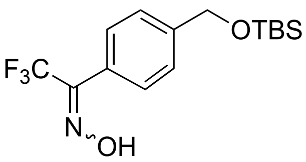

20

Ketone **19** (12.3 g, 38.6 mmol, 1.00 eq) was dissolved in a 1:1 mixture of pyridine (75 mL) and 2-propanol (75 mL). Hydroxyl amine hydrochloride (2.95 g, 42.5 mmol, 1.10 eq) was added and the reaction was stirred with heating in an oil bath set to 80 °C. After 2 hours, the reaction was cooled to room temperature, diluted with diethyl ether (150 mL), washed with 0.1 M HCl (3x 150 mL), saturated sodium bicarbonate solution (1x 150 mL), and saturated sodium chloride solution (1x 150 mL), dried with sodium sulfate, and concentrated under vacuum. The crude material was adsorbed onto celite and chromatographed using silica gel and 20% ethyl acetate in hexanes to provide an inseparable diastereomeric mixture of oxime **20** as a clear, pale-yellow oil (11.6 g, 34.7 mmol, 90%). ^{1}H NMR (500 MHz, $CDCl_3$) δ 8.65 (s, 1H), 8.45 (s, 1H), 7.51 – 7.36 (m, 8H), 4.79 (s, 2H), 4.78 (s, 2H), 0.96 (s, 9H), 0.95 (s, 9H), 0.12 (s, 6H), 0.11 (s, 6H). ^{19}F NMR (471 MHz, $CDCl_3$) δ -62.24, -66.51. Spectral data is consistent with previously published data.[2]

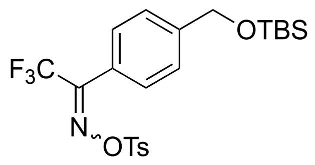

21

Oxime **20** (11.6 g, 34.7 mmol, 1.00 eq) was dissolved in dichloromethane (100 mL). Triethylamine (8.70 mL, 62.5 mmol, 1.80 eq), *p*-toluenesulfonyl chloride (7.28 g, 38.2 mmol, 1.10 eq), and 4-dimethylaminopyridine (170 mg, 1.39 mmol, 0.04 eq) were added sequentially and the reaction was stirred at room temperature. After 1 hour, the reaction was confirmed complete by TLC, diluted with diethyl ether (200 mL), washed with saturated ammonium chloride solution (2x 200 mL), saturated sodium bicarbonate solution (2x 200 mL), and saturated sodium chloride solution (2x 200 mL), dried with sodium sulfate, and concentrated under vacuum to provide an inseparable diastereomeric mixture of crude tosylate **21** (16.8 g), which was used without further purification. ^{1}H NMR (500 MHz, $CDCl_3$) δ 7.93 – 7.86 (m, 4H), 7.48 – 7.31 (m, 12H), 4.78 (s, 2H), 4.77 (s, 2H), 2.48 (s, 3H), 2.47 (s, 3H), 0.96 (s, 9H), 0.95 (s, 9H), 0.13 (s, 6H), 0.11 (s, 6H). ^{19}F NMR (471 MHz, CDCl3) δ -61.37, -66.48. Spectral data is consistent with previously published data.[2]

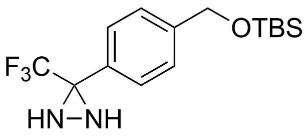

22

Crude tosylate **21** (16.9 g, approx. 34.7 mmol, 1.00 eq) was dissolved in THF (45 mL) and a solution of ammonia in methanol (7 M, 45 mL, 315 mmol, 9.00 eq) was added and the reaction was allowed to stir overnight. After 21 hours, the reaction was confirmed complete by ^{19}F NMR, diluted with diethyl ether (250 mL). The resulting white precipitate (ammonium tosylate) was removed by vacuum filtration, and the resulting liquid filtrate was concentrated under vacuum to provide crude diaziridine **22** (11.5 g), which was used without further purification. ^{1}H NMR (500 MHz, $CDCl_3$) δ 7.58 (d, *J* = 8.2 Hz, 2H), 7.38 (d, *J* = 8.7 Hz, 2H), 4.76 (s, 2H), 2.78 (d, *J* = 8.8 Hz, 1H), 2.20 (d, *J* = 8.8 Hz, 1H), 0.95 (s, 9H), 0.11 (s, 6H). ^{19}F NMR (471 MHz, $CDCl_3$) δ -75.50. Spectral data is consistent with previously published data.[2]

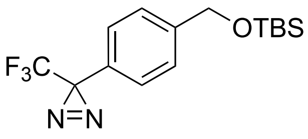

23

Crude diaziridine **22** (11.5 g, approx. 34.7 mmol, 1.00 eq) was combined with methanol (64 mL) and triethylamine (10.2 mL, 72.9 mmol, 2.10 eq). Molecular iodine (8.82 g, 34.7 mmol, 1.00 eq) was added, and the reaction was stirred at room temperature. After 1 hour, the reaction was diluted with diethyl ether (250 mL), washed with saturated sodium thiosulfate solution (2x 250 mL), saturated ammonium chloride solution (1x 250 mL), and saturated sodium chloride solution (1x 250 mL), dried with sodium sulfate, and concentrated under vacuum to provide crude diazirine **23** as a cloudy, brown oil (10.4 g), which was used without further purification. ^{1}H NMR (500 MHz, $CDCl_3$) δ 7.35 (d, J = 8.8 Hz, 2H), 7.16 (d, J = 8.1 Hz, 2H), 4.74 (s, 2H), 0.94 (s, 9H), 0.10 (s, 6H). ^{19}F NMR (471 MHz, $CDCl_3$) δ -65.18. Spectral data is consistent with previously published data.[2]

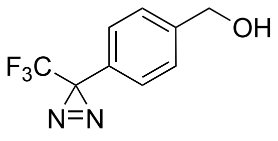

24

Crude silyl ether **23** (10.4 g, approx. 31.4 mmol, 1.00 eq) was dissolved in methanol (100 mL) and cooled in an ice bath. Concentrated hydrochloric acid (1 mL) was added and the reaction was stirred with the ice bath maintained. After 45 minutes, the reaction was confirmed complete by TLC, and concentrated under vacuum without any additional work-up. The crude material was adsorbed onto celite and chromatographed using silica gel and 0 to 30% ethyl acetate in hexanes to provide alcohol **24** as a light brown oil (5.75 g, 26.6 mmol, 77% over 4 steps from oxime **20**, 94% average). ^{1}H NMR (400 MHz, $CDCl_3$) δ 7.39 (d, *J* = 8.6 Hz, 2H), 7.19 (d, *J* = 7.9 Hz, 2H), 4.72 (s, 2H). ^{19}F NMR (376 MHz, $CDCl_3$) δ -65.17 (d, *J* = 1.1 Hz). Spectral data is consistent with previously published data.[2]

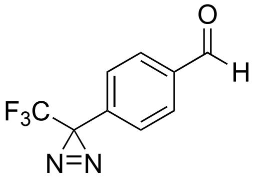

25

Alcohol **24** (5.75 g, 26.6 mmol, 1.00 eq) was dissolved in dichloromethane (90 mL) and cooled in an ice bath. Solid Dess-Martin Periodinane (11.3 g, 26.6 mmol, 1.00 eq) was added and the reaction was stirred. After 5 minutes, the ice bath was removed, and the reaction was allowed to warm to room temperature. After 1 hour, the reaction was confirmed complete by TLC, diluted with diethyl ether (180 mL), washed with a 1:1 mixture of saturated sodium bicarbonate and 10% sodium thiosulfate solution (1x 100 mL), water (1x 100 mL), and saturated sodium chloride solution (1x 100 mL), dried with sodium sulfate and concentrated under vacuum. The crude material was adsorbed onto celite and chromatographed using silica gel and 0 to 20% ethyl acetate in hexanes to provide aldehyde **25** as a pale-yellow oil (4.66 g, 21.8 mmol, 82%). ^{1}H NMR (500 MHz, $CDCl_3$) δ 10.04 (s, 1H), 7.91 (d, J = 8.5 Hz, 2H), 7.35 (d, J = 8.1 Hz, 2H). ^{19}F NMR (471 MHz, $CDCl_3$) δ -64.66. ^{13}C NMR (126 MHz, $CDCl_3$) δ 191.18, 136.89, 135.32, 129.93, 127.05, 121.90 (q, J = 274.8 Hz), 28.55 (q, J = 40.8 Hz). Spectral data is consistent with previously published data.[3]

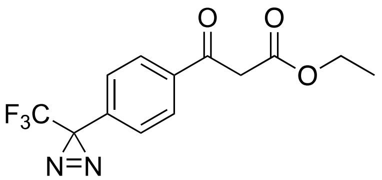

11

Aldehyde **25** (2.14 g, 10.0 mmol, 1.00 eq) and ethyl diazoacetate (commercially provided as approximately 80 wt% in dichloromethane, 3.42 g, 24.0 mmol, 2.40 eq) were dissolved in additional dichloromethane (50 mL). Solid anhydrous tin(II) chloride (1.90 g, 10.0 mmol, 1.00 eq) was added and the reaction was stirred at room temperature. After 19 hours, the reaction was vacuum filtered to remove some solids and the resulting filtrate, whether clear or turbid, was adsorbed onto celite and chromatographed using silica gel and 0 to 10% ethyl acetate in hexanes to provide keto ester **11** (2.06 g, 6.84 mmol, 68%). Keto-tautomer: ^{1}H NMR (500 MHz, $CDCl_3$) δ 7.97 (d, J = 8.5 Hz, 2H), 7.29 (d, J = 8.3 Hz, 2H), 4.21 (q, J = 7.2 Hz, 2H), 3.97 (s, 2H), 1.25 (t, J = 7.1 Hz, 3H). ^{19}F NMR (471 MHz, $CDCl_3$) δ -64.68. Enol-tautomer: ^{1}H NMR (500 MHz, $CDCl_3$) δ 7.79 (d, J = 8.5 Hz, 2H), 7.22 (d, J = 8.3 Hz, 2H), 5.67 (s, 1H), 4.27 (q, J = 7.1 Hz, 2H), 1.34 (t, J = 7.1 Hz, 3H). ^{19}F NMR (471 MHz, $CDCl_3$) δ -64.84. Mixture of tautomers: ^{13}C NMR (126 MHz, $CDCl_3$) δ 191.63, 173.03, 169.72, 167.19, 136.67, 134.73, 134.58, 131.98, 128.90, 126.76, 126.55, 126.43, 122.03 (q, J = 274.7 Hz), 121.89 (q, J = 274.7 Hz), 88.55, 61.72, 60.65, 46.05, 28.48 (q, J = 40.7 Hz), 28.46 (q, J = 40.4 Hz), 14.27, 14.08. HRMS (ESI$^+$), $C_{13}H_{12}F_3N_2O_3^+$ $[M+H]^+$: calc. mass 301.0795, found 301.0790. Spectral data is consistent with previously published data.[4]

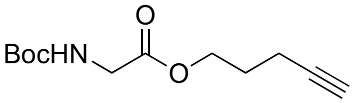

S3

Boc-glycine (3.50 g, 20.0 mmol, 1.00 eq), DIPEA (2.59 g, 20.0 mmol, 1.00 eq), and 4-pentyn-1-ol (1.68 g, 20.0 mmol, 1.00 eq) were combined with DMF (40 mL) in an ice bath. Solid HATU (7.61 g, 20.0 mmol, 1.00 eq) was added to the reaction, which was stirred and allowed to warm to room temperature overnight. After 19 hours, the reaction was diluted with ethyl acetate (360 mL), washed with 0.2 M HCl (3x 360 mL), saturated sodium bicarbonate solution (2x 360 mL), and saturated sodium chloride solution (1x 360 mL), then dried with sodium sulfate, and concentrated under vacuum to provide crude ester **S3** (4.44 g) as a beige solid, which was used without further purification. ^{1}H NMR (400 MHz, $CDCl_3$) δ 5.00 (s, 1H), 4.26 (t, J = 6.3 Hz, 2H), 3.91 (d, J = 5.6 Hz, 2H), 2.29 (td, J = 7.0, 2.7 Hz, 2H), 1.97 (t, J = 2.7 Hz, 1H), 1.87 (p, J = 6.6 Hz, 2H), 1.45 (s, 9H). ^{13}C NMR (101 MHz, $CDCl_3$) δ 170.43, 155.80, 82.88, 80.05, 69.28, 63.87, 42.47, 28.37, 27.47, 15.21. HRMS (ESI^{+}), $C_{12}H_{20}NO_4^{+}$ $[M+H]^{+}$: calc. mass 242.1387, found 242.1388.

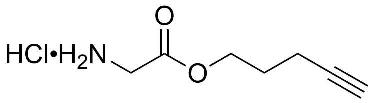

S4

Crude carbamate **S3** was cooled in a flask in an ice bath before a solution of HCl in dioxane (4 M, 55.2 mL, 220.8 mmol, 12.0 eq) was added. The ice bath was removed, and the reaction was stirred while warming to room temperature. After 3.5 hours, the reaction was confirmed complete by TLC and diluted with diethyl ether (approx. 100 mL). The resulting precipitate was homogenized by sonication for approx. 5 minutes, then vacuum filtered and washed with additional ether. The solid was collected in a flask and dried under vacuum to provide crude hydrochloride salt **S4** (2.96 g) as a beige solid, which was used without further purification. ^{1}H NMR (500 MHz, Methanol-D_4) δ 4.35 (t, J = 6.3 Hz, 2H), 3.86 (s, 2H), 3.35 (s, 2H), 2.37 – 2.25 (m, 3H), 1.90 (p, J = 6.6 Hz, 2H). ^{13}C NMR (126 MHz, Methanol-D_4) δ 168.41, 83.63, 70.42, 65.88, 41.03, 28.46, 15.62. HRMS (ESI$^+$), $C_7H_{12}NO_2^+$ [M+H]$^+$: calc. mass 142.0863, found 142.0860.

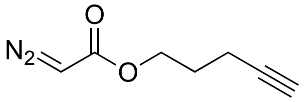

S5

A slurry of amine salt **S4** (2.96 g, 16.7 mmol, 1.00 eq) in dichloromethane (28 mL) was cooled in an ice bath. Sodium nitrite (1.73 g, 25.0 mmol, 1.50 eq) was dissolved in water (28 mL), and this was added to the reaction, which was stirred vigorously while maintaining the ice bath. After 2 hours, the reaction was diluted with saturated sodium bicarbonate solution (approx. 50 mL). The whole mixture was then transferred to a separatory funnel. The organic layer was collected, and the aqueous layer was washed with additional dichloromethane (20 mL). The combined organic extracts were dried with sodium sulfate and concentrated under vacuum to provide diazo ester **S5** (1.60 g, 10.5 mmol, 53% over 3 steps from Boc-glycine, 80% average) as a yellow oil. ^{1}H NMR (400 MHz, $CDCl_3$) δ 4.74 (s, 1H), 4.27 (t, J = 6.3 Hz, 2H), 2.29 (td, J = 7.0, 2.7 Hz, 2H), 1.97 (t, J = 2.7 Hz, 1H), 1.92 – 1.81 (m, 2H). ^{13}C NMR (101 MHz, $CDCl_3$) δ 166.77, 82.96, 69.14, 63.37, 46.22, 27.74, 15.16. HRMS (ESI$^+$), $C_7H_9N_2O_2^+$ [M+H]$^+$: calc. mass 153.0659, found 153.0653.

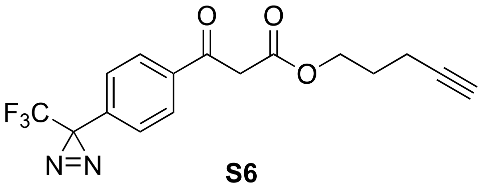

S6

Aldehyde **25** (938 mg, 4.38 mmol, 1.00 eq) and diazo ester **S5** (1.60 g, 10.5 mmol, 2.40 eq) were dissolved in dichloromethane (22 mL). Solid anhydrous tin(II) chloride (1.90 g, 10.0 mmol, 1.00 eq) was added and the reaction was stirred at room temperature. After 24 hours, the reaction was directly adsorbed onto celite and chromatographed using silica gel and 0 to 10% ethyl acetate in hexanes to provide keto ester **S6** (1.20 g, 3.54 mmol, 81%). Mixture of tautomers: ^{1}H NMR (400 MHz, $CDCl_3$) δ 12.49 (d, *J* = 0.9 Hz, 1H), 7.96 (d, *J* = 8.3 Hz, 2H), 7.83 – 7.77 (m, 2H), 7.32 – 7.27 (m, 2H), 7.23 (d, *J* = 8.2 Hz, 2H), 5.68 (d, *J* = 0.9 Hz, 1H), 4.37 – 4.23 (m, 4H), 3.99 (d, *J* = 0.9 Hz, 2H), 2.37 – 2.28 (m, 2H), 2.22 (td, *J* = 7.0, 2.6 Hz, 2H), 1.99 (qd, *J* = 2.7, 0.8 Hz, 2H), 1.96 – 1.80 (m, 4H). ^{19}F NMR (376 MHz, CDCl3) δ -64.71, -64.85. ^{13}C NMR (101 MHz, $CDCl_3$) δ 191.50, 172.94, 169.96, 167.35, 167.10, 136.62, 134.66, 132.07, 128.88, 126.84, 126.61, 126.48, 122.03 (q, *J* = 120.7 Hz), 121.88 (q, *J* = 120.5 Hz), 88.37, 82.94, 82.79, 82.71, 69.39, 69.28, 69.26, 64.75, 64.14, 63.09, 46.01, 40.92, 28.49 (q, *J* = 40.6 Hz), 27.61, 27.37, 15.24, 15.15, 15.07. HRMS (ESI$^+$), $C_{16}H_{14}F_3N_2O_3^+$ $[M+H]^+$: calc. mass 339.0951, found 339.0936. Note: Some additional ^{1}H and ^{13}C NMR signals were observed, potentially due to additional isomerization or impurities; however, this was inconsequential to the success of future reactions.

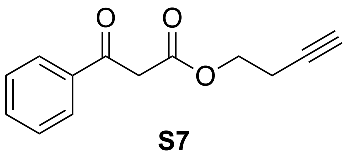

S7

Ethyl benzoylacetate (577 mg, 3.00 mmol, 1.00 eq) and 3-butyn-1-ol (2.10 g, 30.0 mmol, 10.0 eq) were combined with toluene (25 mL) in a flask affixed with an empty/dry reflux condenser. The mixture was stirred with heating in an oil bath set to 125 °C. After 2 days, the reaction was cooled to room temperature and concentrated under vacuum. The crude mixture was chromatographed using silica gel and 0 to 30% ethyl acetate hexanes to provide butynyl ester **S7** (630 mg, 2.91 mmol, 97%). ^{1}H NMR (400 MHz, $CDCl_3$) δ 12.43 (s, 1H), 7.94 (dd, J = 8.5, 1.3 Hz, 3H), 7.80 – 7.76 (m, 1H), 7.64 – 7.56 (m, 2H), 7.52 – 7.38 (m, 4H), 5.71 (s, 1H), 4.32 (t, J = 6.8 Hz, 2H), 4.27 (t, J = 6.9 Hz, 2H), 4.03 (s, 2H), 2.61 (td, J = 6.8, 2.6 Hz, 2H), 2.53 (td, J = 6.9, 2.7 Hz, 2H), 2.04 (t, J = 2.7 Hz, 1H), 1.97 (t, J = 2.7 Hz, 1H). ^{13}C NMR (101 MHz, $CDCl_3$) δ 192.15, 172.63, 171.67, 167.13, 135.73, 133.66, 133.01, 131.31, 128.65, 128.44, 128.34, 125.95, 86.89, 79.96, 79.66, 70.10, 62.77, 61.89, 45.54, 18.87, 18.64. HRMS (ESI$^+$), $C_{13}H_{13}O_3^+$ $[M+H]^+$: calc. mass 217.0859, found 217.0858.

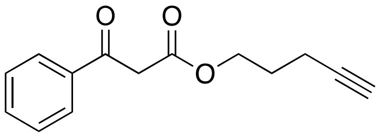

S8

Ethyl benzoylacetate (577 mg, 3.00 mmol, 1.00 eq) and 4-pentyn-1-ol (2.52 g, 30.0 mmol, 10.0 eq) were combined with toluene (25 mL) in a flask affixed with an empty/dry reflux condenser. The mixture was stirred with heating in an oil bath set to 125 °C. After 2 days, the reaction was cooled to room temperature and concentrated under vacuum. The crude mixture was chromatographed using silica gel and 0 to 30% ethyl acetate hexanes to provide pentynyl ester **S8** (633 mg, 2.75 mmol, 92%). ^{1}H NMR (400 MHz, $CDCl_3$) δ 12.52 (s, 1H), 7.97 – 7.89 (m, 3H), 7.81 – 7.73 (m, 1H), 7.65 – 7.56 (m, 2H), 7.54 – 7.36 (m, 4H), 5.68 (s, 1H), 4.32 (t, J = 6.3 Hz, 2H), 4.27 (t, J = 6.2 Hz, 2H), 4.01 (s, 2H), 2.34 (td, J = 7.0, 2.7 Hz, 2H), 2.23 (td, J = 7.0, 2.7 Hz, 2H), 1.99 (t, J = 2.7 Hz, 1H), 1.95 (t, J = 2.6 Hz, 1H), 1.91 – 1.76 (m, 4H). ^{13}C NMR (101 MHz, $CDCl_3$) δ 192.45, 173.11, 171.63, 167.47, 135.95, 133.81, 133.33, 131.35, 128.82, 128.57, 128.47, 126.07, 87.18, 82.98, 82.90, 69.21, 69.16, 63.87, 62.78, 45.90, 27.58, 27.35, 15.17, 15.01. HRMS (ESI^{+}), $C_{14}H_{15}O_3^{+}$ $[M+H]^{+}$: calc. mass 231.1016, found 231.1012.

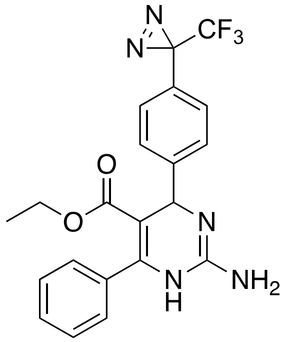

26a

Aldehyde **25** (215 mg, 1.00 mmol, 1.00 eq), ethyl benzoyl acetate (192 mg, 1.10 mmol, 1.10 eq), guanidine carbonate (108 mg, 1.20 mmol, 1.20 eq), and sodium bicarbonate (336 mg, 4.00 mmol, 4.00 eq) were combined in DMF (2.0 mL) and stirred with heating in an oil bath set to 70 °C. After 85 minutes, the reaction was cooled to room temperature and poured onto crushed ice. After the ice had melted, the resulting yellow precipitate was collected by vacuum filtration, washed with additional water, and dried under vacuum. This crude material was suspended in a minimal amount of dichloromethane, vacuum filtered, washed with additional dichloromethane, and dried under vacuum to provide dihydropyrimidine **26a** (186 mg, 0.43 mmol 43%) as a yellow solid. ^{1}H NMR (400 MHz, DMSO-D_6) δ 7.50 (d, J = 8.4 Hz, 2H), 7.37 – 6.97 (m, 7H), 6.39 (s, br, 2H), 5.33 (s, 1H), 3.67 (q, J = 7.1 Hz, 2H), 0.72 (t, J = 7.1 Hz, 3H) (one broad N-H singlet buried underneath the aromatic signals is unreported). ^{19}F NMR (376 MHz, DMSO-D_6) δ -64.52. ^{13}C NMR (126 MHz, DMSO-D_6) δ 166.12, 161.84, 155.52, 148.50, 142.45, 128.14, 127.32, 127.14, 126.85, 126.67, 126.29, 121.98 (q, J = 274.8 Hz), 96.77, 58.17, 52.41, 28.08 (q, J = 39.9 Hz), 13.62. HRMS (ESI$^+$), $C_{21}H_{19}F_3N_5O_2^+$ $[M+H]^+$: calc. mass 430.1485, found 430.1466.

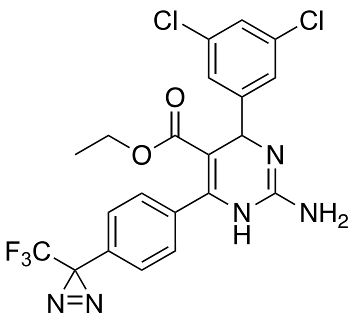

26b

3,5-Dichlorobenzaldehyde (100 mg, 0.57 mmol, 1.00 eq), keto ester **11** (188 mg, 0.63 mmol, 1.10 eq), guanidine carbonate (61 mg, 0.68 mmol, 1.20 eq), and sodium bicarbonate (192 mg, 2.28 mmol, 4.00 eq) were combined in DMF (1.1 mL) and stirred with heating in an oil bath set to 70 °C. After 60 minutes, the reaction was cooled to room temperature, diluted with ethyl acetate (20 mL), washed with water (2x 20 mL), saturated sodium chloride solution (1x 20 mL), dried with sodium sulfate, and concentrated under vacuum. The crude material was chromatographed once using 50 to 100% ethyl acetate in hexanes and chromatographed a second time using 80 to 100% ethyl acetate in hexanes to provide dihydropyrimidine **26b** (93 mg, 0.19 mmol, 33%) as a yellow solid. ^{1}H NMR (500 MHz, DMSO-D_6) δ 7.51 (t, J = 1.9 Hz, 1H), 7.41 – 7.31 (m, 4H), 7.22 (d, J = 8.0 Hz, 2H), 6.70 (s, br, 2H), 5.38 (s, 1H), 3.72 (qd, J = 7.1, 2.8 Hz, 2H), 0.73 (t, J = 7.1 Hz, 3H) (one broad N-H singlet buried underneath the aromatic signals is unreported). ^{19}F NMR (471 MHz, DMSO-D_6) δ -64.54. ^{13}C NMR (126 MHz, DMSO-D_6) δ 165.49, 159.56, 155.19, 149.70, 143.51, 134.10, 129.09, 126.94, 126.63, 125.33, 125.11, 121.98 (q, J = 274.7 Hz), 97.13, 58.61, 52.02, 28.15 (q, J = 39.9 Hz), 13.36. HRMS (ESI$^+$), $C_{21}H_{17}Cl_2F_3N_5O_2^+$ [M+H]$^+$: calc. mass 498.0706, found 498.0697.

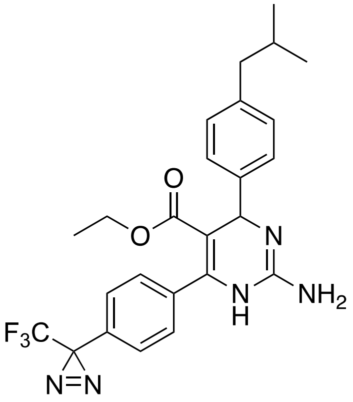

26c

4-Isobutylbenzaldehyde (162 mg, 1.00 mmol, 1.00 eq), keto ester **11** (223 mg, 1.10 mmol, 1.10 eq), guanidine carbonate (108 mg, 1.20 mmol, 1.20 eq), and sodium bicarbonate (336 mg, 4.00 mmol, 4.00 eq) were combined in DMF (2.0 mL) and stirred with heating in an oil bath set to 70 °C. After 5 hours (reaction may be done in less time), the reaction was cooled to room temperature, diluted with ethyl acetate (40 mL), washed with water (1x 40 mL), saturated sodium chloride solution (1x 40 mL), dried with sodium sulfate, and concentrated under vacuum. The crude material was chromatographed once using 0 to 100% ethyl acetate in hexanes then 0 to 100% methanol in ethyl acetate and chromatographed a second time using 0 to 100% methanol in ethyl acetate to provide dihydropyrimidine **26c** (144 mg, 0.30 mmol, 30%) as a yellow solid. ^{1}H NMR (500 MHz, DMSO-D_6) δ 7.36 (dd, *J* = 8.5, 1.8 Hz, 2H), 7.26 (dd, *J* = 8.1, 1.8 Hz, 2H), 7.20 (d, *J* = 7.9 Hz, 2H), 7.12 (d, *J* = 7.5 Hz, 2H), 6.40 (s, br, 2H), 5.28 (d, *J* = 1.8 Hz, 1H), 3.66 (qt, *J* = 7.2, 1.9 Hz, 2H), 2.42 (dd, *J* = 7.1, 1.7 Hz, 2H), 1.87 – 1.71 (m, 1H), 0.85 (d, *J* = 6.6 Hz, 6H), 0.75 – 0.62 (m, 3H) (one broad N-H singlet buried underneath the aromatic signals is unreported). ^{19}F NMR (471 MHz, DMSO-D_6) δ -64.57. ^{13}C NMR (126 MHz, DMSO-D_6) δ 165.68, 155.20, 144.24, 143.29, 140.14, 129.05, 128.96, 126.21, 126.15, 125.22 (d, *J* = 1.3 Hz), 122.00 (q, *J* = 274.7 Hz), 98.46, 58.30, 52.59, 44.32, 29.65, 28.16 (q, *J* = 39.9 Hz), 22.20, 13.39. HRMS (ESI$^+$), $C_{25}H_{27}F_3N_5O_2^+$ [M+H]$^+$: calc. mass 486.2111, found 486.2105.

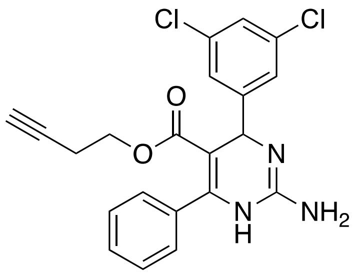

26d

3,5-Dichlorobenzaldehyde (175 mg, 1.00 mmol, 1.00 eq), keto ester **S7** (238 mg, 1.10 mmol, 1.10 eq), guanidine carbonate (108 mg, 1.20 mmol, 1.20 eq), and sodium bicarbonate (336 mg, 4.00 mmol, 4.00 eq) were combined in DMF (2.0 mL) and stirred with heating in an oil bath set to 70 °C. After 6 hours (reaction may be done in less time), the reaction was cooled to room temperature and poured onto crushed ice. After the ice had melted, the resulting yellow precipitate was collected by vacuum filtration, washed with additional water, and dried under vacuum. The crude material was chromatographed once using 0 to 60% methanol in ethyl acetate and chromatographed a second time using 0 to 50% methanol in ethyl acetate to provide dihydropyrimidine **26d** (34 mg, 0.082 mmol, 8%) as a yellow solid. ^{1}H NMR (500 MHz, DMSO-D_6) δ 7.52 (t, J = 1.9 Hz, 1H), 7.38 (d, J = 1.9 Hz, 2H), 7.33 – 7.22 (m, 5H), 6.76 (s, br, 2H), 5.37 (s, 1H), 3.79 (td, J = 6.8, 2.4 Hz, 2H), 2.73 (t, J = 2.6 Hz, 1H), 2.15 – 1.90 (m, 2H) (one broad N-H singlet buried underneath the aromatic signals is unreported). ^{13}C NMR (126 MHz, DMSO-D_6) δ 165.35, 155.10, 149.71, 134.09, 128.49, 128.30, 127.82, 127.64, 127.13, 126.91, 125.09, 80.87, 72.17, 60.77, 51.92, 40.43, 18.08. HRMS (ESI$^+$), $C_{21}H_{18}Cl_2N_3O_2^+$ $[M+H]^+$: calc. mass 414.0771, found 414.0758.

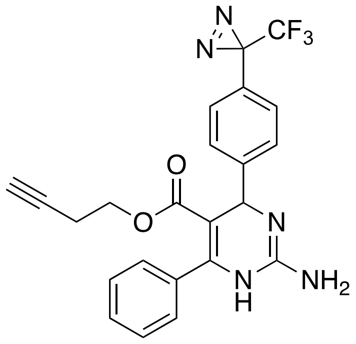

26e

Aldehyde **25** (354 mg, 1.65 mmol, 1.00 eq), keto ester **S7** (394 mg, 1.82 mmol, 1.10 eq), guanidine carbonate (178 mg, 1.98 mmol, 1.20 eq), and sodium bicarbonate (554 mg, 6.60 mmol, 4.00 eq) were combined in DMF (3.3 mL) and stirred with heating in an oil bath set to 70 °C. After 60 minutes, the reaction was cooled to room temperature and poured onto crushed ice. After the ice had melted, the resulting yellow precipitate was collected by vacuum filtration, washed with additional water, and dried under vacuum. The crude material was chromatographed once using 0 to 100% ethyl acetate in hexanes then 0 to 100% methanol in ethyl acetate, and chromatographed a second time using C18 silica gel and 0 to 100% acetonitrile in water (with 0.1% trifluoroacetic acid) to provide the trifluoroacetate salt of dihydropyrimidine **26e** (75 mg, 0.08 mmol, 5%) as a yellow solid. ^{1}H NMR (500 MHz, DMSO-D_6) δ 10.77 (s, 1H), 9.59 (s, 1H), 7.58 (d, *J* = 8.4 Hz, 2H), 7.55 – 7.44 (m, 5H), 7.38 (d, *J* = 8.1 Hz, 2H), 5.61 (s, 1H), 3.85 (qt, *J* = 10.7, 6.6 Hz, 2H), 2.78 (t, *J* = 2.6 Hz, 1H), 2.09 (tt, *J* = 6.6, 3.1 Hz, 2H) (one broad N-H singlet buried underneath the aromatic signals is unreported). ^{19}F NMR (471 MHz, DMSO-D_6) δ -64.45, -73.66. ^{13}C NMR (126 MHz, DMSO-D_6) δ 163.61, 151.17, 145.69, 143.88, 133.07, 130.09, 128.73, 128.19, 127.68, 127.67, 127.24, 127.23, 121.90 (q, *J* = 274.8 Hz), 102.80, 80.50, 72.45, 62.08, 51.88, 28.04 (q, *J* = 40.1 Hz), 17.83. HRMS (ESI$^+$), $C_{23}H_{19}F_3N_5O_2^+$ [M+H]$^+$: calc. mass 454.1485, found 454.1465.

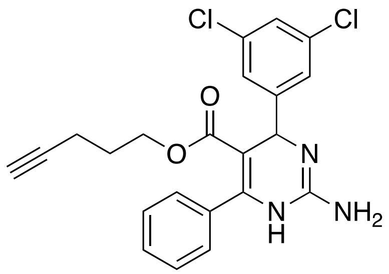

26f

3,5-Dichlorobenzaldehyde (175 mg, 1.00 mmol, 1.00 eq), keto ester **S8** (253 mg, 1.10 mmol, 1.10 eq), guanidine carbonate (108 mg, 1.20 mmol, 1.20 eq), and sodium bicarbonate (336 mg, 4.00 mmol, 4.00 eq) were combined in DMF (2.0 mL) and stirred with heating in an oil bath set to 70 °C. After 60 minutes, the reaction was cooled to room temperature and poured onto crushed ice. After the ice had melted, the resulting yellow precipitate was collected by vacuum filtration, washed with additional water, and dried under vacuum. The crude material was chromatographed using 0 to 40% methanol in ethyl acetate to provide dihydropyrimidine **26f** (120 mg, 0.28 mmol, 28%) as a yellow solid. ^{1}H NMR (400 MHz, DMSO-D_6) δ 7.53 (t, J = 1.9 Hz, 1H), 7.36 (d, J = 1.9 Hz, 2H), 7.32 – 7.19 (m, 5H), 6.60 (s, br, 2H), 5.36 (s, 1H), 3.75 (td, J = 6.3, 2.6 Hz, 2H), 2.69 (t, J = 2.7 Hz, 1H), 1.73 – 1.62 (m, 2H), 1.30 (qd, J = 7.0, 5.5 Hz, 2H) (one broad N-H singlet buried underneath the aromatic signals is unreported). ^{13}C NMR (126 MHz, DMSO-D_6) δ 165.93, 161.02, 155.09, 149.87, 141.48, 134.16, 128.12, 127.72, 127.22, 126.91, 125.10, 96.30, 83.58, 71.28, 61.54, 52.07, 27.07, 14.36. HRMS (ESI$^+$), $C_{22}H_{20}Cl_2N_3O_2^+$ [M+H]$^+$: calc. mass 428.0927, found 428.0915.

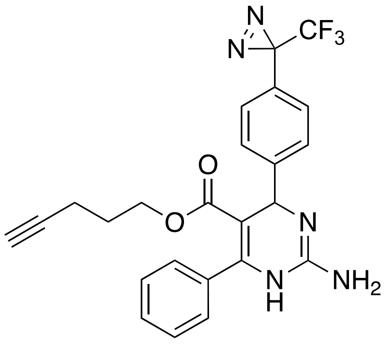

26g

Aldehyde **25** (214 mg, 1.00 mmol, 1.00 eq), keto ester **S8** (253 mg, 1.10 mmol, 1.10 eq), guanidine carbonate (108 mg, 1.20 mmol, 1.20 eq), and sodium bicarbonate (336 mg, 4.00 mmol, 4.00 eq) were combined in DMF (2.0 mL) and stirred with heating in an oil bath set to 70 °C. After 80 minutes, the reaction was cooled to room temperature and poured onto crushed ice. After the ice had melted, the resulting yellow precipitate was collected by vacuum filtration, washed with additional water, and dried under vacuum. The crude material was chromatographed once using 0 to 100% ethyl acetate in hexanes then 0 to 100% methanol in ethyl acetate, then chromatographed a second time using 0 to 100% methanol in ethyl acetate to provide dihydropyrimidine **26g** (105 mg, 0.22 mmol, 22%) as a yellow solid. ^{1}H NMR (400 MHz, DMSO-D_6) δ 7.51 (d, J = 8.4 Hz, 2H), 7.31 (dd, J = 5.8, 2.3 Hz, 7H), 6.62 (s, 2H), 5.38 (s, 1H), 3.71 (q, J = 5.9 Hz, 2H), 2.69 (t, J = 2.6 Hz, 1H), 1.64 (td, J = 7.2, 2.6 Hz, 2H), 1.28 (p, J = 7.0 Hz, 2H) (one broad N-H singlet buried underneath the aromatic signals is unreported). ^{19}F NMR (471 MHz, DMSO-D_6) δ -64.52. ^{13}C NMR (126 MHz, DMSO-D_6) δ 165.64, 154.49, 147.49, 140.55, 128.21, 127.89, 127.43, 127.30, 126.83 (d, J = 1.4 Hz), 126.69, 122.00 (q, J = 274.7 Hz), 97.85, 83.57, 71.25, 61.63, 52.39, 28.11 (q, J = 39.9 Hz), 27.03, 14.35. HRMS (ESI$^+$), $C_{24}H_{21}F_3N_5O_2^+$ $[M+H]^+$: calc. mass 468.1642, found 468.1634.

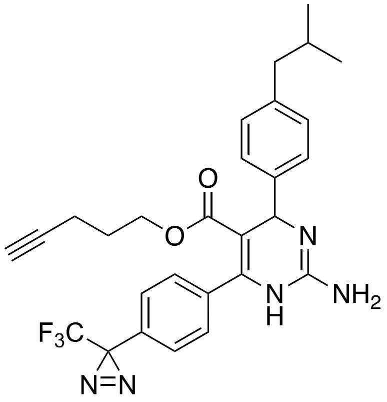

26h 28h

4-Isobutylbenzaldehyde (162 mg, 1.00 mmol, 1.00 eq), keto ester **S6** (372 mg, 1.10 mmol, 1.10 eq), guanidine carbonate (108 mg, 1.20 mmol, 1.20 eq), and sodium bicarbonate (336 mg, 4.00 mmol, 4.00 eq) were combined in DMF (2.0 mL) and stirred with heating in an oil bath set to 70 °C. After 80 minutes, the reaction was diluted with ethyl acetate (40 mL), washed with water (1x 40 mL), saturated sodium chloride solution (1x 40 mL), dried with sodium sulfate, and concentrated under vacuum. The crude material was chromatographed using 0 to 100% ethyl acetate in hexanes then 0 to 100% methanol in ethyl acetate to provide dihydropyrimidine **26h** (109 mg, 0.21 mmol, 21%) as a yellow solid. ^{1}H NMR (500 MHz, DMSO-D_6) δ 7.56 (s, br, 1H), 7.37 (d, J = 8.3 Hz, 2H), 7.26 (d, J = 8.1 Hz, 2H), 7.20 (d, J = 8.1 Hz, 2H), 7.12 (d, J = 8.0 Hz, 2H), 6.38 en(s, br, 2H), 5.28 (s, 1H), 3.78 – 3.60 (m, 2H), 2.69 (t, J = 2.7 Hz, 1H), 2.42 (d, J = 7.2 Hz, 2H), 1.81 (hept, J = 6.7 Hz, 1H), 1.61 (td, J = 7.3, 2.6 Hz, 2H), 1.27 (p, J = 7.0 Hz, 2H), 0.86 (d, J = 6.7 Hz, 6H). ^{19}F NMR (471 MHz, DMSO-D_6) δ -64.44. ^{13}C NMR (126 MHz, DMSO-D_6) δ 166.14, 160.11, 155.83, 144.99, 143.86, 140.68, 122.53 (q, J = 274.7 Hz), 98.34, 83.83, 71.67, 61.78, 53.11, 44.87, 30.18, 28.69 (q, J = 39.8 Hz), 27.61, 22.71 (d, J = 2.2 Hz), 14.83. HRMS (ESI$^+$), $C_{28}H_{29}F_3N_5O_2^+$ [M+H]$^+$: calc. mass 524.2268, found 524.2267.

Antibacterial Microdilution Assay (MIC Determination)

The MIC determination assays were performed in a BSL-2 laboratory with appropriate security and biosafety measures. The MICs were determined using a broth microdilution assay closely following the guidelines of the Clinical and Laboratories Standards Institute (CLSI). MICs were determined in duplicate for two biological replicates using 2-fold serial dilutions of test compounds or commercial antibiotics in cation-adjusted Mueller-Hinton broth. The central 60 wells of 96-well plates were prepared with the compound of interest at the indicated concentration, where 2 μL of the compound in DMSO were added prior to the addition of the culture. Overnight cultures of the different *Staphylococcus aureus* strains were diluted to provide an initial inoculum of approximately 5 X 10^5 CFU/mL that was delivered to each well in 198 μL of culture (final DMSO concentration of ~1%). Initial inoculum concentrations were verified by plating to count CFU/mL. Each plate was equipped with experimental controls for full growth (6 wells) and full growth inhibition (6 wells), and all control wells were in the presence of 1% DMSO. The outer 36 wells were filled with 200 μL of culture each to prevent growth effects on the interior 60 wells. The plates were incubated at 37 °C for 18 hours, at which time they were removed from the incubator and were allowed to equilibrate to room temperature for 30 minutes. Absorbance of each well at 600 nm was then read using a microplate reader to determine the MIC, and visual confirmation of growth inhibition was also performed. If variation was seen in the experimental values, the highest value of two independent trials was listed.

Hemolysis Assay

1.5 mL of mechanically defibrinated sheep blood (Hemostat Labs: DSB100) was placed in a microcentrifuge tube and was centrifuged at 10,000 rpm for ten minutes. Following the removal of the supernatant, the cells were resuspended in 1 mL of phosphate-buffered saline (PBS). This process was repeated two more times. The final suspension was diluted ten-fold with PBS and was aliquoted into microcentrifuge tubes containing the compounds which were previously serial diluted in PBS. TritonX (1% by volume) served as a positive control, while sterile PBS served as a negative control. All samples were incubated at 37 °C and shaken at 200 rpm for 1 hour. After the 1 hour, the samples were centrifuged at 10,000 rpm for 5 minutes and the absorbance of the supernatant was measured via UV spectroscopy at a 540 nm wavelength. The concentrations that produced >20% hemolysis are noted in the manuscript.

MTT Assay

Mammalian cytotoxicity was evaluated using a commercially available MTT Cell proliferation assay (ATCC 30-1010K; Manassas, VA) following the included protocol. Briefly, Human Embryonic Kidney 293 (HEK-293; ATCC CRL-1353) cells were seeded onto 96 well plates (Nunclon Delta; ThermoFisher 167008; Waltham, MA) coated with Poly-D-Lysine (Thermofisher A3890401) at a density of 100,00 cells per well in 80 µL of Eagle's Minimum Essential Medium (EMEM; ATCC 30-2003) with 10% Fetal Bovine Serum (ATCC 30-2021). Cells were incubated at 37 °C in a 5% CO2 atmosphere and allowed to adhere overnight, and then treated for 24 h with 20 µL of drugs dissolved in PBS with 0.5% DMSO, at concentrations ranging from 1×10-4 M to 1×10-9 M. Following treatment, 10 µL of MTT reagent was added to each well and allowed to incubate at 37 °C for 4 h. Then, 100 µL of detergent reagent was added to all wells, and they were stored at room temperature in the dark for 2 h. Absorbance was then read using a multi-mode spectrophotometer (Spectramax i3X; San Jose, CA) at a wavelength of 570 nm. Raw values were normalized to a 0-100% scale using the maximum and minimum responses. To generate dose-response curves and IC50 values, normalized data were plotted against the log of the drug concentrations and fit to nonlinear curve [log(inhibitor) vs. response – variable slope (four parameters)] using GraphPad Prism version 7.05 (GraphPad Software; San Diego, CA).

NMR Spectra:

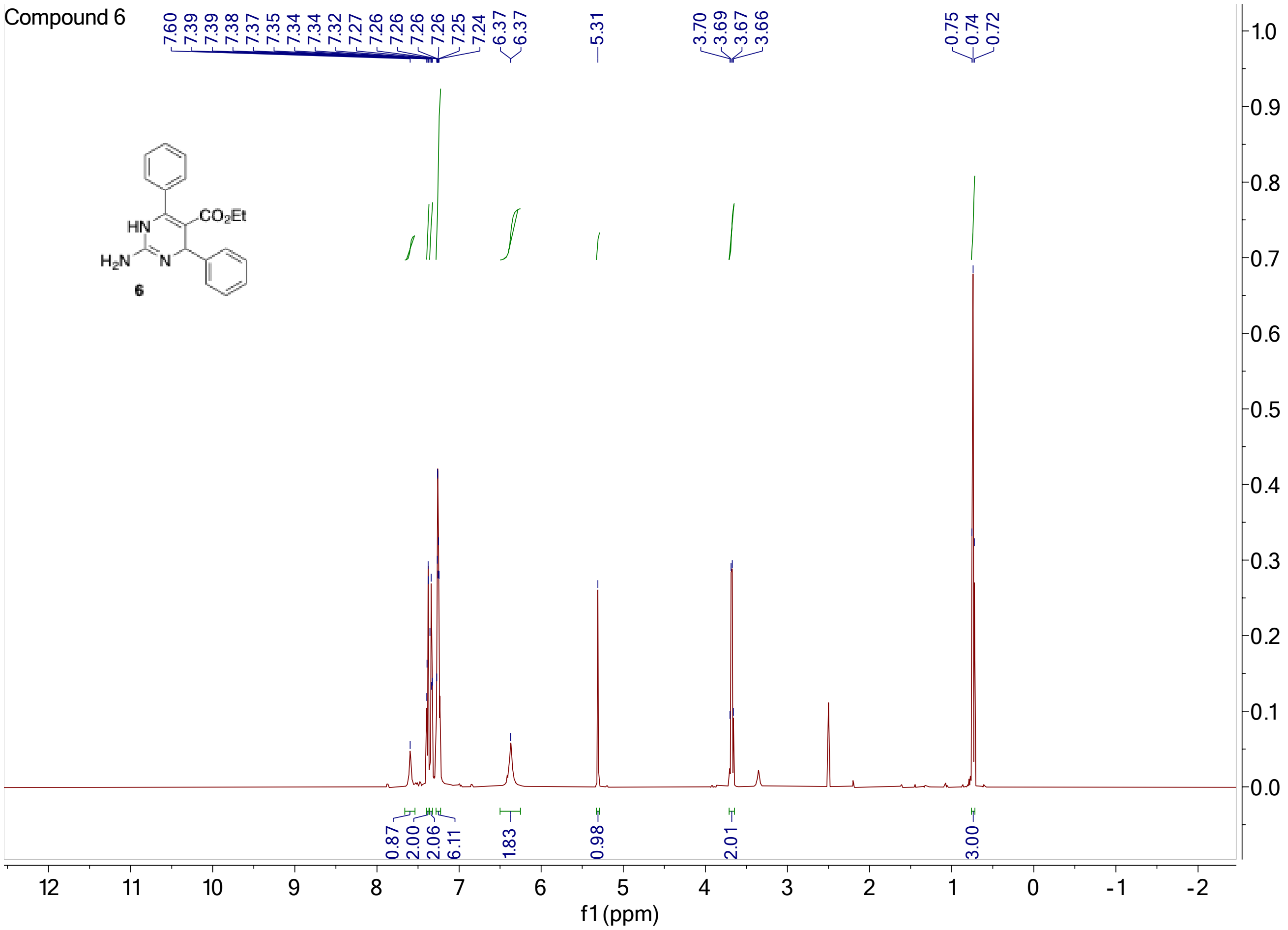
Compound 6
7.60
7.39
7.39
7.38
7.37
7.35
7.34
7.34
7.32
7.27
7.26
7.26
7.26
7.26
7.25
7.24
6.37
6.37
5.31
3.70
3.69
3.67
3.66
0.75
0.74
0.72
CO2Et
HN
H2N
N
6
0.87
2.00
2.06
6.11
1.83
0.98
2.01
3.00
f1 (ppm)

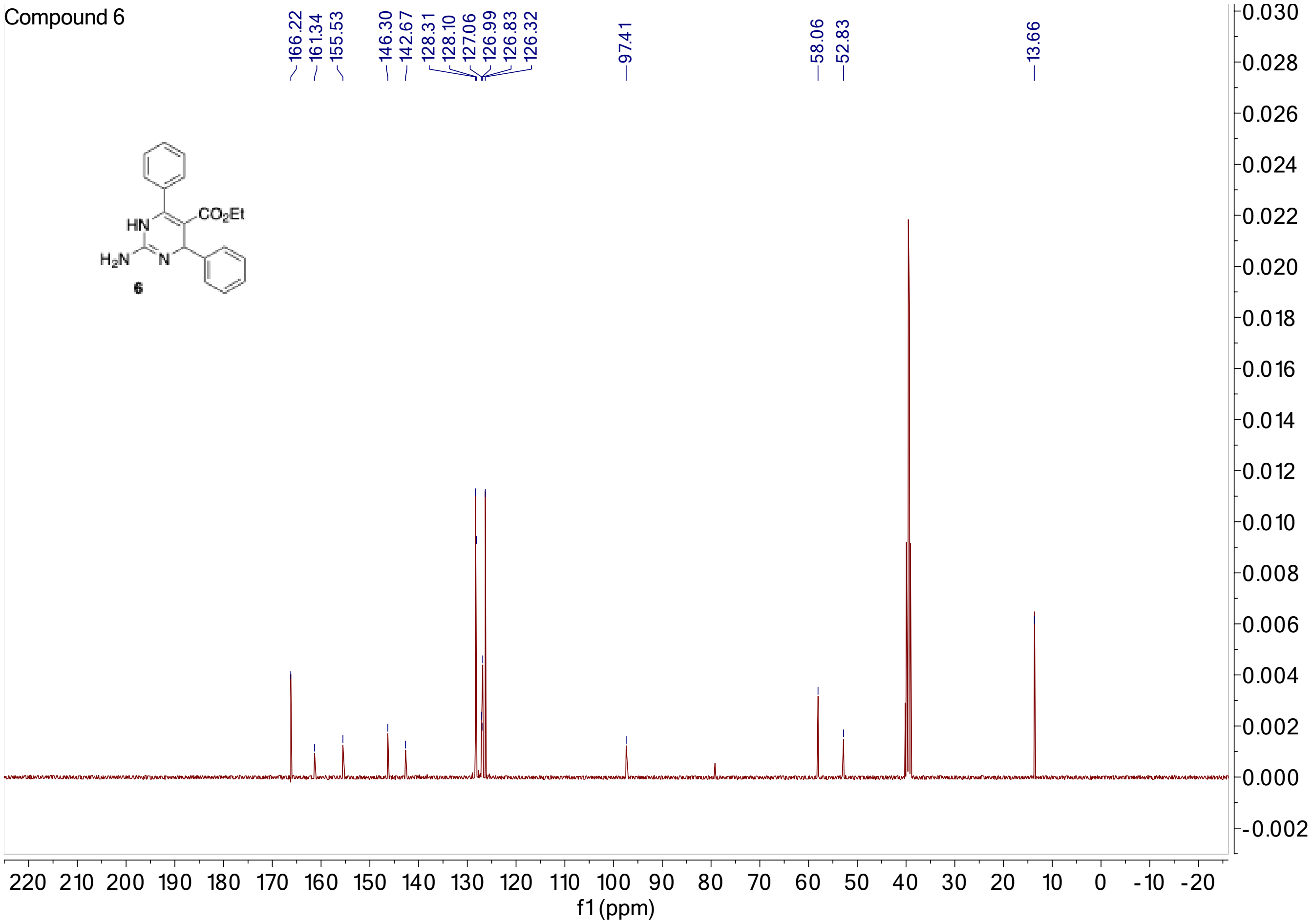
Compound 6
166.22
161.34
155.53
146.30
142.67
128.31
128.10
127.06
126.99
126.83
126.32
97.41
58.06
52.83
13.66
CO2Et
HN
H2N
N
6
f1 (ppm)
220 210 200 190 180 170 160 150 140 130 120 110 100 90 80 70 60 50 40 30 20 10 0 -10 -20
0.030 0.028 0.026 0.024 0.022 0.020 0.018 0.016 0.014 0.012 0.010 0.008 0.006 0.004 0.002 0.000 -0.002

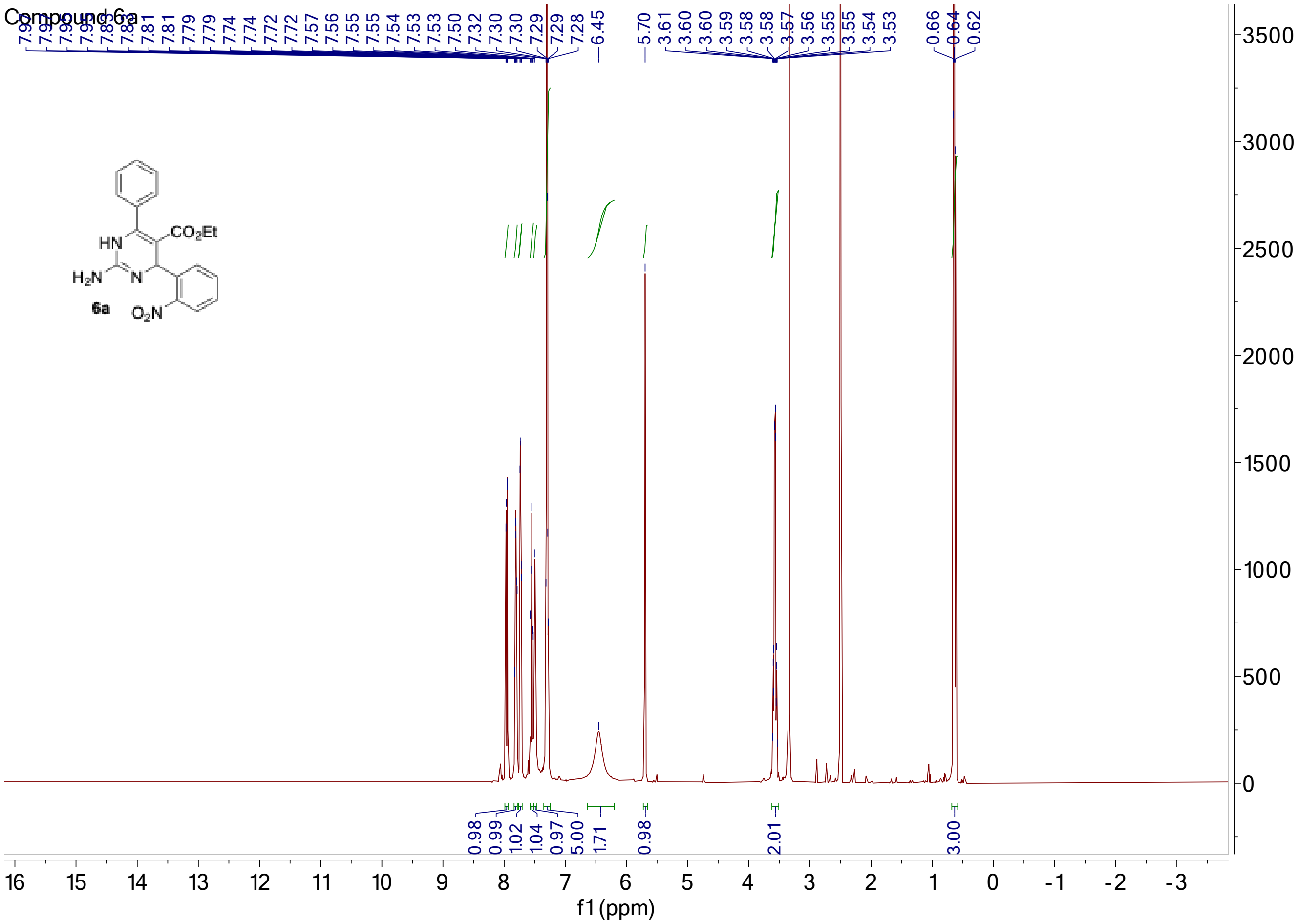
Compound 6a
6a
f1 (ppm)

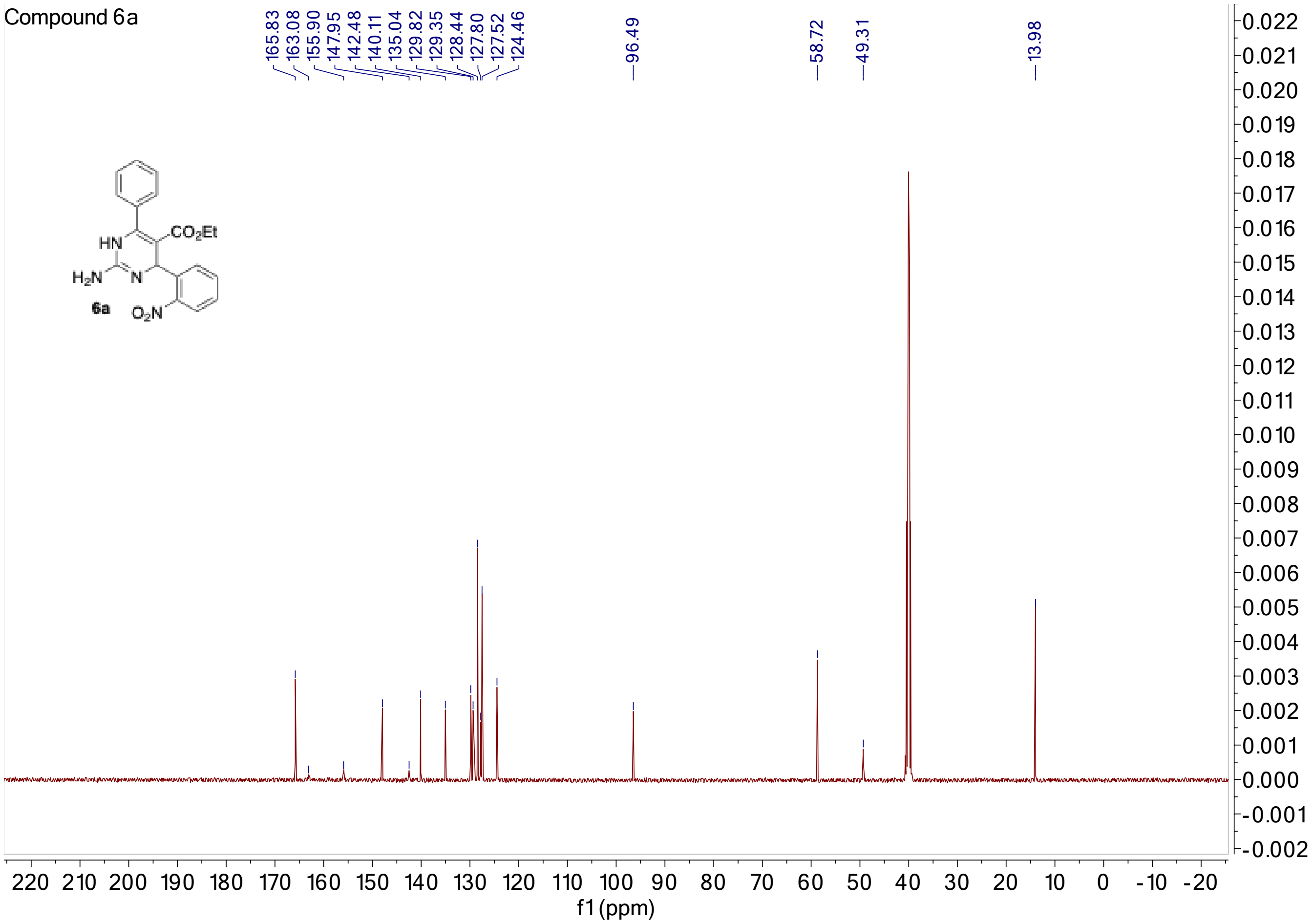
Compound 6a
165.83
163.08
155.90
147.95
142.48
140.11
135.04
129.82
129.35
128.44
127.80
127.52
124.46
96.49
58.72
49.31
13.98
CO2Et
HN
H2N
N
6a
O2N
f1 (ppm)

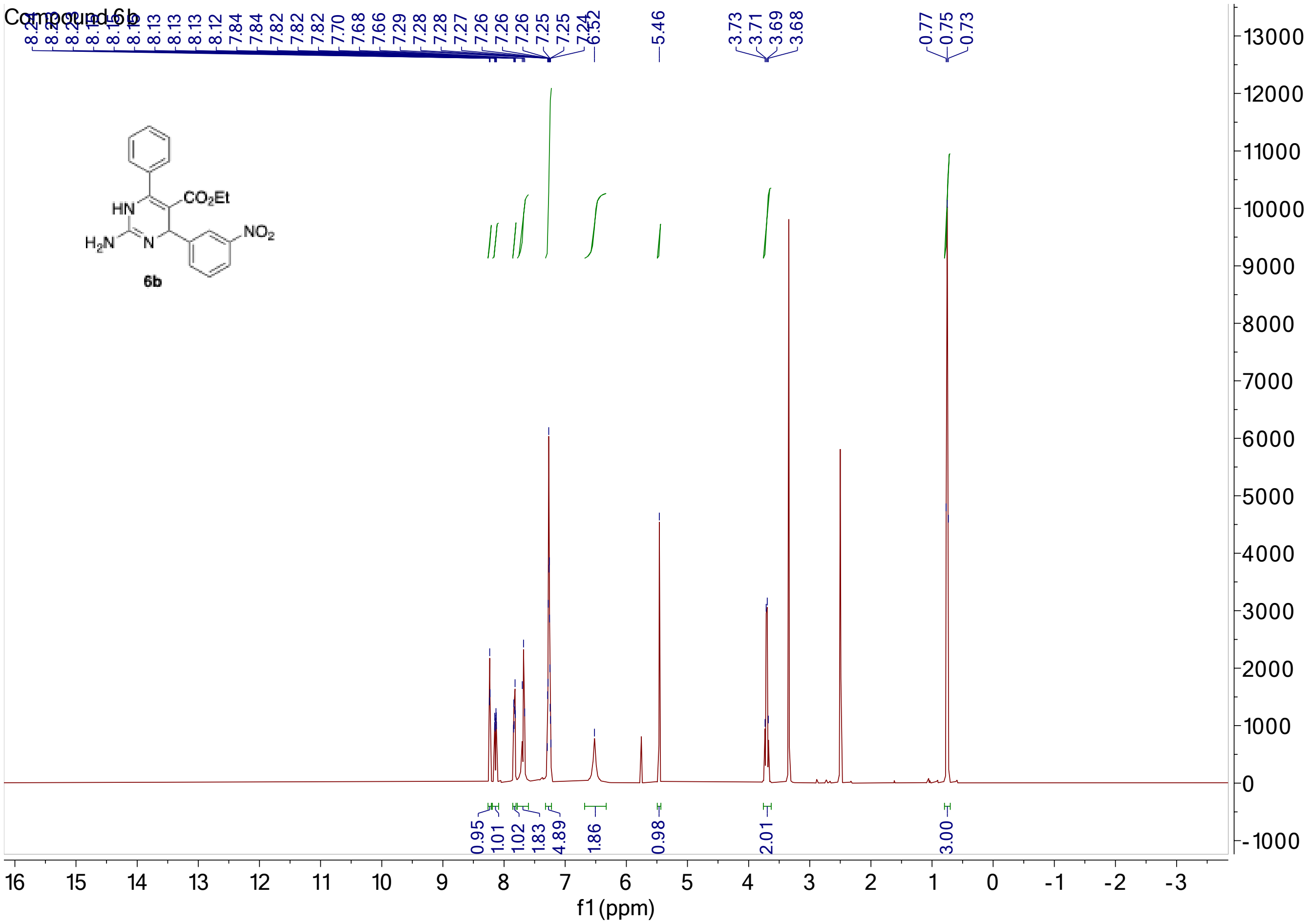
Compound 6b
6b
f1 (ppm)

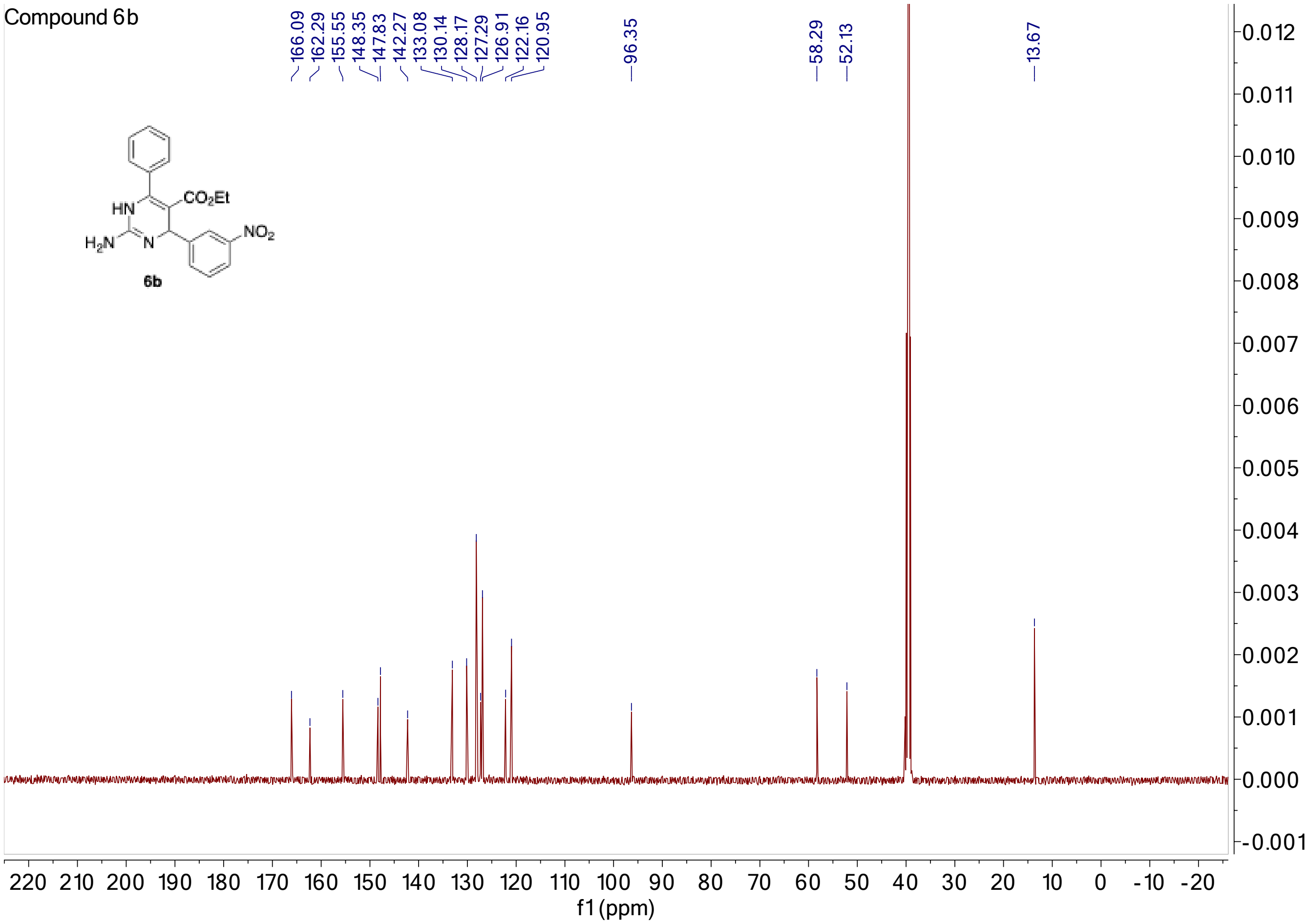
Compound 6b
166.09
162.29
155.55
148.35
147.83
142.27
133.08
130.14
128.17
127.29
126.91
122.16
120.95
96.35
58.29
52.13
13.67
HN
CO2Et
NO2
H2N
N
6b
0.012
0.011
0.010
0.009
0.008
0.007
0.006
0.005
0.004
0.003
0.002
0.001
0.000
-0.001
220 210 200 190 180 170 160 150 140 130 120 110 100 90 80 70 60 50 40 30 20 10 0 -10 -20
f1 (ppm)

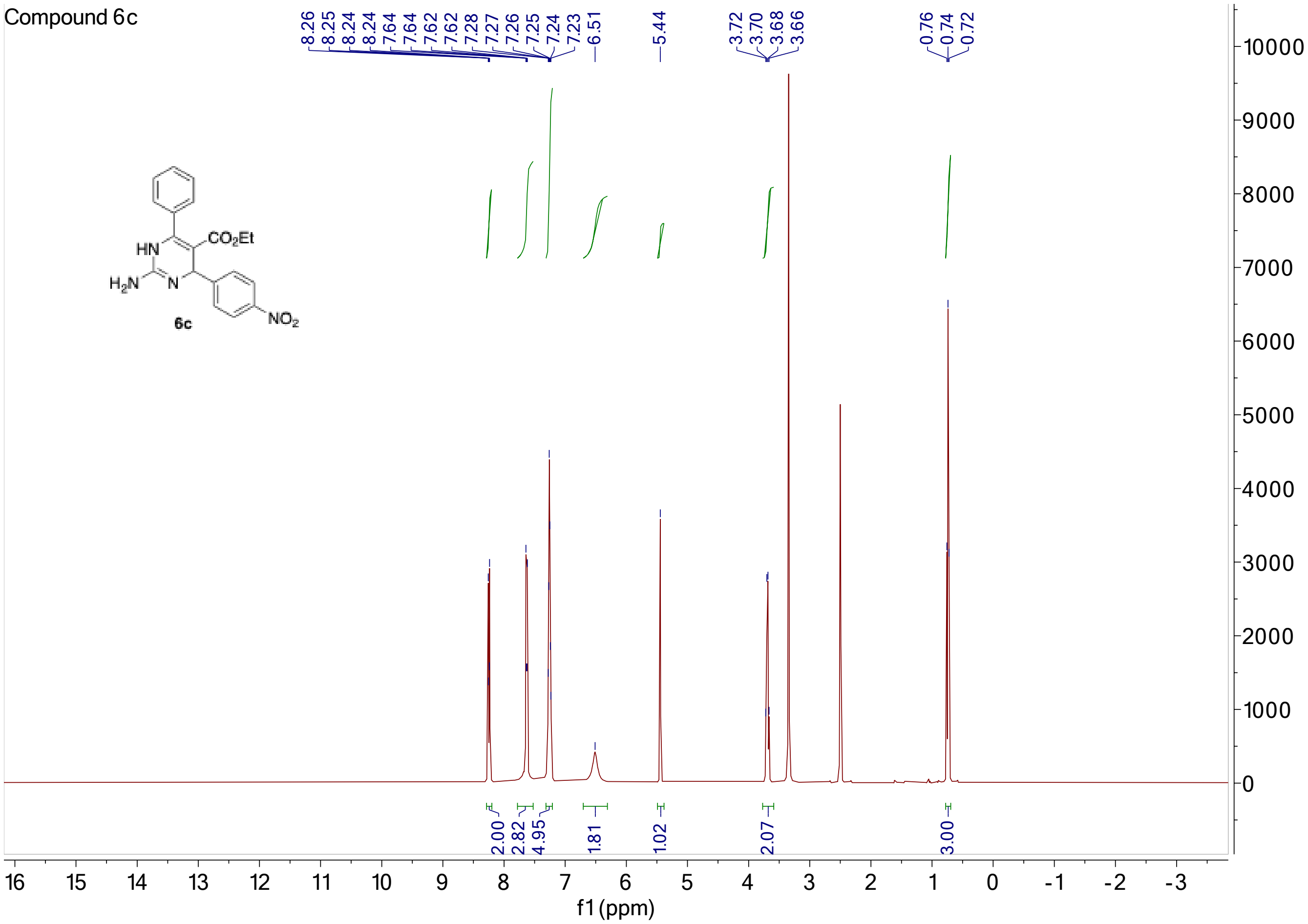
Compound 6c
8.26
8.25
8.24
8.24
7.64
7.64
7.62
7.62
7.28
7.27
7.26
7.25
7.24
7.23
6.51
5.44
3.72
3.70
3.68
3.66
0.76
0.74
0.72
HN
CO2Et
H2N
N
NO2
6c
2.00
2.82
4.95
1.81
1.02
2.07
3.00
f1 (ppm)

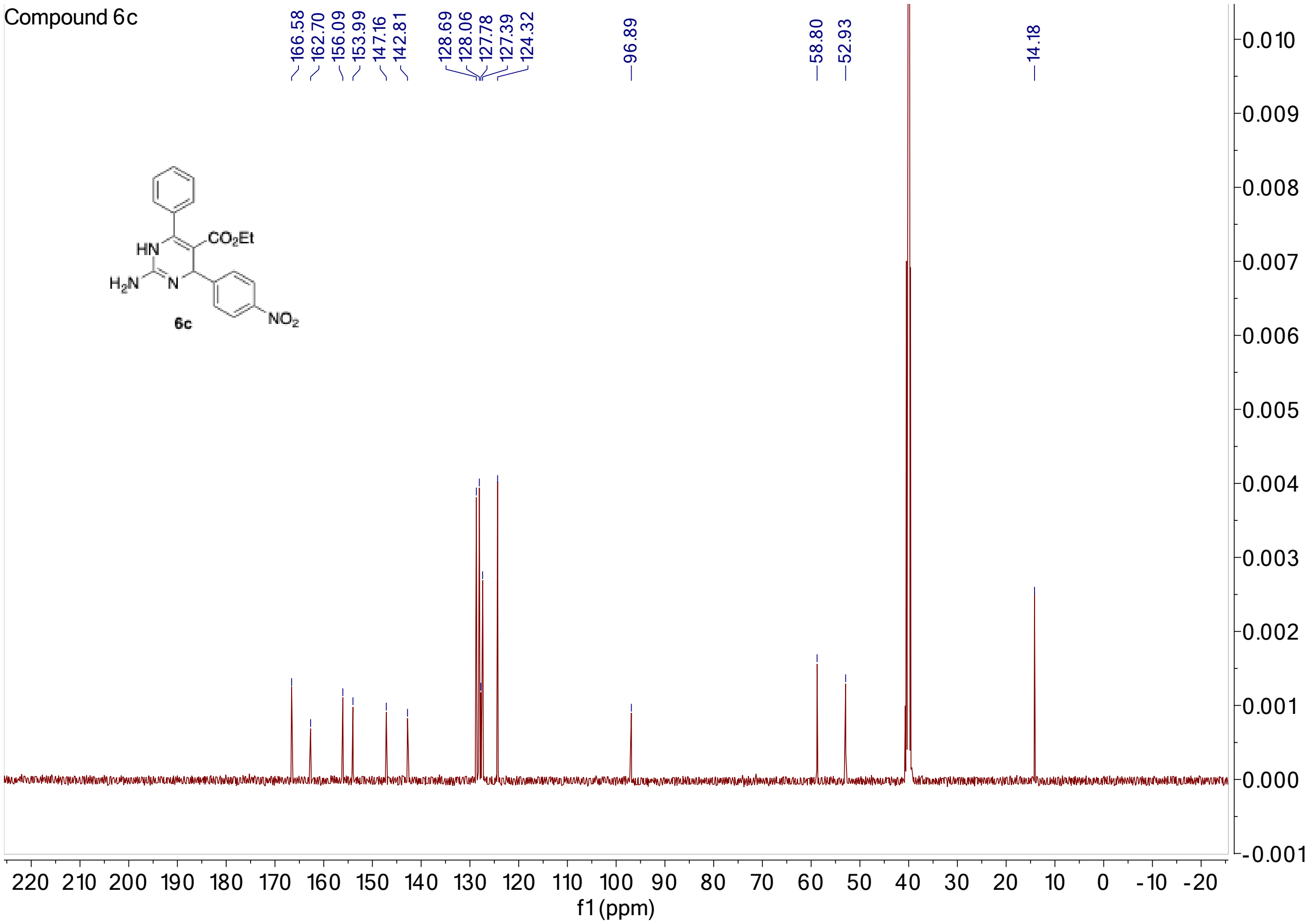
Compound 6c
166.58
162.70
156.09
153.99
147.16
142.81
128.69
128.06
127.78
127.39
124.32
96.89
58.80
52.93
14.18
HN
CO2Et
H2N
N
NO2
6c
0.010
0.009
0.008
0.007
0.006
0.005
0.004
0.003
0.002
0.001
0.000
-0.001
220 210 200 190 180 170 160 150 140 130 120 110 100 90 80 70 60 50 40 30 20 10 0 -10 -20
f1 (ppm)

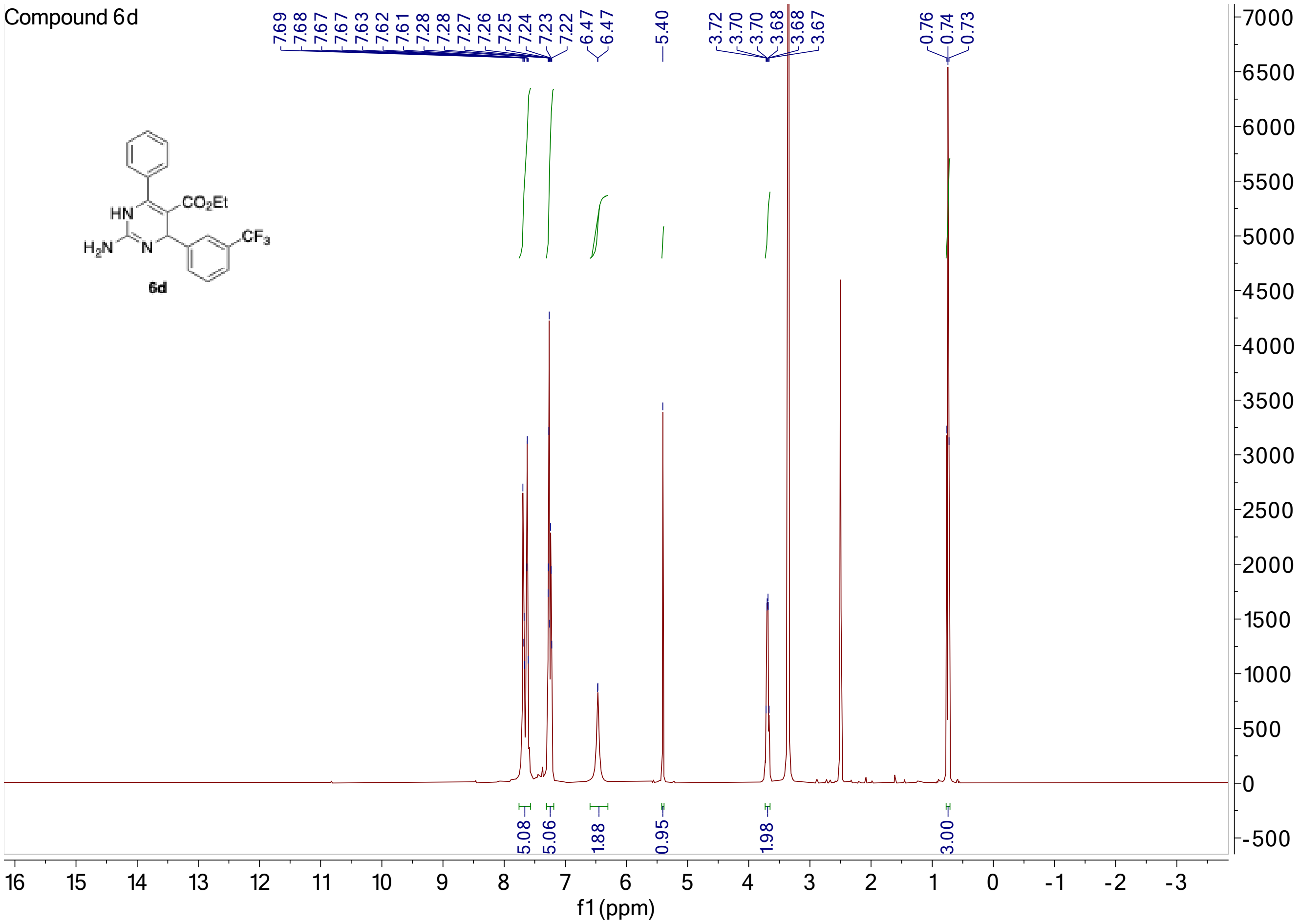
Compound 6d
7.69
7.68
7.67
7.67
7.63
7.62
7.61
7.28
7.28
7.27
7.26
7.25
7.24
7.23
7.22
6.47
6.47
5.40
3.72
3.70
3.70
3.68
3.68
3.67
0.76
0.74
0.73
CO2Et
HN
H2N
N
CF3
6d
5.08
5.06
1.88
0.95
1.98
3.00
f1 (ppm)

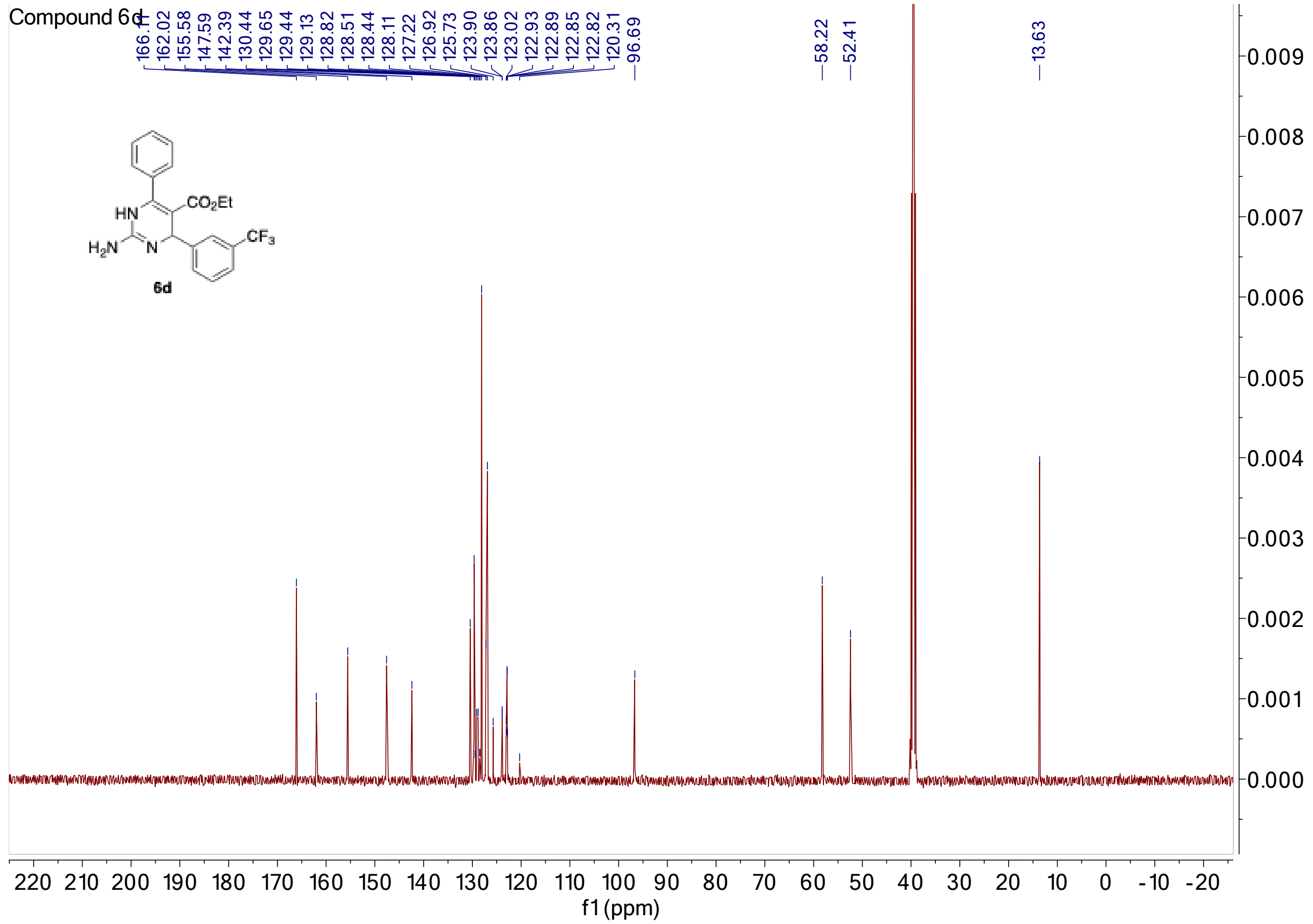
Compound 6d
166.11
162.02
155.58
147.59
142.39
130.44
129.65
129.44
129.13
128.82
128.51
128.44
128.11
127.22
126.92
125.73
123.90
123.86
123.02
122.93
122.89
122.85
122.82
120.31
96.69
58.22
52.41
13.63
CO2Et
HN
H2N
N
CF3
6d
0.009
0.008
0.007
0.006
0.005
0.004
0.003
0.002
0.001
0.000
220 210 200 190 180 170 160 150 140 130 120 110 100 90 80 70 60 50 40 30 20 10 0 -10 -20
f1 (ppm)

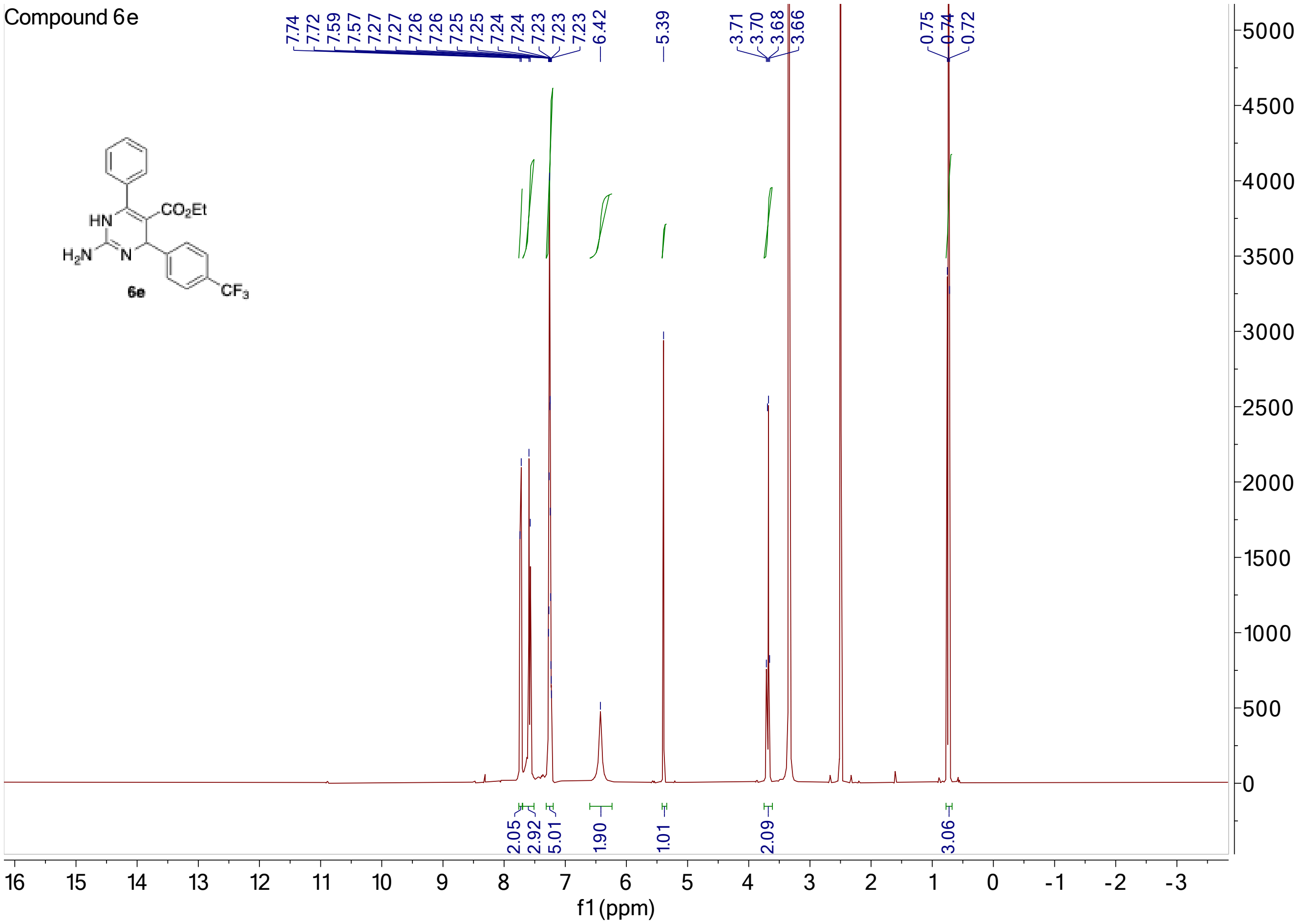
Compound 6e
7.74
7.72
7.59
7.57
7.27
7.27
7.26
7.26
7.25
7.25
7.24
7.24
7.23
7.23
7.23
6.42
5.39
3.71
3.70
3.68
3.66
0.75
0.74
0.72
2.05
2.92
5.01
1.90
1.01
2.09
3.06
CO2Et
HN
H2N
N
CF3
6e
f1 (ppm)

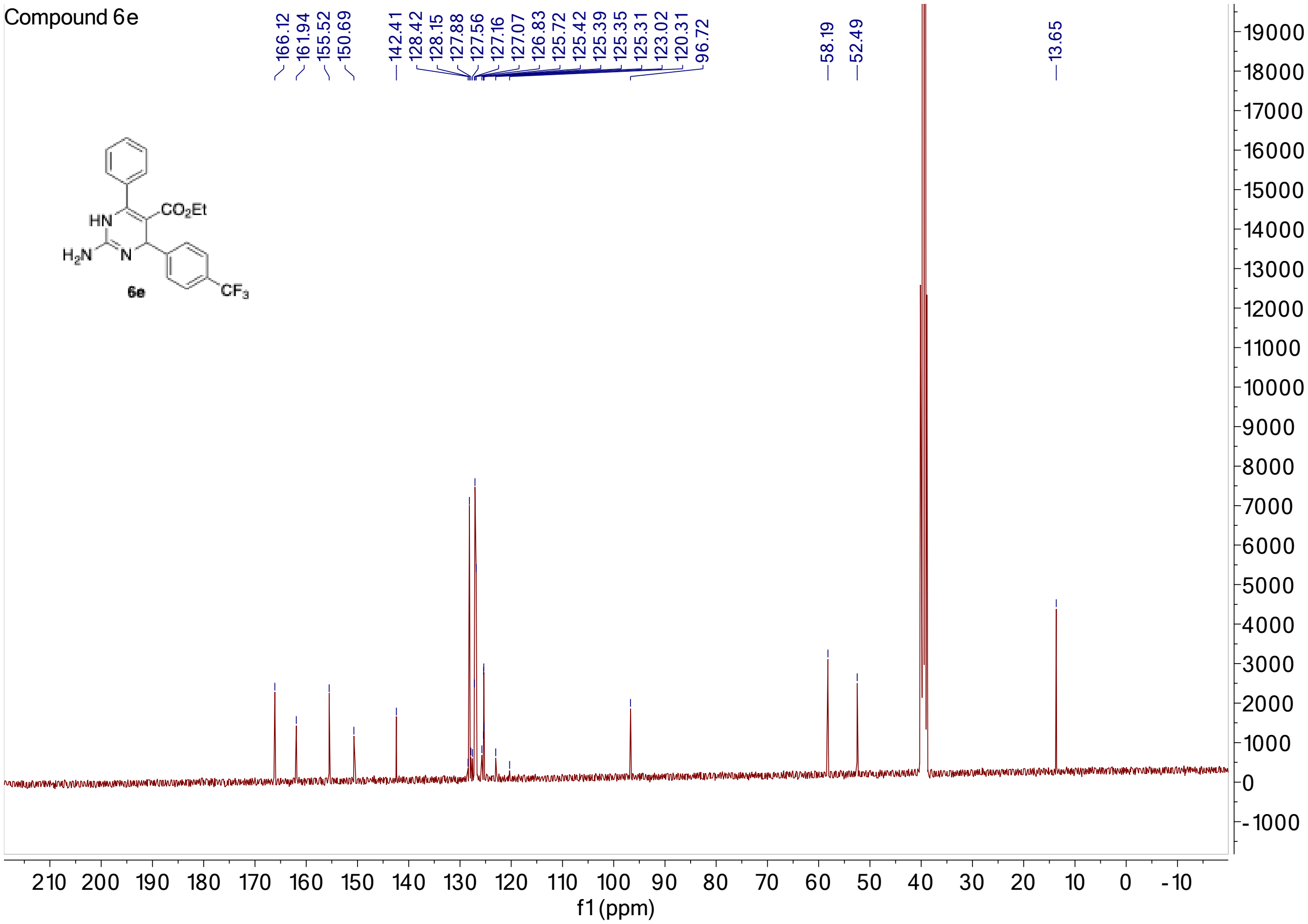
Compound 6e
166.12
161.94
155.52
150.69
142.41
128.42
128.15
127.88
127.56
127.16
127.07
126.83
125.72
125.42
125.39
125.35
125.31
123.02
120.31
96.72
58.19
52.49
13.65
HN
CO_2Et
H_2N
N
6e
CF_3
f1 (ppm)

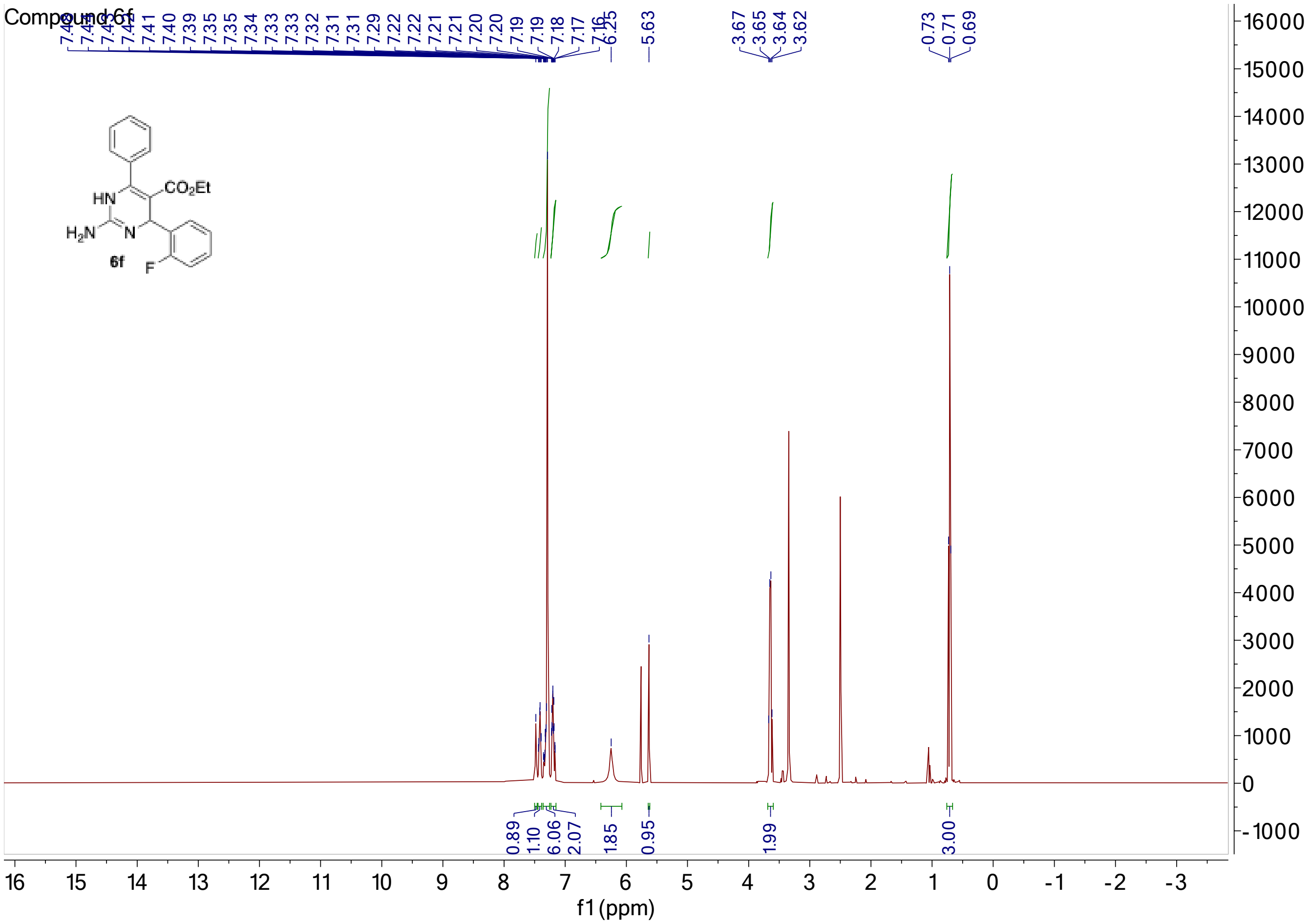
Compound 6f
7.41
7.40
7.39
7.35
7.35
7.34
7.33
7.33
7.32
7.31
7.31
7.29
7.22
7.22
7.21
7.21
7.20
7.20
7.19
7.19
7.18
7.17
6.25
5.63
3.67
3.65
3.64
3.62
0.73
0.71
0.69
CO2Et
HN
H2N
N
6f
F
0.89
1.10
6.06
2.07
1.85
0.95
1.99
3.00
f1 (ppm)

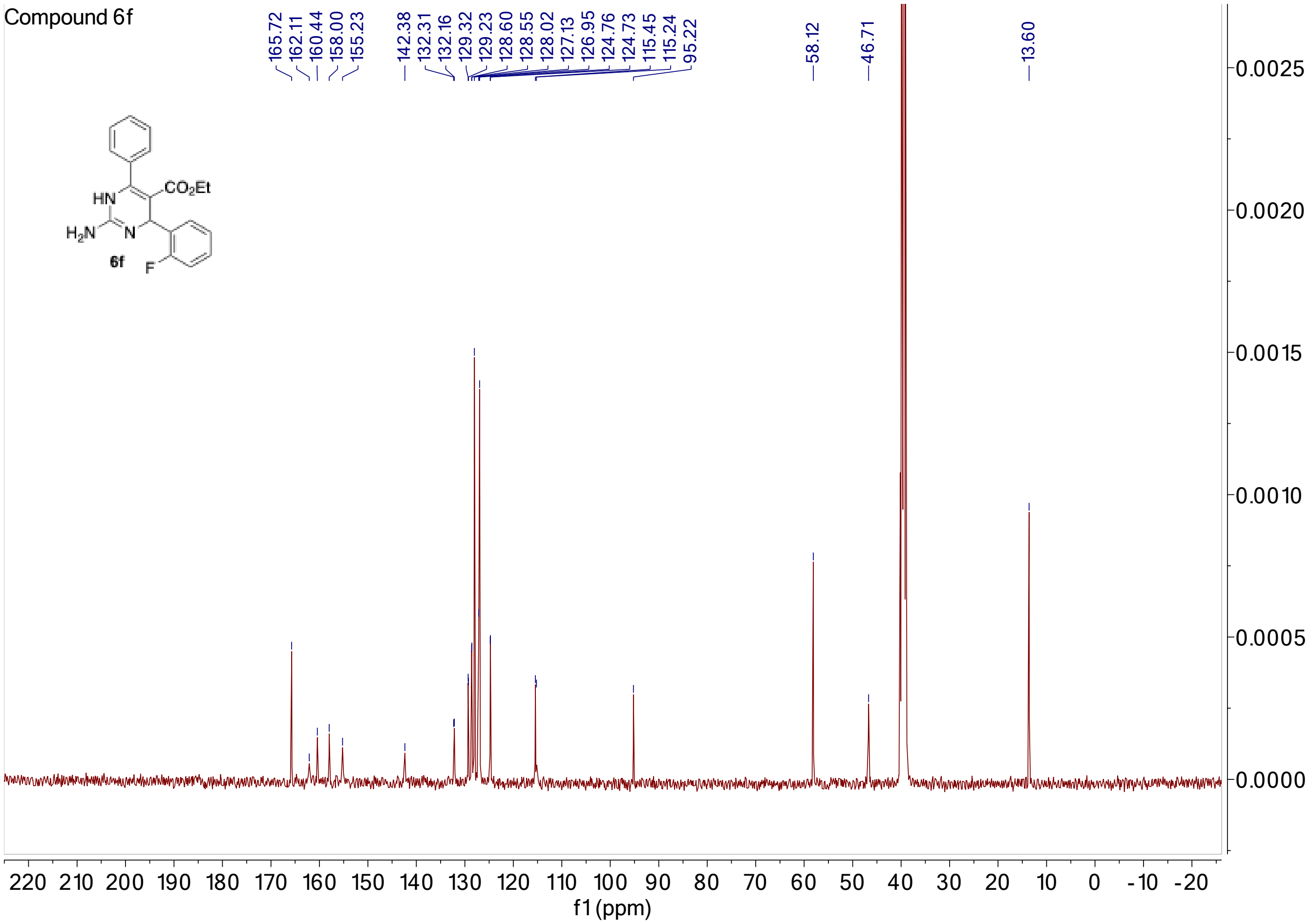
Compound 6f
165.72
162.11
160.44
158.00
155.23
142.38
132.31
132.16
129.32
129.23
128.60
128.55
128.02
127.13
126.95
124.76
124.73
115.45
115.24
95.22
58.12
46.71
13.60
CO2Et
HN
H2N
N
6f
F
0.0025
0.0020
0.0015
0.0010
0.0005
0.0000
220 210 200 190 180 170 160 150 140 130 120 110 100 90 80 70 60 50 40 30 20 10 0 -10 -20
f1 (ppm)

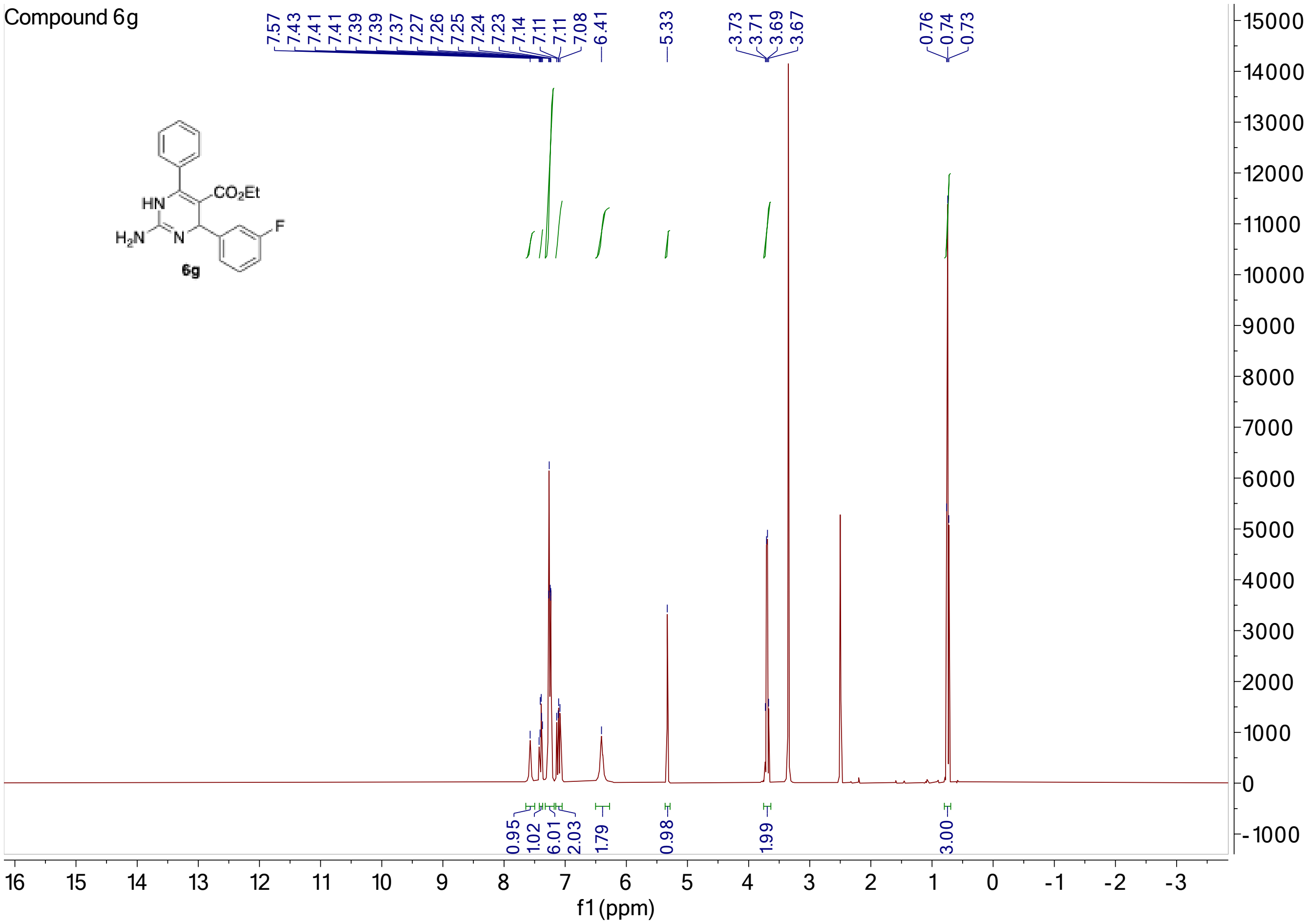
Compound 6g
7.57
7.43
7.41
7.41
7.39
7.39
7.37
7.27
7.26
7.25
7.24
7.23
7.14
7.11
7.11
7.08
6.41
5.33
3.73
3.71
3.69
3.67
0.76
0.74
0.73
HN
CO2Et
H2N
N
F
6g
0.95
1.02
6.01
2.03
1.79
0.98
1.99
3.00
f1 (ppm)

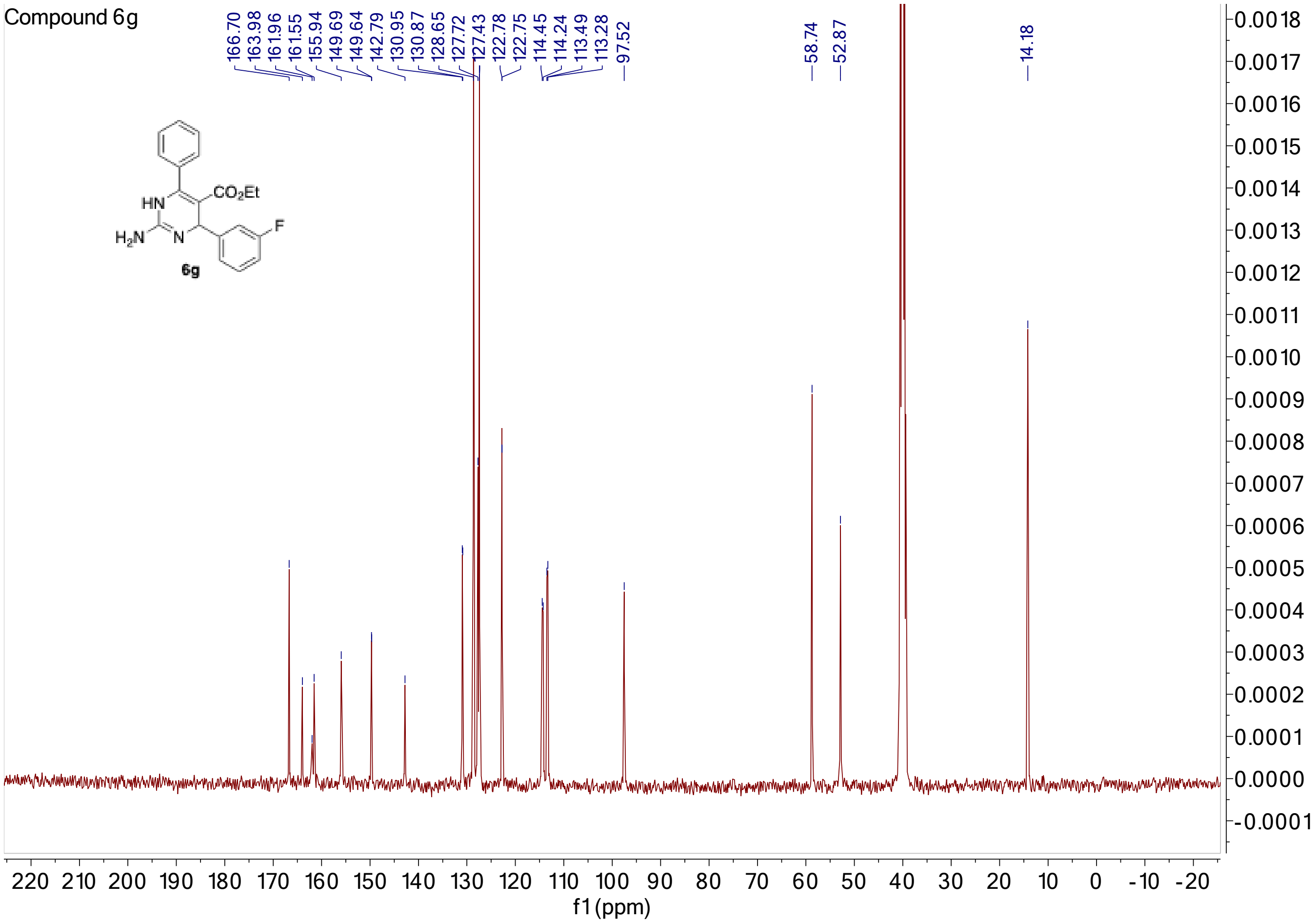
Compound 6g
166.70
163.98
161.96
161.55
155.94
149.69
149.64
142.79
130.95
130.87
128.65
127.72
127.43
122.78
122.75
114.45
114.24
113.49
113.28
97.52
58.74
52.87
14.18
CO2Et
HN
H2N
N
F
6g
0.0018
0.0017
0.0016
0.0015
0.0014
0.0013
0.0012
0.0011
0.0010
0.0009
0.0008
0.0007
0.0006
0.0005
0.0004
0.0003
0.0002
0.0001
0.0000
-0.0001
220 210 200 190 180 170 160 150 140 130 120 110 100 90 80 70 60 50 40 30 20 10 0 -10 -20
f1 (ppm)

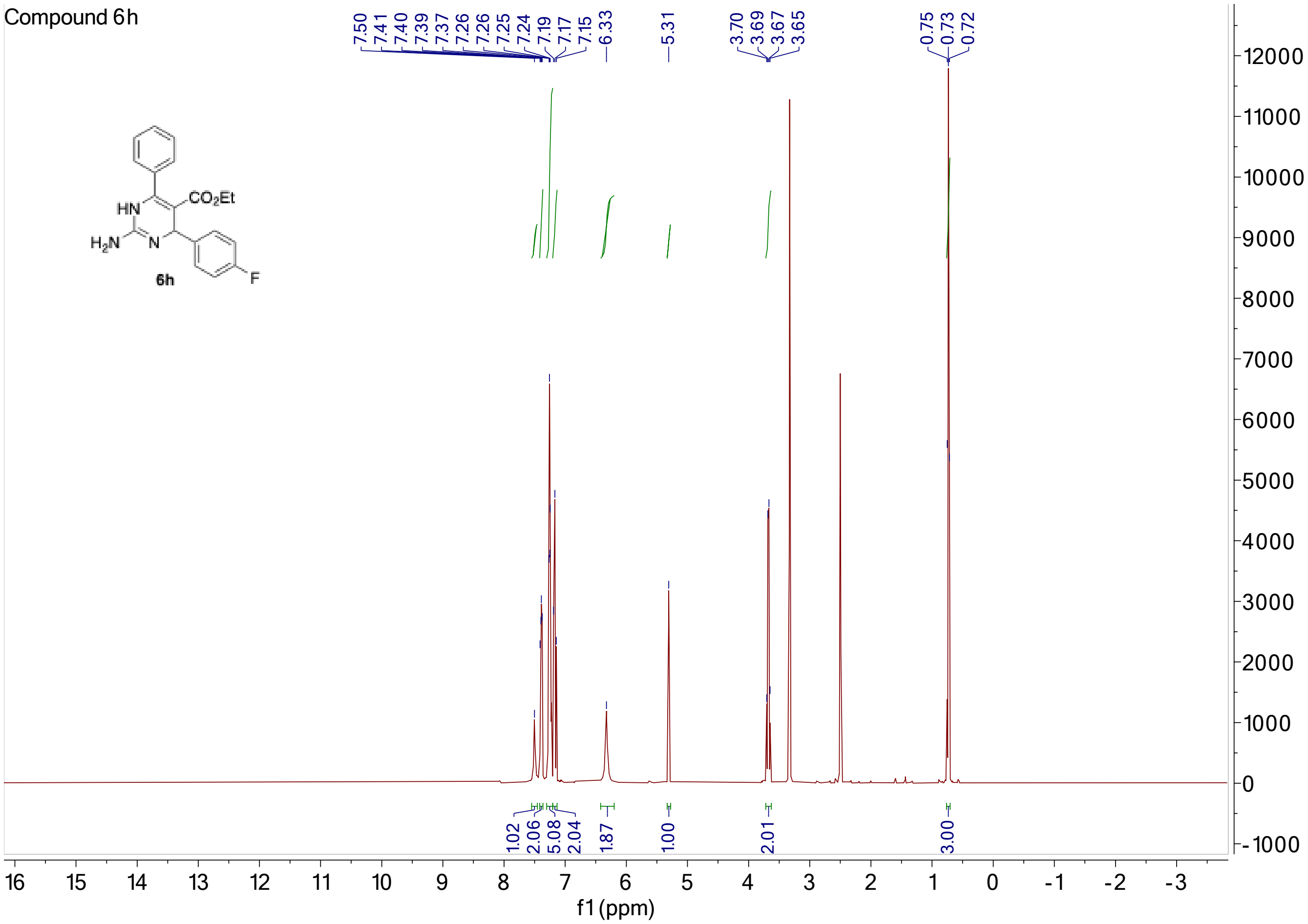
Compound 6h
7.50 7.41 7.40 7.39 7.37 7.26 7.26 7.25 7.24 7.19 7.17 7.15
6.33
5.31
3.70 3.69 3.67 3.65
0.75 0.73 0.72
HN
CO2Et
H2N
N
F
6h
1.02 2.06 5.08 2.04
1.87
1.00
2.01
3.00
f1 (ppm)

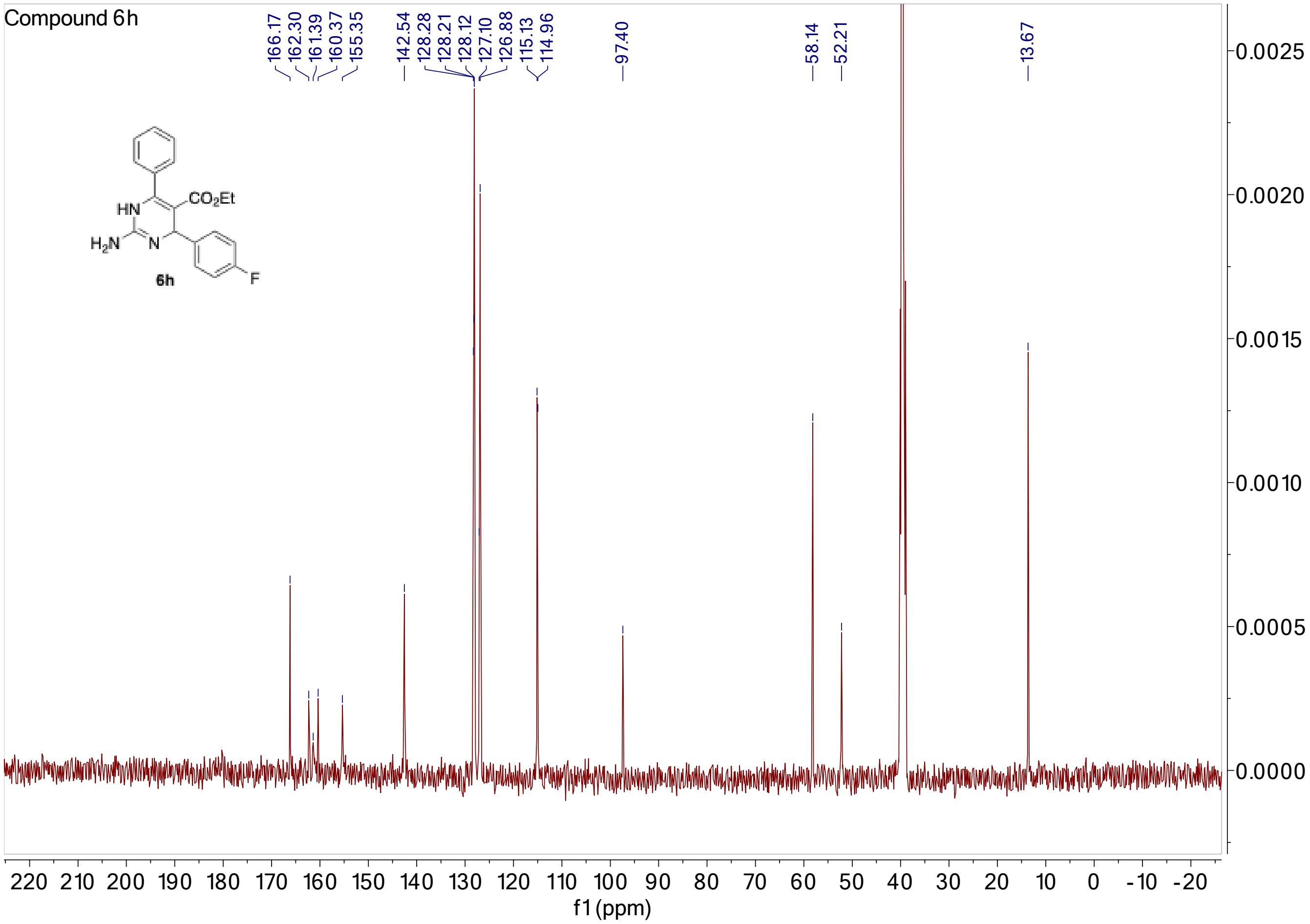
Compound 6h
166.17
162.30
161.39
160.37
155.35
142.54
128.28
128.21
128.12
127.10
126.88
115.13
114.96
97.40
58.14
52.21
13.67
HN
CO2Et
H2N
N
F
6h
0.0025
0.0020
0.0015
0.0010
0.0005
0.0000
220 210 200 190 180 170 160 150 140 130 120 110 100 90 80 70 60 50 40 30 20 10 0 -10 -20
f1 (ppm)

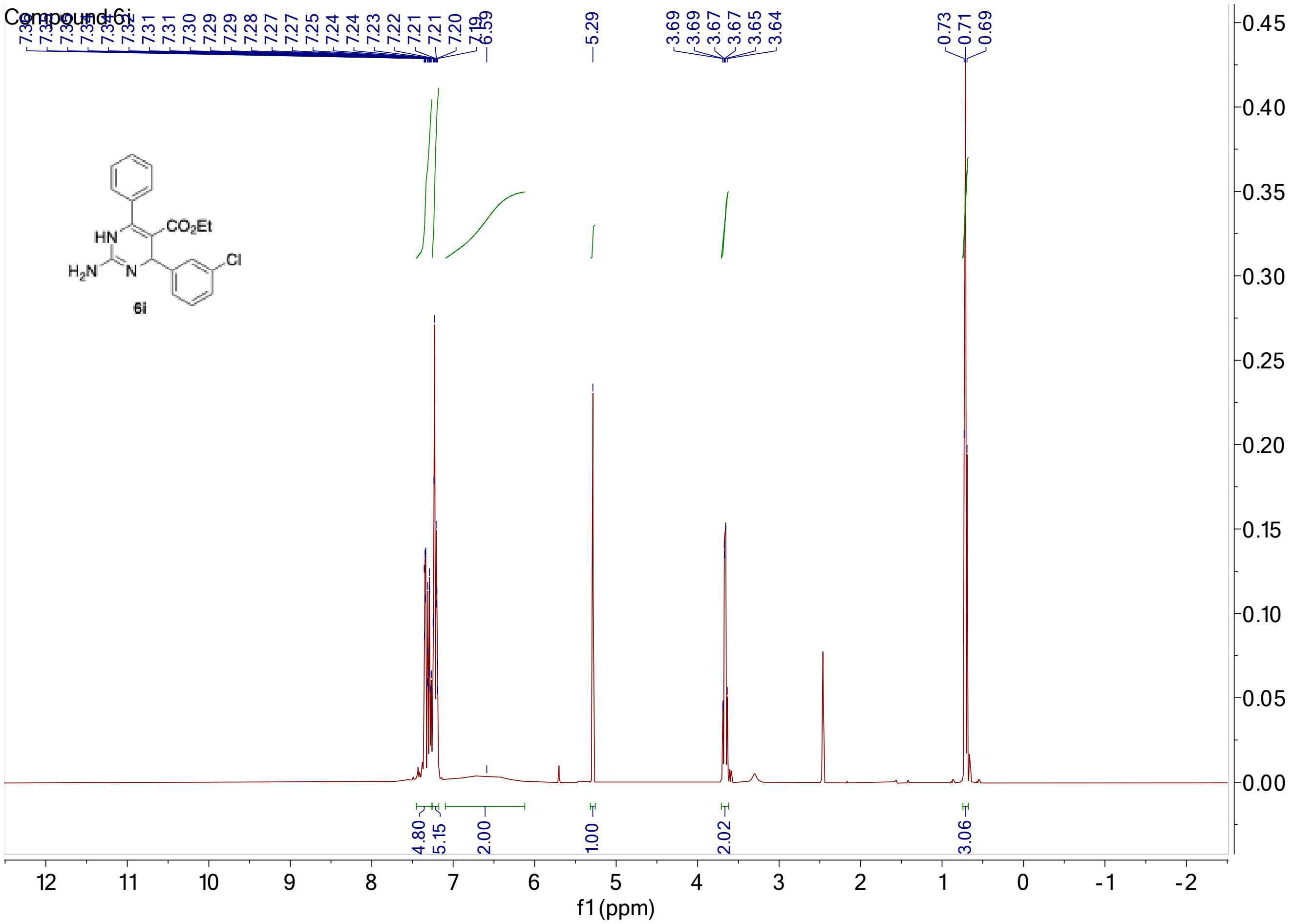
Compound 6i
7.21
7.20
7.19
6.59
5.29
3.69
3.69
3.67
3.67
3.65
3.64
0.73
0.71
0.69
CO2Et
HN
H2N
N
Cl
6i
4.80
5.15
2.00
1.00
2.02
3.06
f1 (ppm)

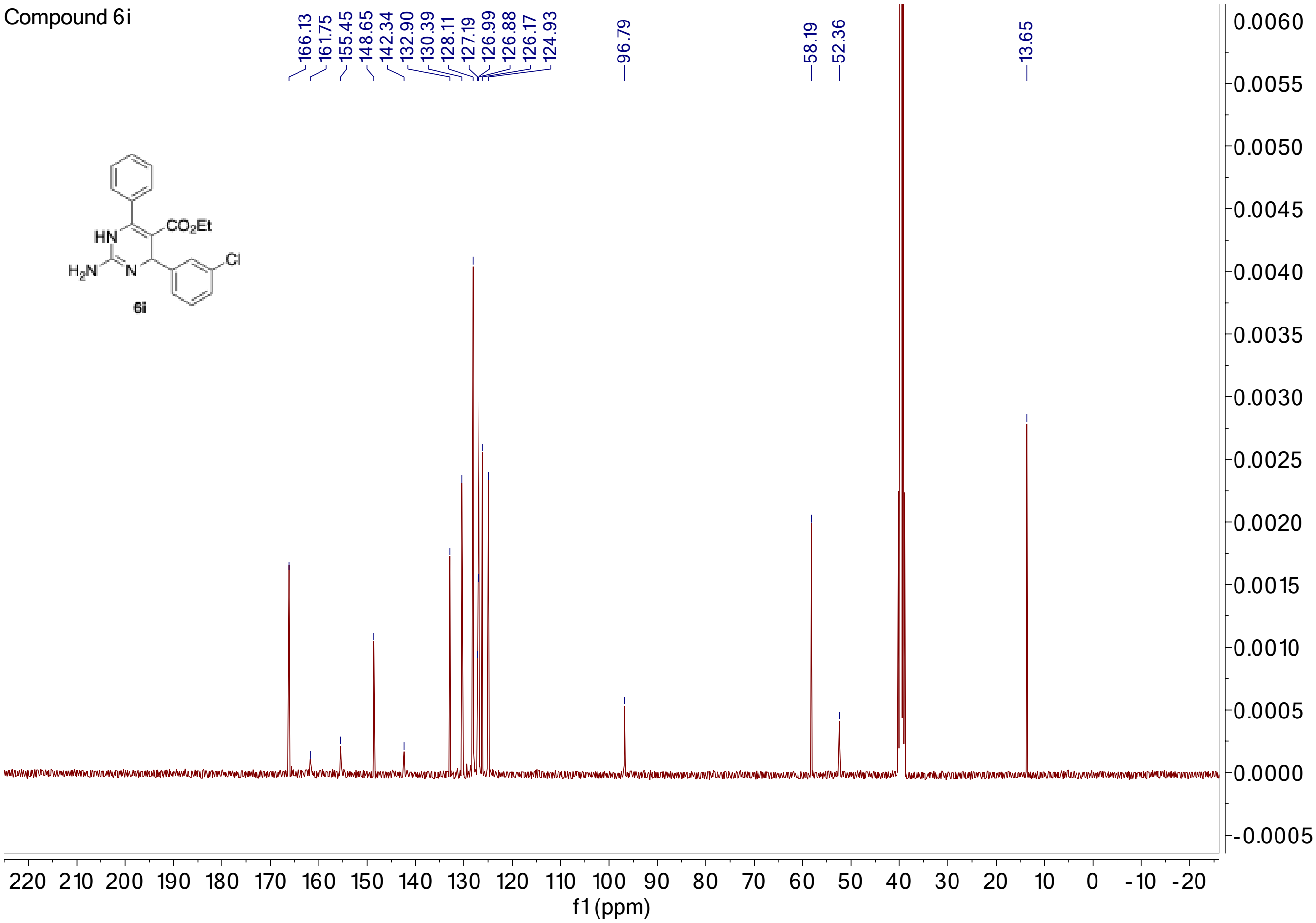
Compound 6i
166.13
161.75
155.45
148.65
142.34
132.90
130.39
128.11
127.19
126.99
126.88
126.17
124.93
96.79
58.19
52.36
13.65
CO2Et
HN
H2N
N
Cl
6i
0.0060
0.0055
0.0050
0.0045
0.0040
0.0035
0.0030
0.0025
0.0020
0.0015
0.0010
0.0005
0.0000
-0.0005
220 210 200 190 180 170 160 150 140 130 120 110 100 90 80 70 60 50 40 30 20 10 0 -10 -20
f1 (ppm)

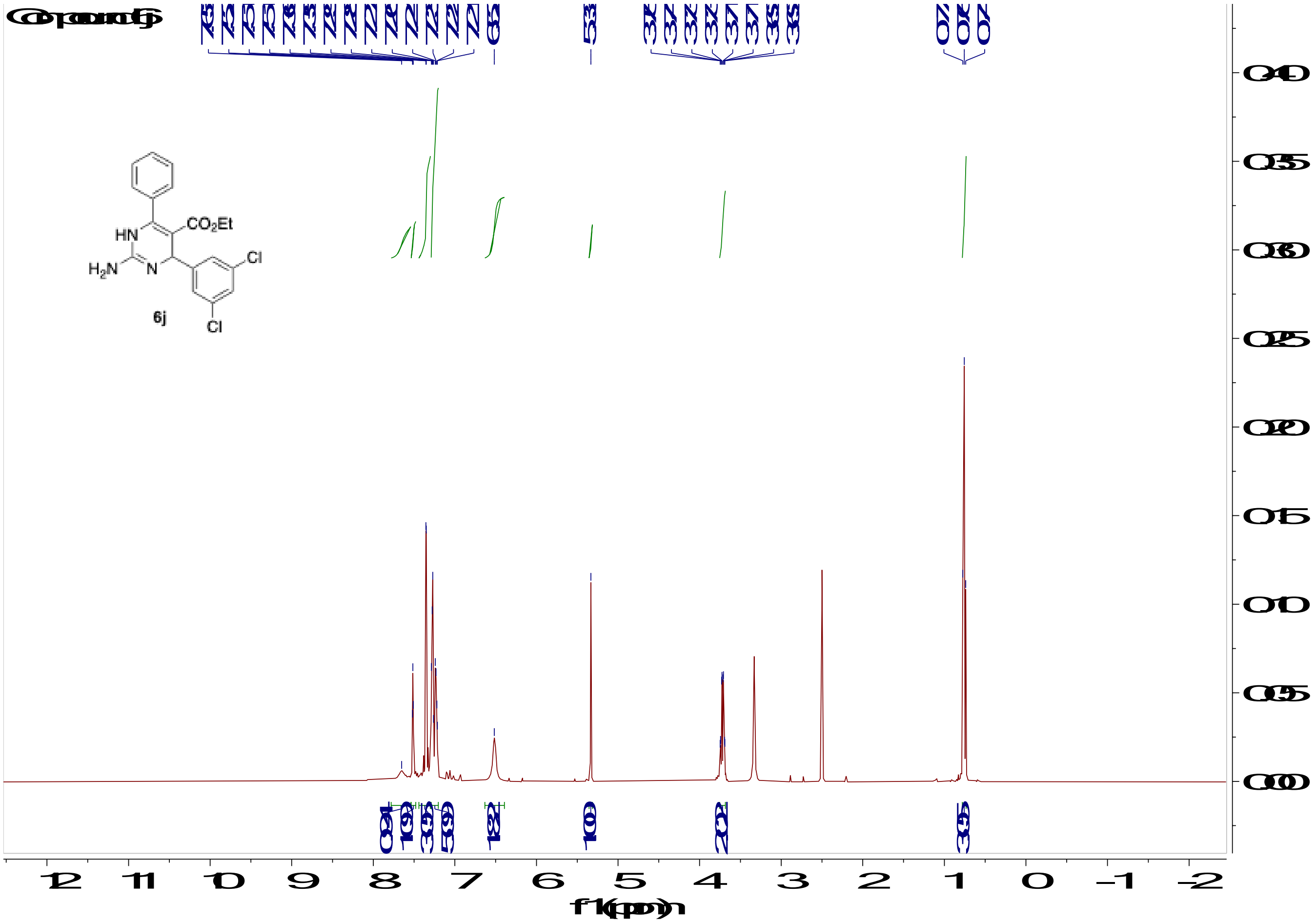
CO2Et
HN
H2N
N
Cl
6j
Cl

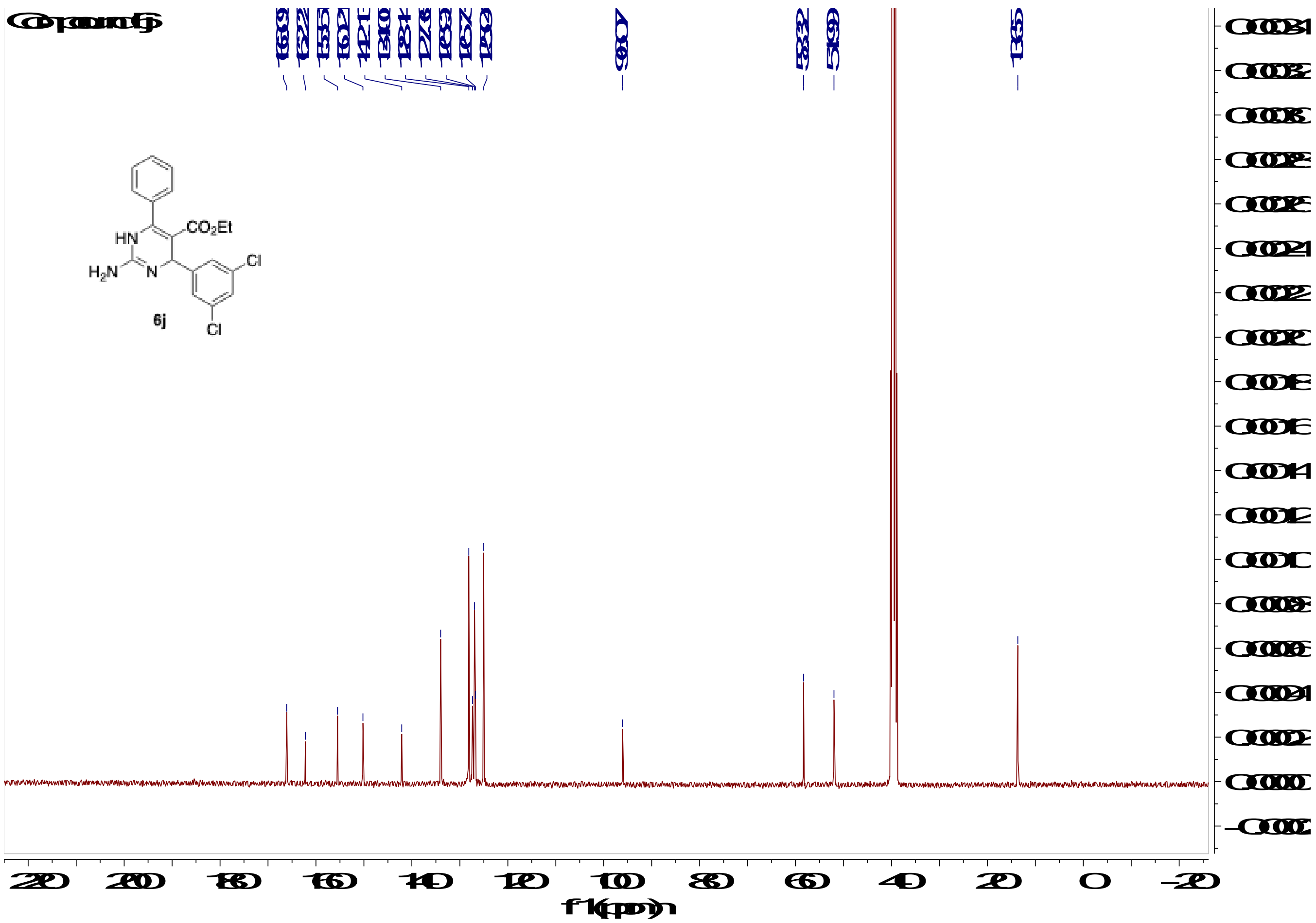
CO2Et
HN
H2N
N
Cl
Cl
6j

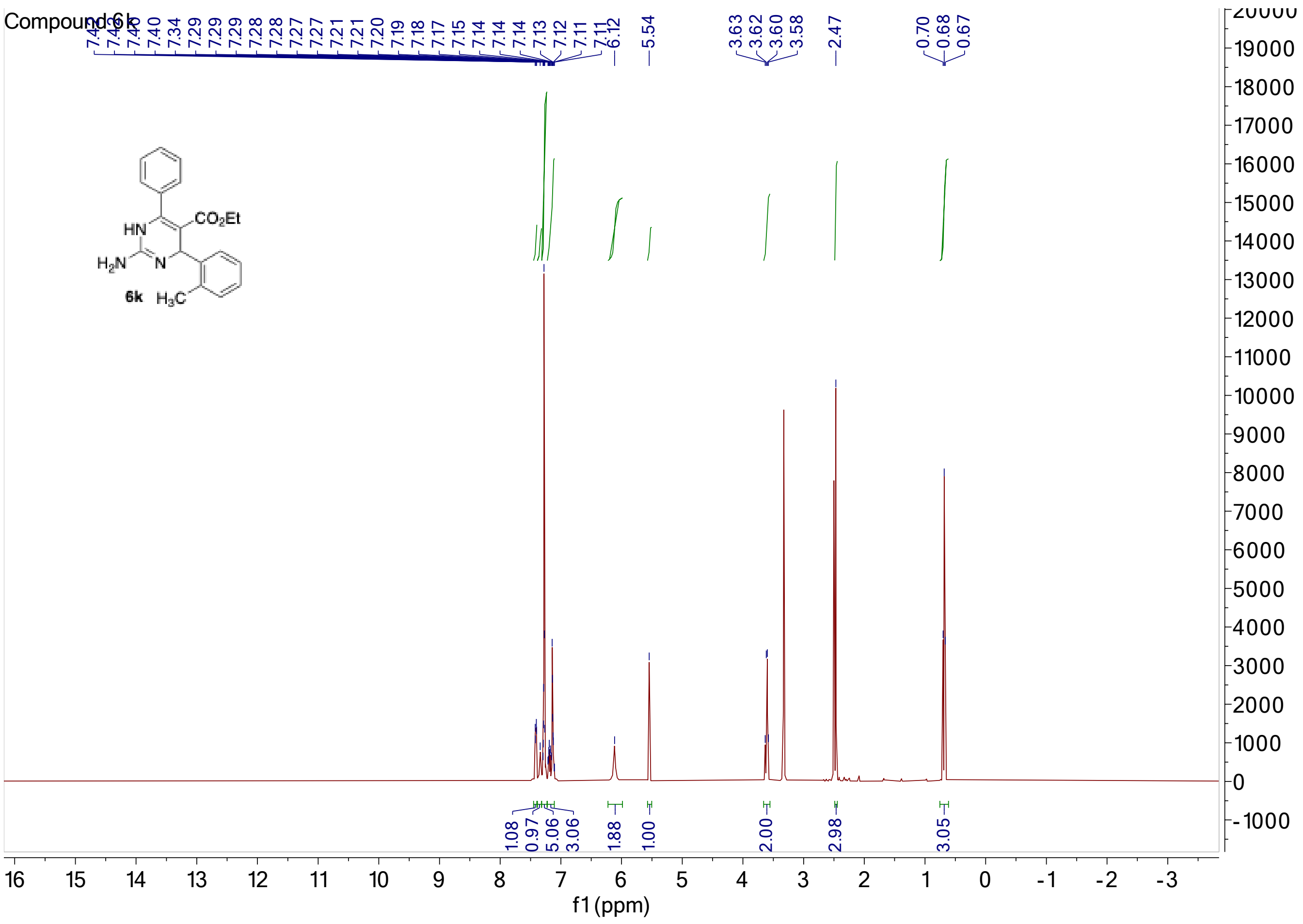

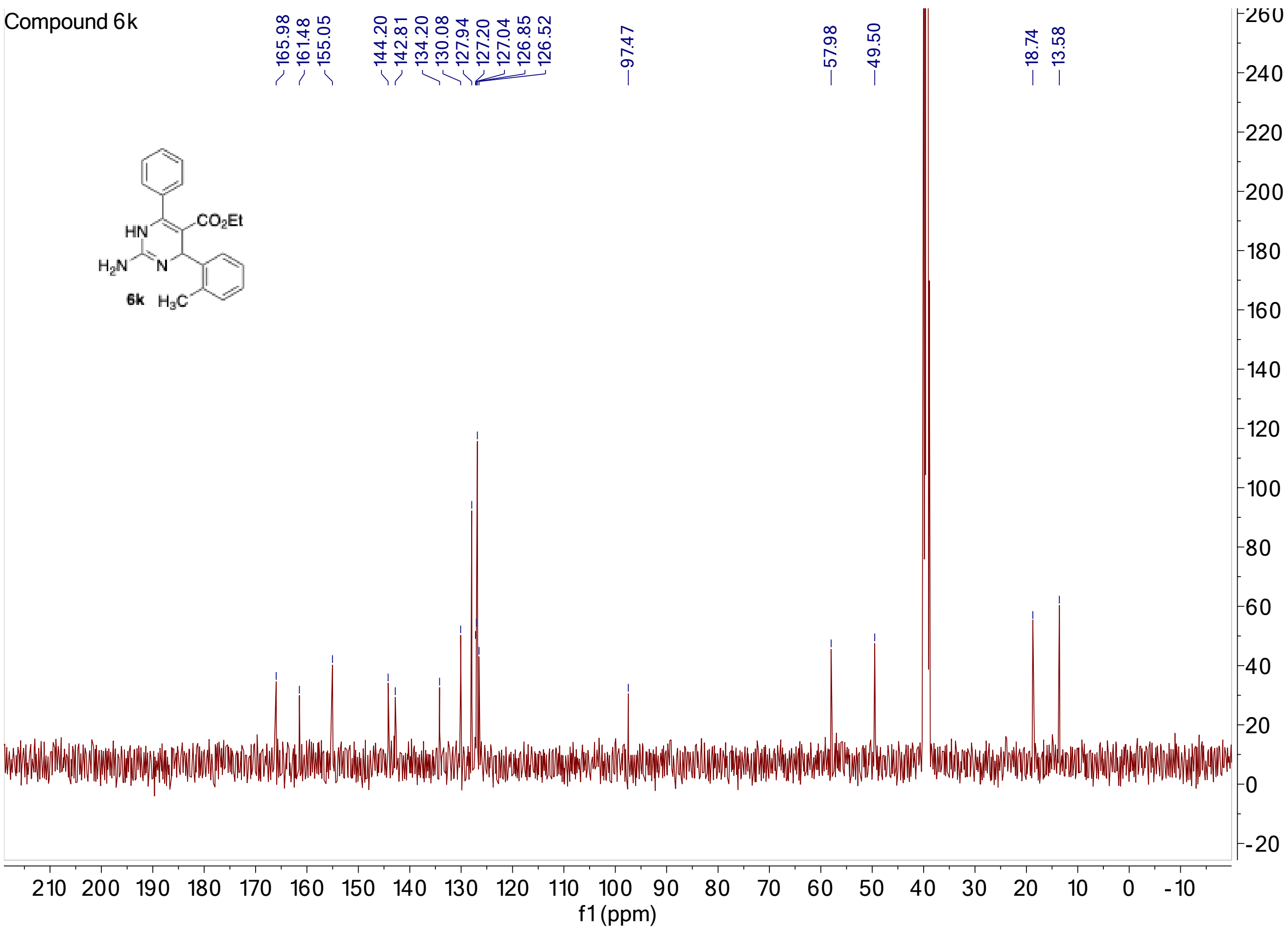
Compound 6k
165.98
161.48
155.05
144.20
142.81
134.20
130.08
127.94
127.20
127.04
126.85
126.52
97.47
57.98
49.50
18.74
13.58
HN
CO_2Et
H_2N
N
6k
H_3C
f1 (ppm)

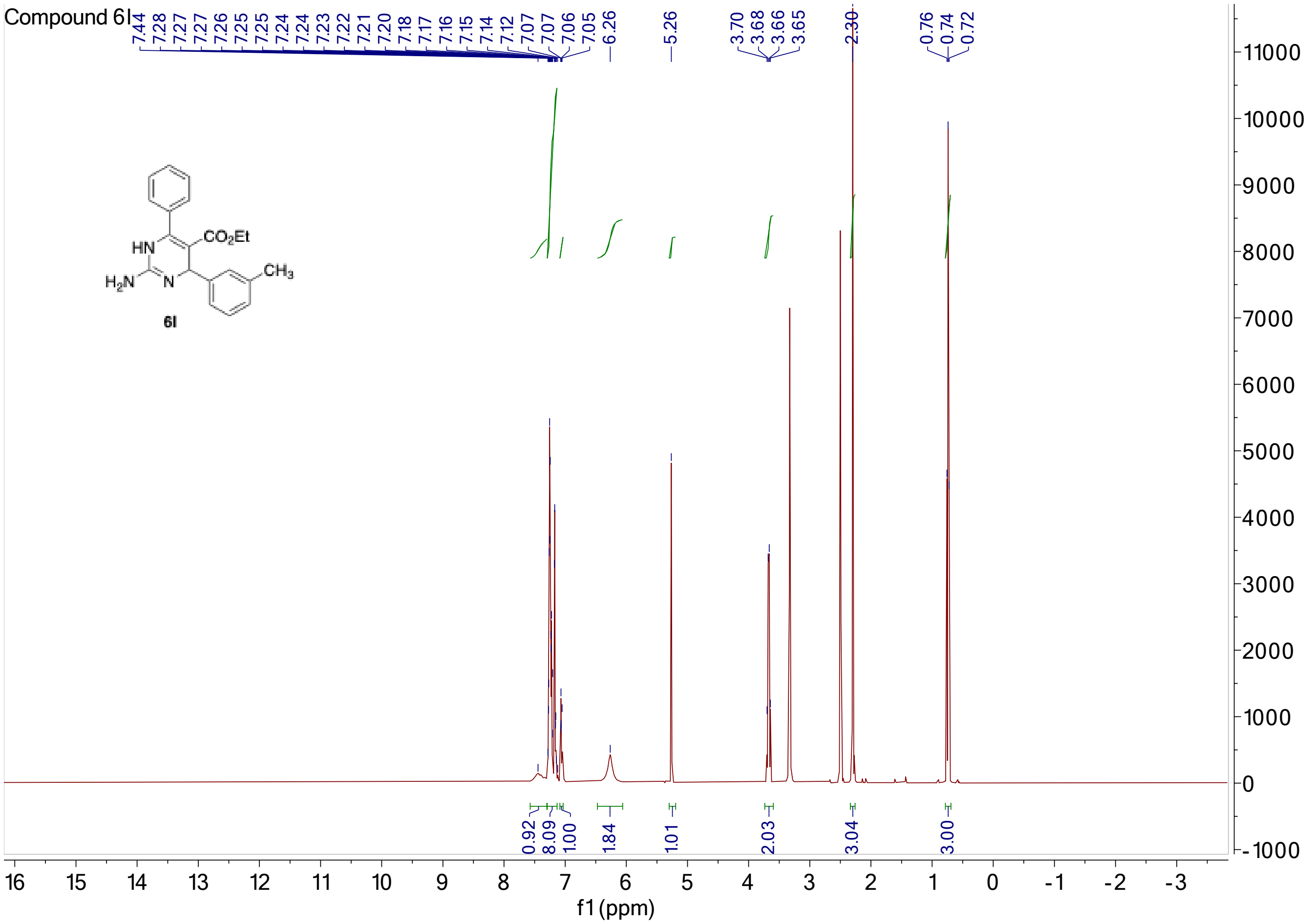
Compound 6l
7.44 7.28 7.27 7.27 7.26 7.25 7.25 7.24 7.24 7.23 7.22 7.21 7.20 7.18 7.17 7.16 7.15 7.14 7.12 7.07 7.07 7.06 7.05
6.26
5.26
3.70 3.68 3.66 3.65
2.30
0.76 0.74 0.72
HN
CO2Et
H2N
N
CH3
6l
0.92 8.09 1.00 1.84 1.01 2.03 3.04 3.00
f1 (ppm)

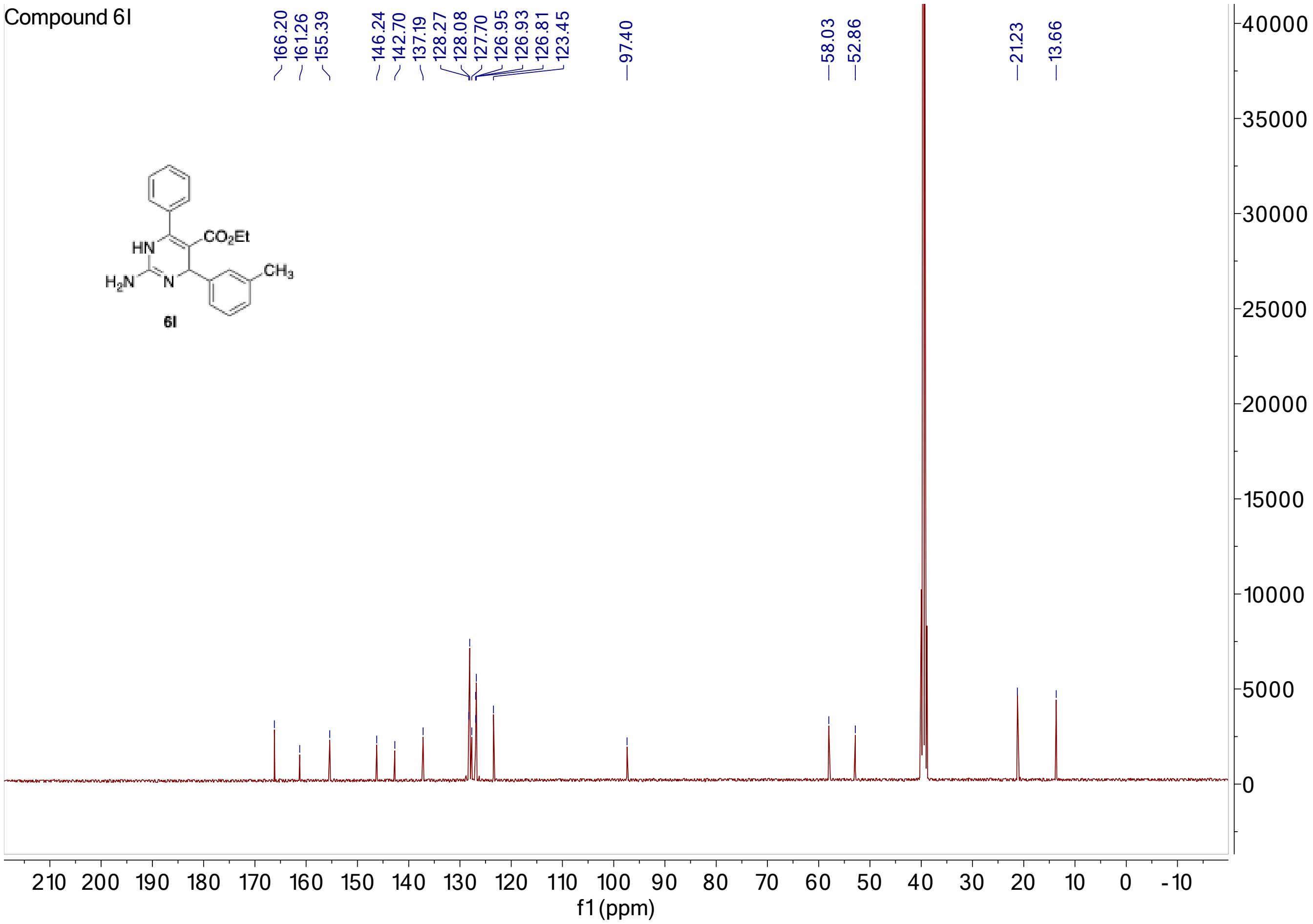
Compound 6l
166.20
161.26
155.39
146.24
142.70
137.19
128.27
128.08
127.70
126.95
126.93
126.81
123.45
97.40
58.03
52.86
21.23
13.66
HN
CO_2Et
H_2N
N
CH_3
6l
40000
35000
30000
25000
20000
15000
10000
5000
0
210 200 190 180 170 160 150 140 130 120 110 100 90 80 70 60 50 40 30 20 10 0 -10
f1 (ppm)

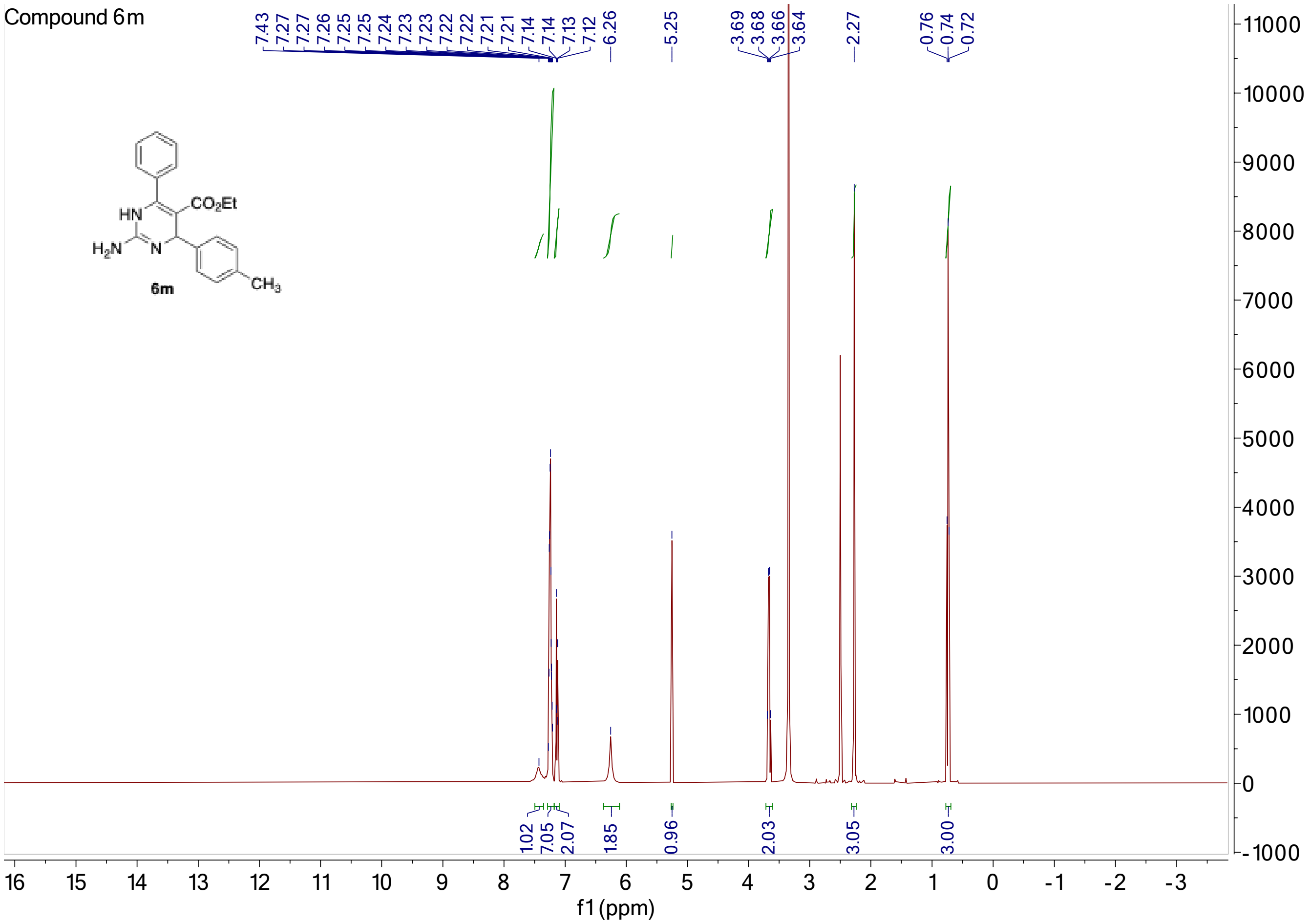
Compound 6m
7.43
7.27
7.27
7.26
7.25
7.25
7.24
7.23
7.23
7.22
7.22
7.21
7.21
7.14
7.14
7.13
7.12
6.26
5.25
3.69
3.68
3.66
3.64
2.27
0.76
0.74
0.72
CO2Et
HN
H2N
N
CH3
6m
1.02
7.05
2.07
1.85
0.96
2.03
3.05
3.00
11000
10000
9000
8000
7000
6000
5000
4000
3000
2000
1000
0
-1000
16
15
14
13
12
11
10
9
8
7
6
5
4
3
2
1
0
-1
-2
-3
f1 (ppm)

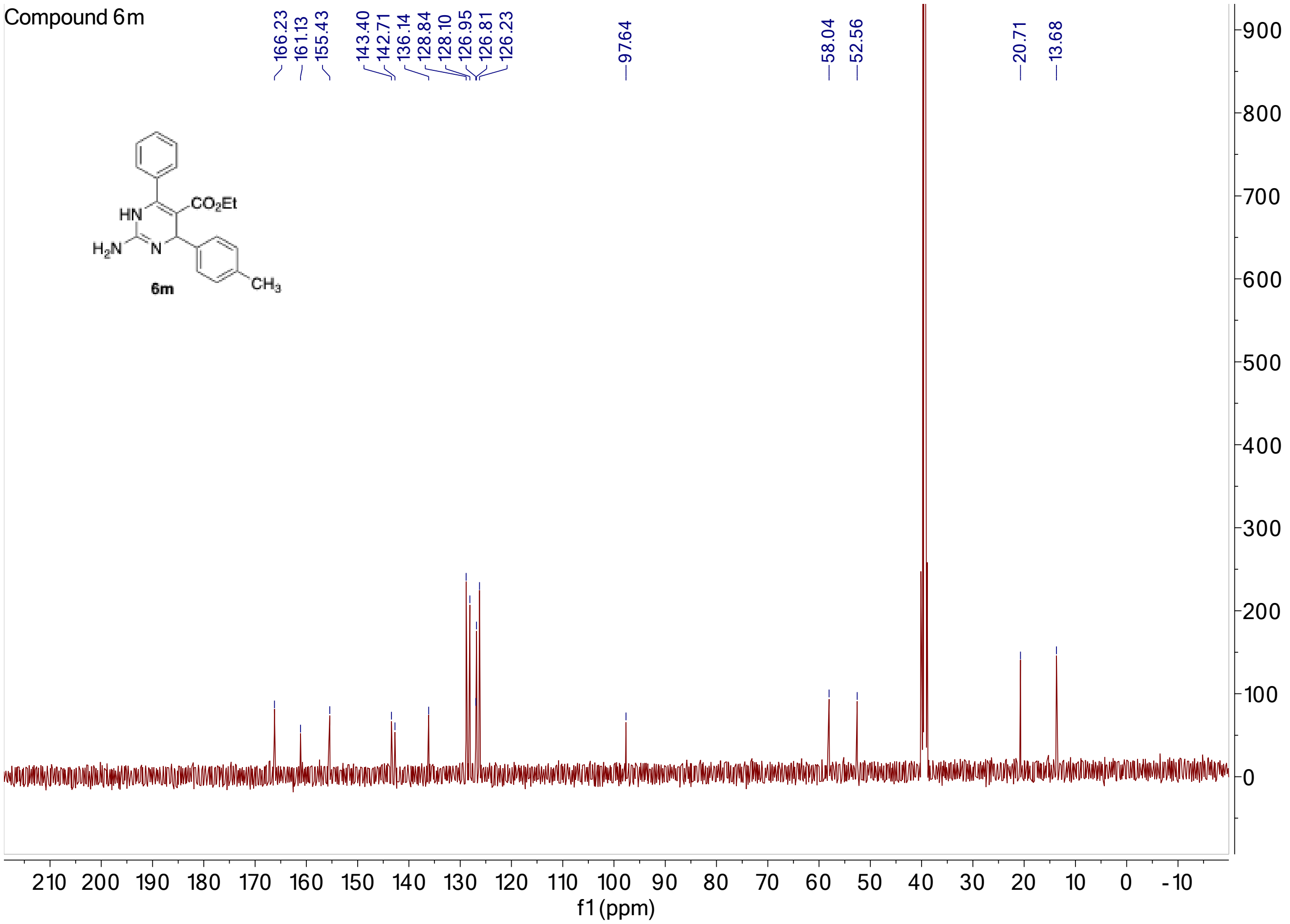

Compound 6m
166.23
161.13
155.43
143.40
142.71
136.14
128.84
128.10
126.95
126.81
126.23
97.64
58.04
52.56
20.71
13.68
HN
CO2Et
H2N
N
CH3
6m
900
800
700
600
500
400
300
200
100
0
210 200 190 180 170 160 150 140 130 120 110 100 90 80 70 60 50 40 30 20 10 0 -10
f1 (ppm)

1590

1595

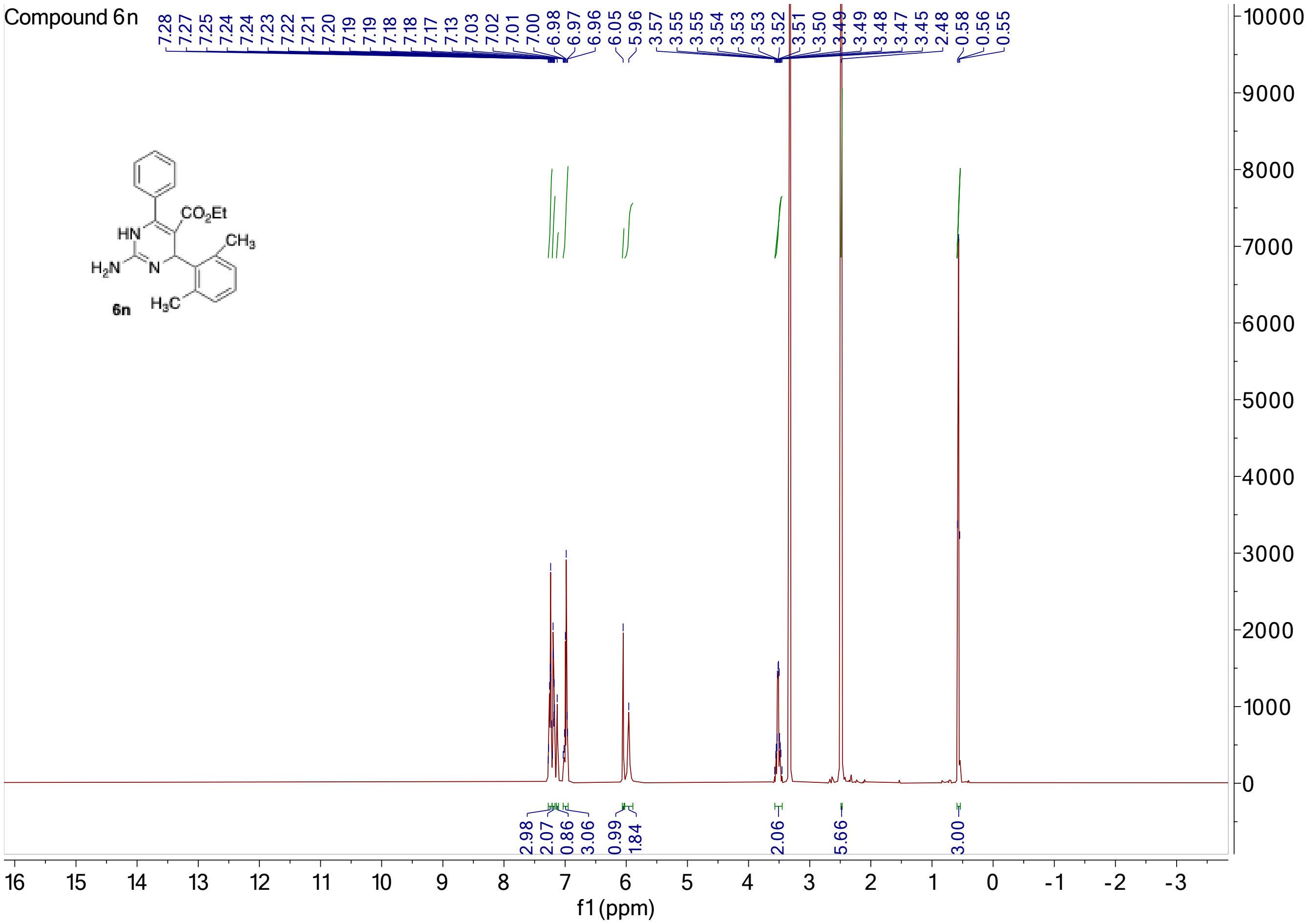
Compound 6n
7.28 7.27 7.25 7.24 7.24 7.23 7.22 7.21 7.20 7.19 7.19 7.18 7.18 7.17 7.13 7.03 7.02 7.01 7.00 6.98 6.97 6.96 6.05 5.96 3.57 3.55 3.55 3.54 3.53 3.53 3.52 3.51 3.50 3.49 3.49 3.48 3.47 3.45 2.48 0.58 0.56 0.55
CO2Et
HN
CH3
H2N
N
6n
H3C
2.98 2.07 0.86 3.06 0.99 1.84 2.06 5.66 3.00
f1 (ppm)

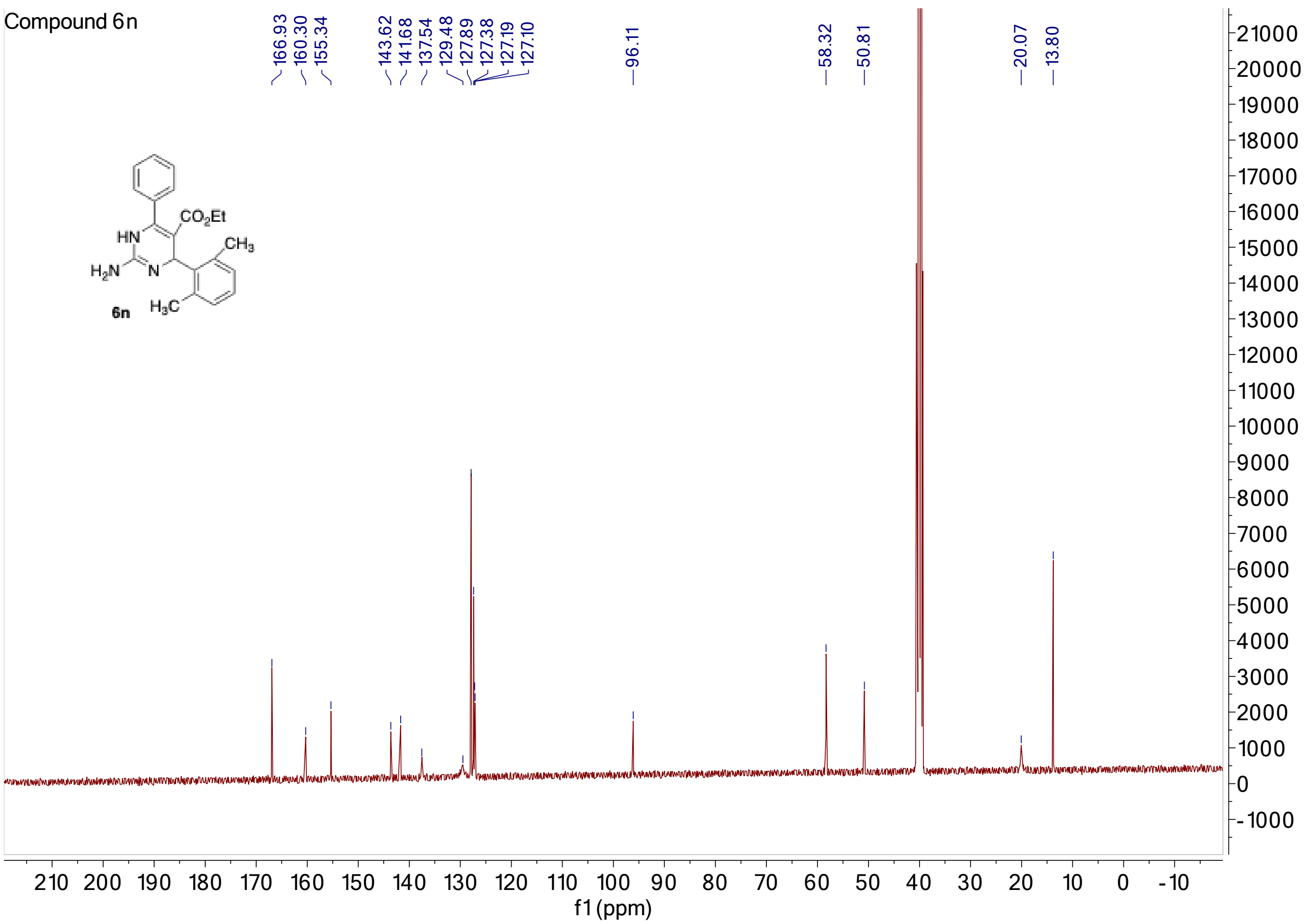

Compound 6n
166.93
160.30
155.34
143.62
141.68
137.54
129.48
127.89
127.38
127.19
127.10
96.11
58.32
50.81
20.07
13.80
CO2Et
HN
CH3
H2N
N
6n
H3C
f1 (ppm)

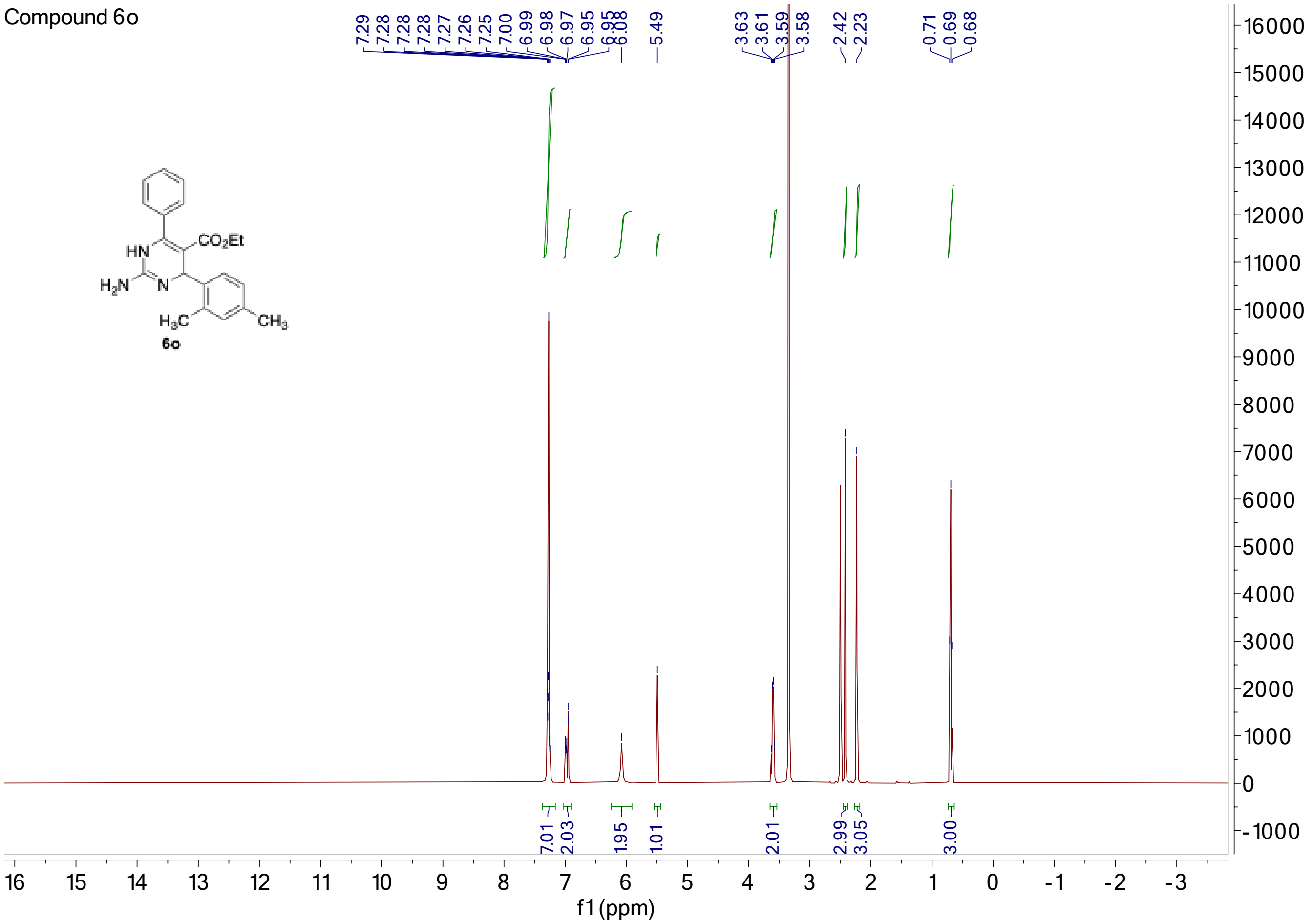
Compound 6o
7.29 7.28 7.28 7.28 7.27 7.26 7.25 7.00 6.99 6.98 6.97 6.95 6.95 6.08
5.49
3.63 3.61 3.59 3.58
2.42 2.23
0.71 0.69 0.68
CO2Et
HN
H2N
N
H3C
CH3
6o
7.01 2.03 1.95 1.01 2.01 2.99 3.05 3.00
f1 (ppm)

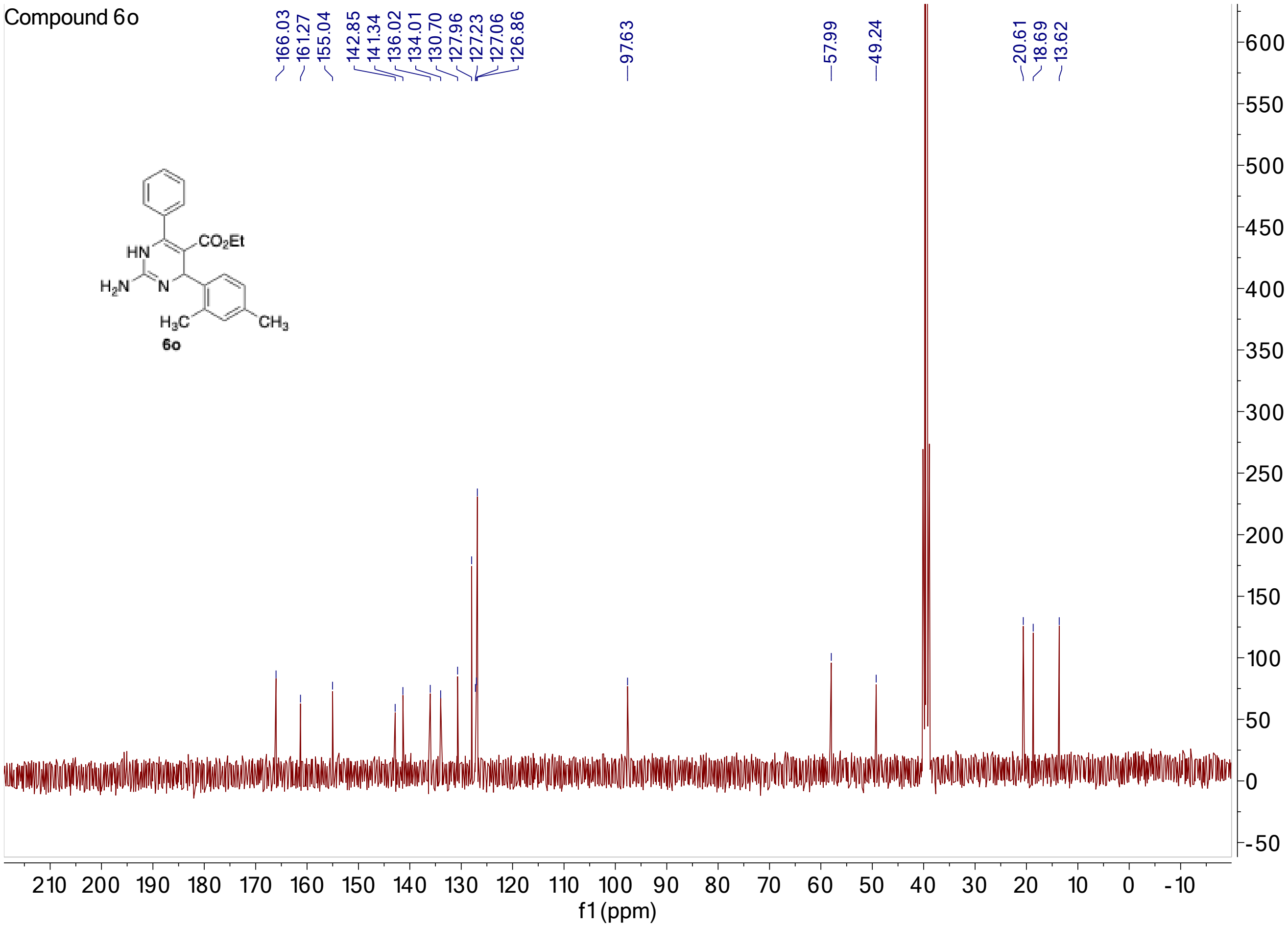

Compound 6o
166.03
161.27
155.04
142.85
141.34
136.02
134.01
130.70
127.96
127.23
127.06
126.86
97.63
57.99
49.24
20.61
18.69
13.62
CO2Et
HN
H2N
N
H3C
CH3
6o
f1 (ppm)

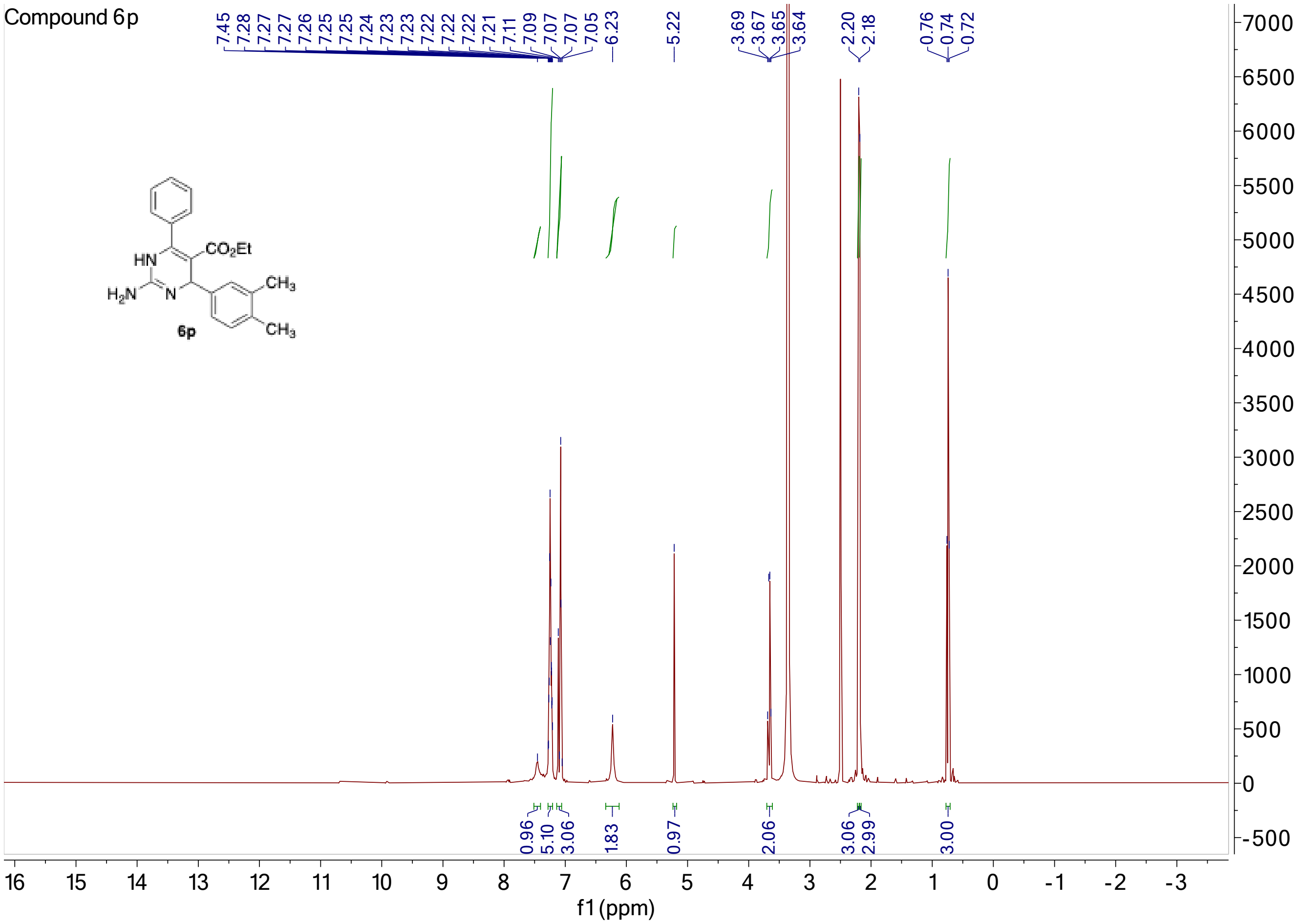
Compound 6p
7.45
7.28
7.27
7.27
7.26
7.25
7.25
7.24
7.23
7.23
7.22
7.22
7.22
7.21
7.11
7.09
7.07
7.07
7.05
6.23
5.22
3.69
3.67
3.65
3.64
2.20
2.18
0.76
0.74
0.72
HN
CO2Et
H2N
N
CH3
CH3
6p
0.96
5.10
3.06
1.83
0.97
2.06
3.06
2.99
3.00
f1 (ppm)

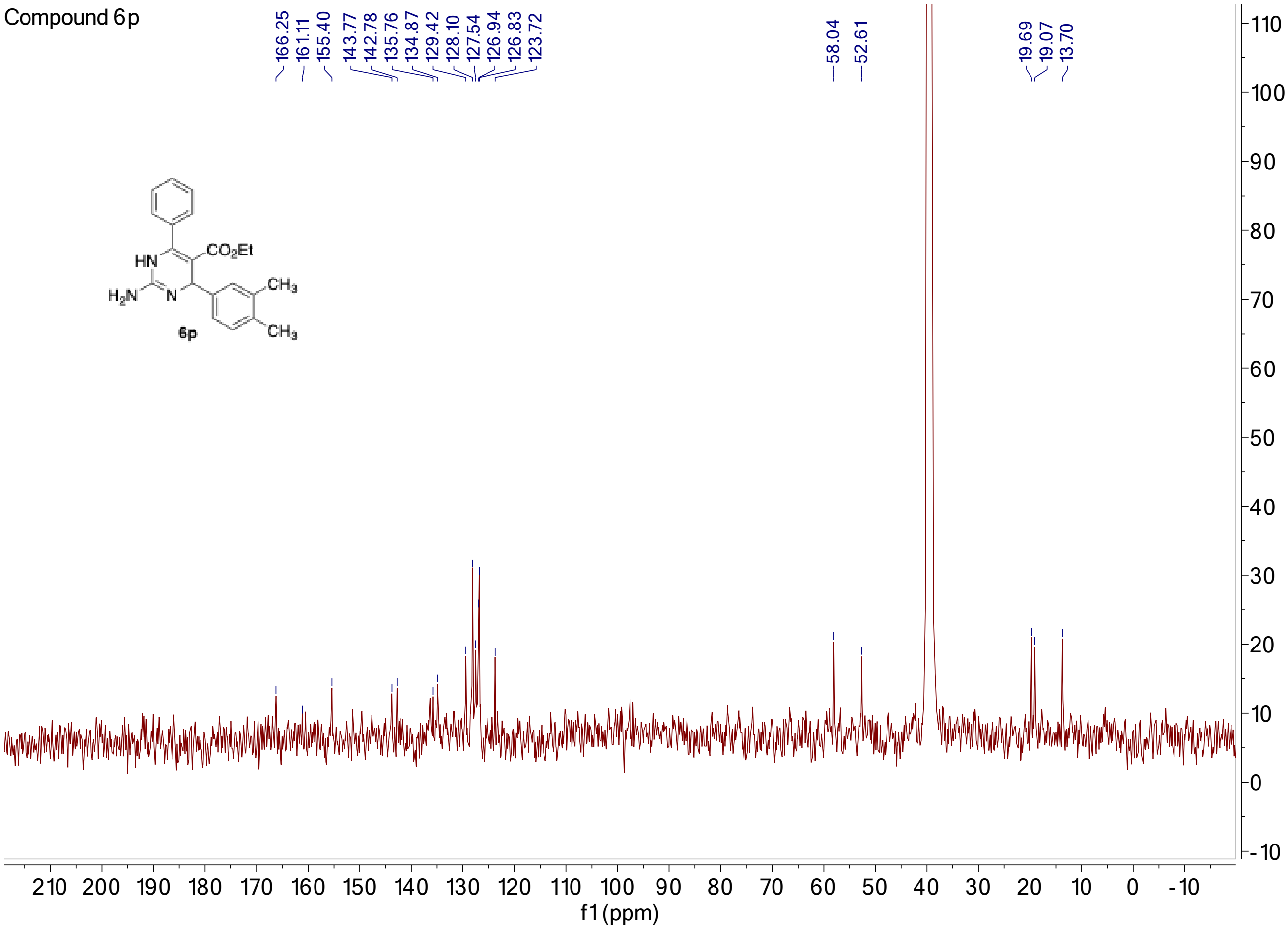
Compound 6p
166.25
161.11
155.40
143.77
142.78
135.76
134.87
129.42
128.10
127.54
126.94
126.83
123.72
58.04
52.61
19.69
19.07
13.70
CO2Et
HN
H2N
N
CH3
CH3
6p
f1 (ppm)

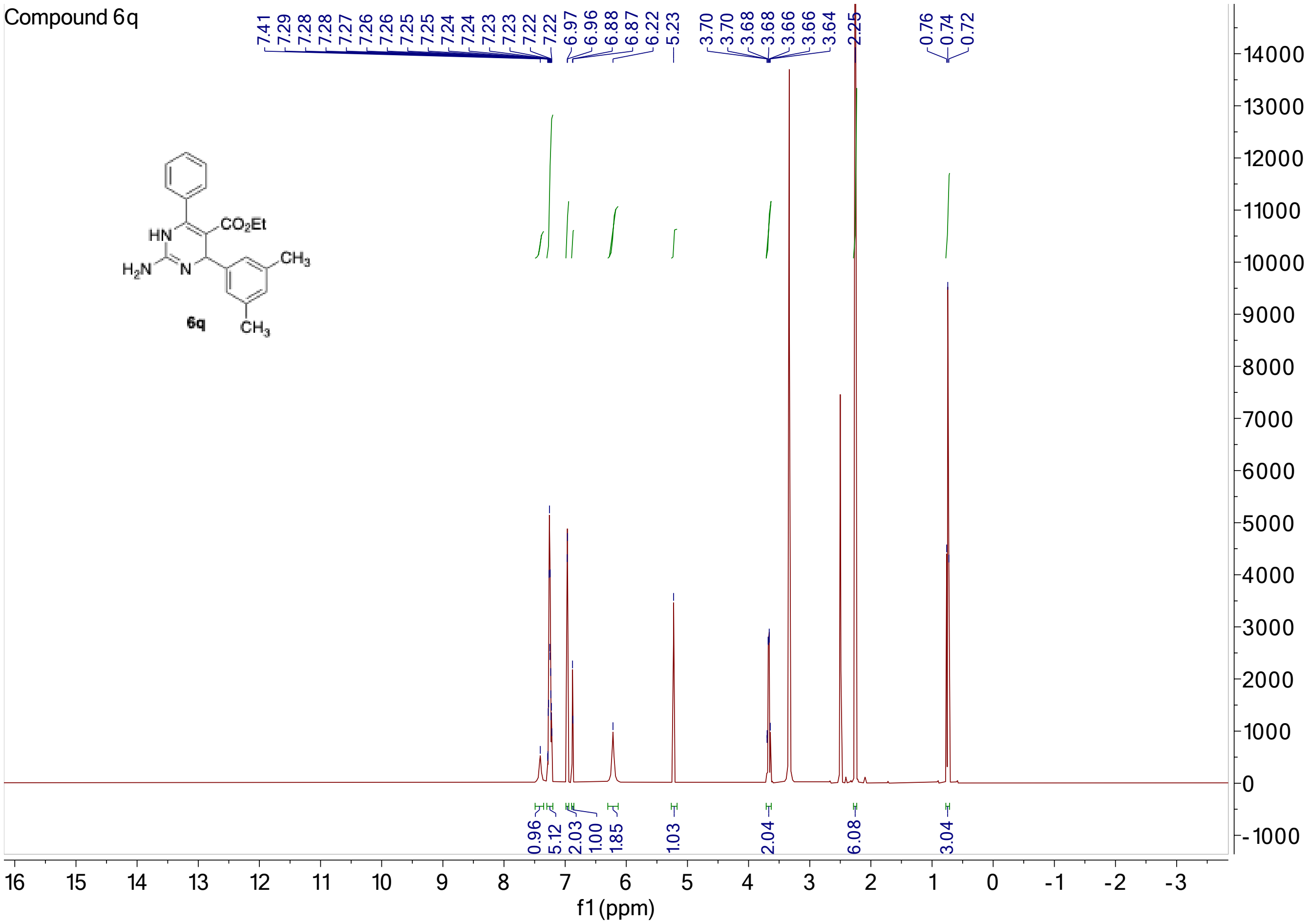
Compound 6q
7.41
7.29
7.28
7.28
7.27
7.26
7.25
7.25
7.24
7.24
7.23
7.23
7.22
7.22
6.97
6.96
6.88
6.87
6.22
5.23
3.70
3.70
3.68
3.68
3.66
3.66
3.64
2.25
0.76
0.74
0.72
CO2Et
HN
H2N
N
CH3
CH3
6q
0.96
5.12
2.03
1.00
1.85
1.03
2.04
6.08
3.04
f1 (ppm)

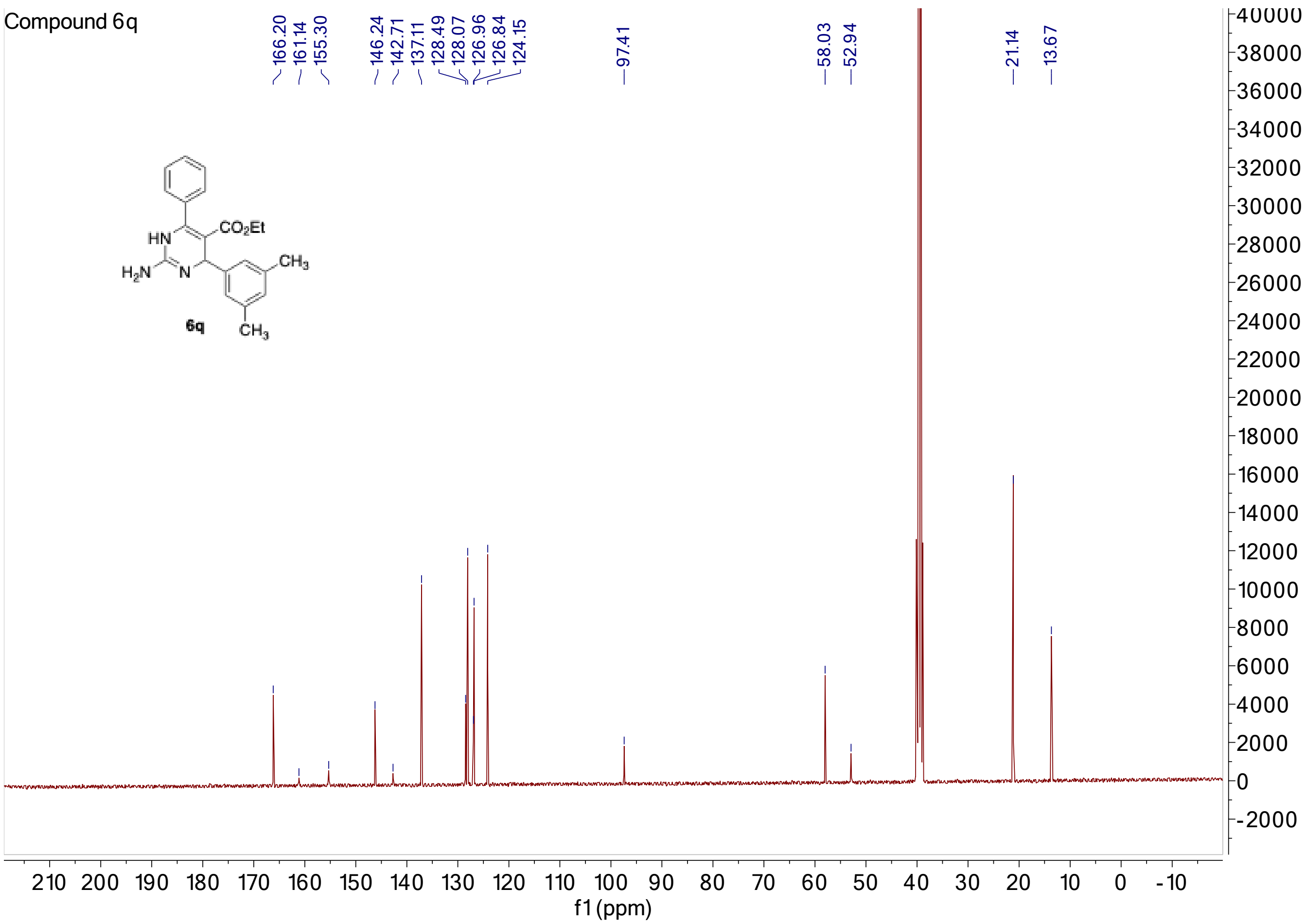
Compound 6q
166.20
161.14
155.30
146.24
142.71
137.11
128.49
128.07
126.96
126.84
124.15
97.41
58.03
52.94
21.14
13.67
HN
CO_2Et
H_2N
N
CH_3
6q
CH_3
f1 (ppm)

Compound 6r

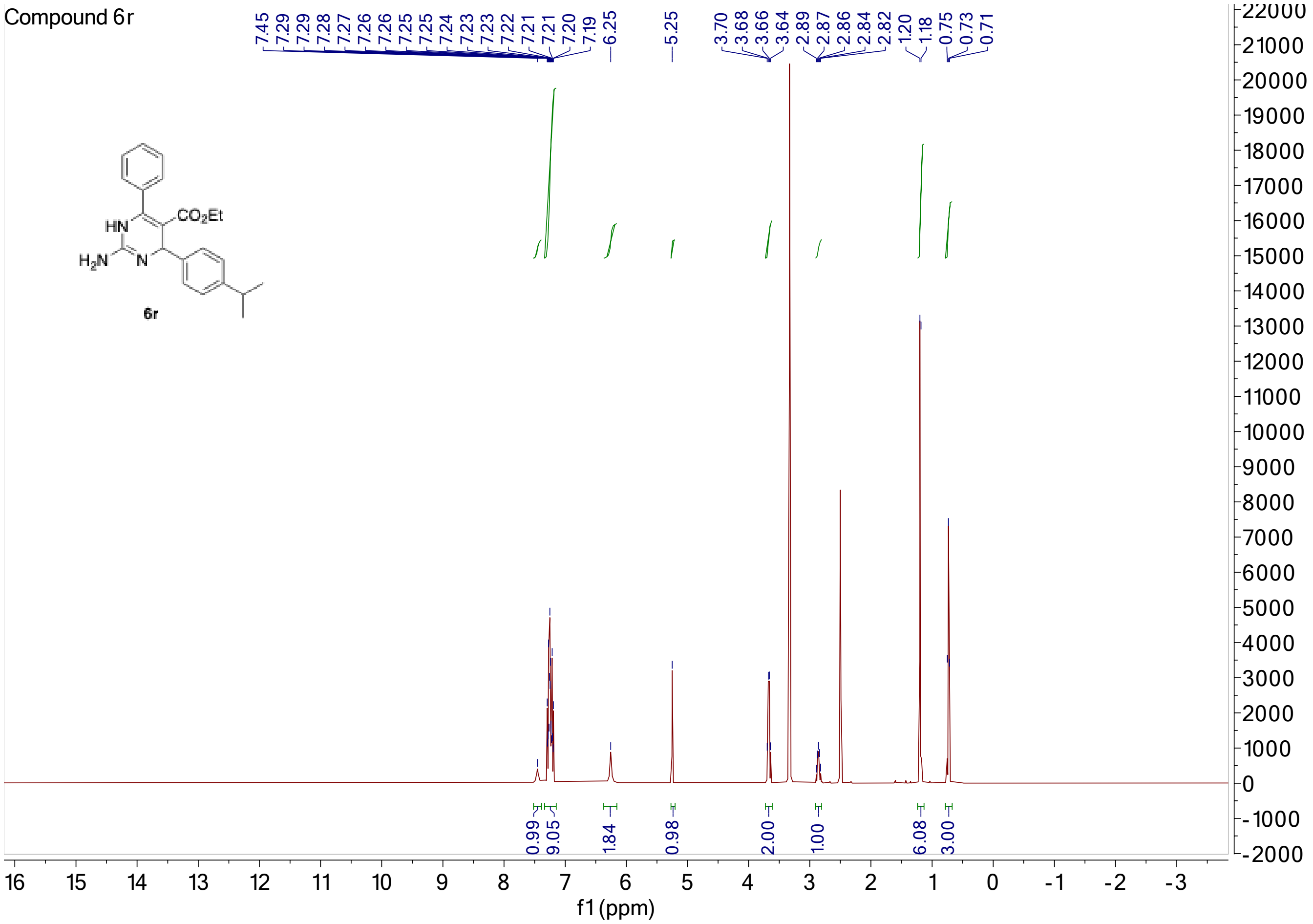

7.45
7.29
7.29
7.28
7.27
7.26
7.26
7.25
7.25
7.24
7.23
7.23
7.22
7.21
7.21
7.20
7.19
6.25
5.25
3.70
3.68
3.66
3.64
2.89
2.87
2.86
2.84
2.82
1.20
1.18
0.75
0.73
0.71
CO2Et
HN
H2N
N
6r
0.99
9.05
1.84
0.98
2.00
1.00
6.08
3.00
f1 (ppm)

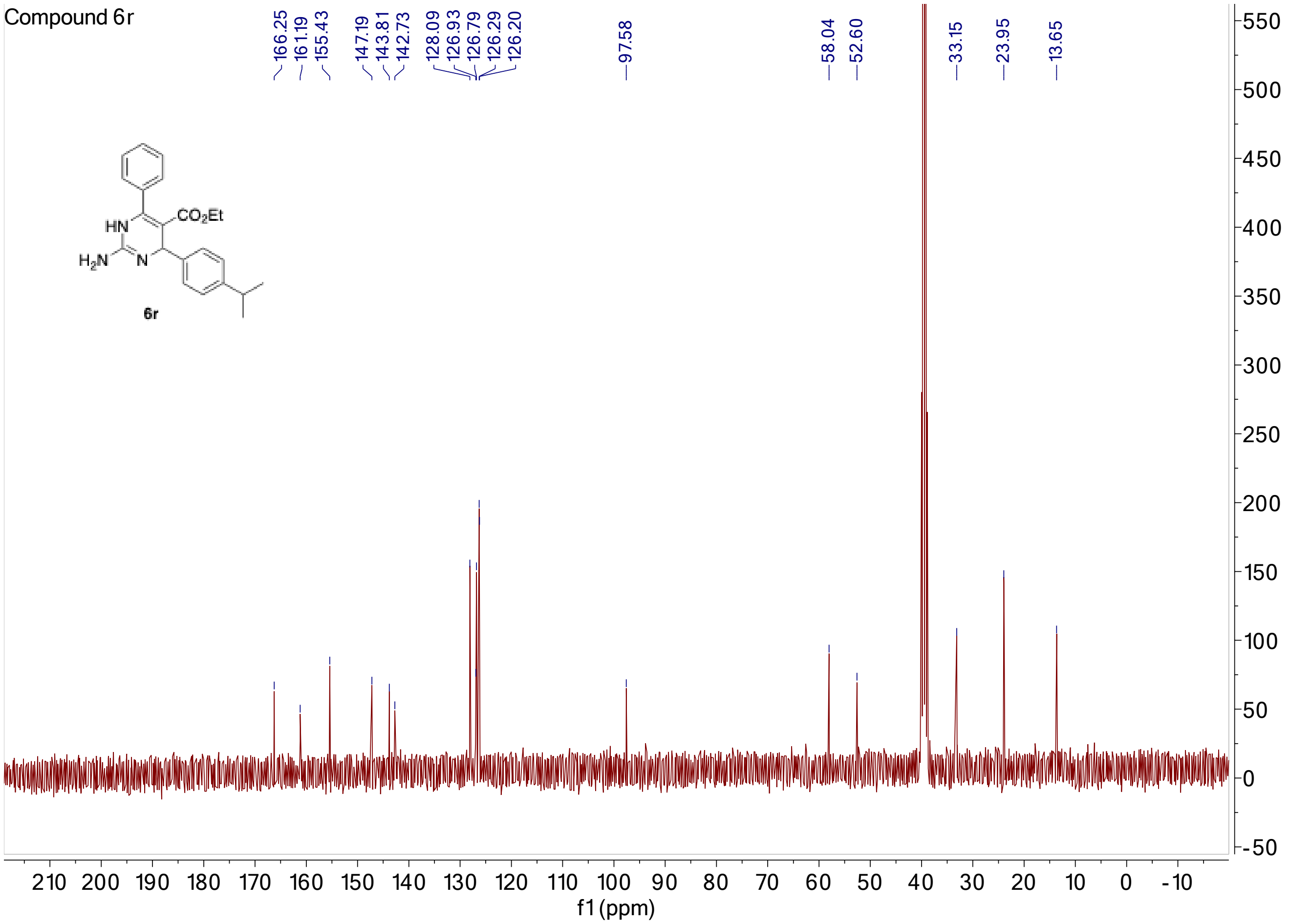
Compound 6r
166.25
161.19
155.43
147.19
143.81
142.73
128.09
126.93
126.79
126.29
126.20
97.58
58.04
52.60
33.15
23.95
13.65
CO2Et
HN
H2N
N
6r
550
500
450
400
350
300
250
200
150
100
50
0
-50
210 200 190 180 170 160 150 140 130 120 110 100 90 80 70 60 50 40 30 20 10 0 -10
f1 (ppm)

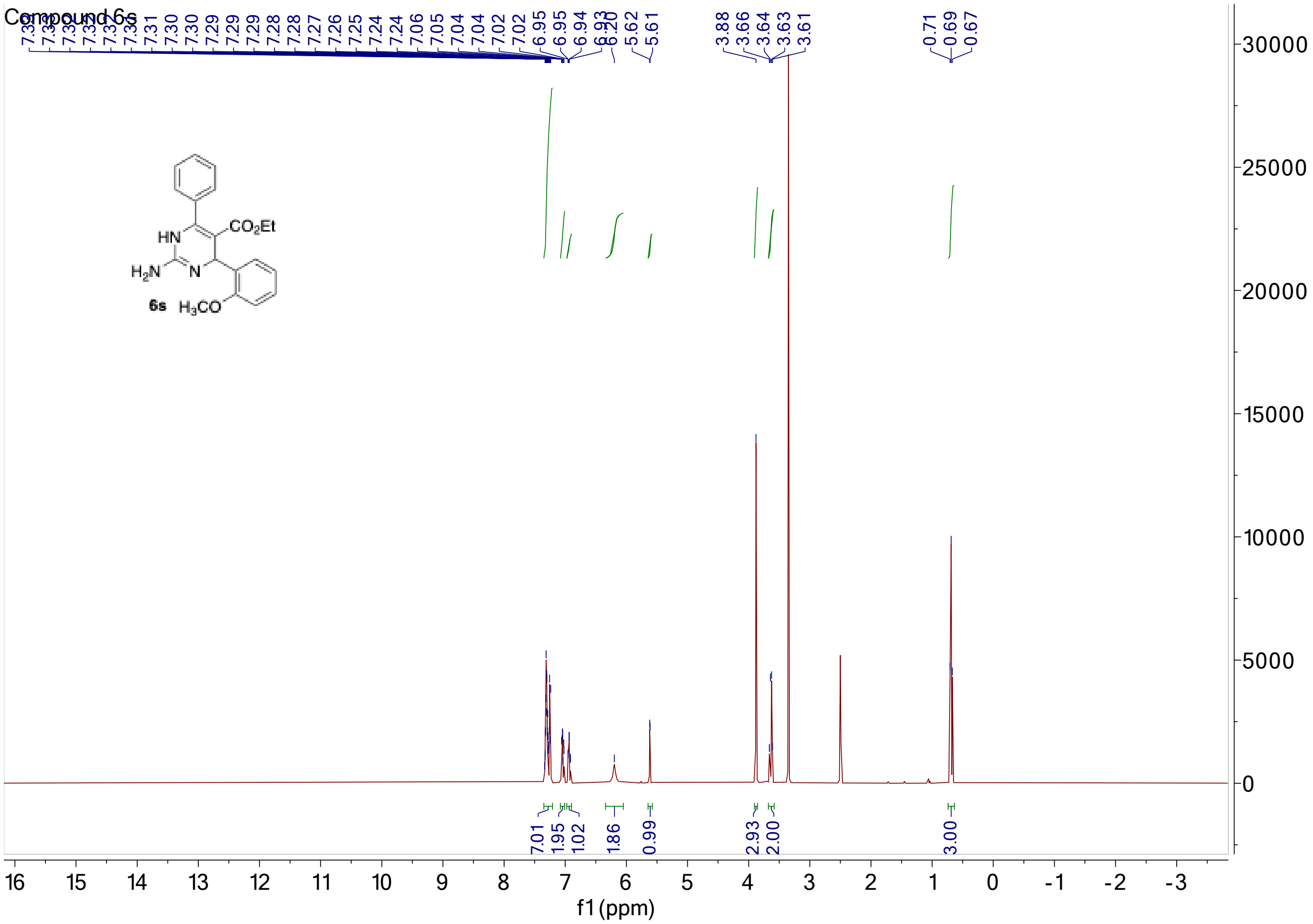
Compound 6s
HN
CO2Et
H2N
N
6s
H3CO
7.01
1.95
1.02
1.86
0.99
2.93
2.00
3.00
f1 (ppm)

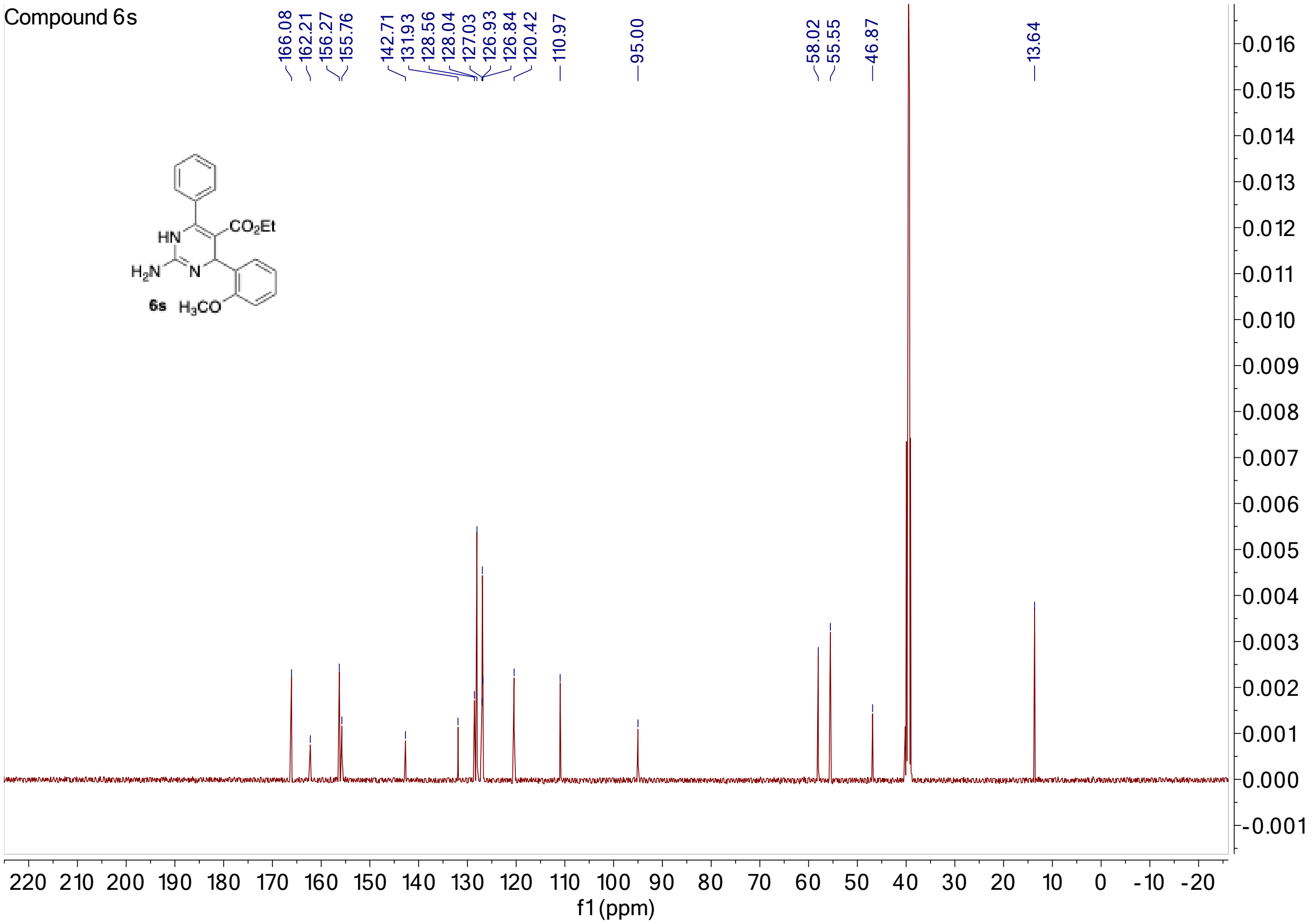
Compound 6s
166.08
162.21
156.27
155.76
142.71
131.93
128.56
128.04
127.03
126.93
126.84
120.42
110.97
95.00
58.02
55.55
46.87
13.64
CO2Et
HN
H2N
N
6s
H3CO
f1 (ppm)

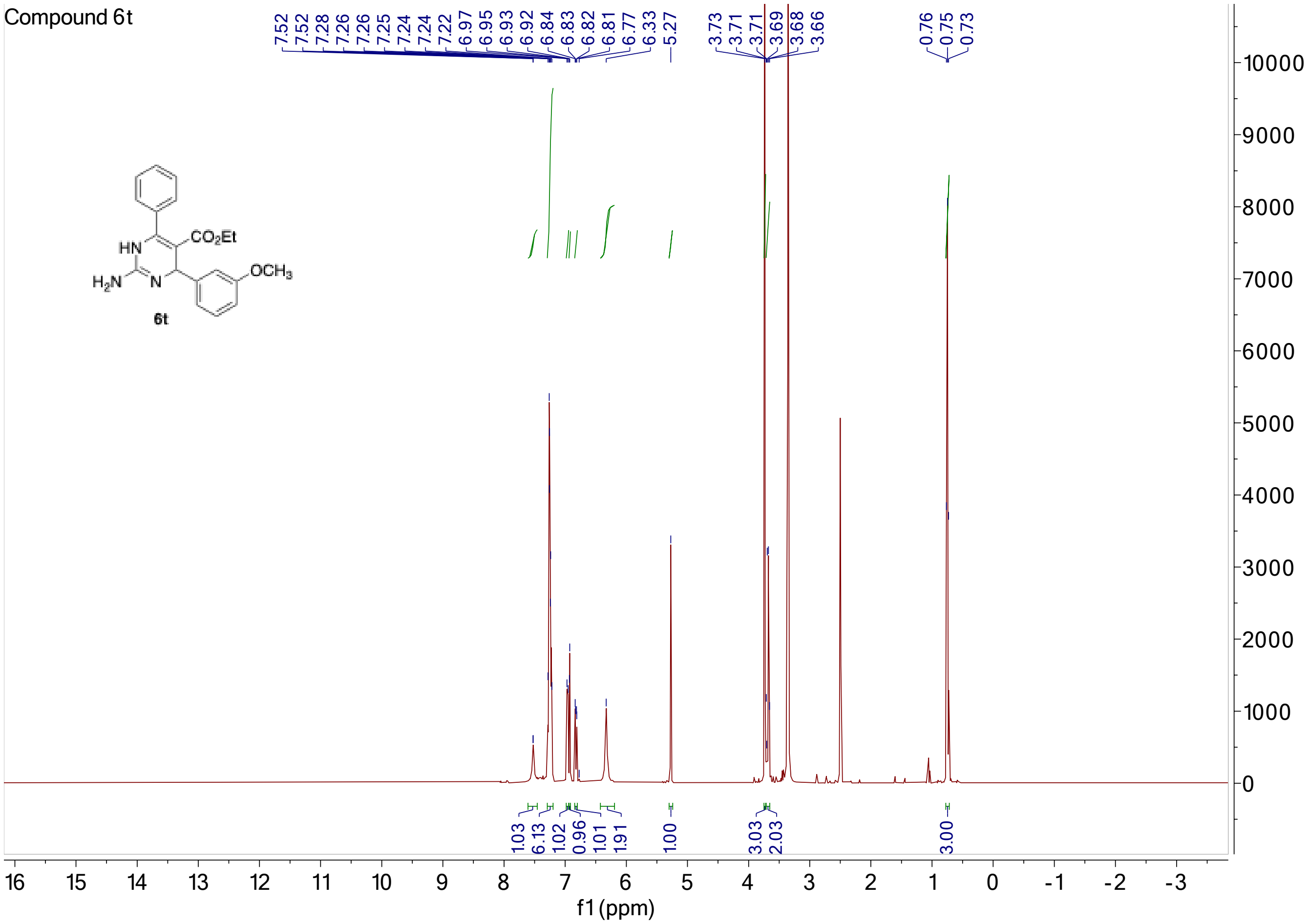
Compound 6t
7.52
7.52
7.28
7.26
7.26
7.25
7.24
7.24
7.22
6.97
6.95
6.93
6.92
6.84
6.83
6.82
6.81
6.77
6.33
5.27
3.73
3.71
3.71
3.69
3.68
3.66
0.76
0.75
0.73
CO2Et
HN
H2N
N
OCH3
6t
1.03
6.13
1.02
0.96
1.01
1.91
1.00
3.03
2.03
3.00
f1 (ppm)

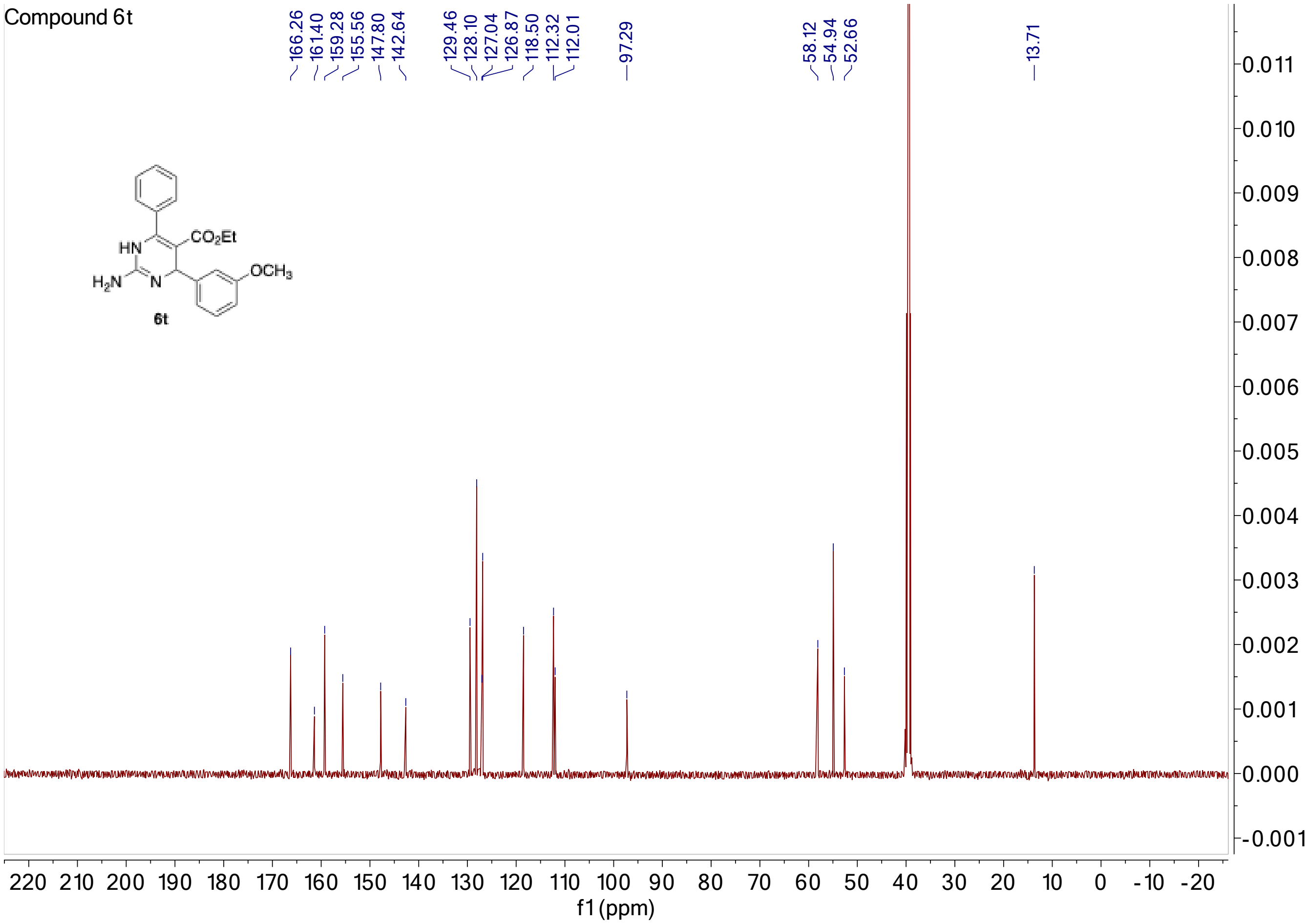
Compound 6t
166.26
161.40
159.28
155.56
147.80
142.64
129.46
128.10
127.04
126.87
118.50
112.32
112.01
97.29
58.12
54.94
52.66
13.71
CO2Et
HN
H2N
N
OCH3
6t
0.011
0.010
0.009
0.008
0.007
0.006
0.005
0.004
0.003
0.002
0.001
0.000
-0.001
220 210 200 190 180 170 160 150 140 130 120 110 100 90 80 70 60 50 40 30 20 10 0 -10 -20
f1 (ppm)

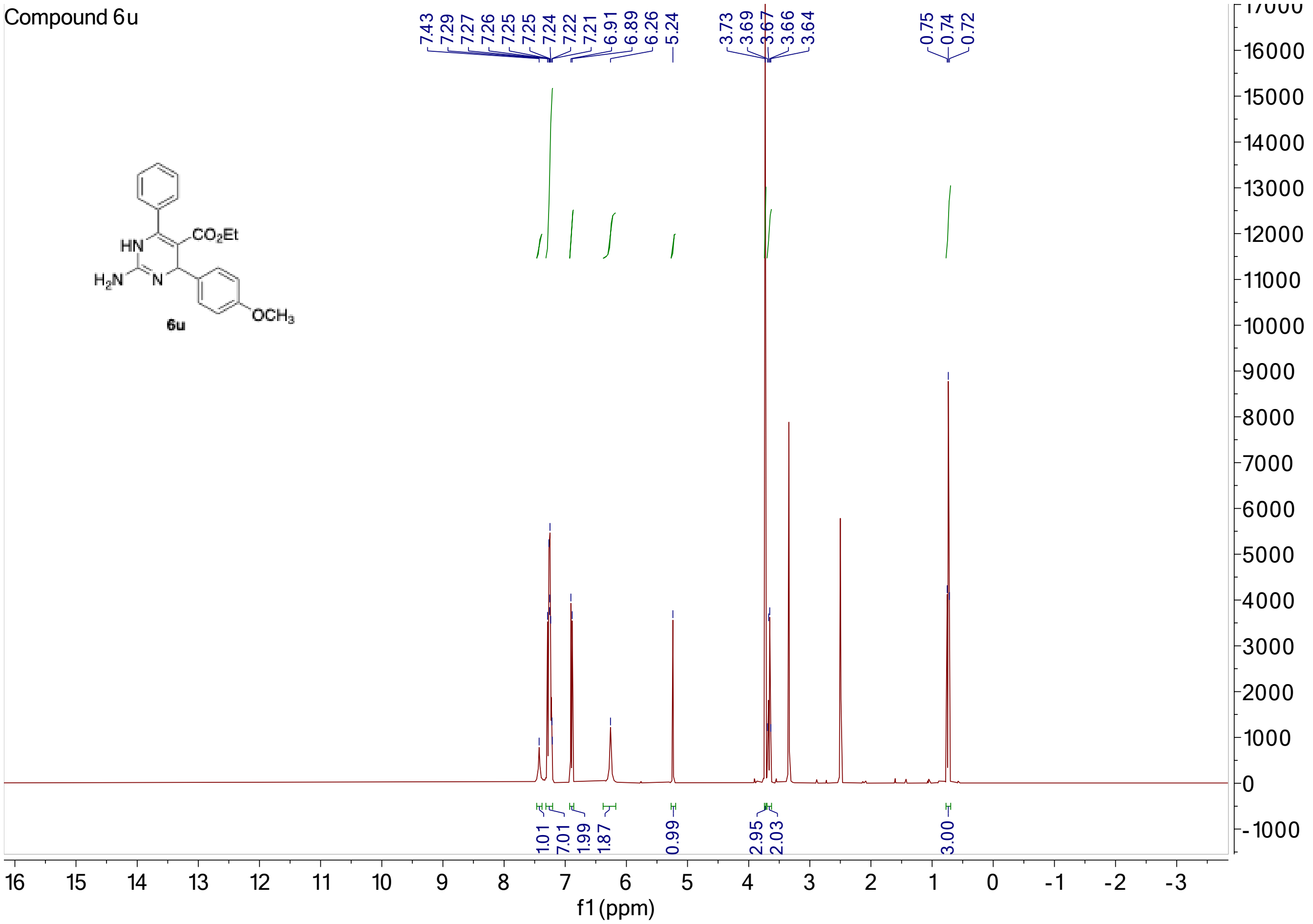
Compound 6u
7.43 7.29 7.27 7.26 7.25 7.25 7.24 7.22 7.21 6.91 6.89 6.26 5.24
3.73 3.69 3.67 3.66 3.64
0.75 0.74 0.72
HN
CO2Et
H2N
N
OCH3
6u
1.01 7.01 1.99 1.87 0.99 2.95 2.03 3.00
f1 (ppm)

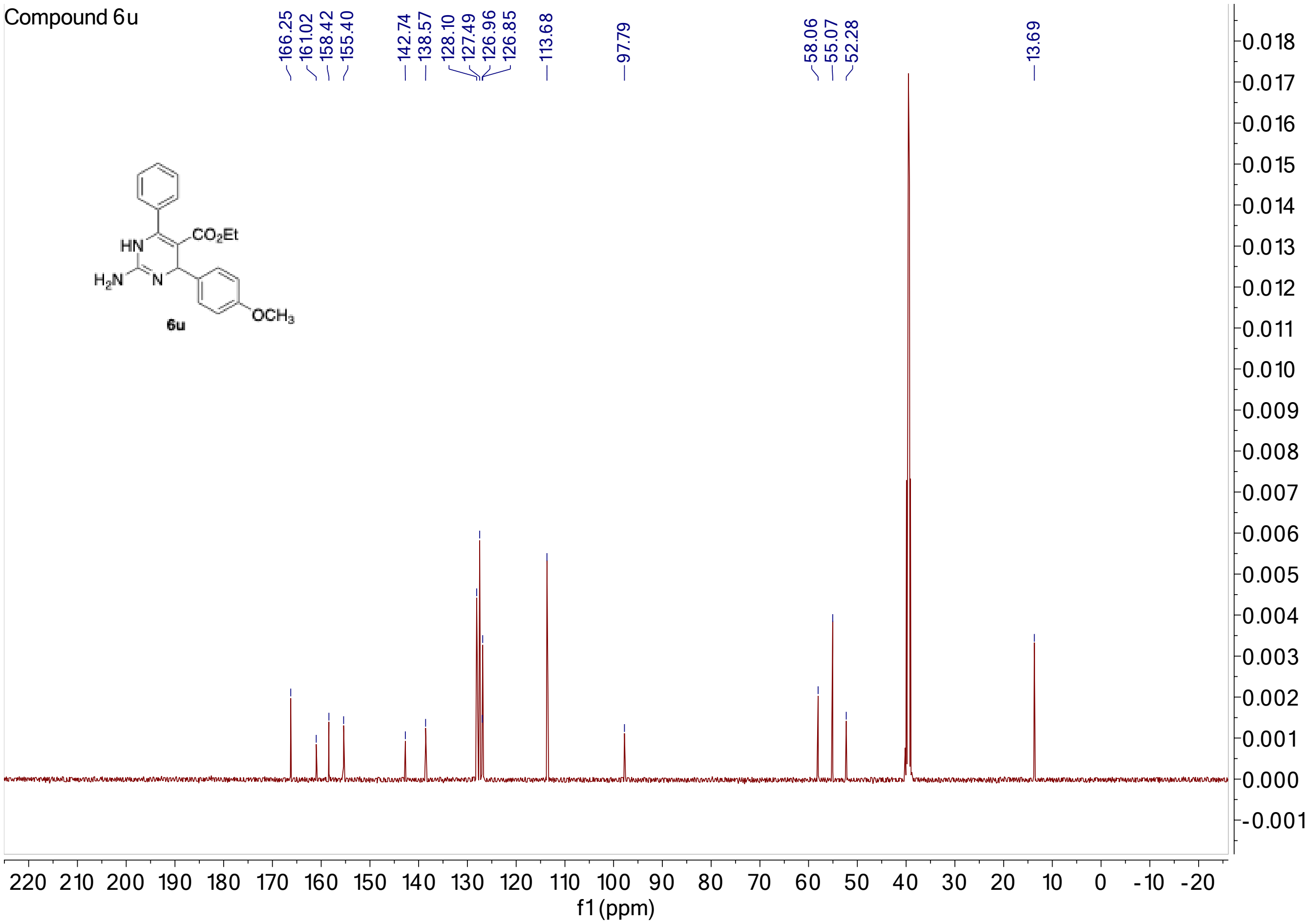
Compound 6u
166.25
161.02
158.42
155.40
142.74
138.57
128.10
127.49
126.96
126.85
113.68
97.79
58.06
55.07
52.28
13.69
HN
H2N
N
CO2Et
OCH3
6u
0.018
0.017
0.016
0.015
0.014
0.013
0.012
0.011
0.010
0.009
0.008
0.007
0.006
0.005
0.004
0.003
0.002
0.001
0.000
-0.001
220 210 200 190 180 170 160 150 140 130 120 110 100 90 80 70 60 50 40 30 20 10 0 -10 -20
f1 (ppm)

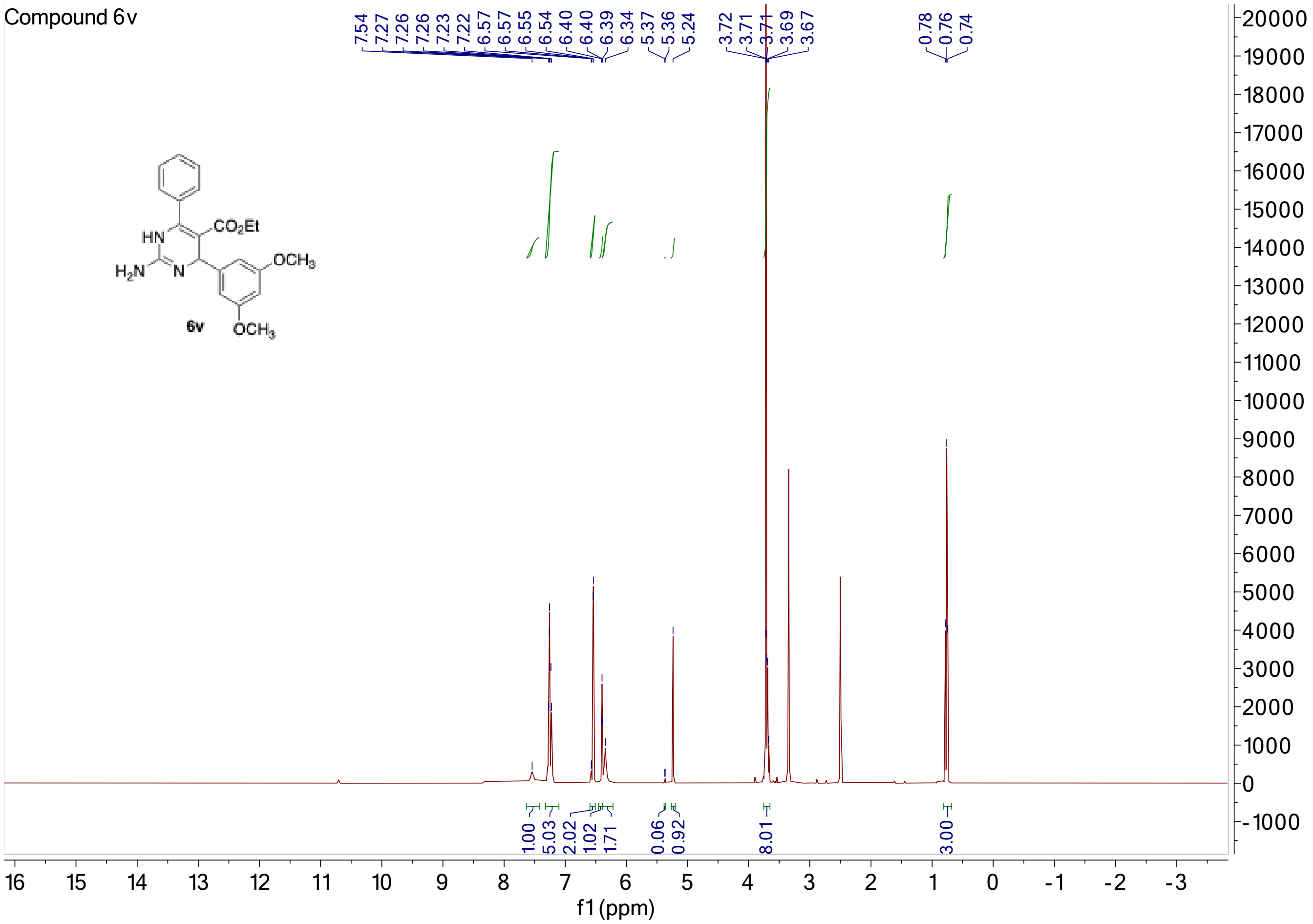
Compound 6v
7.54
7.27
7.26
7.26
7.23
7.22
6.57
6.57
6.55
6.54
6.40
6.40
6.39
6.34
5.37
5.36
5.24
3.72
3.71
3.71
3.69
3.67
0.78
0.76
0.74
CO2Et
HN
H2N
N
OCH3
6v
OCH3
1.00
5.03
2.02
1.02
1.71
0.06
0.92
8.01
3.00
f1 (ppm)

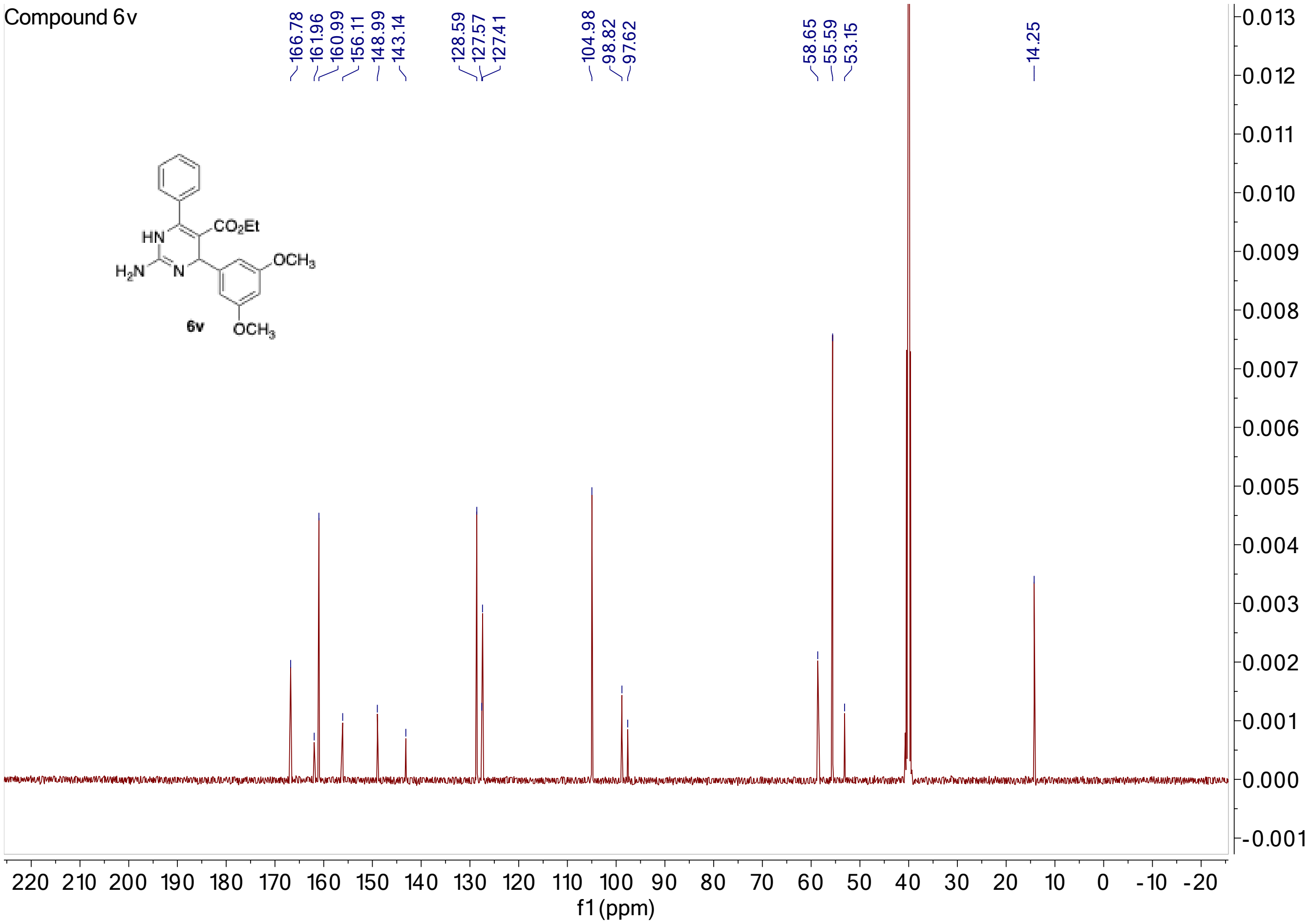
Compound 6v
166.78
161.96
160.99
156.11
148.99
143.14
128.59
127.57
127.41
104.98
98.82
97.62
58.65
55.59
53.15
14.25
HN
CO2Et
H2N
N
OCH3
6v
OCH3
0.013
0.012
0.011
0.010
0.009
0.008
0.007
0.006
0.005
0.004
0.003
0.002
0.001
0.000
-0.001
220 210 200 190 180 170 160 150 140 130 120 110 100 90 80 70 60 50 40 30 20 10 0 -10 -20
f1 (ppm)

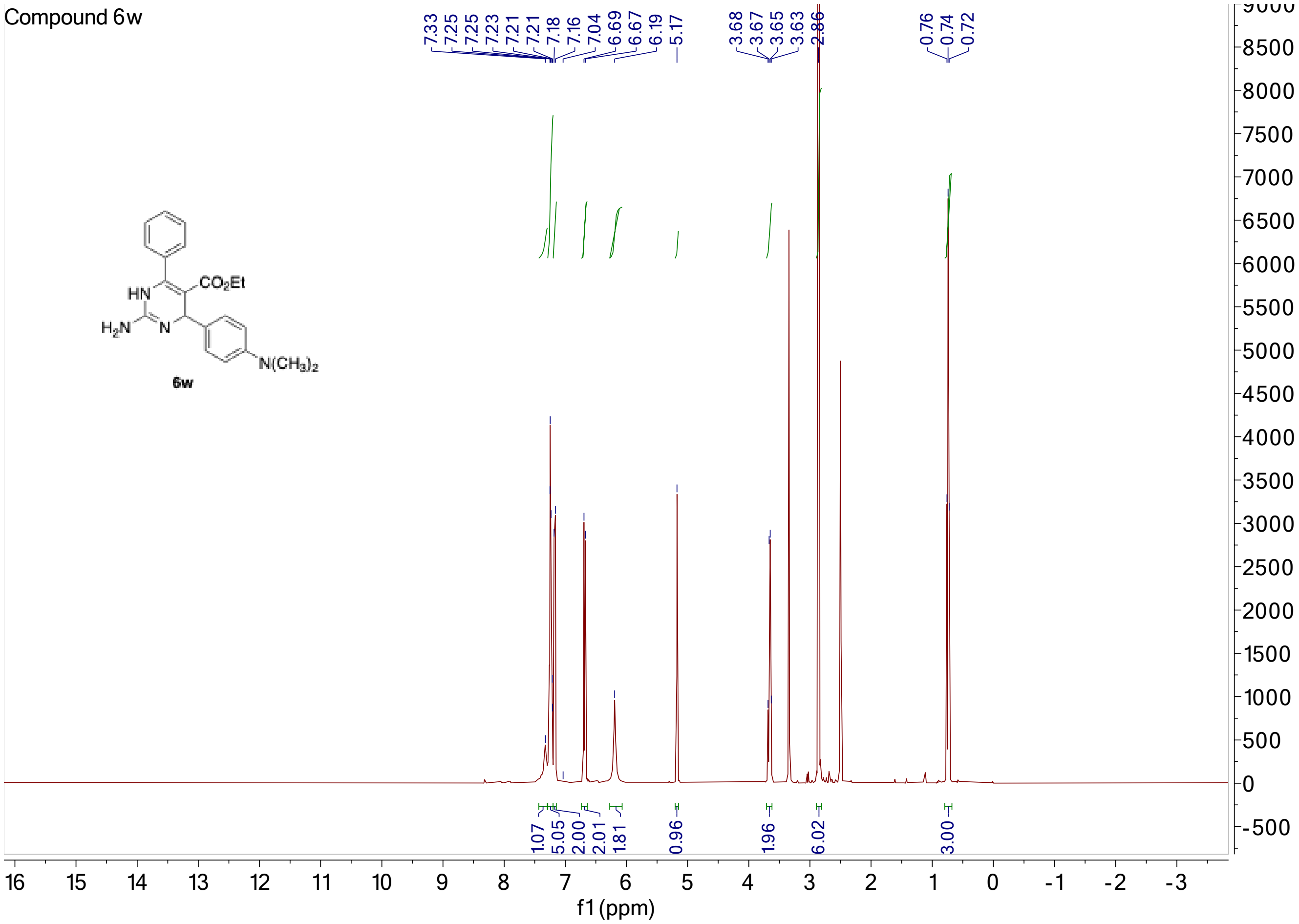
Compound 6w
HN
CO2Et
H2N
N
N(CH3)2
6w
7.33
7.25
7.25
7.23
7.21
7.21
7.18
7.16
7.04
6.69
6.67
6.19
5.17
3.68
3.67
3.65
3.63
2.86
0.76
0.74
0.72
1.07
5.05
2.00
2.01
1.81
0.96
1.96
6.02
3.00
f1 (ppm)

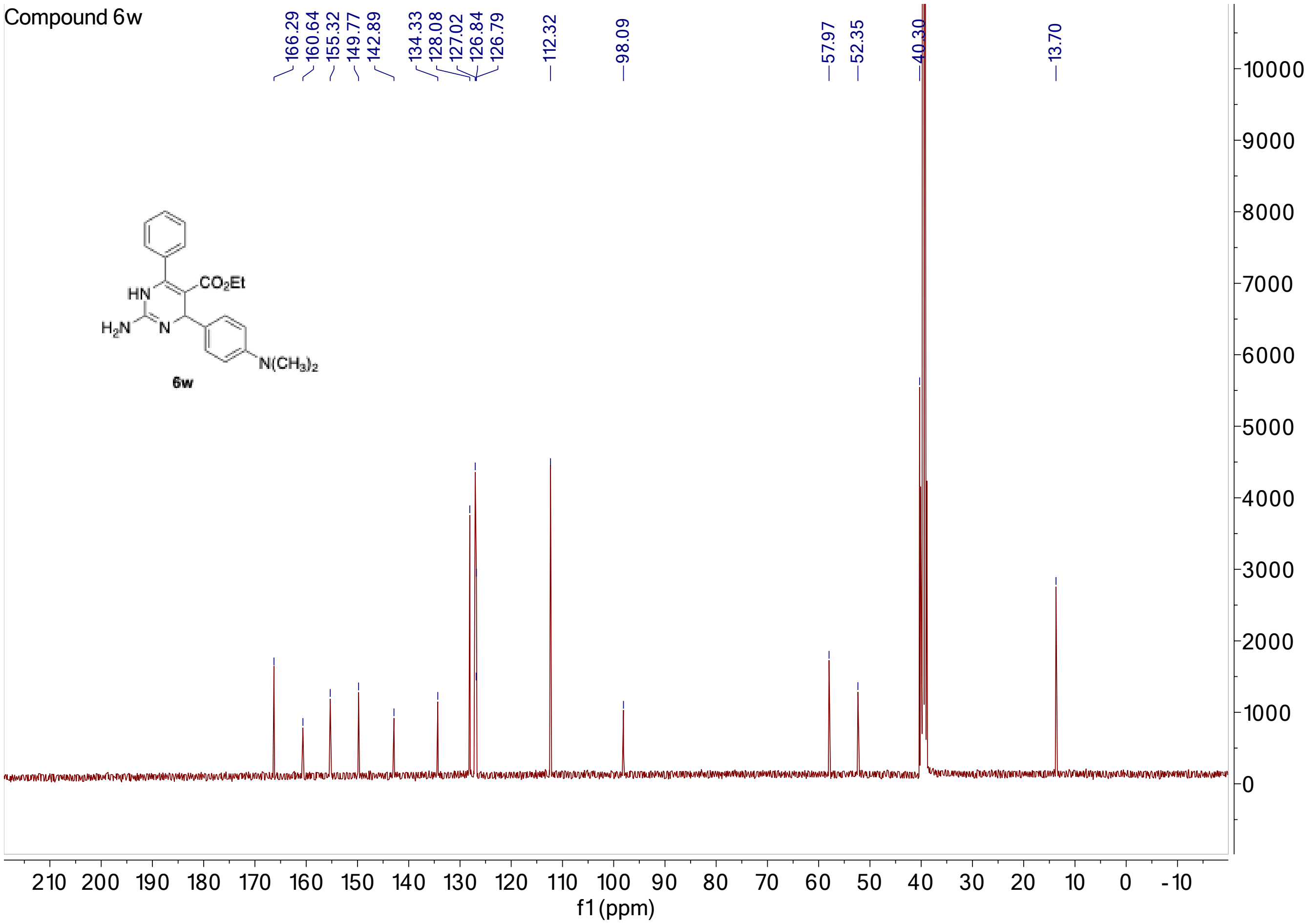
Compound 6w
166.29
160.64
155.32
149.77
142.89
134.33
128.08
127.02
126.84
126.79
112.32
98.09
57.97
52.35
40.30
13.70
CO2Et
HN
H2N
N
N(CH3)2
6w
10000
9000
8000
7000
6000
5000
4000
3000
2000
1000
0
210 200 190 180 170 160 150 140 130 120 110 100 90 80 70 60 50 40 30 20 10 0 -10
f1 (ppm)

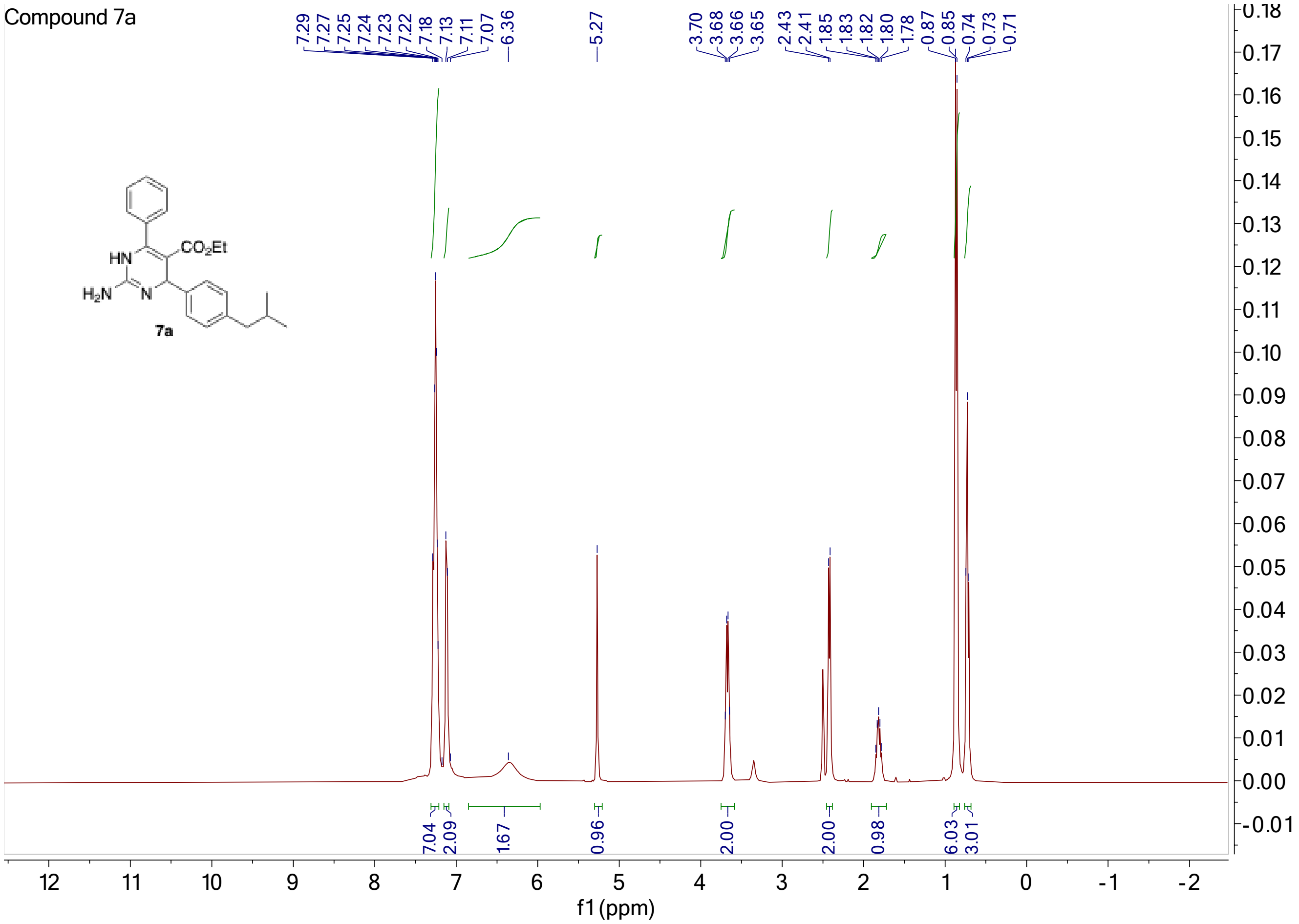
Compound 7a
7.29
7.27
7.25
7.24
7.23
7.22
7.18
7.13
7.11
7.07
6.36
5.27
3.70
3.68
3.66
3.65
2.43
2.41
1.85
1.83
1.82
1.80
1.78
0.87
0.85
0.74
0.73
0.71
HN
CO2Et
H2N
N
7a
7.04
2.09
1.67
0.96
2.00
2.00
0.98
6.03
3.01
f1 (ppm)

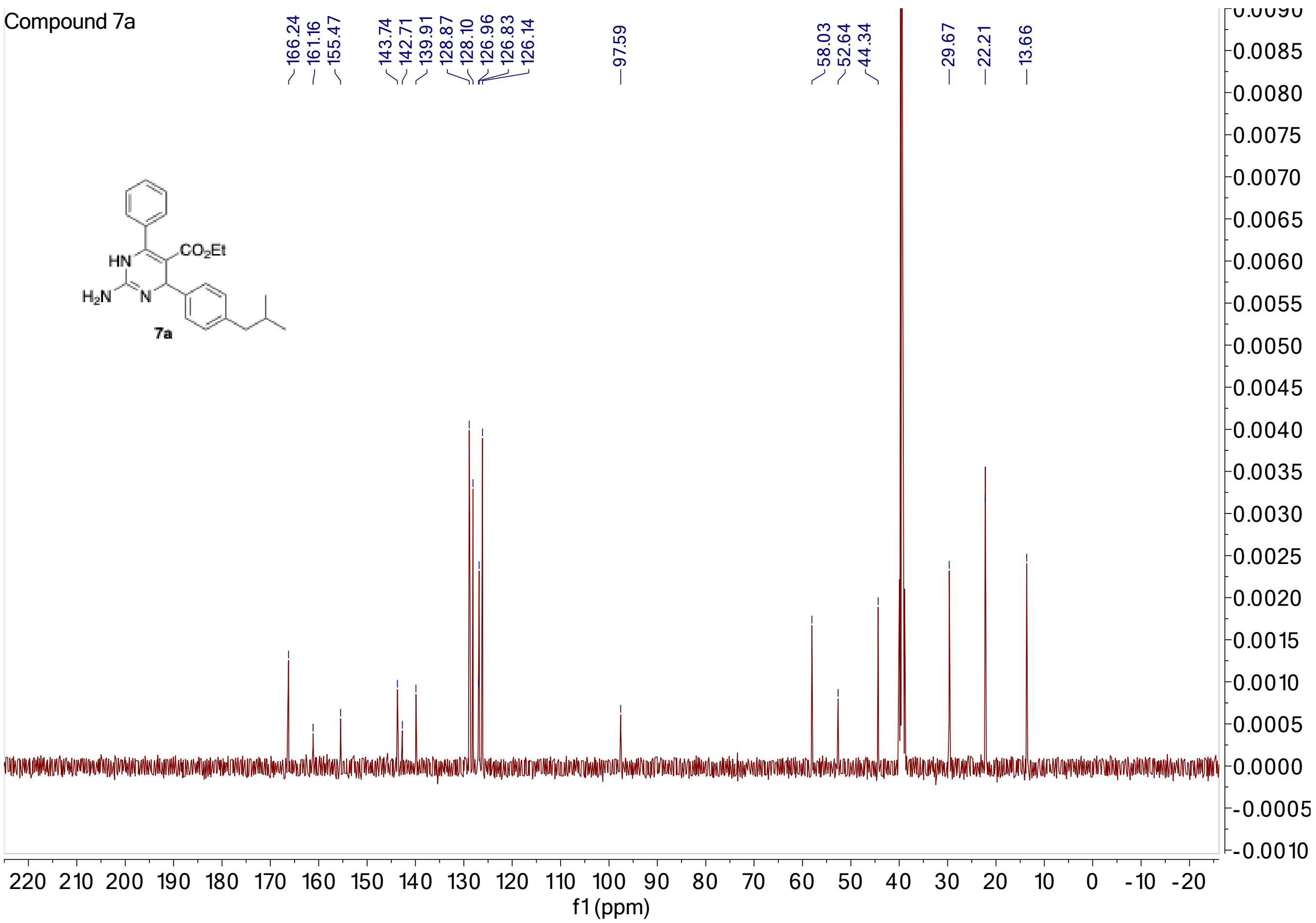
Compound 7a
166.24
161.16
155.47
143.74
142.71
139.91
128.87
128.10
126.96
126.83
126.14
97.59
58.03
52.64
44.34
29.67
22.21
13.66
HN
CO_2Et
H_2N
N
7a
f1 (ppm)

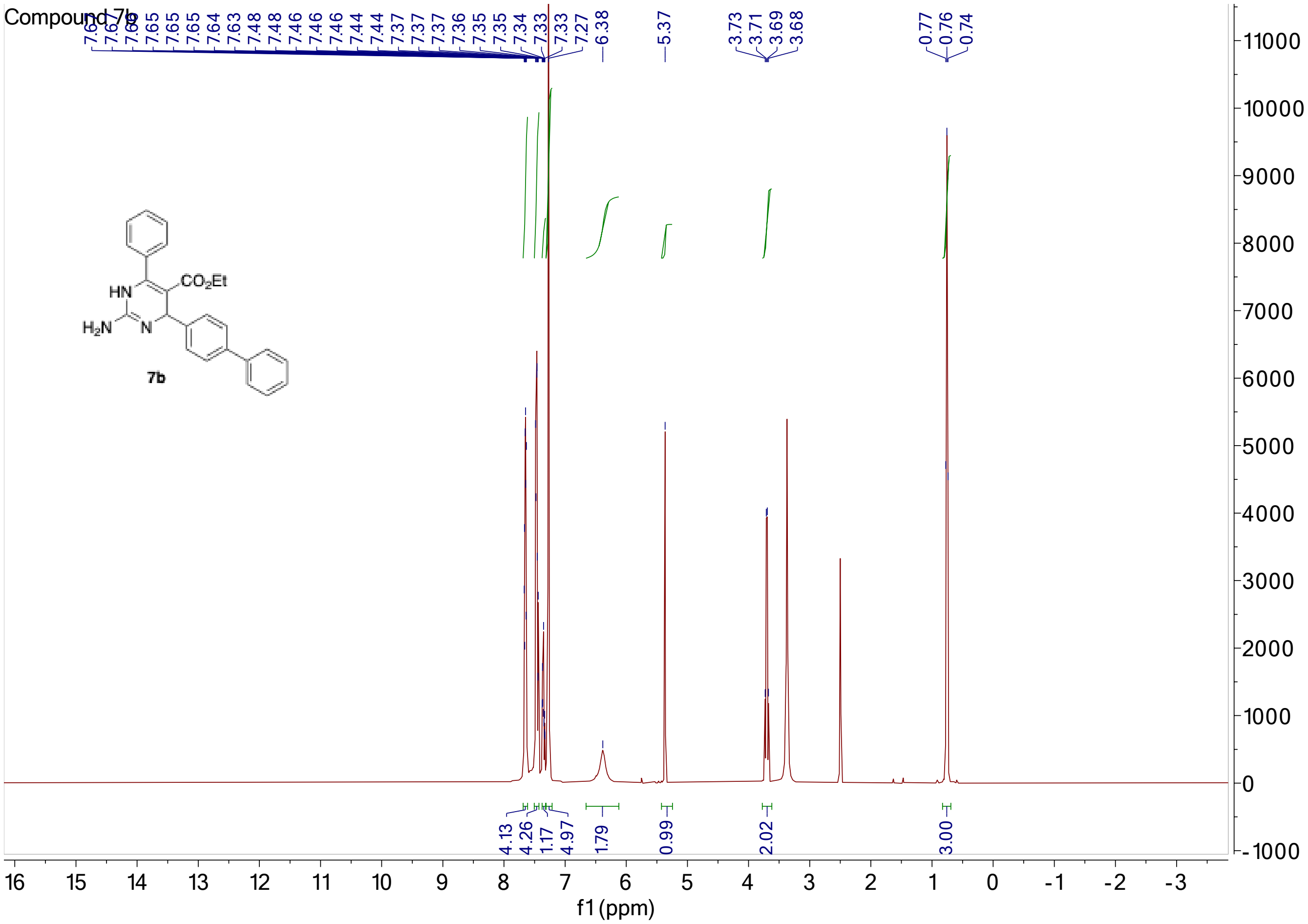
Compound 7b
7.65
7.65
7.64
7.63
7.48
7.48
7.46
7.46
7.46
7.44
7.44
7.37
7.37
7.37
7.36
7.35
7.35
7.34
7.33
7.33
7.27
6.38
5.37
3.73
3.71
3.69
3.68
0.77
0.76
0.74
CO2Et
HN
H2N
N
7b
4.13
4.26
1.17
4.97
1.79
0.99
2.02
3.00
f1 (ppm)

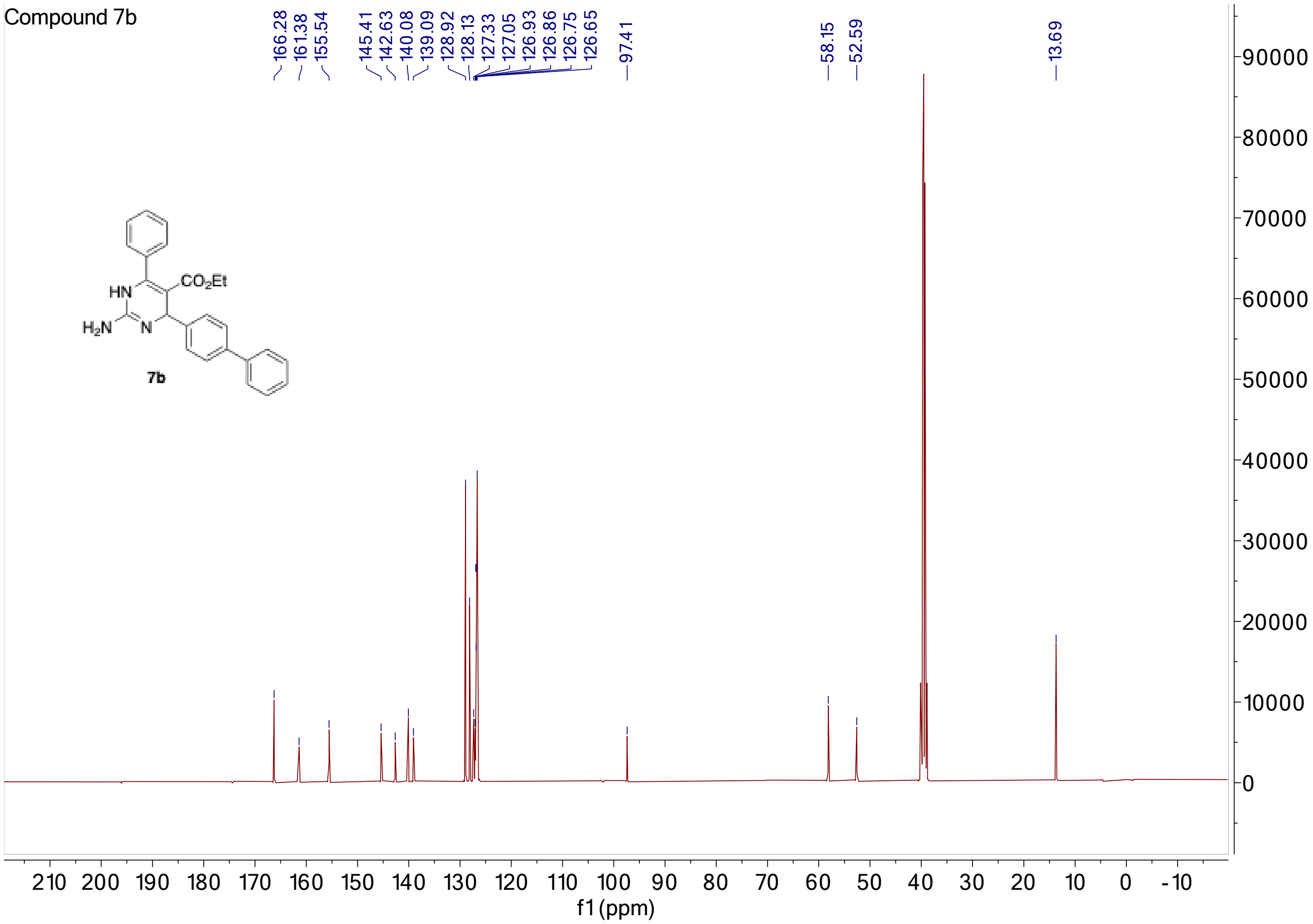
Compound 7b
166.28
161.38
155.54
145.41
142.63
140.08
139.09
128.92
128.13
127.33
127.05
126.93
126.86
126.75
126.65
97.41
58.15
52.59
13.69
HN
CO2Et
H2N
N
7b
90000
80000
70000
60000
50000
40000
30000
20000
10000
0
210 200 190 180 170 160 150 140 130 120 110 100 90 80 70 60 50 40 30 20 10 0 -10
f1 (ppm)

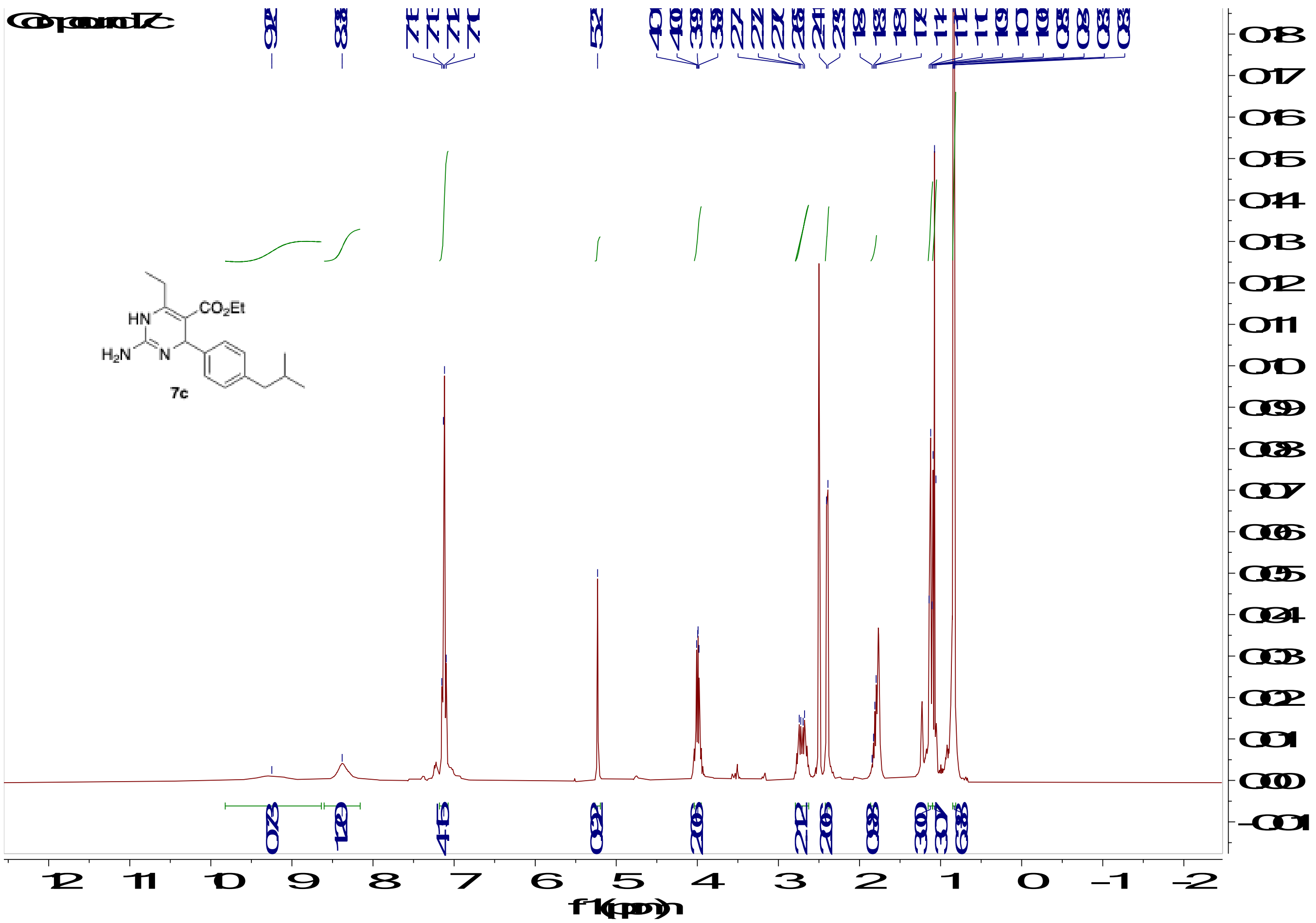
HN
CO2Et
H2N
N
7c

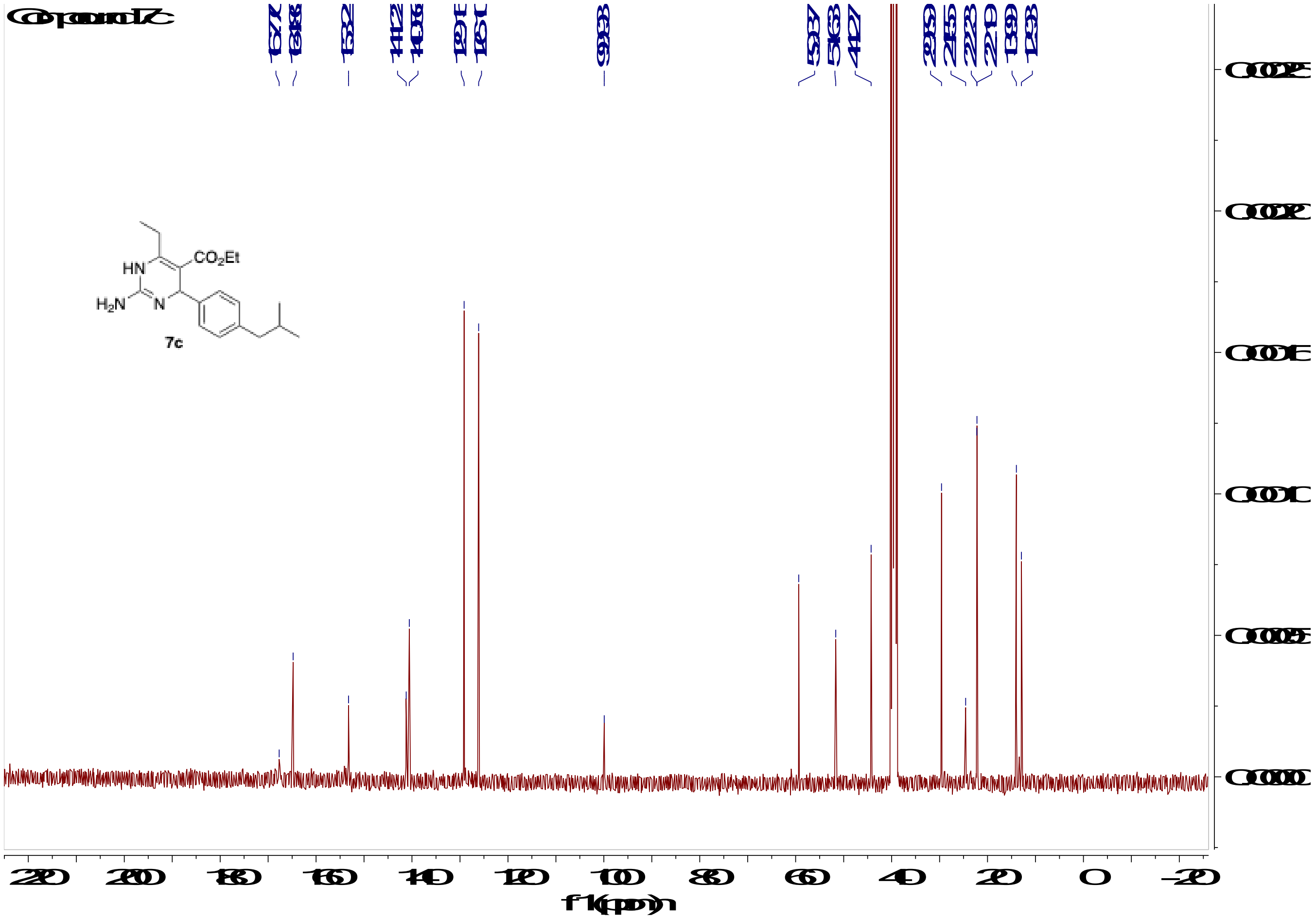
CO2Et
HN
H2N
N
7c

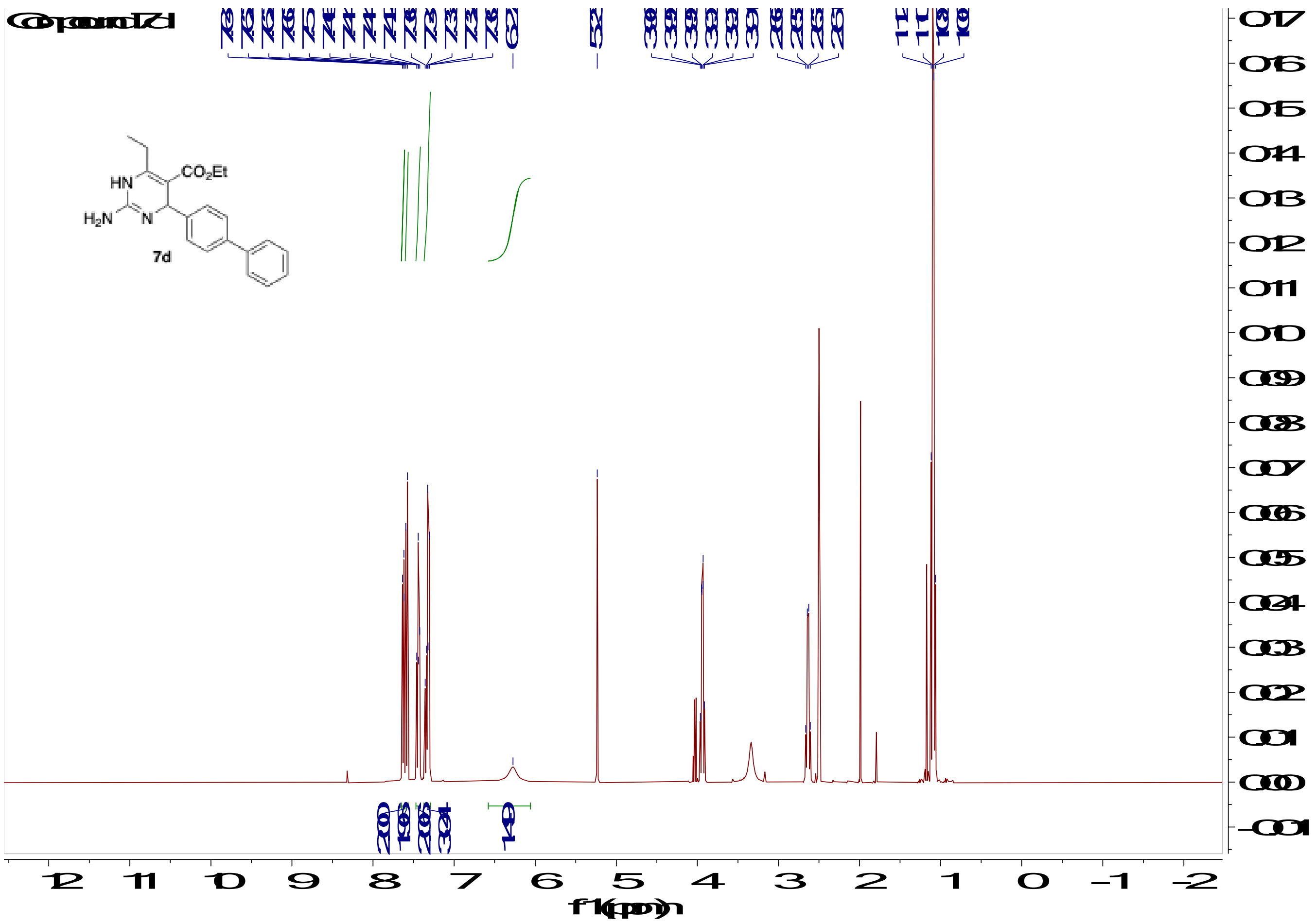

HN
CO2Et
H2N
N
7d
12
11
10
9
8
7
6
5
4
3
2
1
0
-1
-2

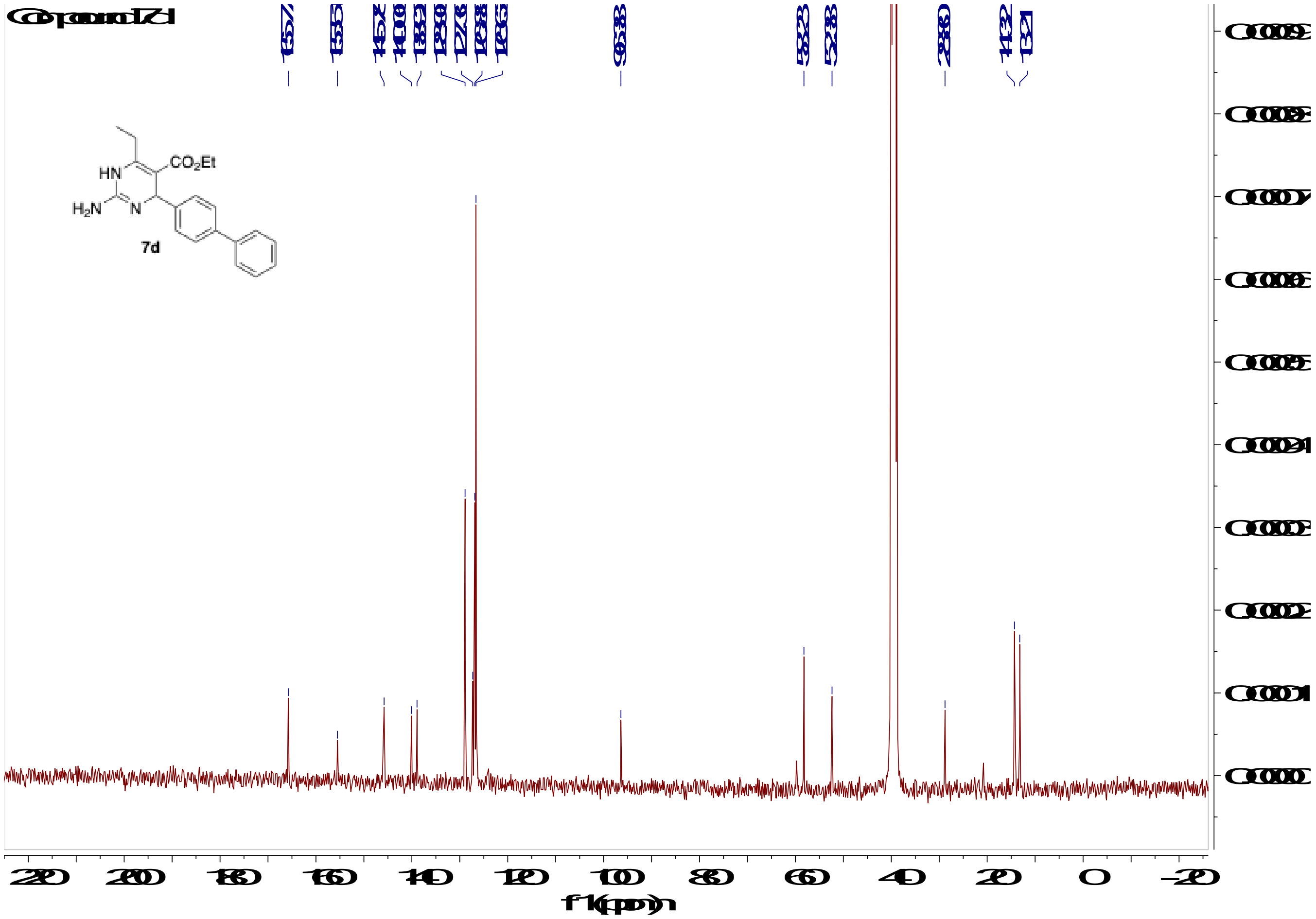
CO2Et
HN
H2N
N
7d

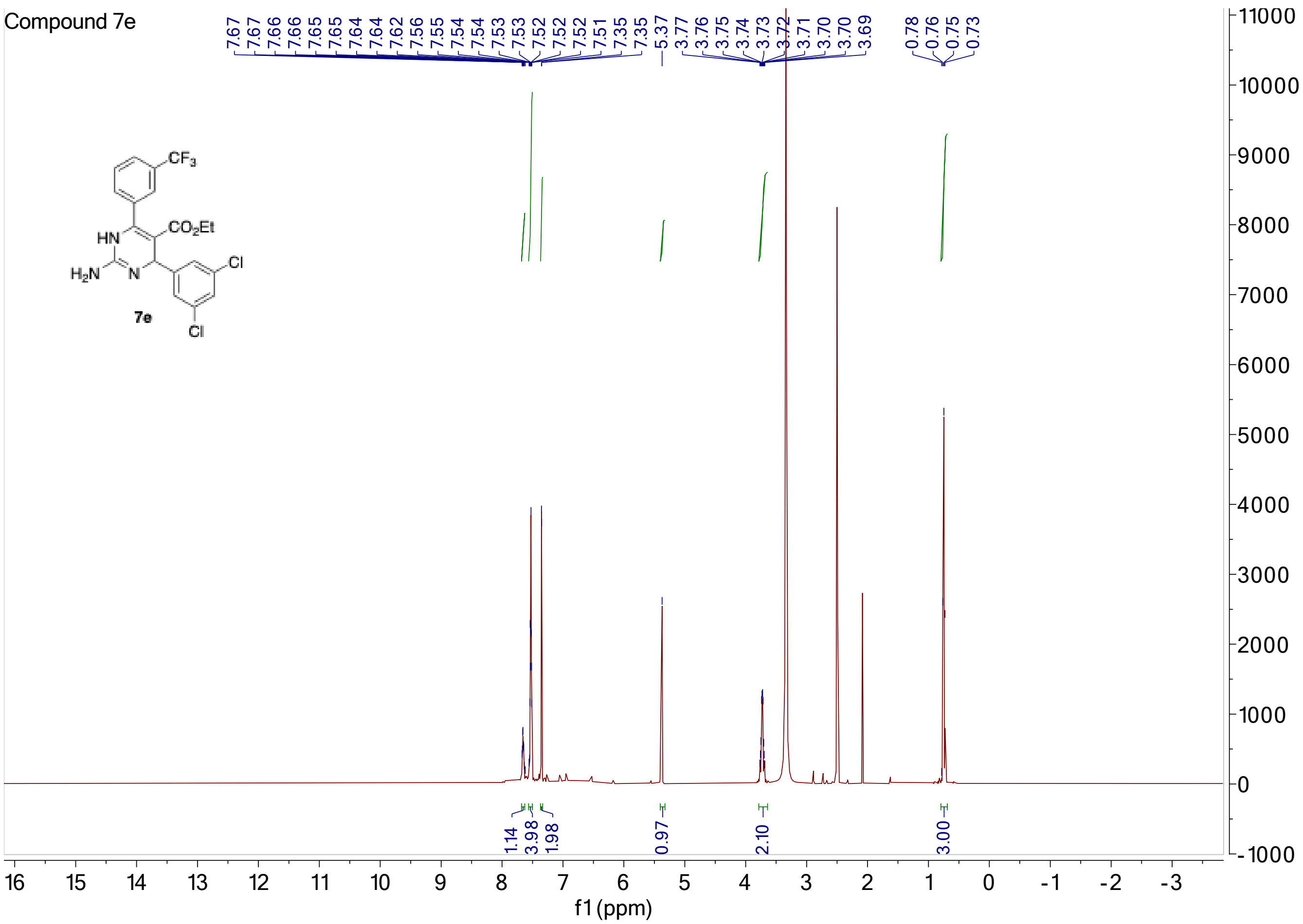
Compound 7e
7.67
7.67
7.66
7.66
7.65
7.65
7.64
7.64
7.62
7.56
7.55
7.54
7.54
7.53
7.53
7.52
7.52
7.52
7.51
7.35
7.35
5.37
3.77
3.76
3.75
3.74
3.73
3.72
3.71
3.70
3.70
3.69
0.78
0.76
0.75
0.73
CF3
HN
CO2Et
Cl
H2N
N
7e
Cl
1.14
3.98
1.98
0.97
2.10
3.00
f1 (ppm)

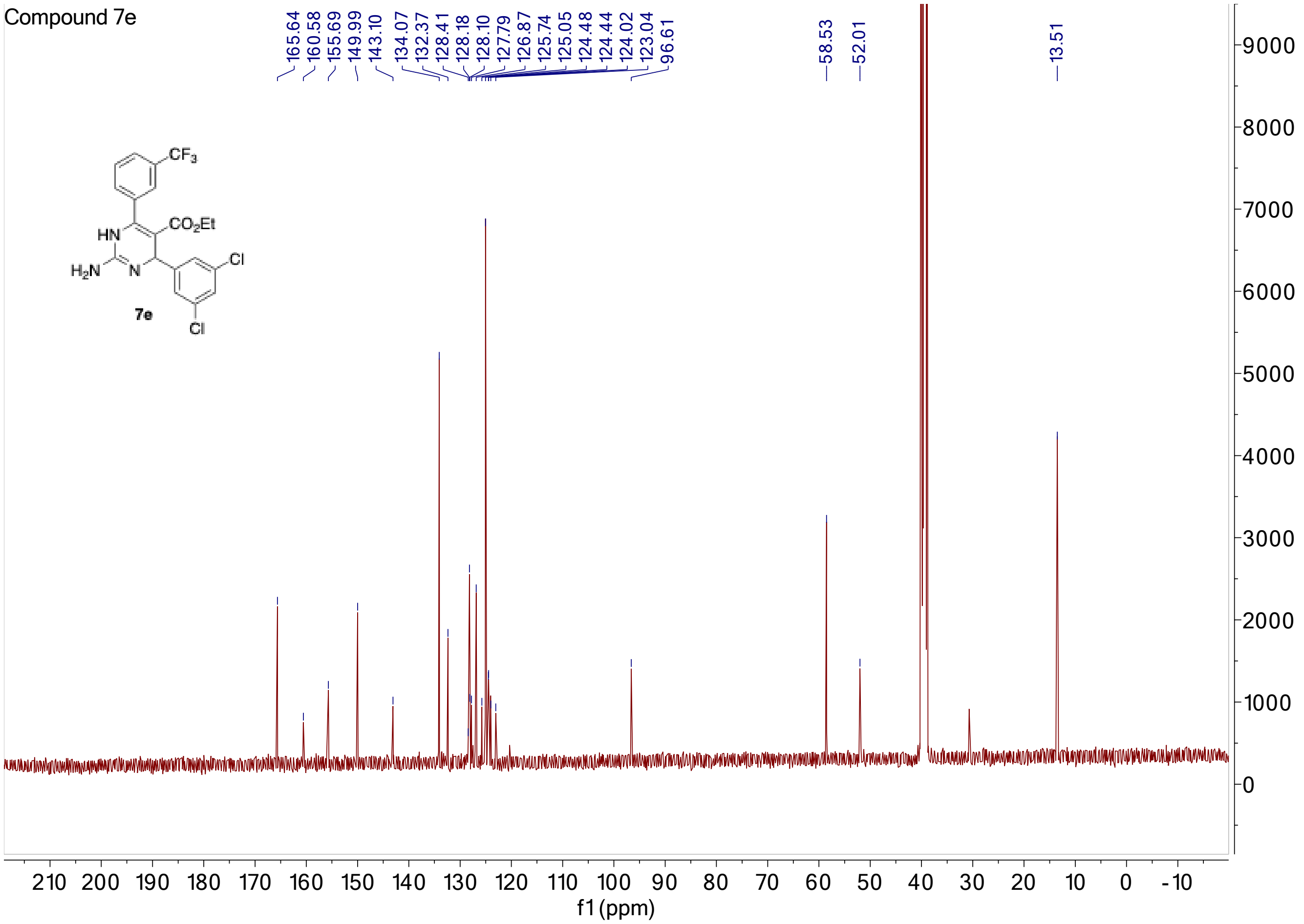
Compound 7e
165.64
160.58
155.69
149.99
143.10
134.07
132.37
128.41
128.18
128.10
127.79
126.87
125.74
125.05
124.48
124.44
124.02
123.04
96.61
58.53
52.01
13.51
CF3
CO2Et
HN
H2N
N
Cl
Cl
7e
9000
8000
7000
6000
5000
4000
3000
2000
1000
0
210 200 190 180 170 160 150 140 130 120 110 100 90 80 70 60 50 40 30 20 10 0 -10
f1 (ppm)

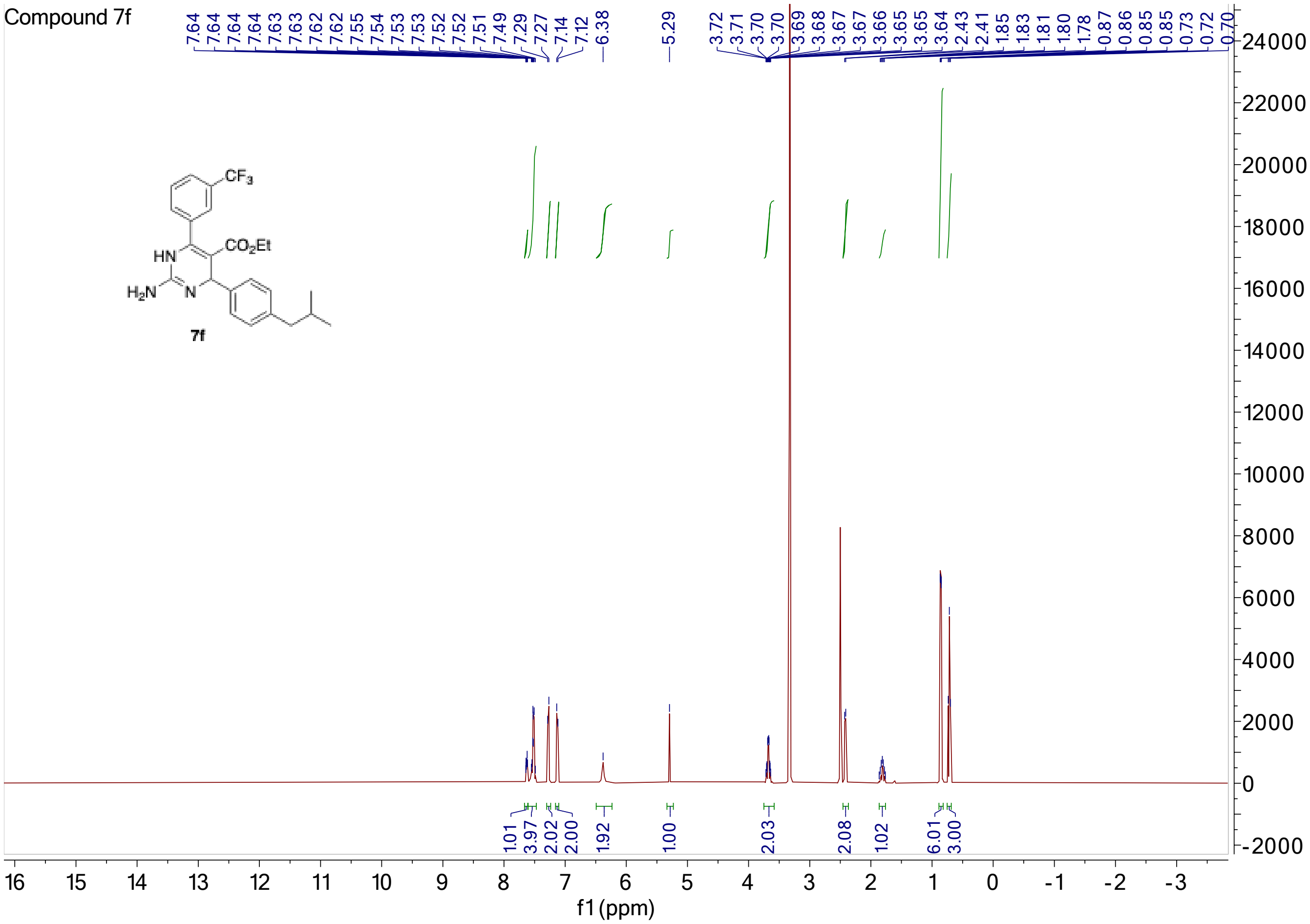
Compound 7f
7.64 7.64 7.64 7.64 7.63 7.63 7.62 7.62 7.55 7.54 7.53 7.53 7.52 7.52 7.51 7.49 7.29 7.27 7.14 7.12 6.38
5.29
3.72 3.71 3.70 3.70 3.69 3.68 3.67 3.67 3.66 3.65 3.65 3.64 2.43 2.41 1.85 1.83 1.81 1.80 1.78 0.87 0.86 0.85 0.85 0.73 0.72 0.70
CF3
CO2Et
HN
H2N
N
7f
1.01 3.97 2.02 2.00 1.92 1.00 2.03 2.08 1.02 6.01 3.00
f1 (ppm)

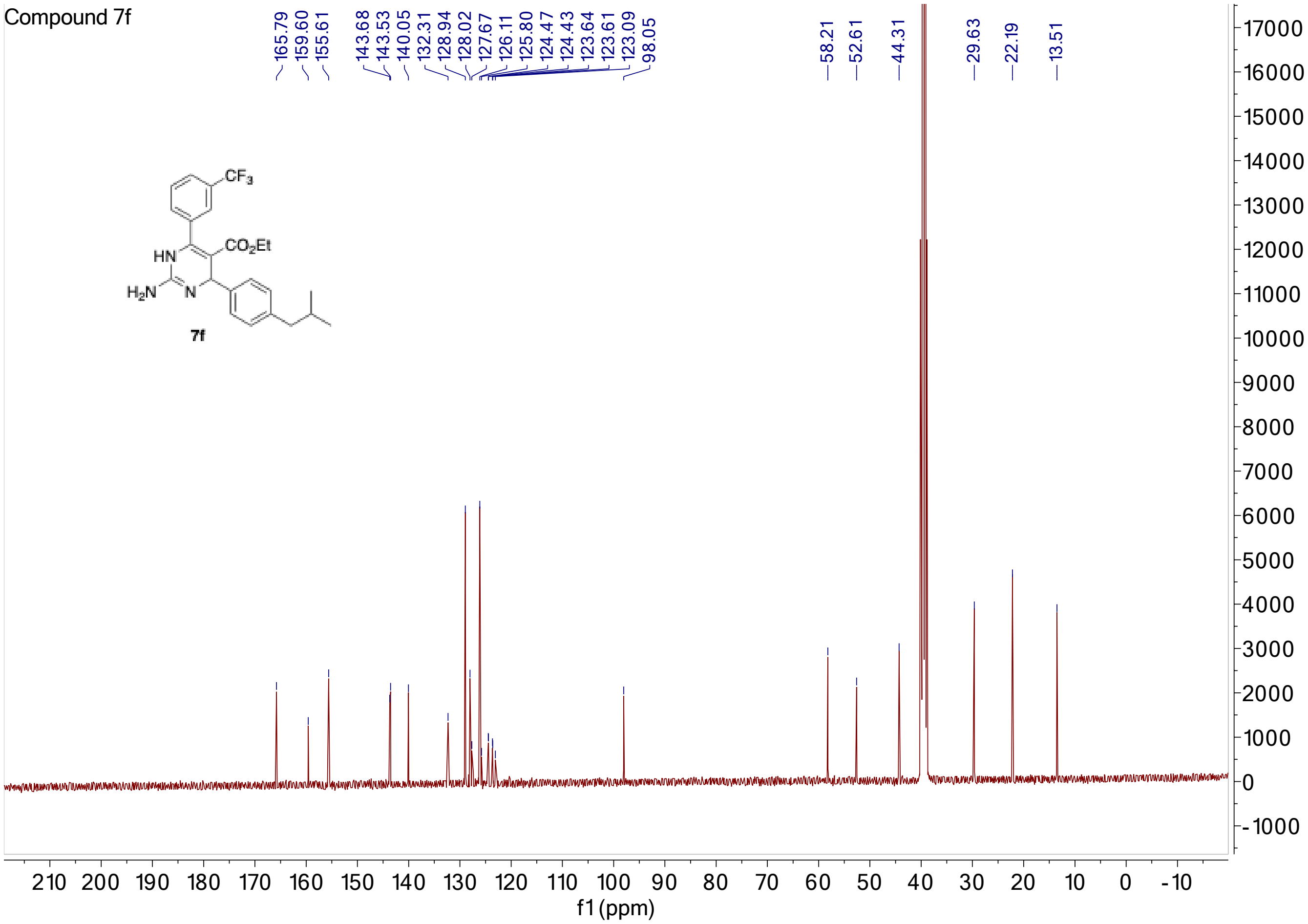
Compound 7f
165.79
159.60
155.61
143.68
143.53
140.05
132.31
128.94
128.02
127.67
126.11
125.80
124.47
124.43
123.64
123.61
123.09
98.05
58.21
52.61
44.31
29.63
22.19
13.51
CF3
CO2Et
HN
H2N
N
7f
f1 (ppm)

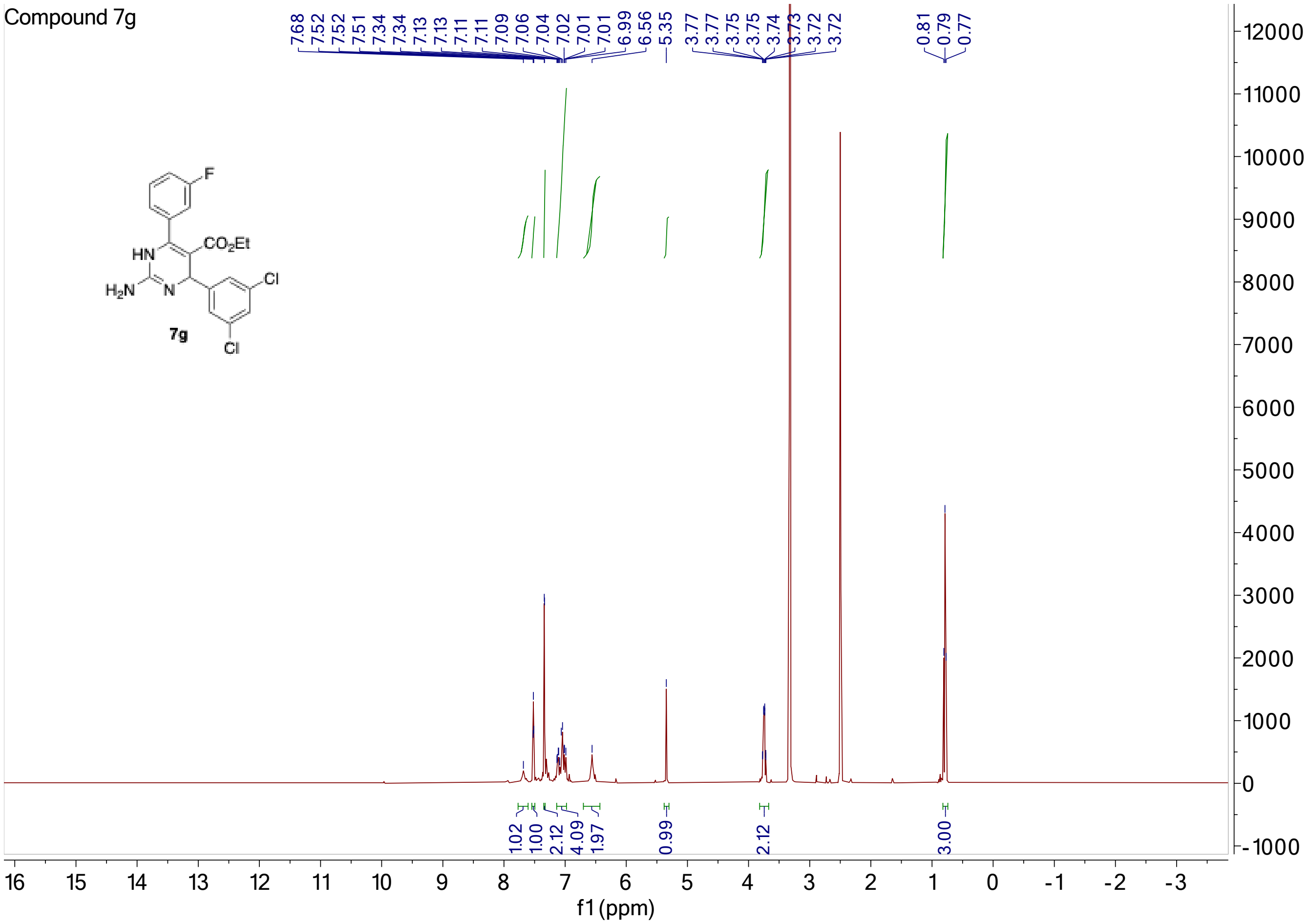
Compound 7g
7.68 7.52 7.52 7.51 7.34 7.34 7.13 7.13 7.11 7.11 7.09 7.06 7.04 7.02 7.01 7.01 6.99 6.56
5.35
3.77 3.77 3.75 3.75 3.74 3.73 3.72 3.72
0.81 0.79 0.77
F
HN
CO2Et
H2N
N
Cl
7g
Cl
1.02 1.00 2.12 4.09 1.97 0.99 2.12 3.00
12000 11000 10000 9000 8000 7000 6000 5000 4000 3000 2000 1000 0 -1000
16 15 14 13 12 11 10 9 8 7 6 5 4 3 2 1 0 -1 -2 -3
f1 (ppm)

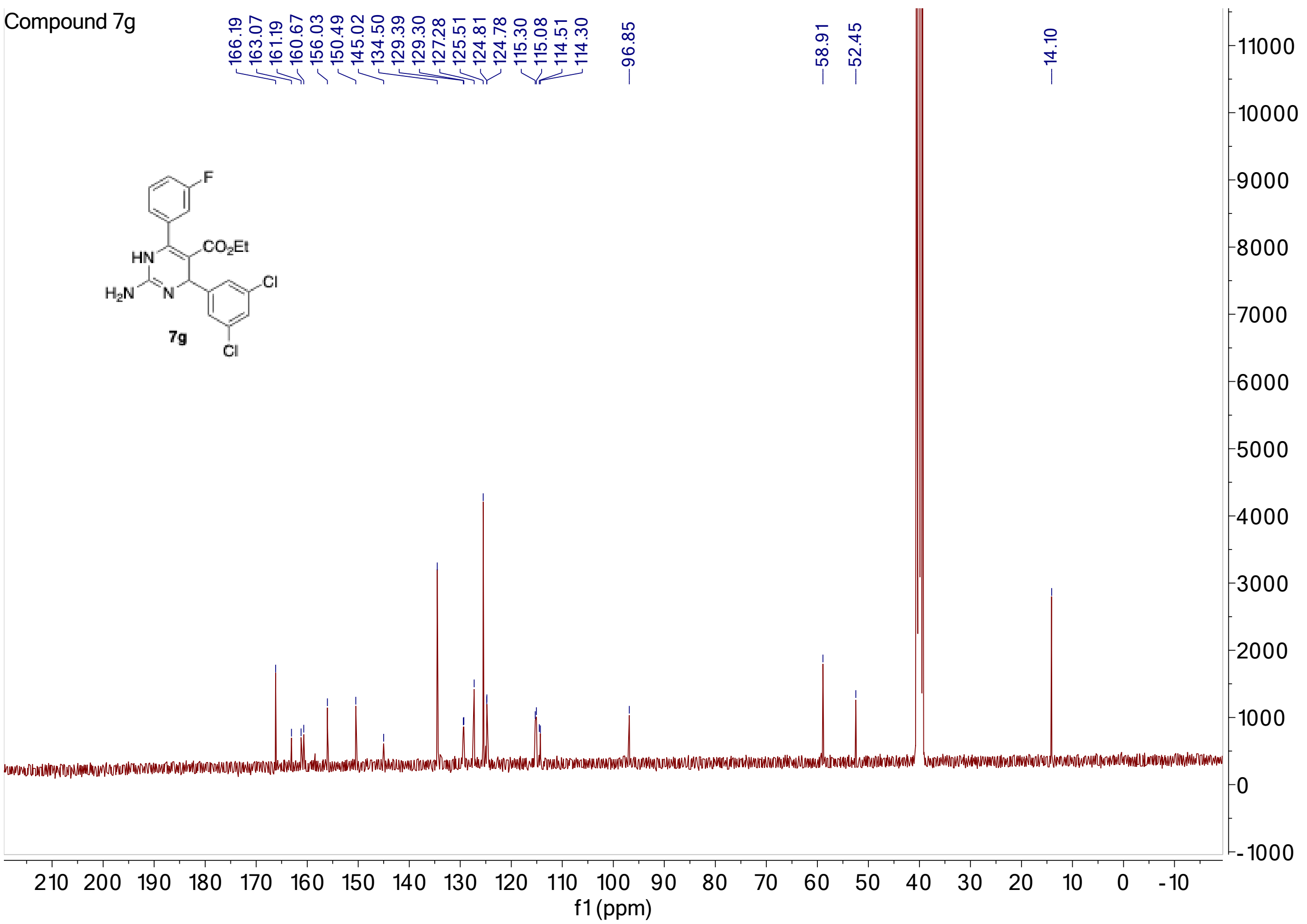
Compound 7g
166.19
163.07
161.19
160.67
156.03
150.49
145.02
134.50
129.39
129.30
127.28
125.51
124.81
124.78
115.30
115.08
114.51
114.30
96.85
58.91
52.45
14.10
F
CO_2Et
HN
H_2N
N
Cl
Cl
7g
11000
10000
9000
8000
7000
6000
5000
4000
3000
2000
1000
0
-1000
210 200 190 180 170 160 150 140 130 120 110 100 90 80 70 60 50 40 30 20 10 0 -10
f1 (ppm)

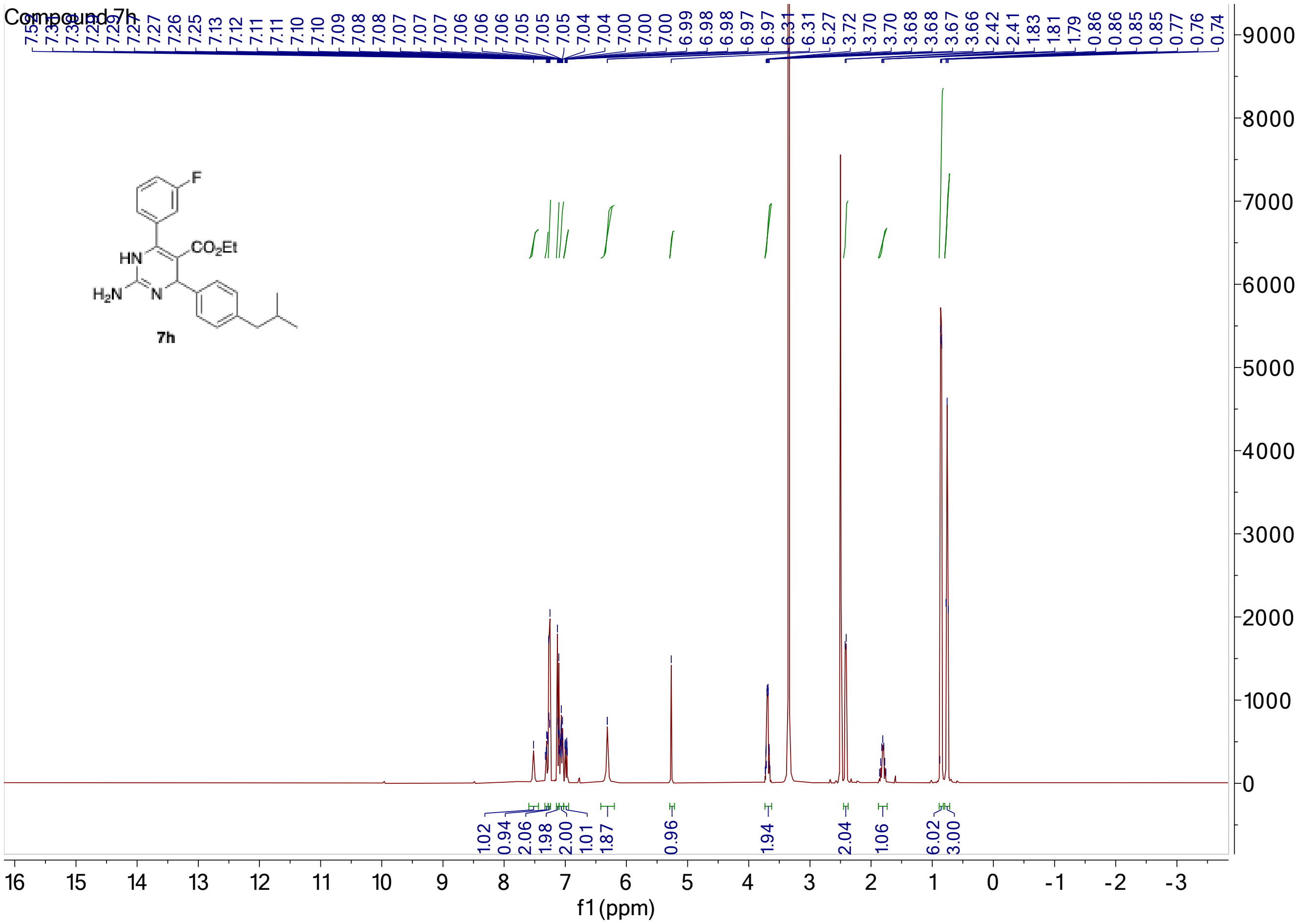
Compound 7h
F
HN
CO2Et
H2N
N
7h
1.02
0.94
2.06
1.98
2.00
1.01
1.87
0.96
1.94
2.04
1.06
6.02
3.00
f1 (ppm)

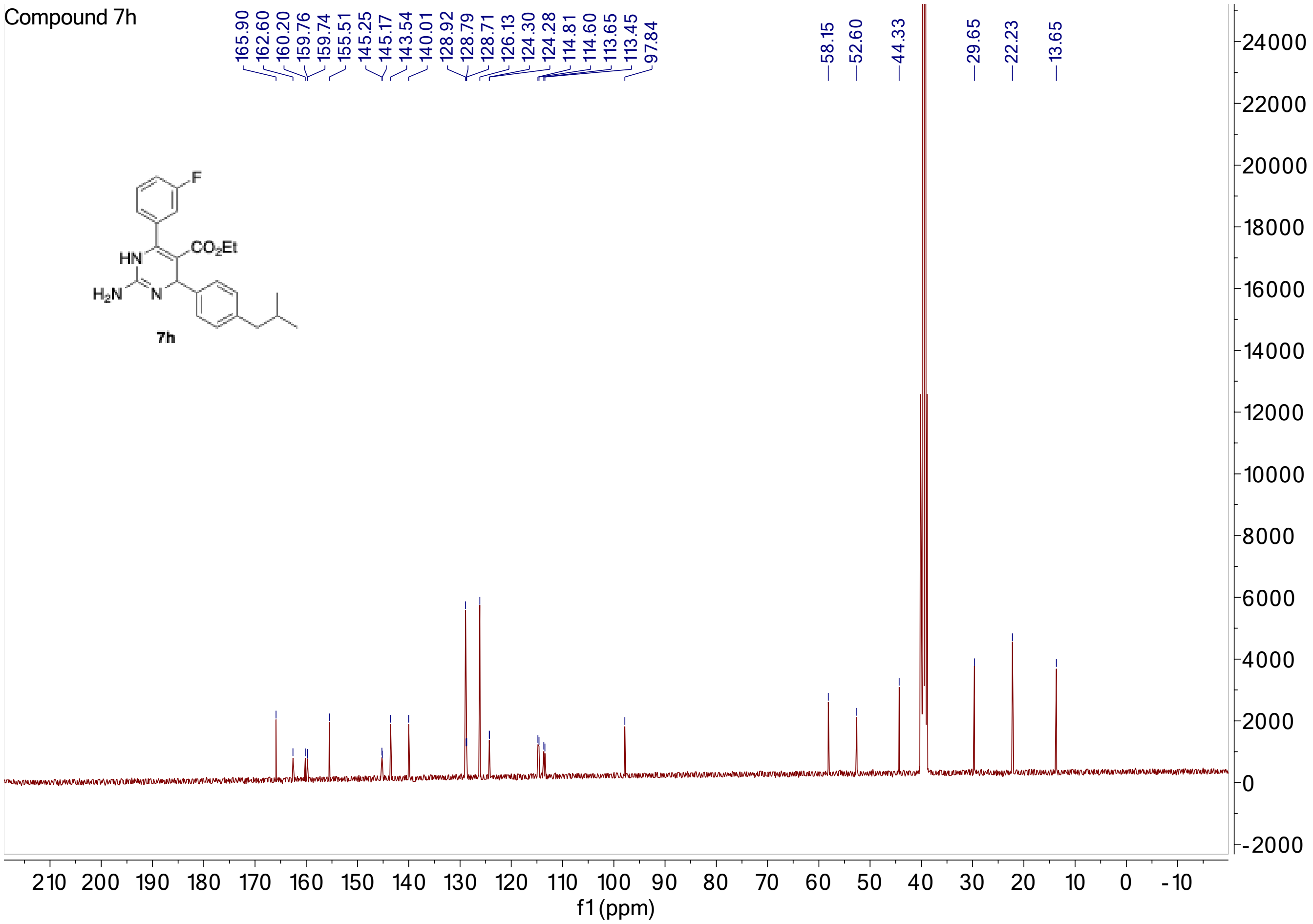
Compound 7h
165.90
162.60
160.20
159.76
159.74
155.51
145.25
145.17
143.54
140.01
128.92
128.79
128.71
126.13
124.30
124.28
114.81
114.60
113.65
113.45
97.84
58.15
52.60
44.33
29.65
22.23
13.65
F
HN
CO2Et
H2N
N
7h
f1 (ppm)

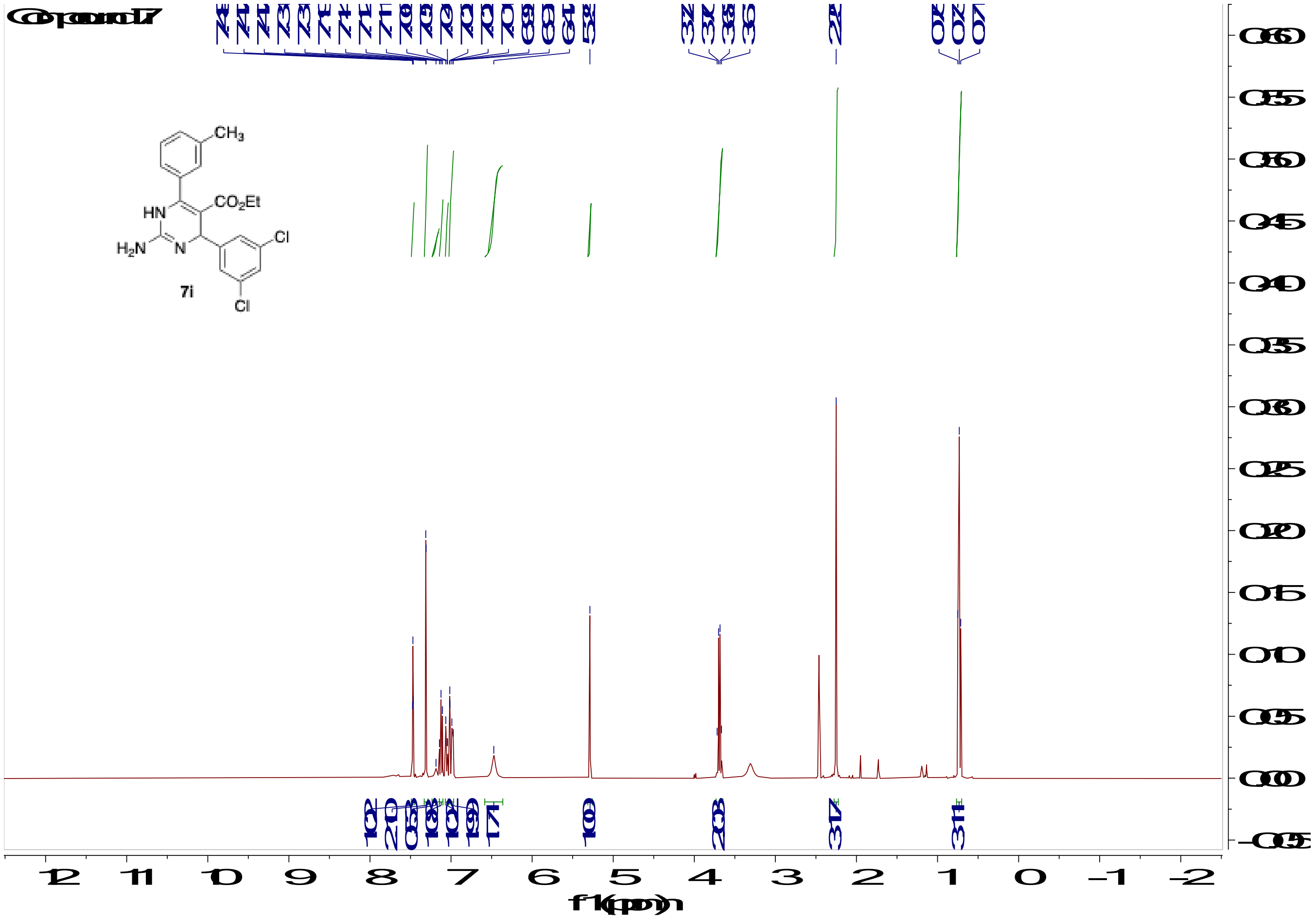
CH3
CO2Et
HN
H2N
N
Cl
Cl
7i

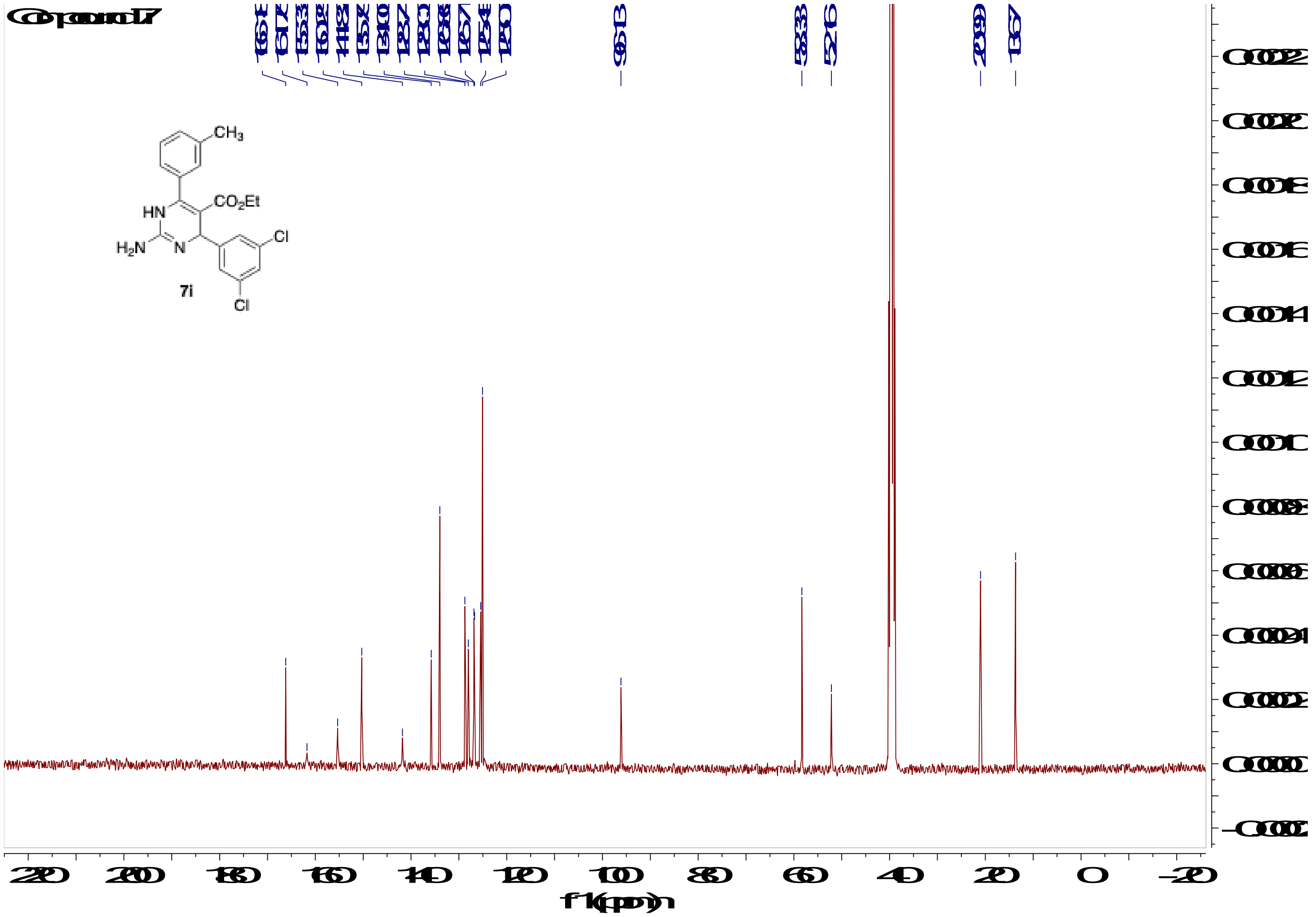
CH3
CO2Et
HN
H2N
N
Cl
Cl
7i

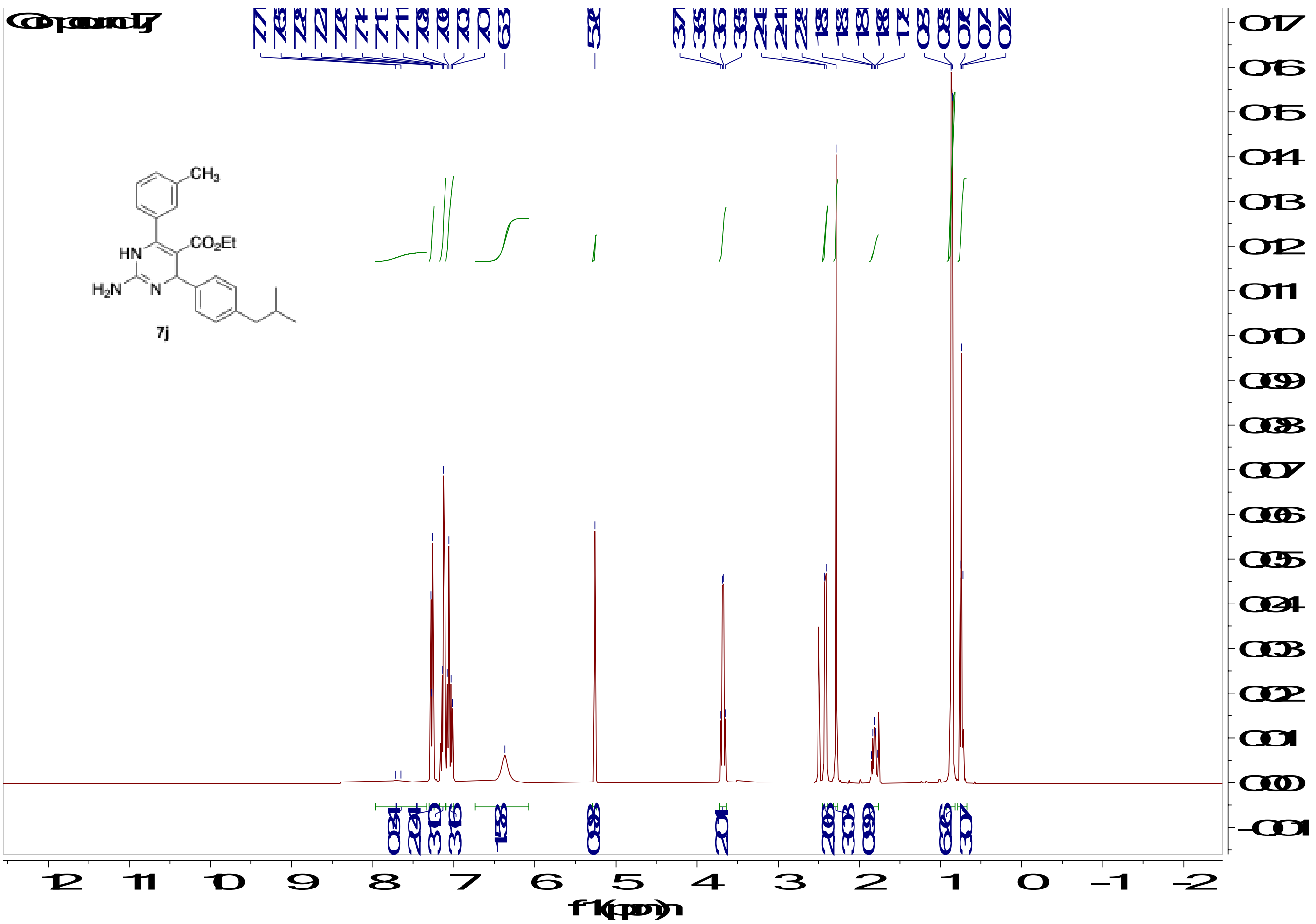
CH3
CO2Et
HN
H2N
N
7j
f1 (ppm)

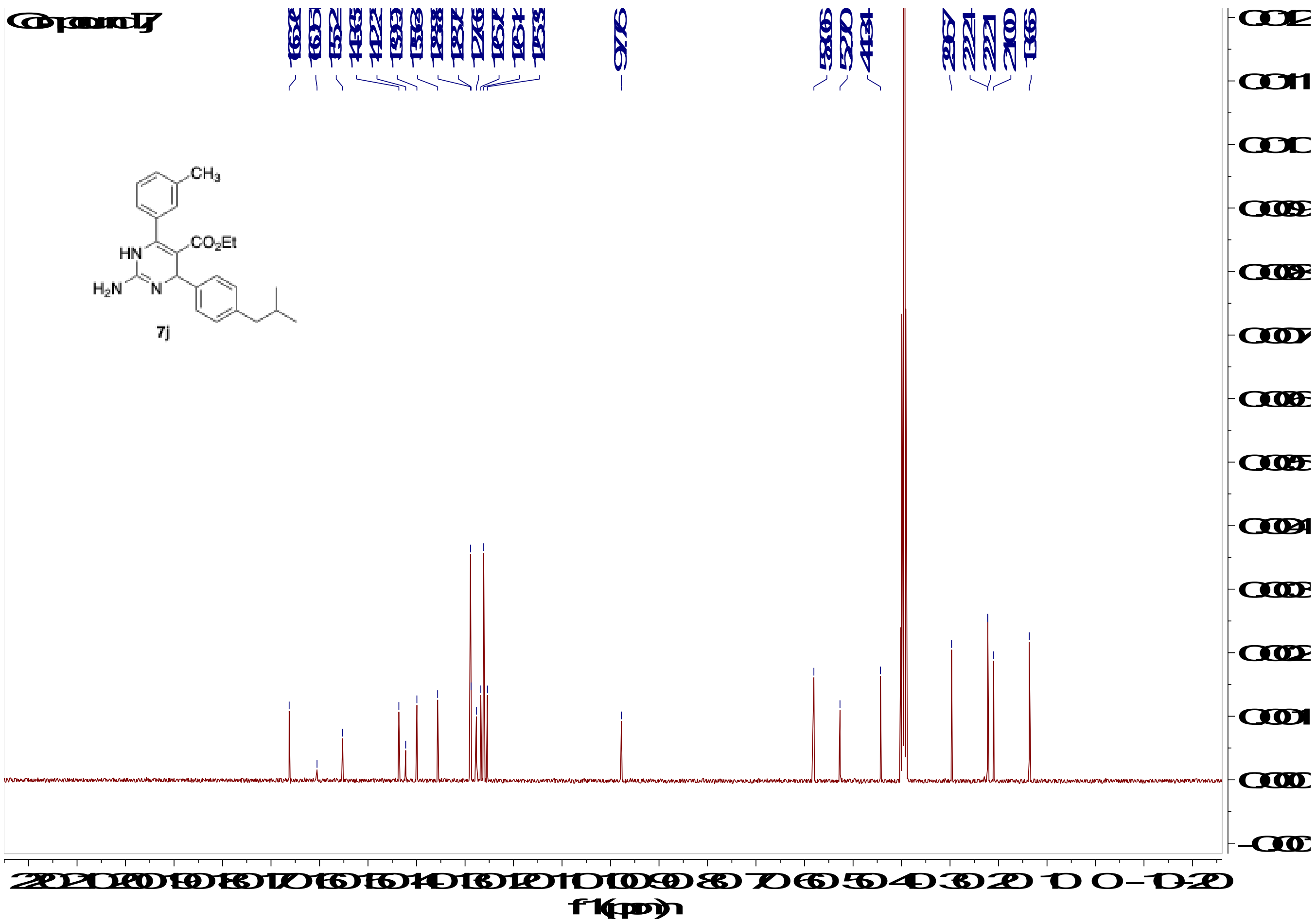
CH3
CO2Et
HN
H2N
N
7j

Compound 8a

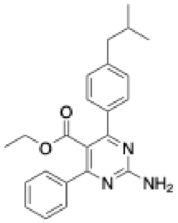

8a

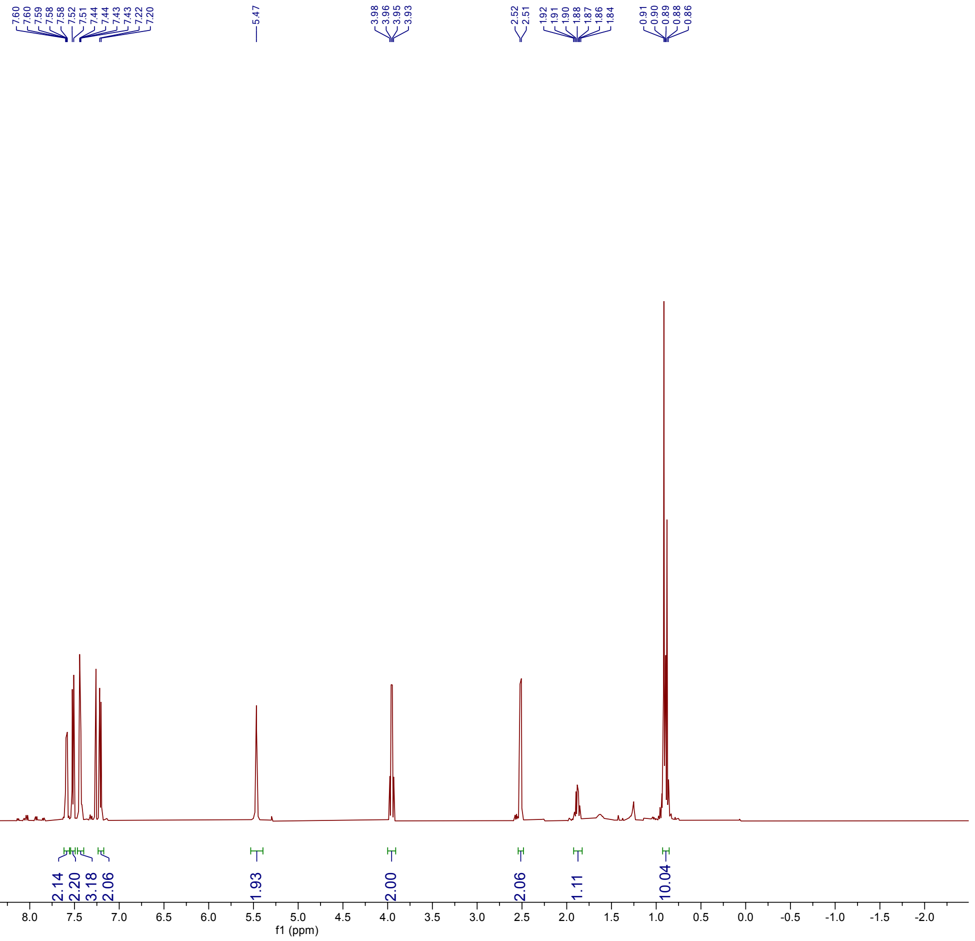

2040

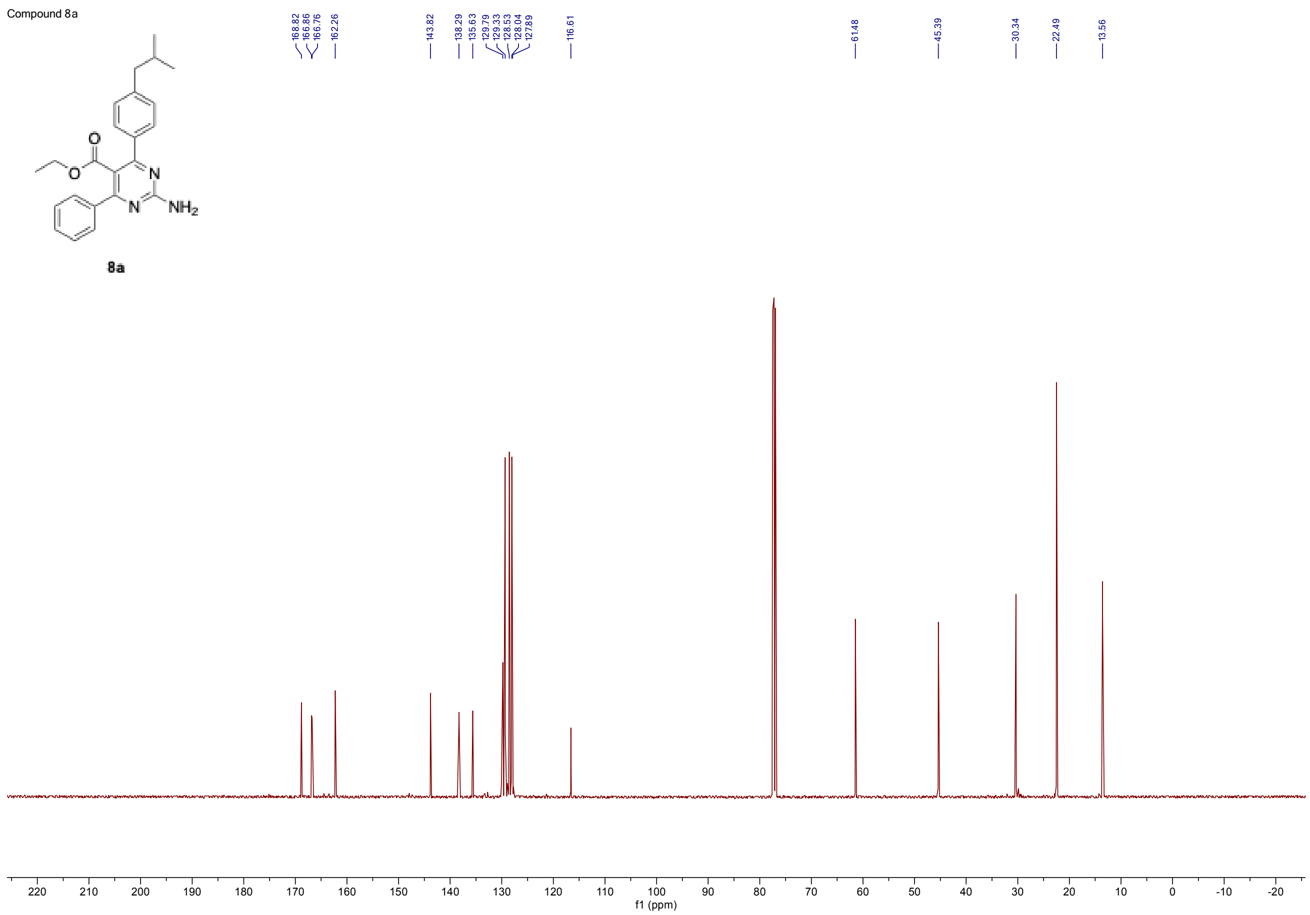
Compound 8a
168.82
166.86
166.76
162.26
143.82
138.29
135.63
129.79
129.33
128.53
128.04
127.89
116.61
61.48
45.39
30.34
22.49
13.56
O
O
N
N
NH2
8a
220
210
200
190
180
170
160
150
140
130
120
110
100
90
80
70
60
50
40
30
20
10
0
-10
-20
f1 (ppm)

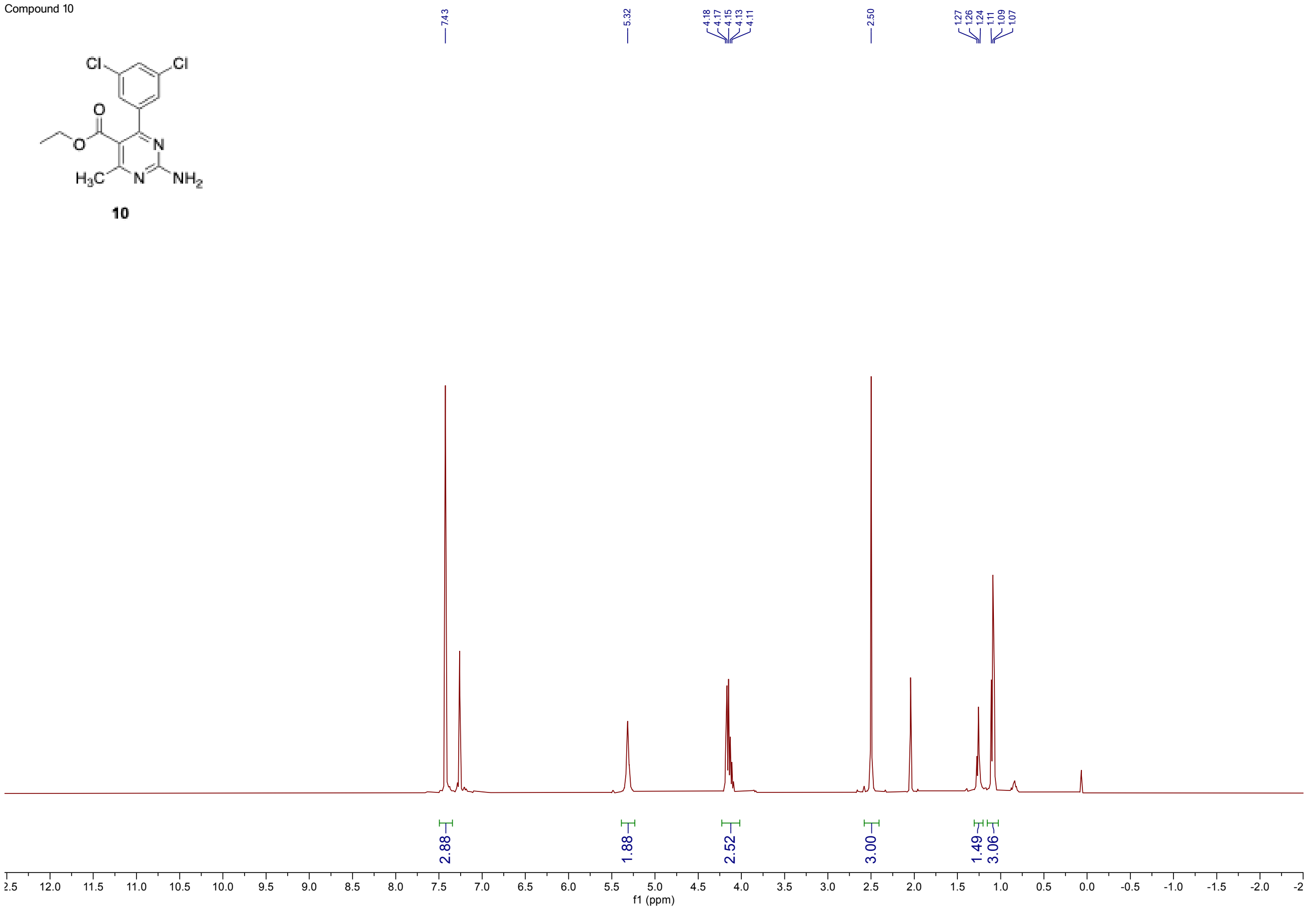

Compound 10
Cl
Cl
O
O
N
H_3C
N
NH_2
10
7.43
5.32
4.18
4.17
4.15
4.13
4.11
2.50
1.27
1.26
1.24
1.11
1.09
1.07
2.88
1.88
2.52
3.00
1.49
3.06
12.0
11.5
11.0
10.5
10.0
9.5
9.0
8.5
8.0
7.5
7.0
6.5
6.0
5.5
5.0
4.5
4.0
3.5
3.0
2.5
2.0
1.5
1.0
0.5
0.0
-0.5
-1.0
-1.5
-2.0
f1 (ppm)

Compound 18

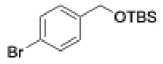

18

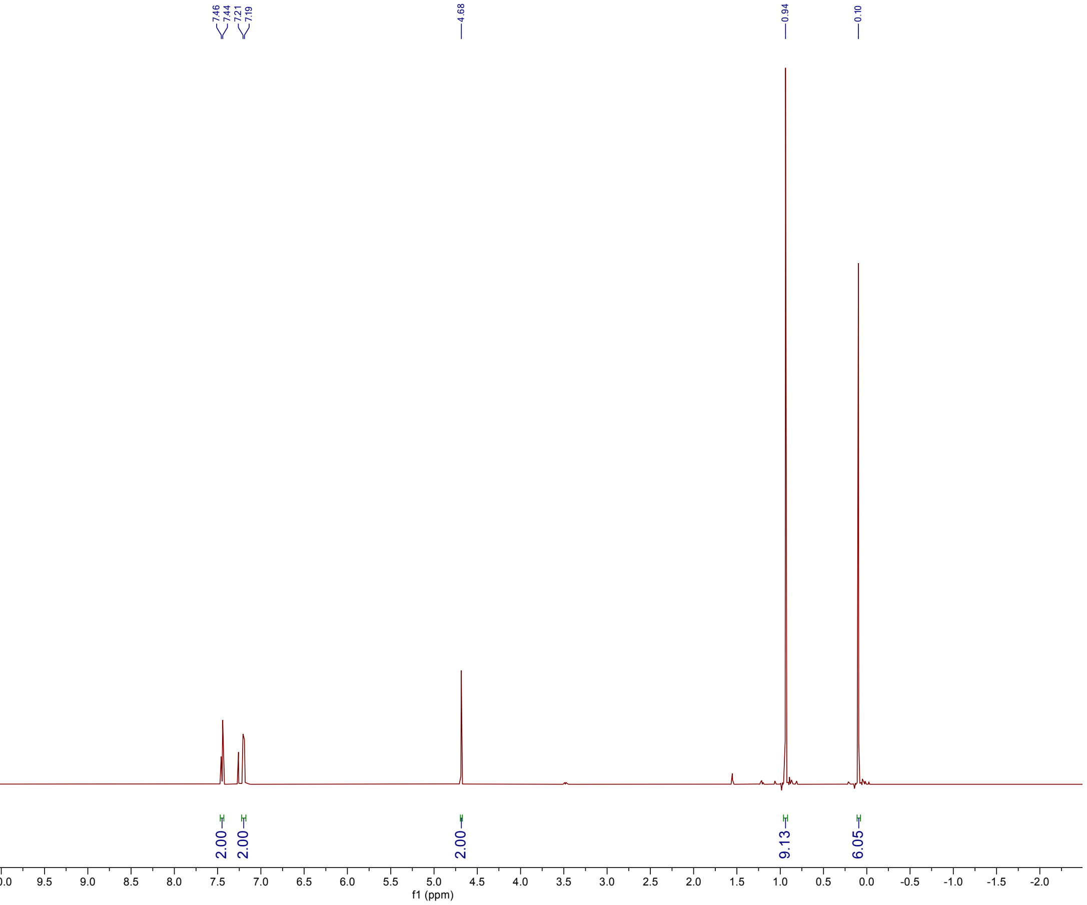

2045

Compound 19

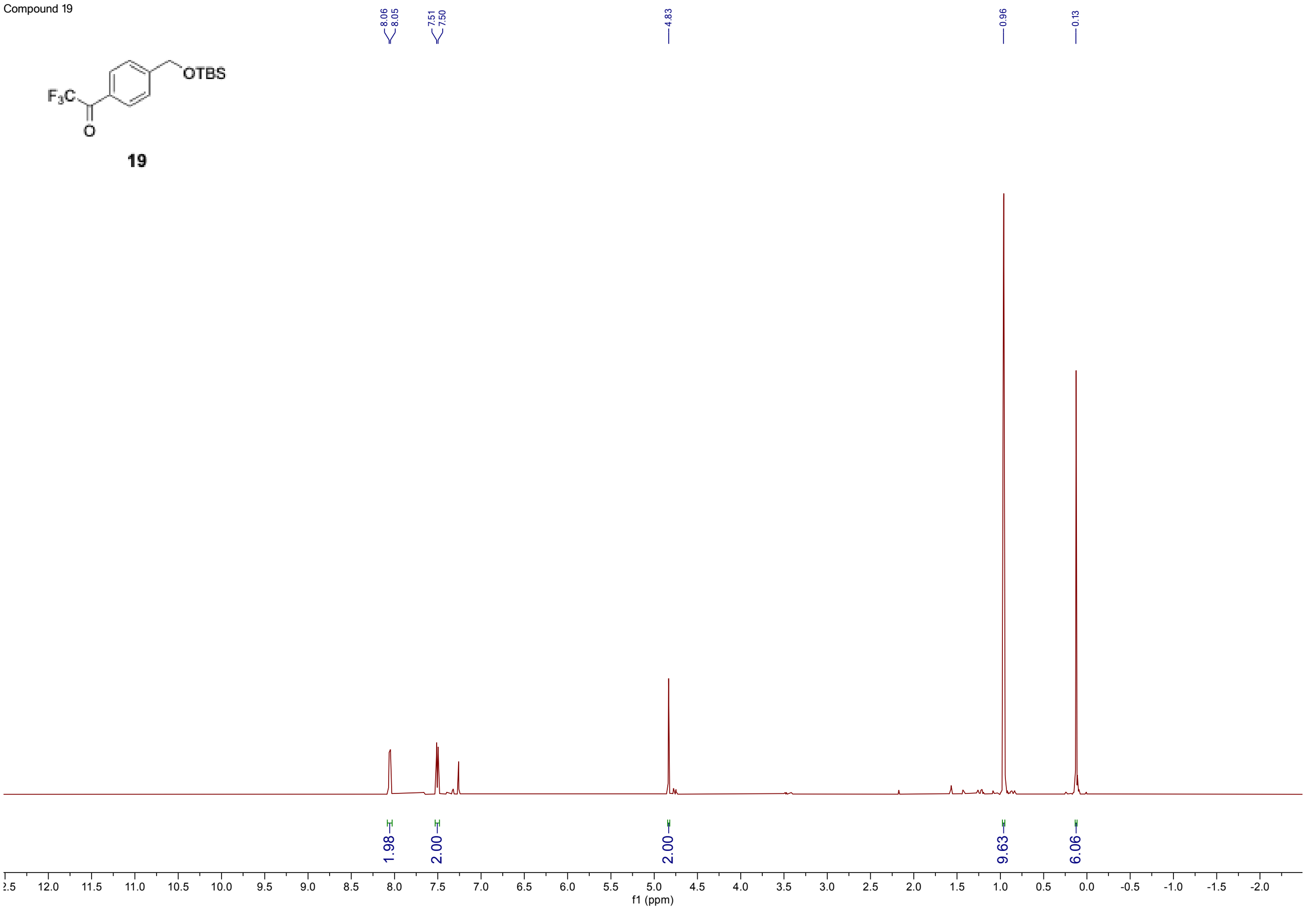
OTBS
F3C
O
19
8.06
8.05
7.51
7.50
4.83
0.96
0.13
1.98
2.00
2.00
9.63
6.06
12.0
11.5
11.0
10.5
10.0
9.5
9.0
8.5
8.0
7.5
7.0
6.5
6.0
5.5
5.0
4.5
4.0
3.5
3.0
2.5
2.0
1.5
1.0
0.5
0.0
-0.5
-1.0
-1.5
-2.0
f1 (ppm)

Compound 19

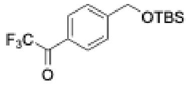

19

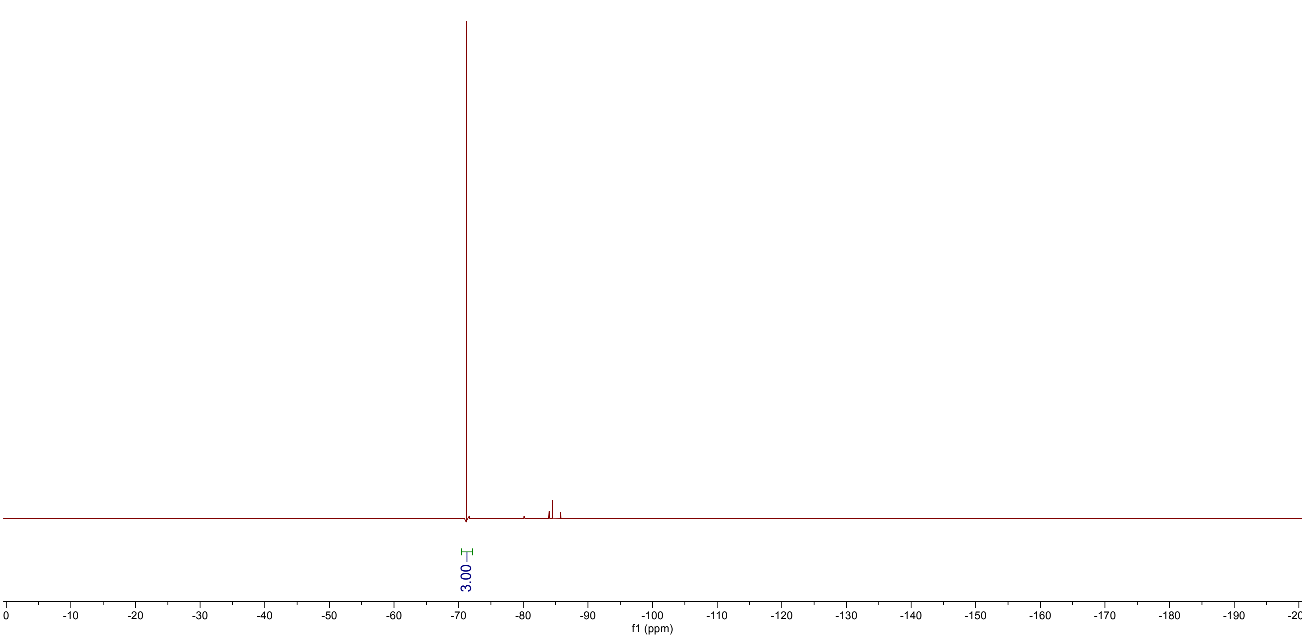

2050

Compound 20

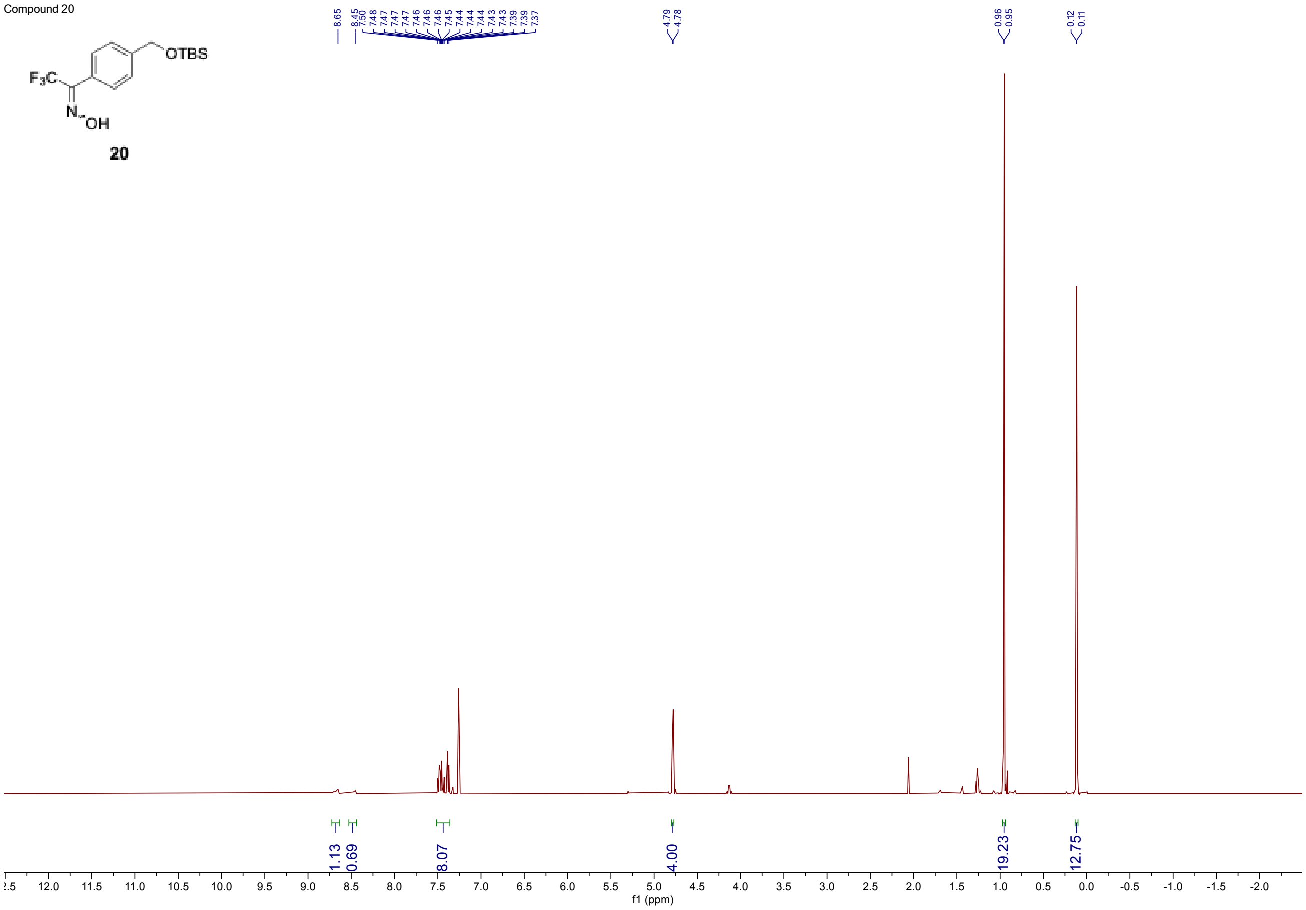
OTBS
F3C
N
OH
20
8.65
8.45
7.50
7.48
7.47
7.47
7.46
7.46
7.46
7.45
7.44
7.44
7.44
7.43
7.43
7.39
7.39
7.37
4.79
4.78
0.96
0.95
0.12
0.11
1.13
0.69
8.07
4.00
19.23
12.75
12.0
11.5
11.0
10.5
10.0
9.5
9.0
8.5
8.0
7.5
7.0
6.5
6.0
5.5
5.0
4.5
4.0
3.5
3.0
2.5
2.0
1.5
1.0
0.5
0.0
-0.5
-1.0
-1.5
-2.0
f1 (ppm)

Compound 20

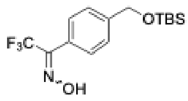

20

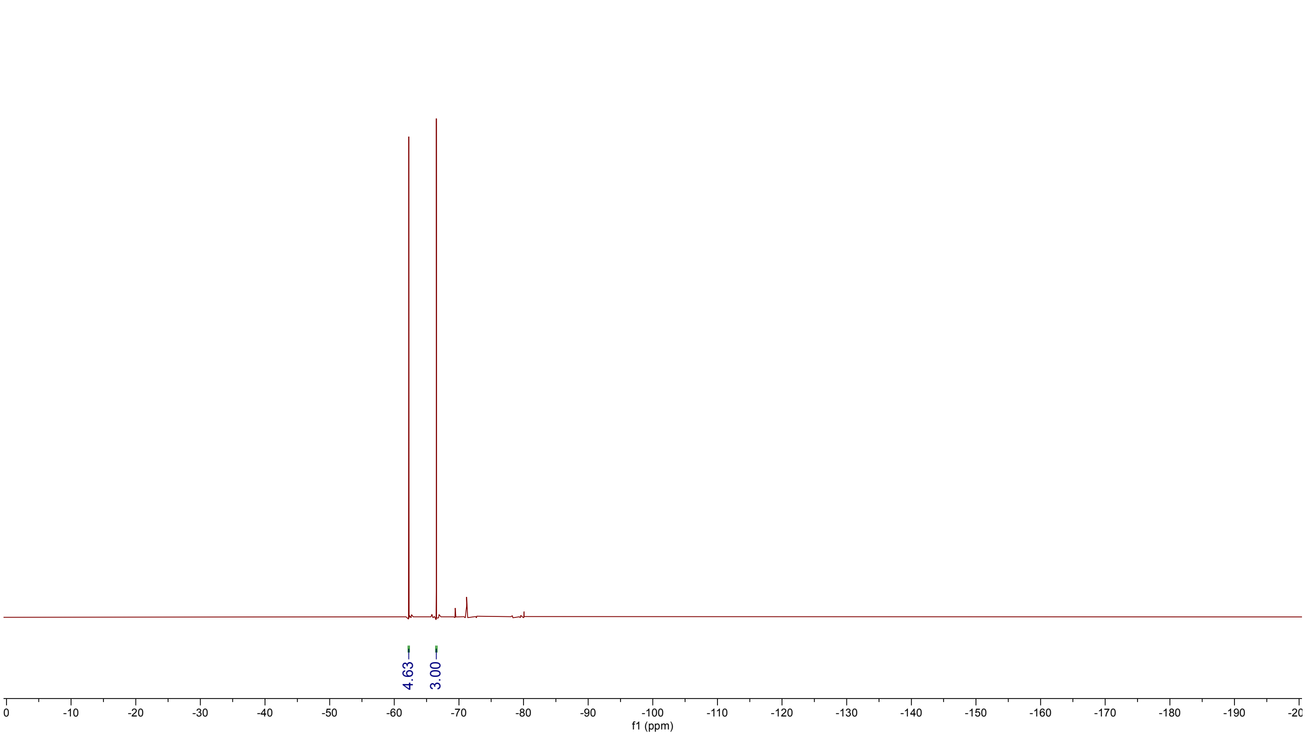

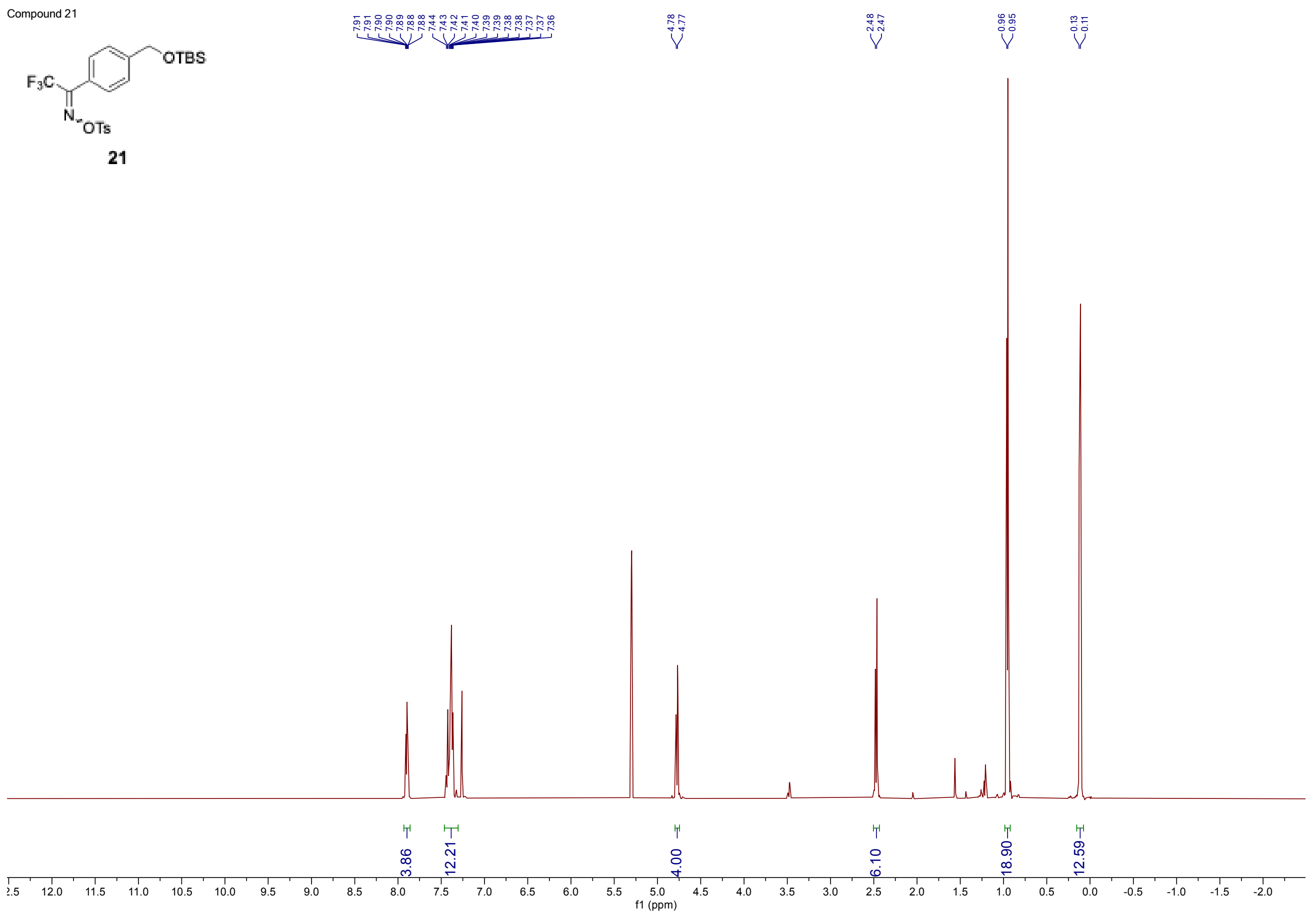

Compound 21
OTBS
F3C
N
OTs
21
7.91
7.91
7.90
7.89
7.88
7.88
7.44
7.43
7.42
7.41
7.40
7.39
7.39
7.38
7.38
7.37
7.37
7.36
4.78
4.77
2.48
2.47
0.96
0.95
0.13
0.11
3.86
12.21
4.00
6.10
18.90
12.59
f1 (ppm)

2055

Compound 21

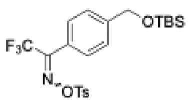

21

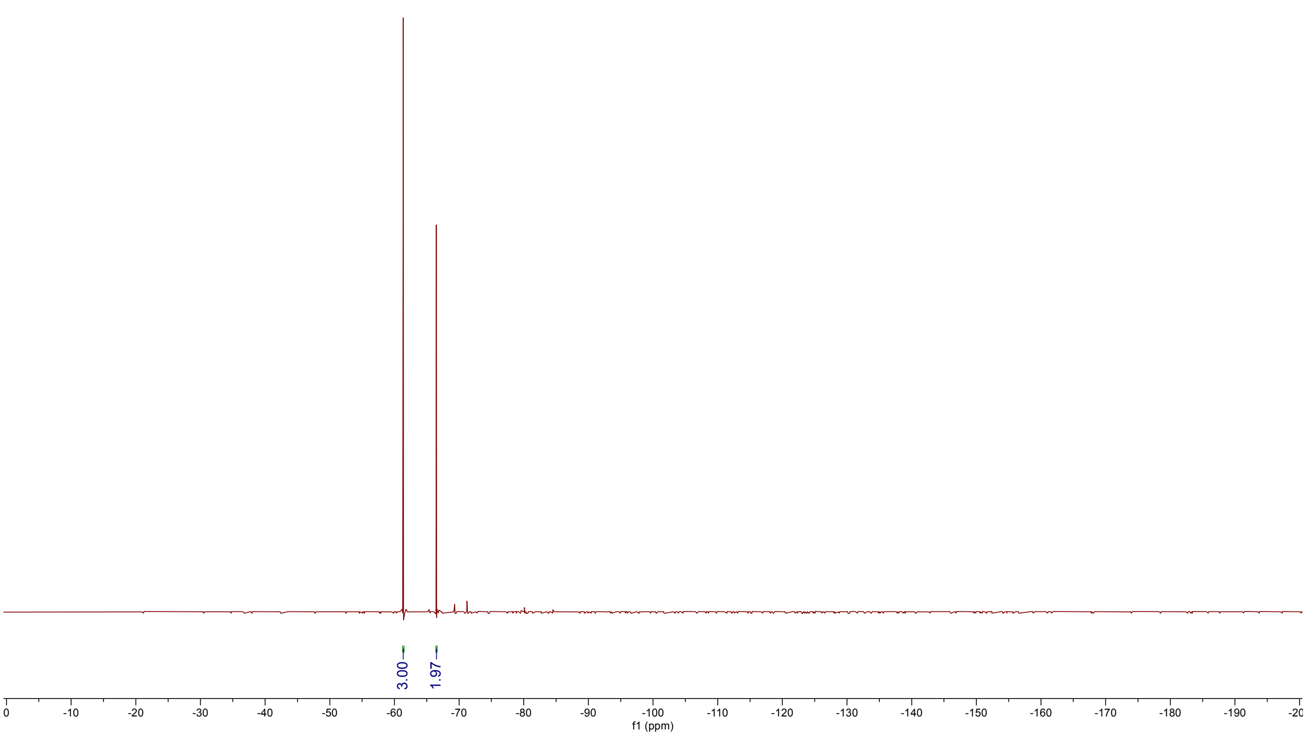

Compound 22

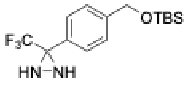

22

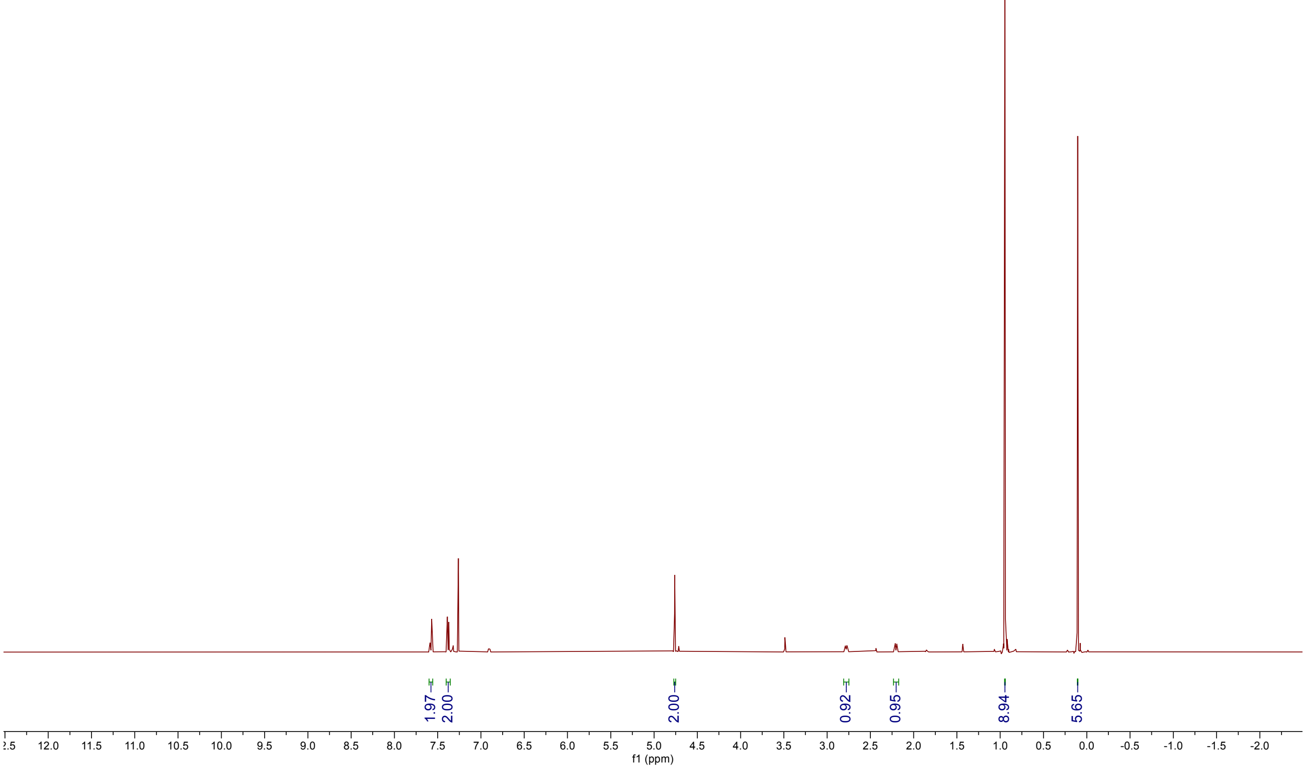

2060

Compound 22

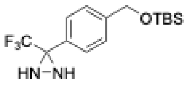

22

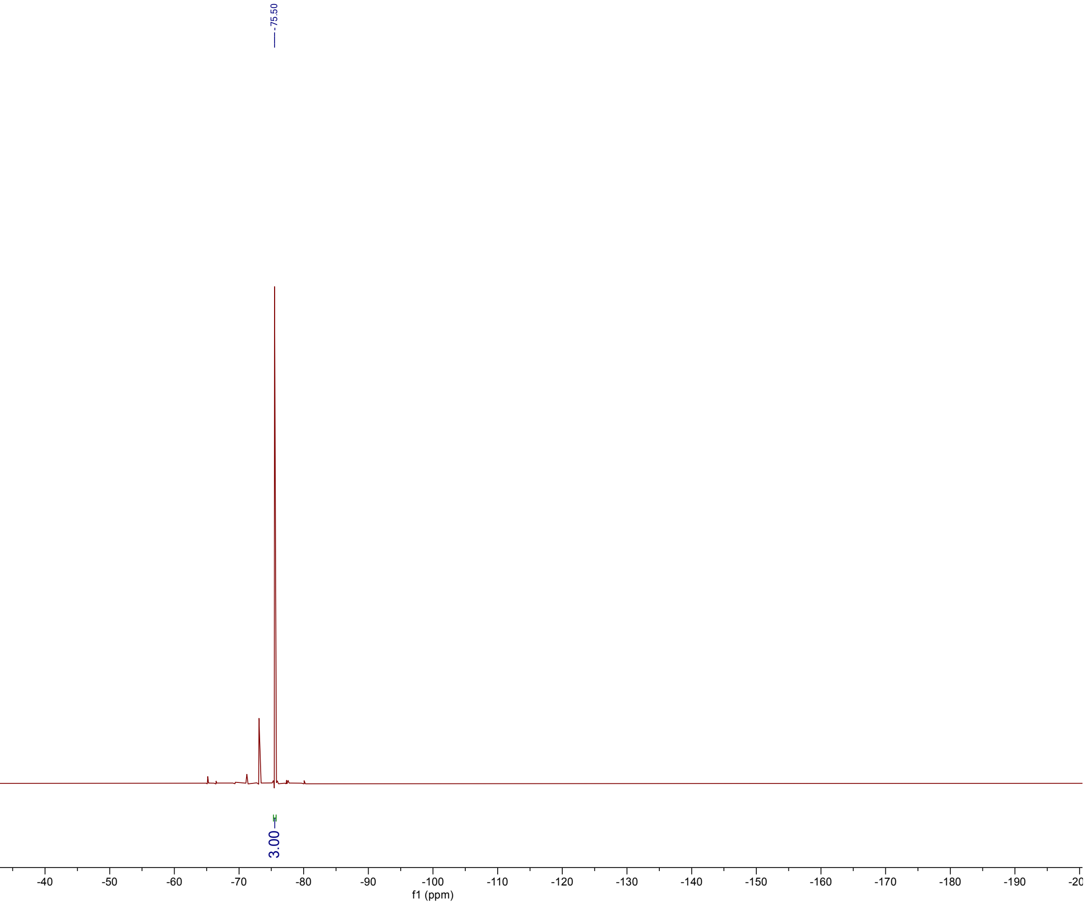

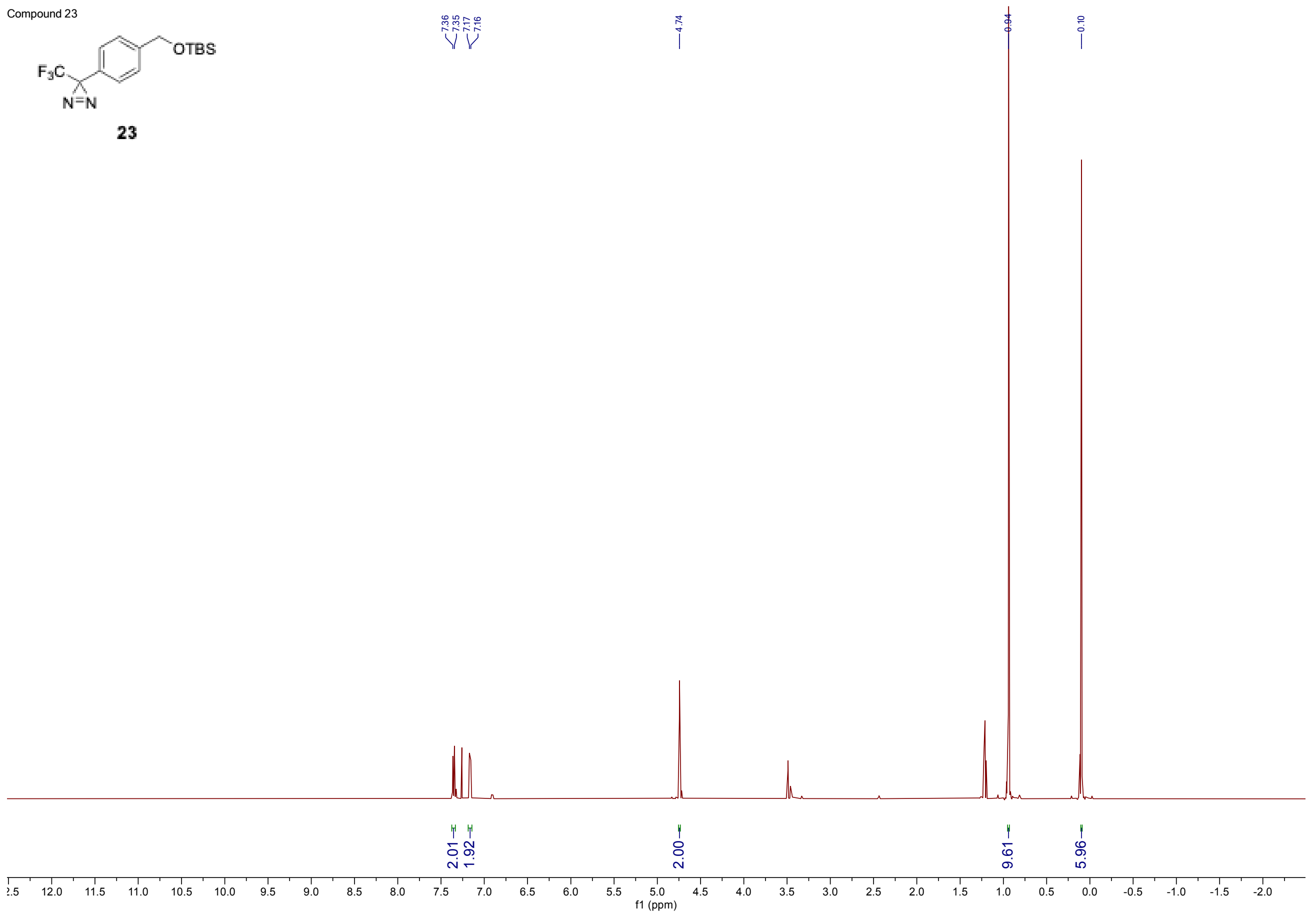
Compound 23
OTBS
F3C
N=N
23
7.36
7.35
7.17
7.16
4.74
0.94
0.10
2.01
1.92
2.00
9.61
5.96
f1 (ppm)

Compound 23

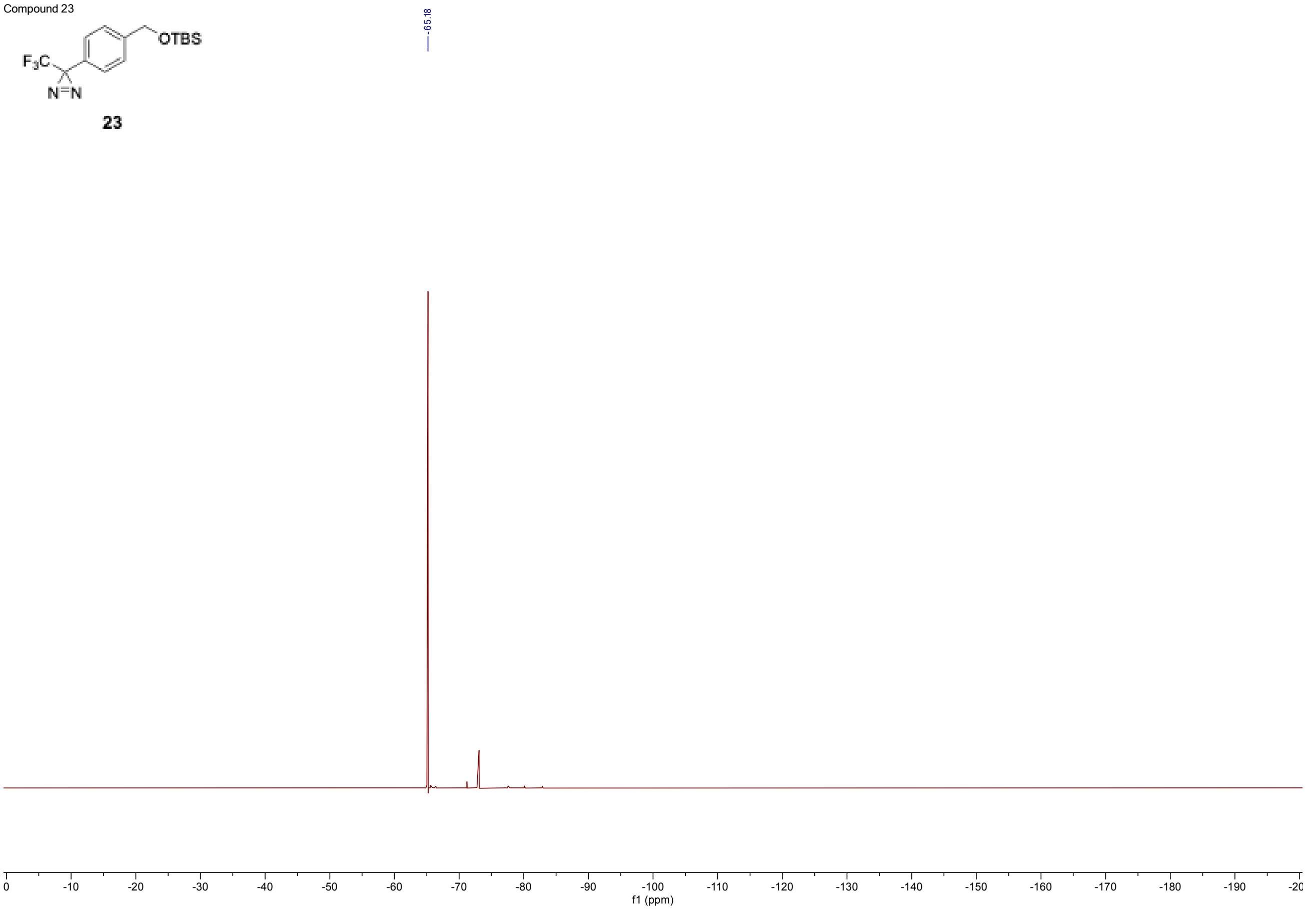

2065

Compound 24

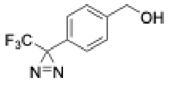

24

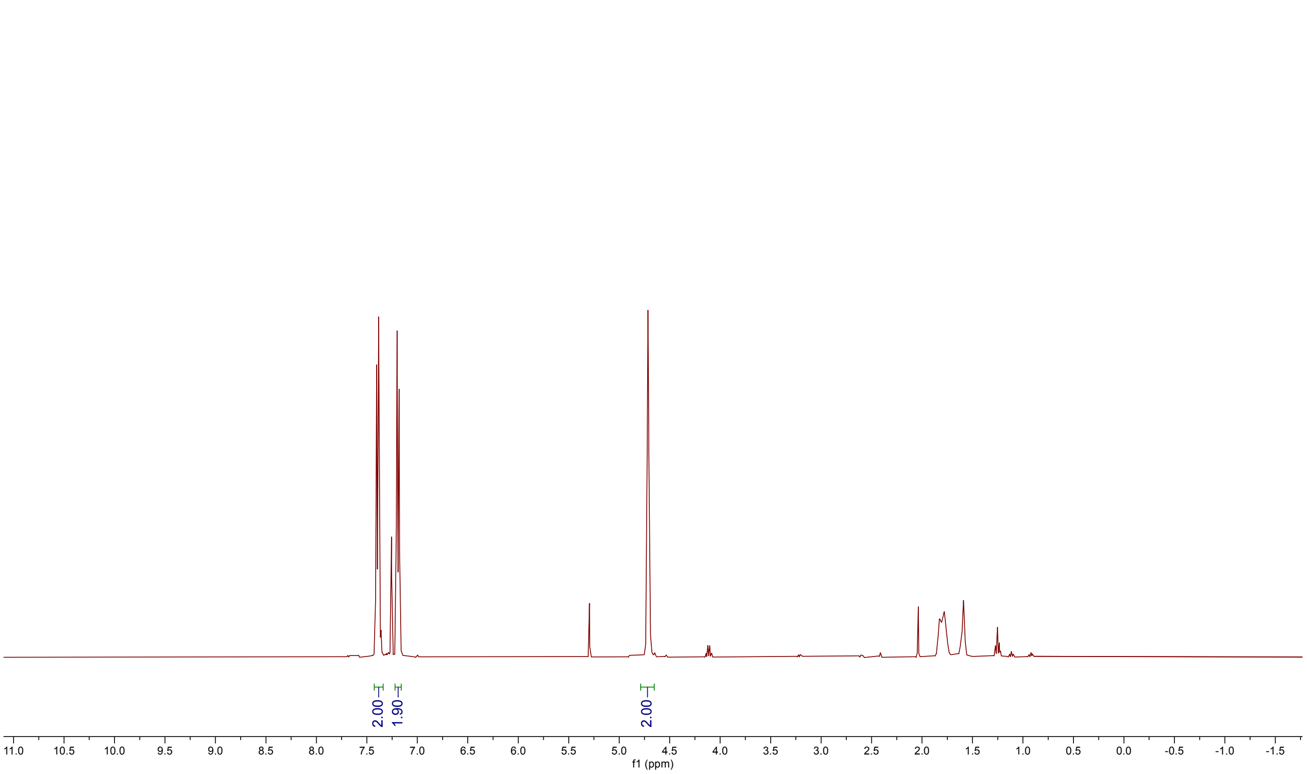

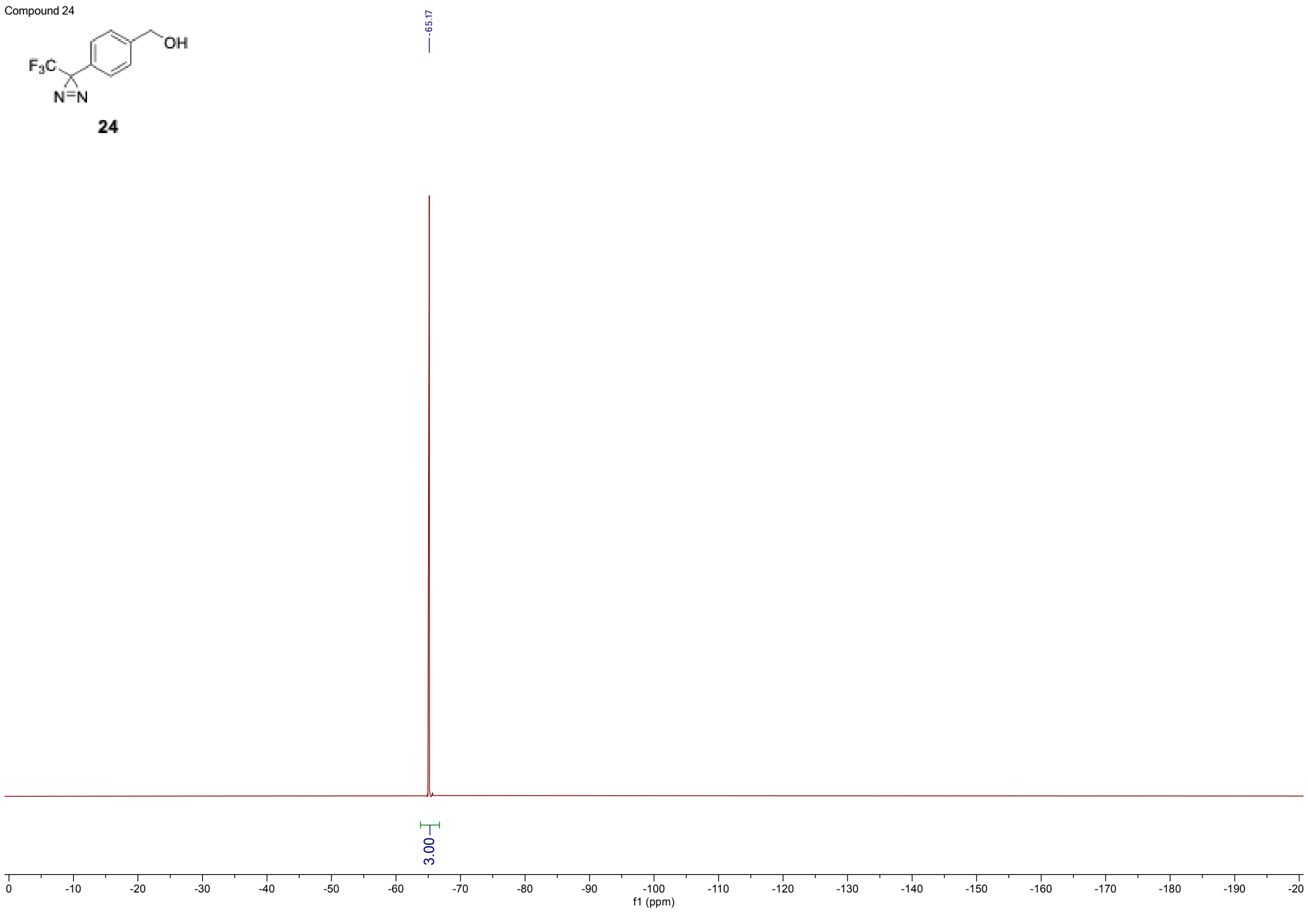
Compound 24
F3C
OH
N=N
24
-65.17
3.00
0
-10
-20
-30
-40
-50
-60
-70
-80
-90
-100
-110
-120
-130
-140
-150
-160
-170
-180
-190
-20
f1 (ppm)

2070

Compound 25

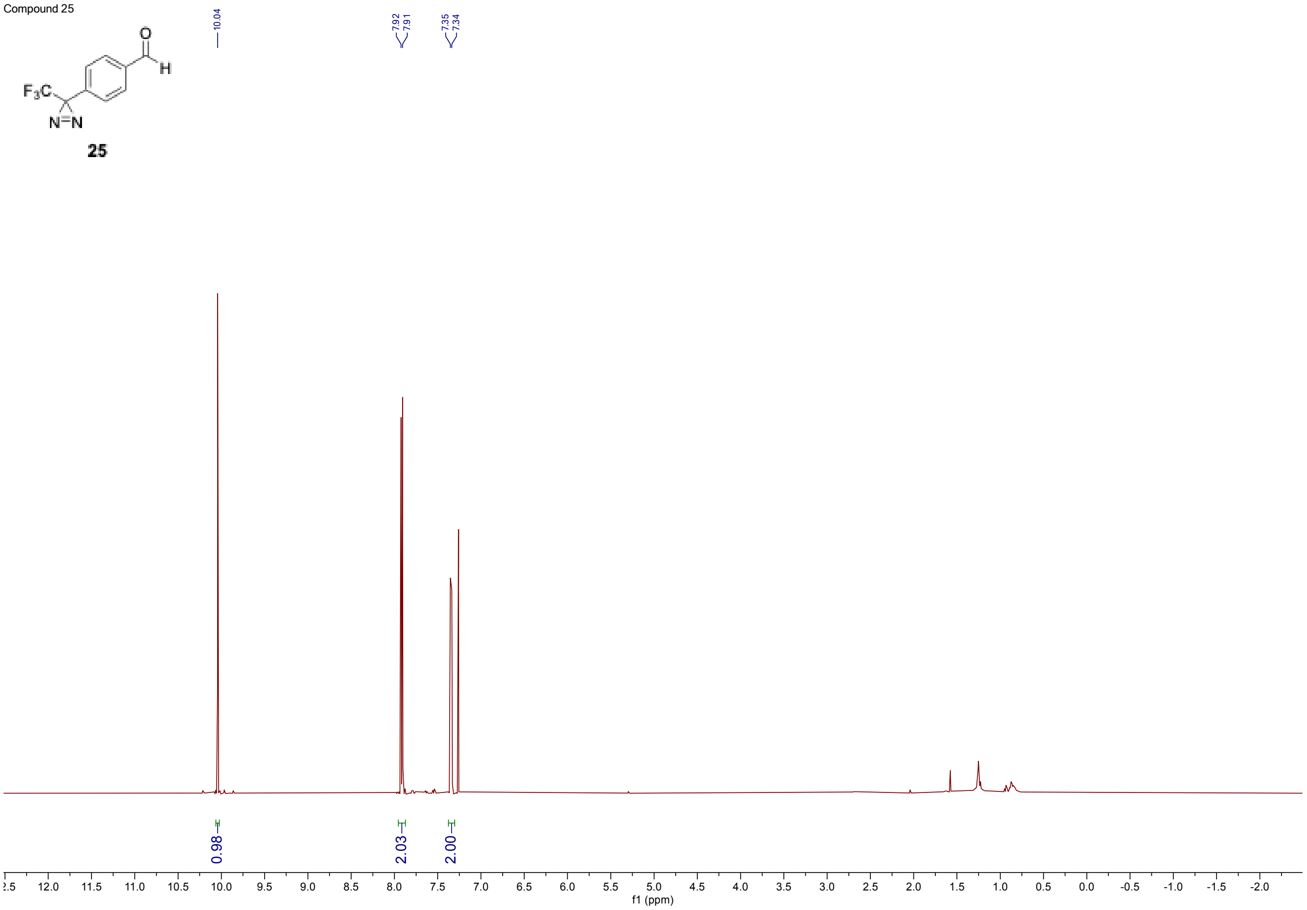

10.04
7.92
7.91
7.35
7.34
F_3C
N=N
O
H
25
0.98
2.03
2.00
12.0
11.5
11.0
10.5
10.0
9.5
9.0
8.5
8.0
7.5
7.0
6.5
6.0
5.5
5.0
4.5
4.0
3.5
3.0
2.5
2.0
1.5
1.0
0.5
0.0
-0.5
-1.0
-1.5
-2.0
f1 (ppm)

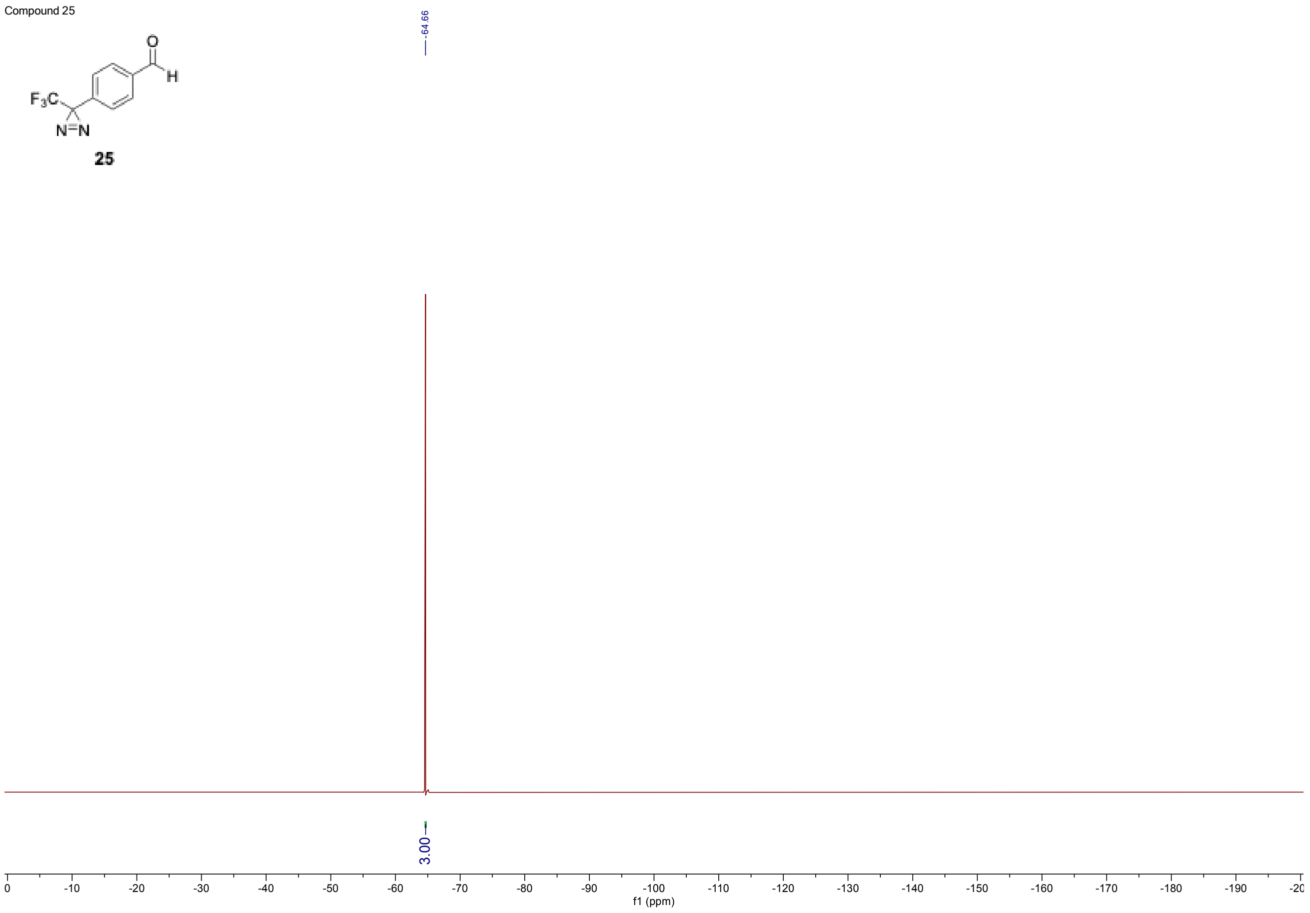
Compound 25
O
H
F3C
N=N
25
-64.66
3.00
0
-10
-20
-30
-40
-50
-60
-70
-80
-90
-100
-110
-120
-130
-140
-150
-160
-170
-180
-190
-20
f1 (ppm)

Compound 25

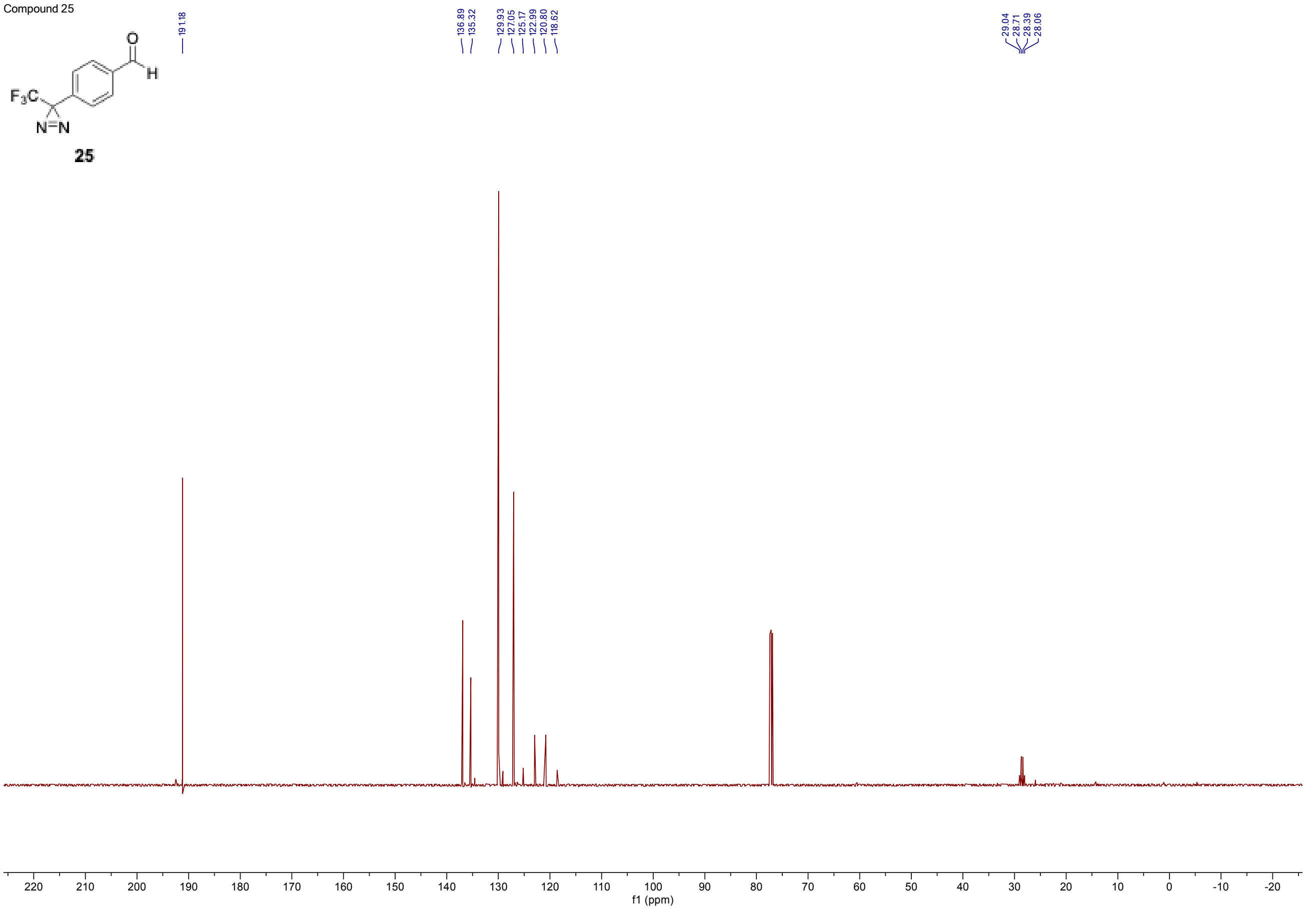

2075

Compound 11

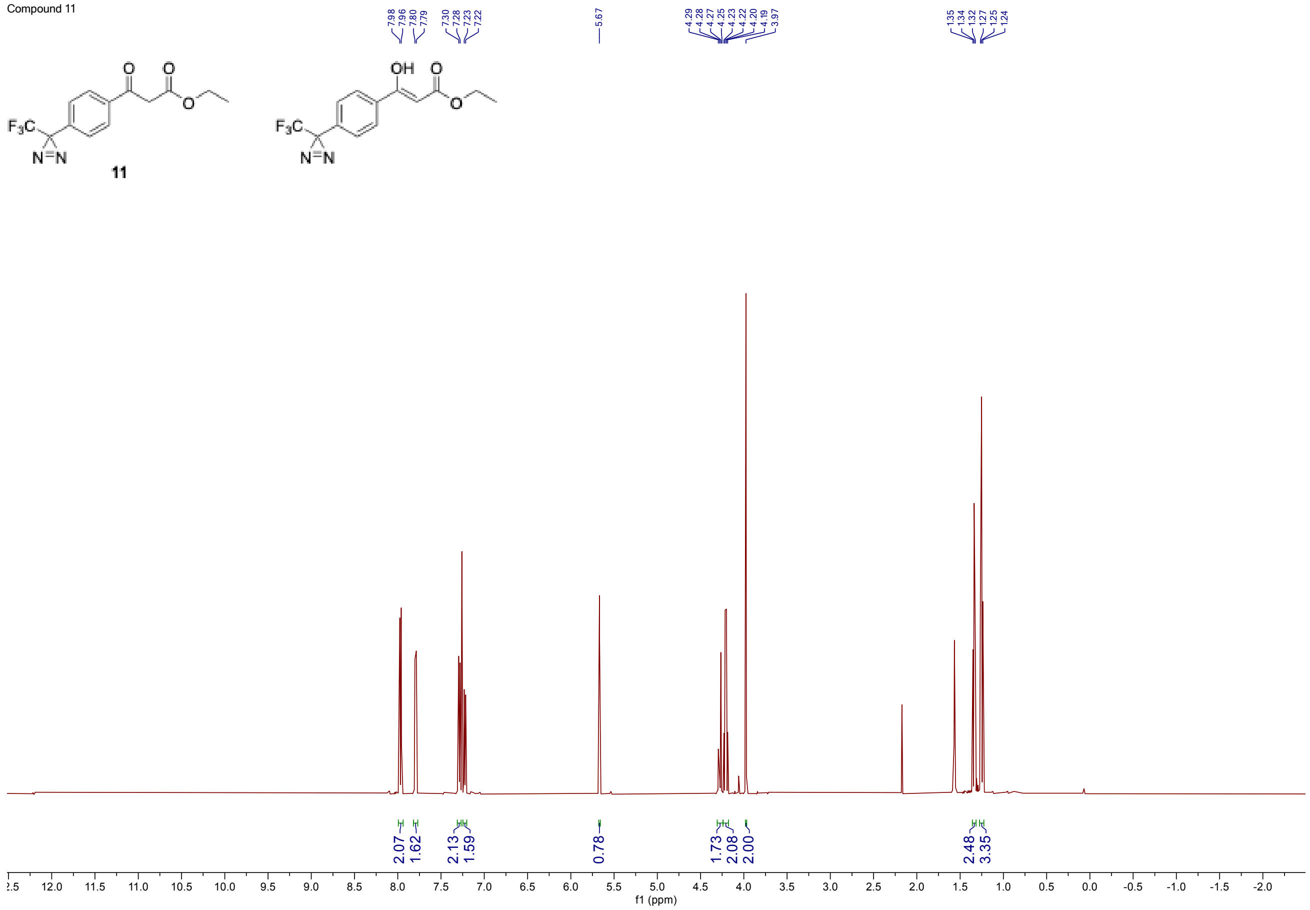

Compound 11

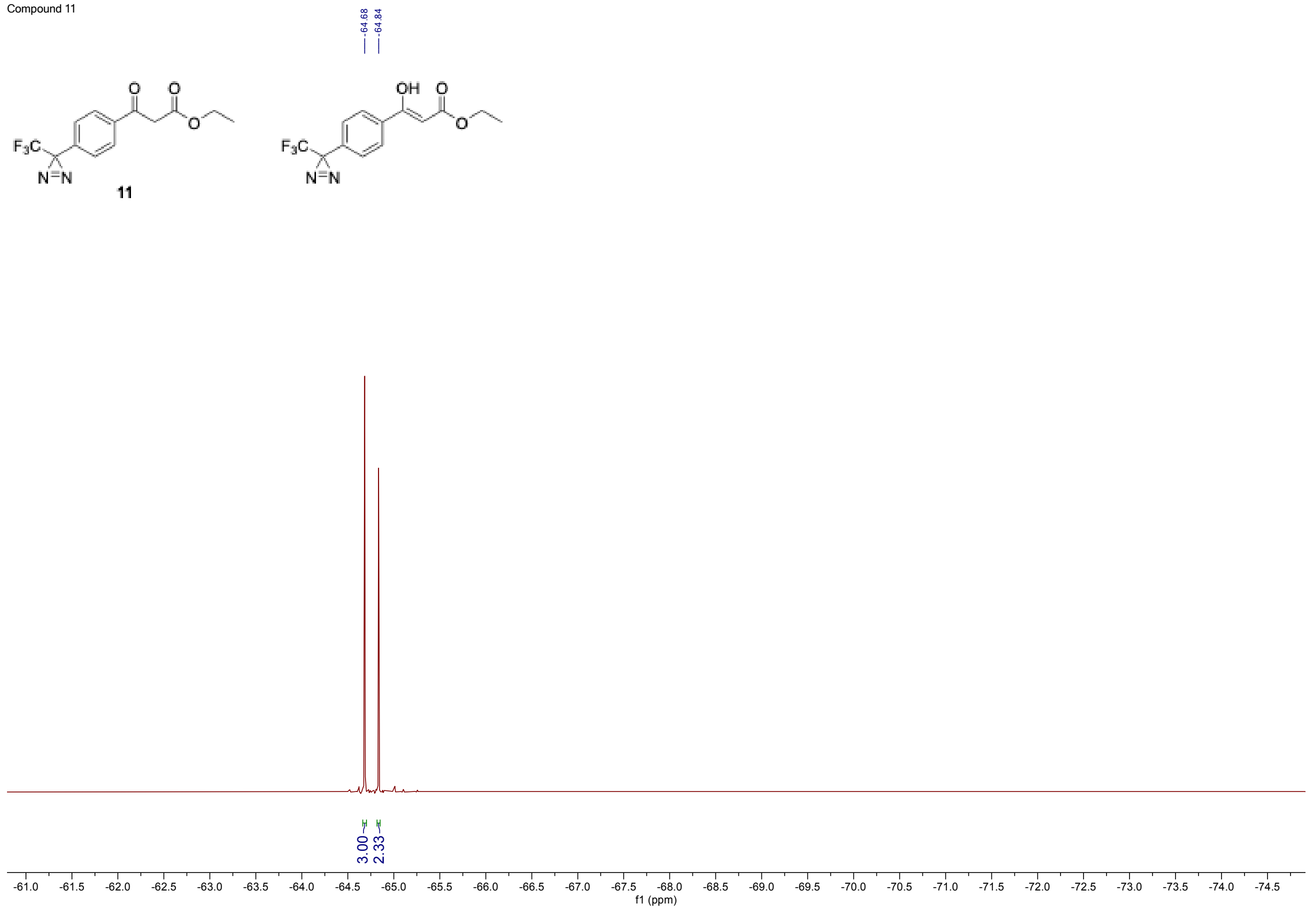

2080

Compound 11

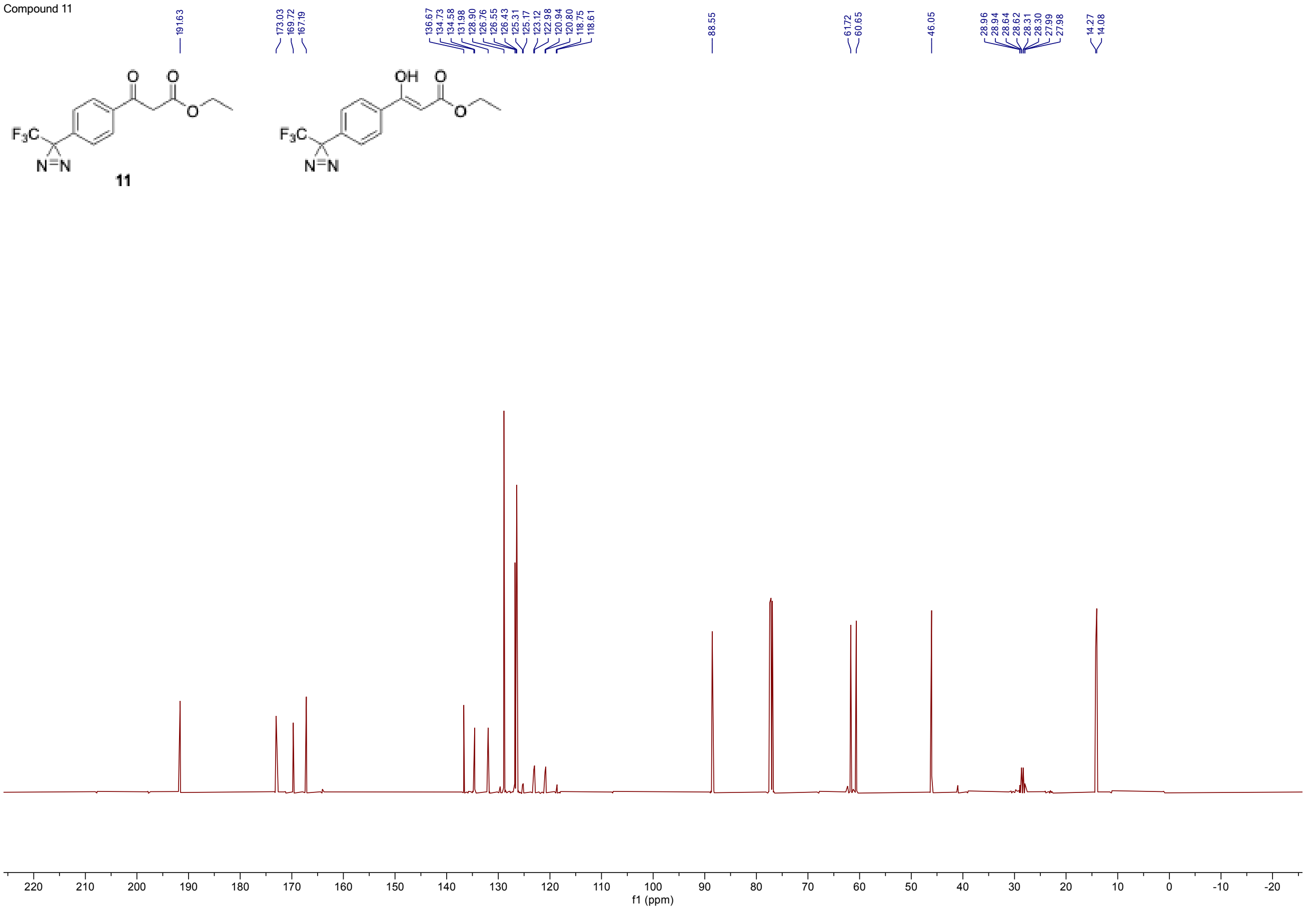
191.63
173.03
169.72
167.19
136.67
134.73
134.58
131.98
128.90
126.76
126.55
126.43
125.31
125.17
123.12
122.98
120.94
120.80
118.75
118.61
88.55
61.72
60.65
46.05
28.96
28.94
28.64
28.62
28.31
28.30
27.99
27.98
14.27
14.08
OH
O
F3C
N=N
11
220 210 200 190 180 170 160 150 140 130 120 110 100 90 80 70 60 50 40 30 20 10 0 -10 -20
f1 (ppm)

Compound S3

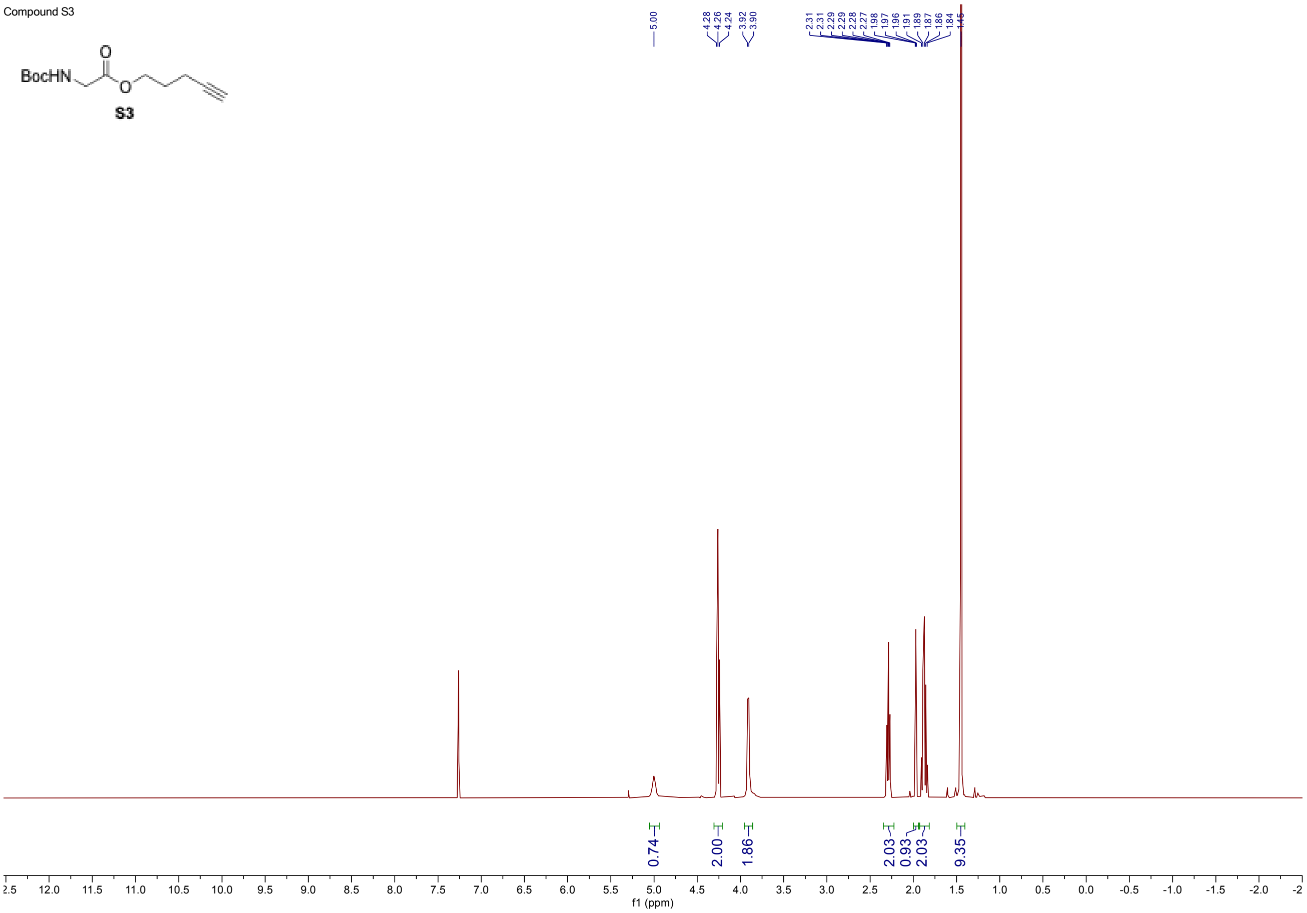

Compound S3

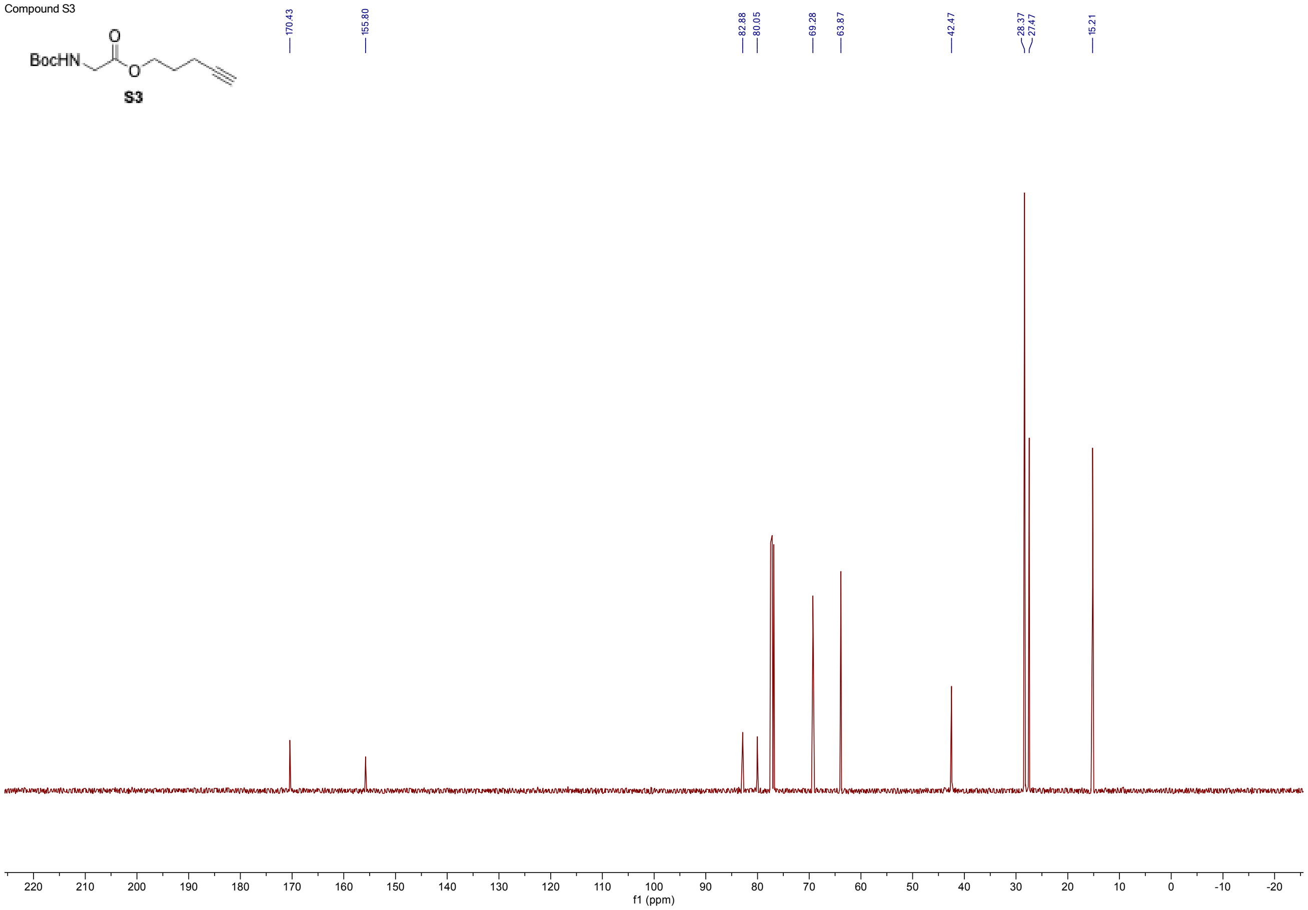

2085

Compound S4

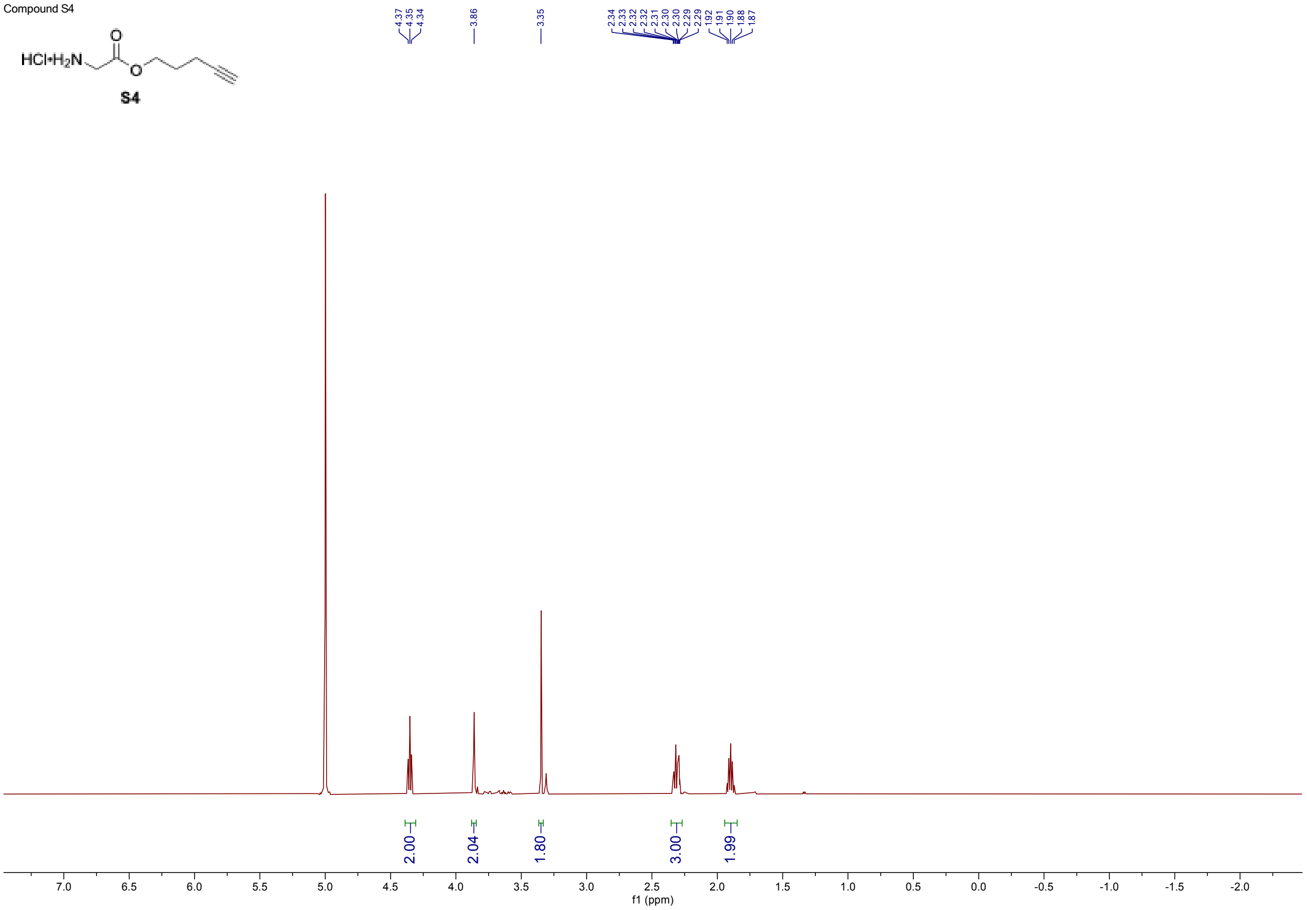
HCl•H2N
O
S4
4.37
4.35
4.34
3.86
3.35
2.34
2.33
2.32
2.32
2.31
2.30
2.30
2.29
2.29
1.92
1.91
1.90
1.88
1.87
2.00
2.04
1.80
3.00
1.99
7.0
6.5
6.0
5.5
5.0
4.5
4.0
3.5
3.0
2.5
2.0
1.5
1.0
0.5
0.0
-0.5
-1.0
-1.5
-2.0
f1 (ppm)

Compound S4

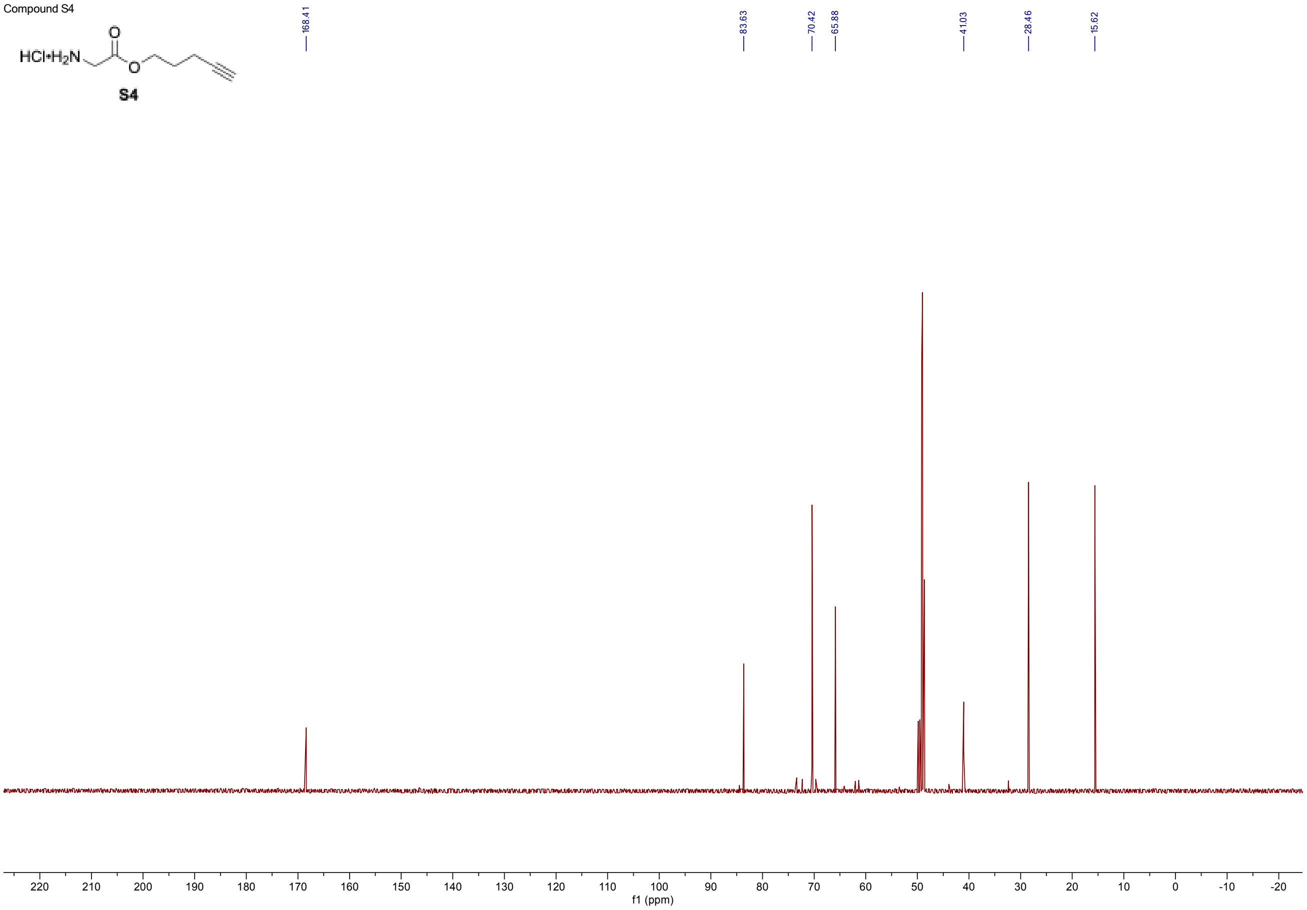

2090

Compound S5

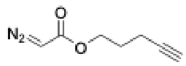

S5

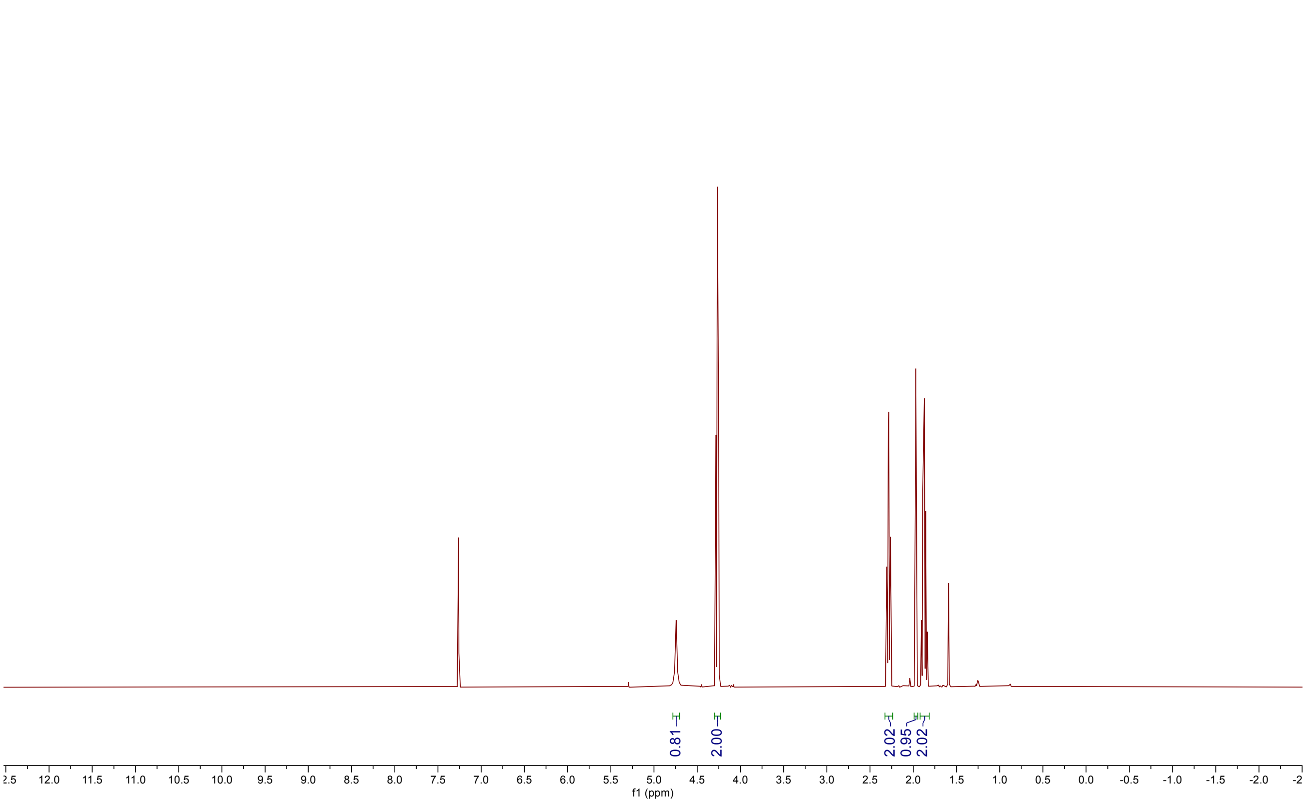

Compound S5

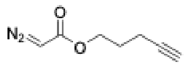

S5

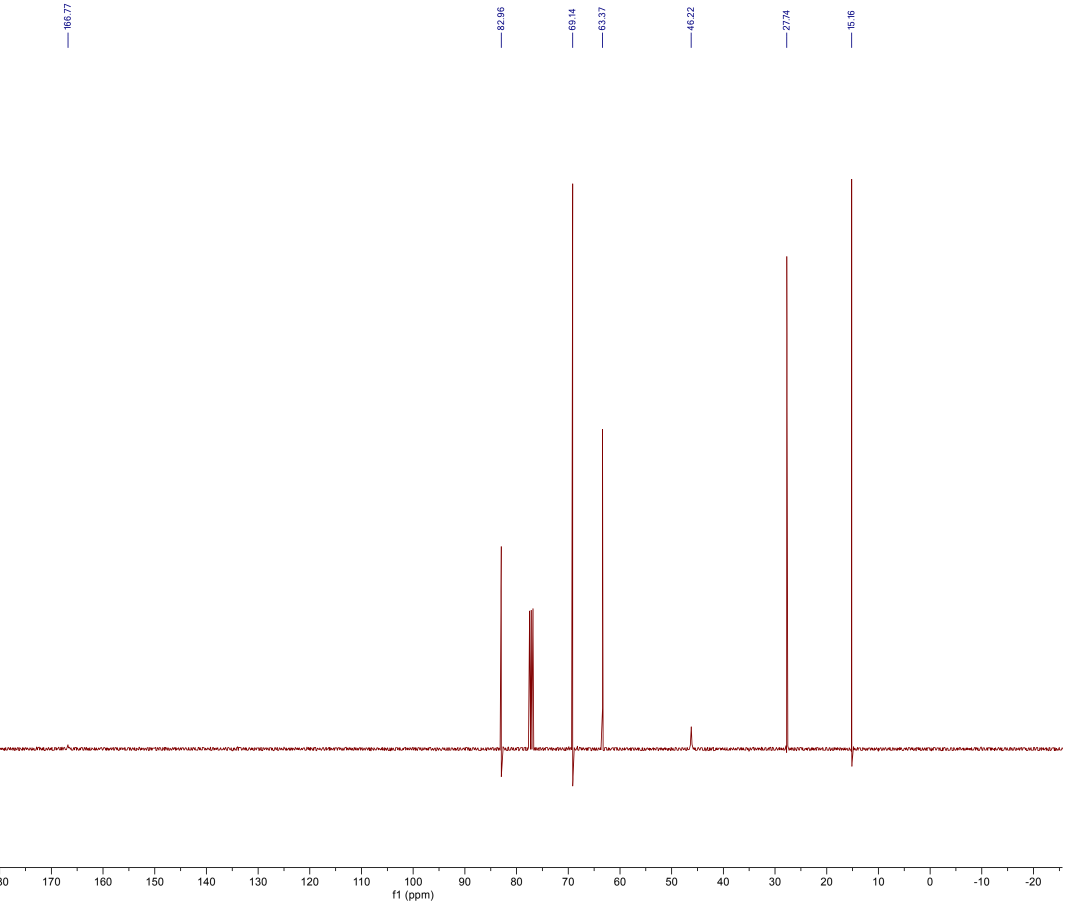

Compound S6

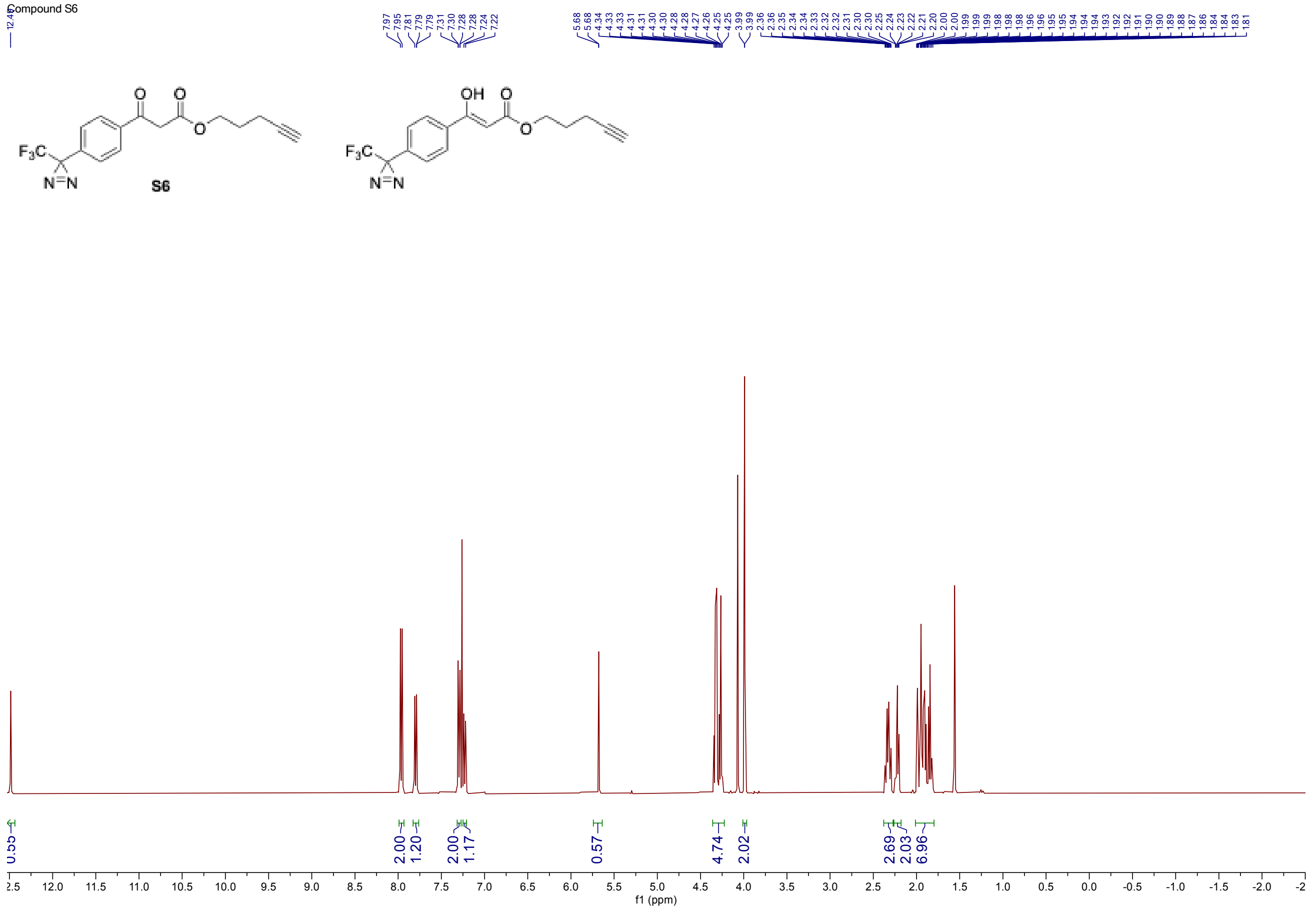

2095

Compound S6

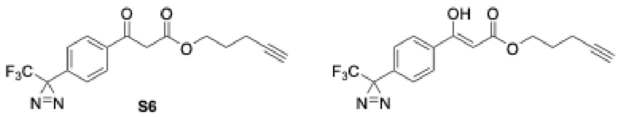

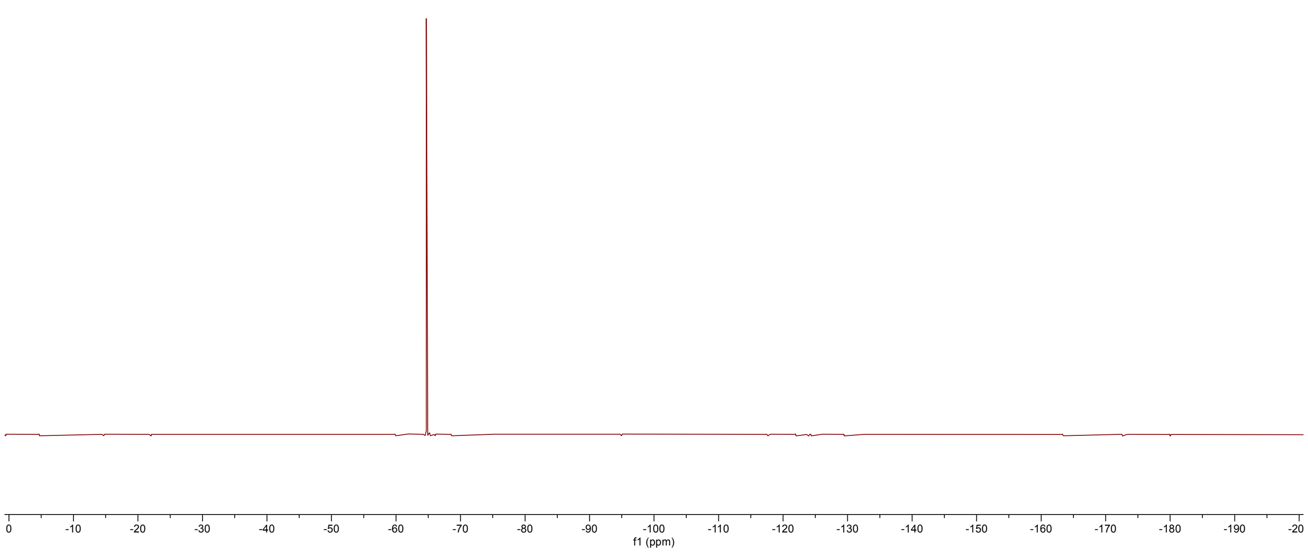

Compound S6

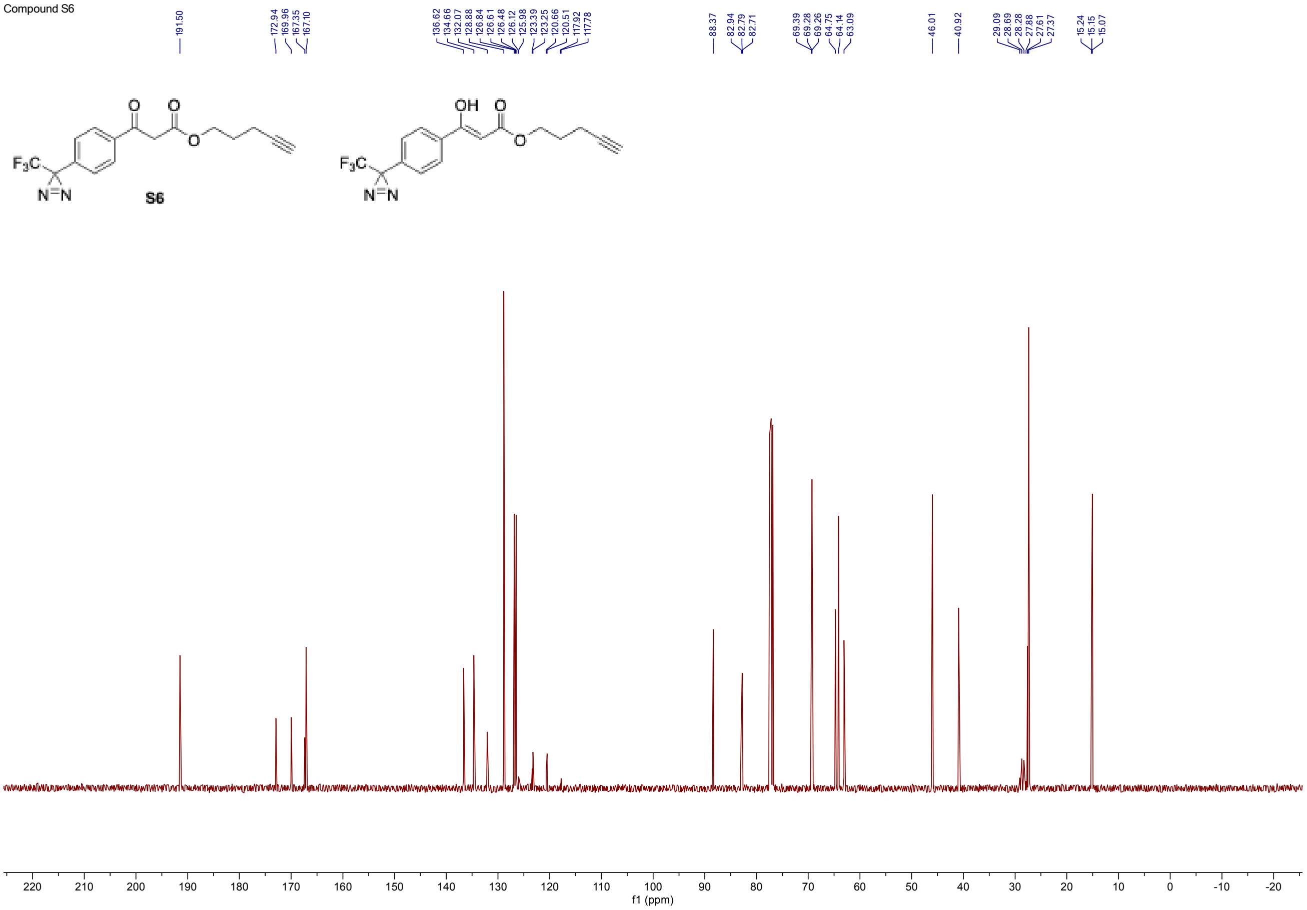

2100

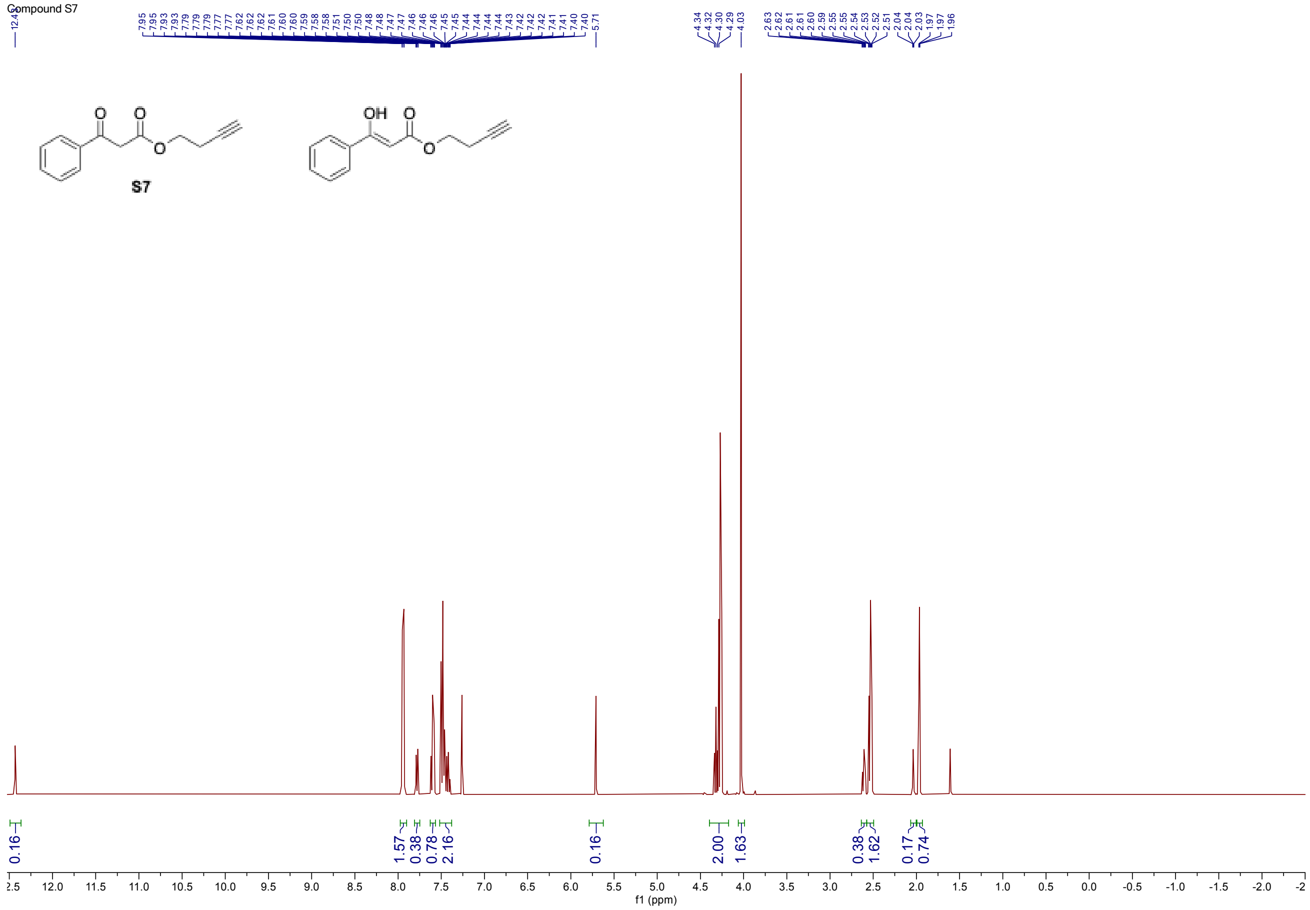
Compound S7
S7
f1 (ppm)

Compound S7

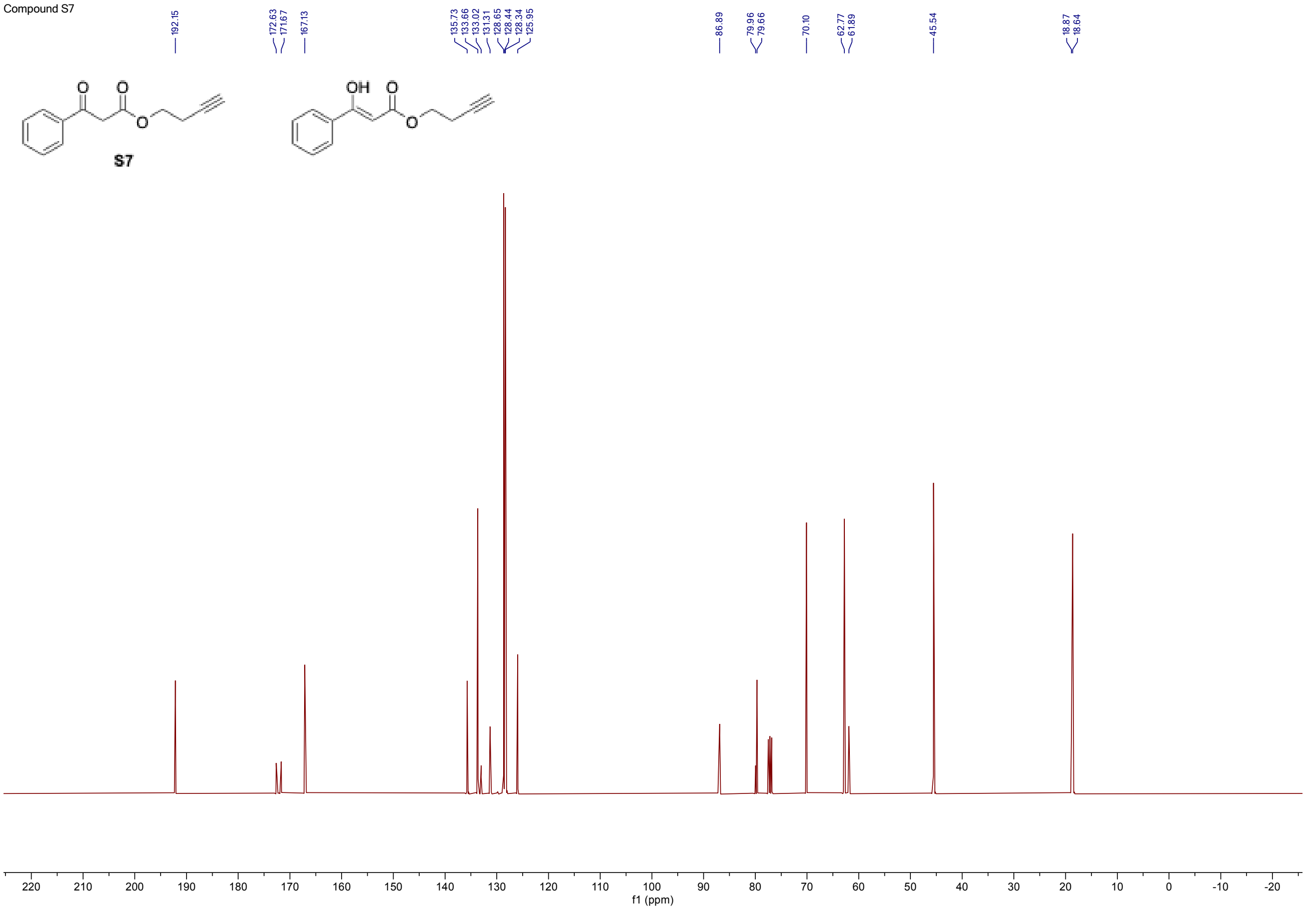
192.15
172.63
171.67
167.13
135.73
133.66
133.02
131.31
128.65
128.44
128.34
125.95
86.89
79.96
79.66
70.10
62.77
61.89
45.54
18.87
18.64
OH
S7
220
210
200
190
180
170
160
150
140
130
120
110
100
90
80
70
60
50
40
30
20
10
0
-10
-20
f1 (ppm)

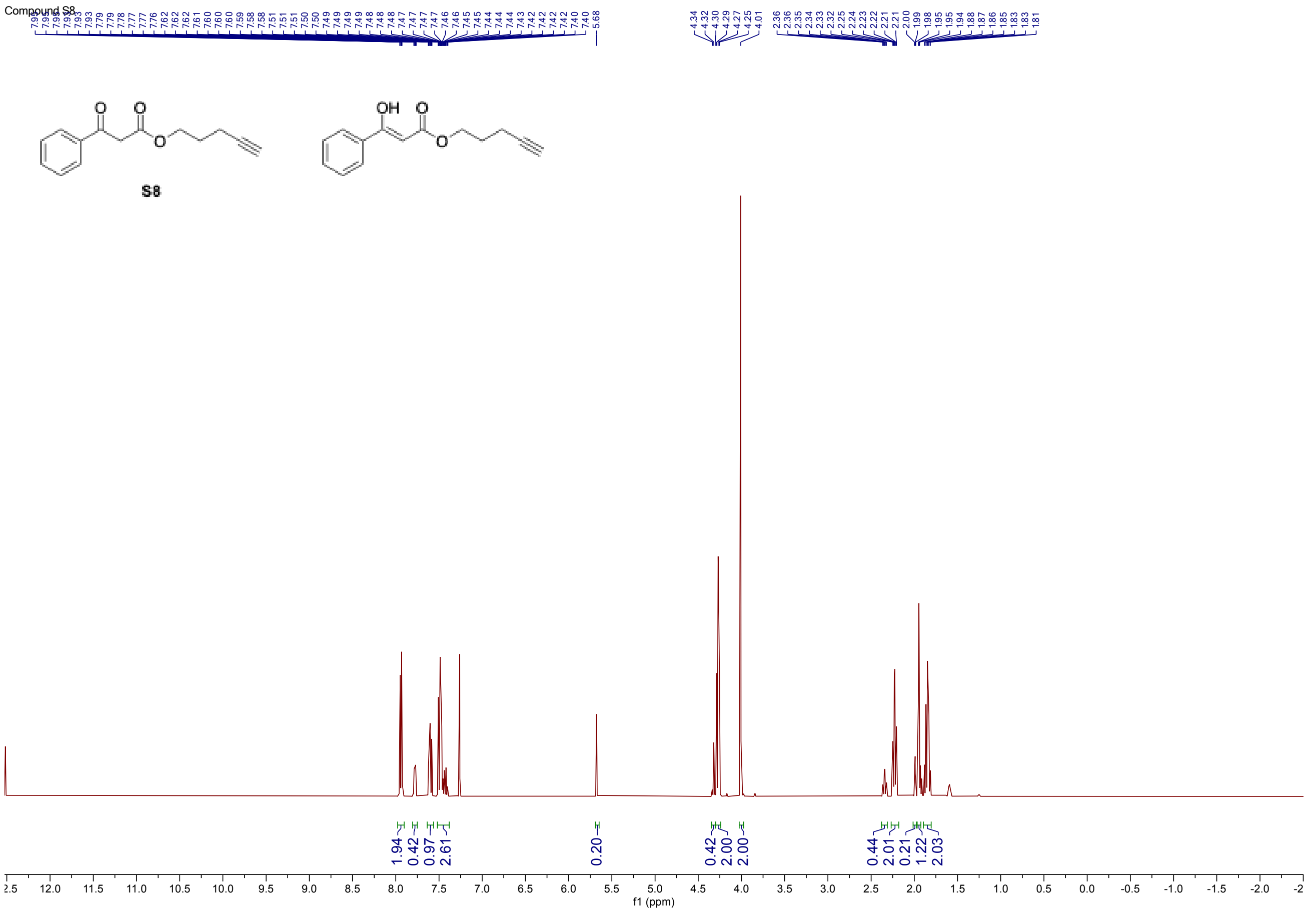
Compound S8
S8
OH
1.94 0.42 0.97 2.61
0.20
0.42 2.00 2.00
0.44 2.01 0.21 1.22 2.03
f1 (ppm)

2105

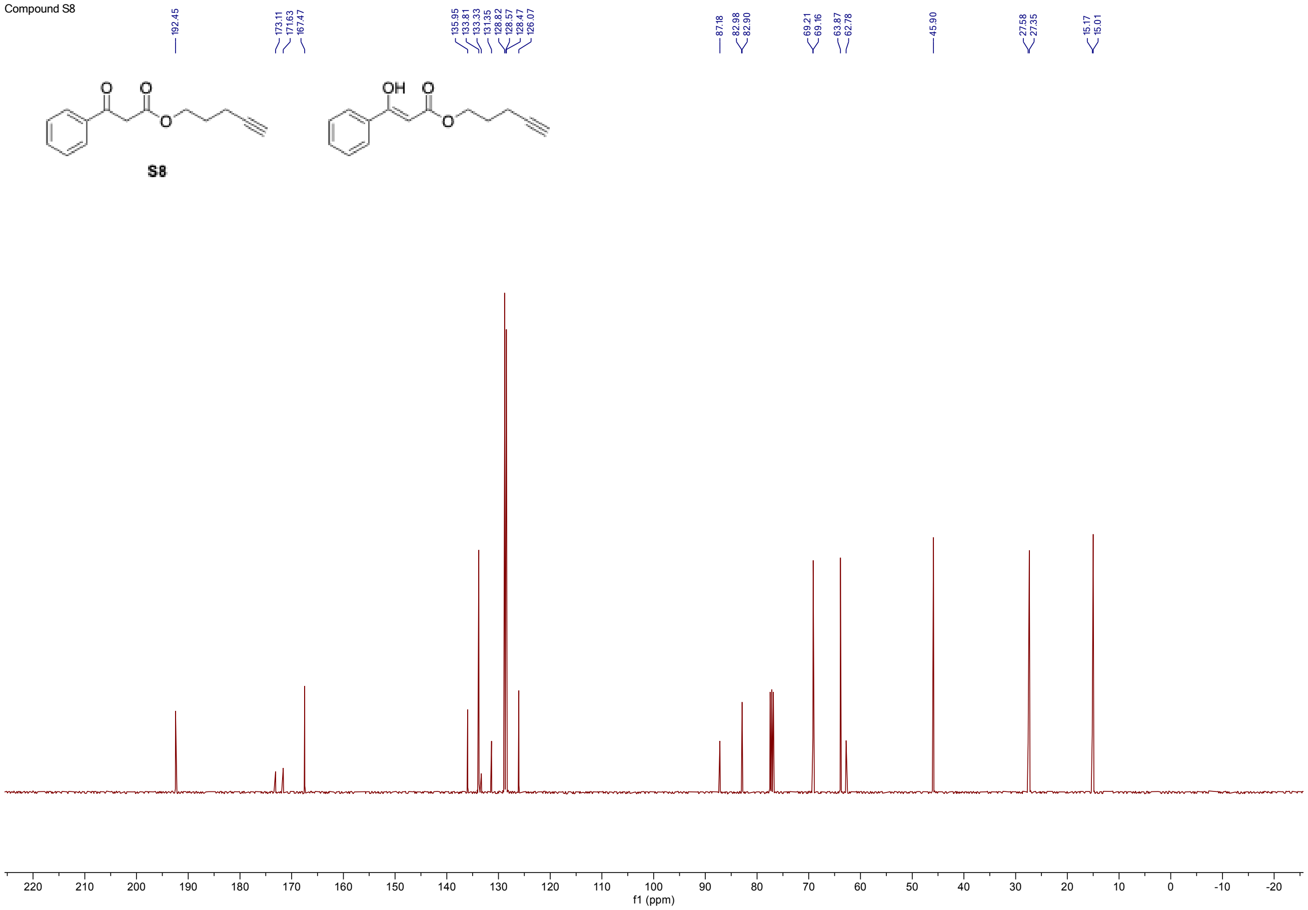
Compound S8
192.45
173.11
171.63
167.47
135.95
133.81
133.33
131.35
128.82
128.57
128.47
126.07
87.18
82.98
82.90
69.21
69.16
63.87
62.78
45.90
27.58
27.35
15.17
15.01
OH
S8
220 210 200 190 180 170 160 150 140 130 120 110 100 90 80 70 60 50 40 30 20 10 0 -10 -20
f1 (ppm)

Compound 26a

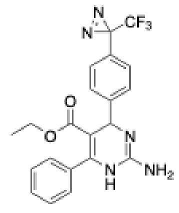

26a

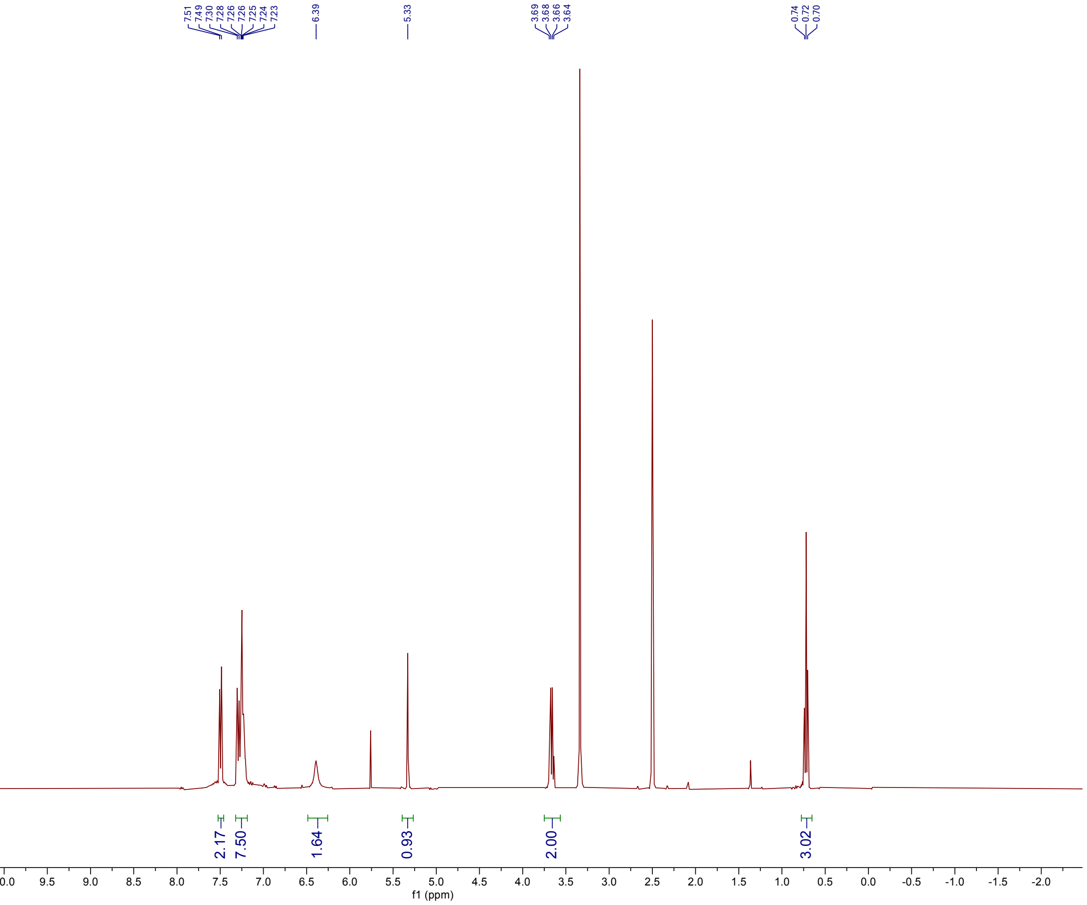

2110

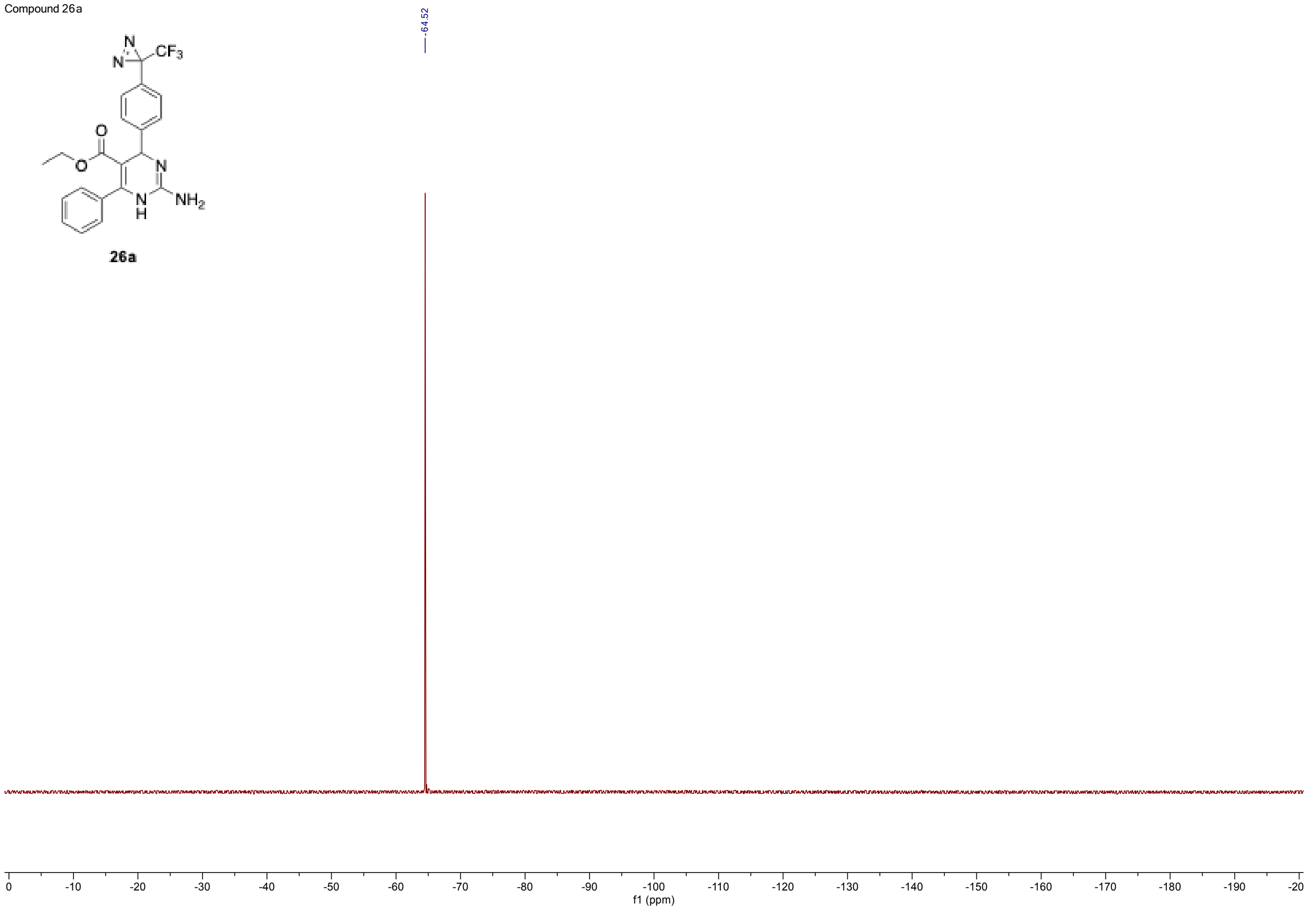
Compound 26a
-64.52
N
N
CF3
O
O
N
N
H
NH2
26a
0 -10 -20 -30 -40 -50 -60 -70 -80 -90 -100 -110 -120 -130 -140 -150 -160 -170 -180 -190 -20
f1 (ppm)

Compound 26a

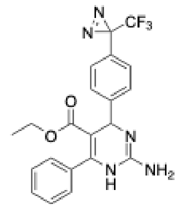

26a

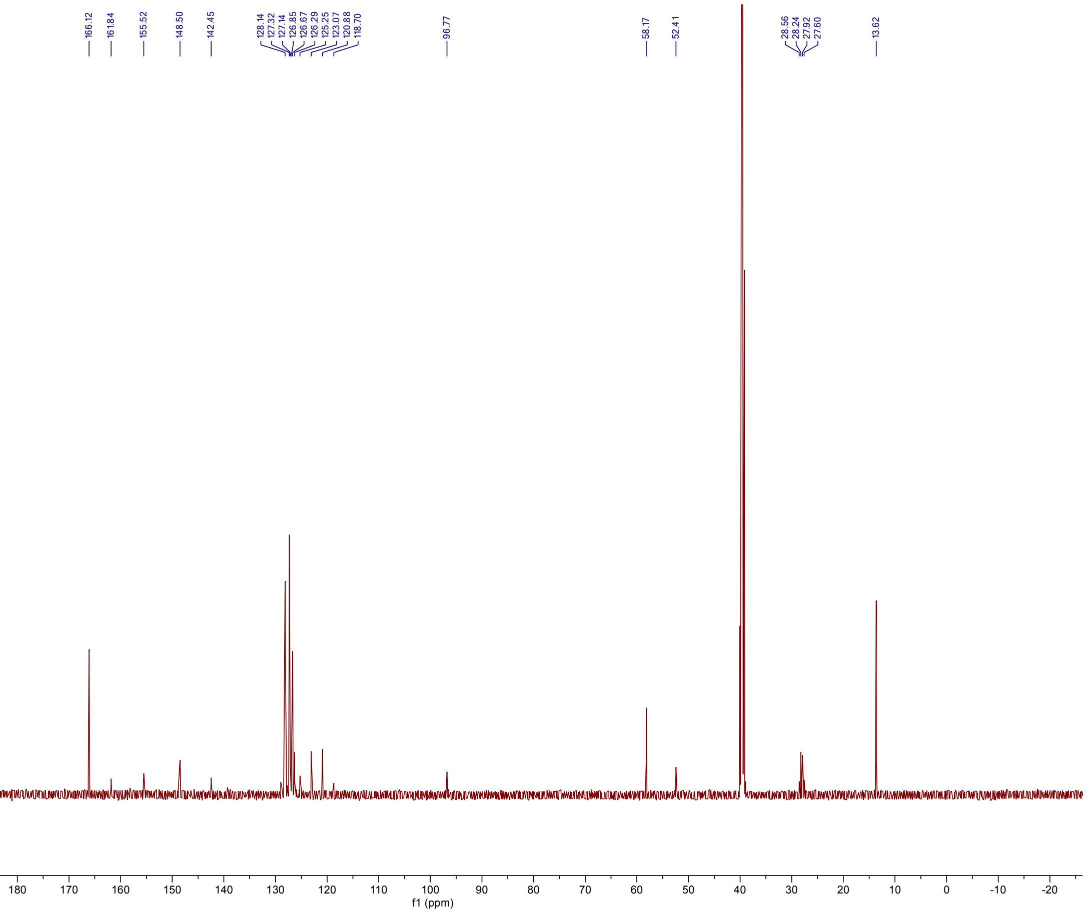

Compound 26b

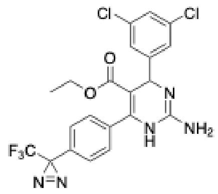

26b

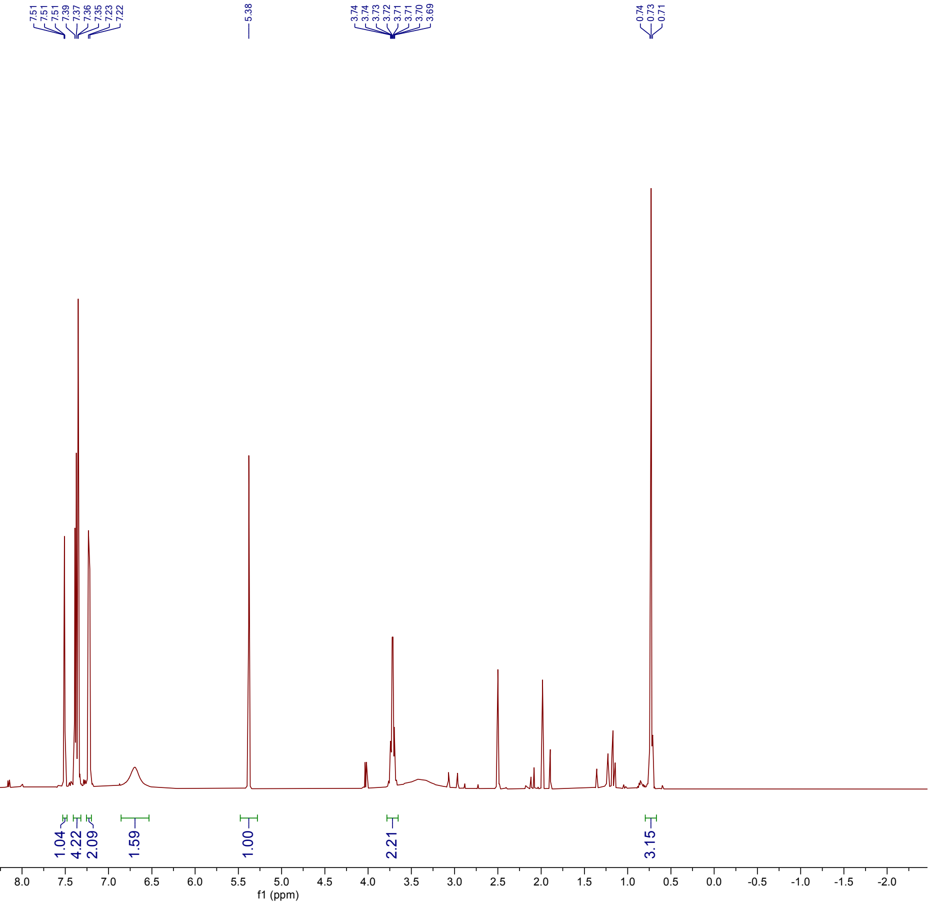

2115

Compound 26b

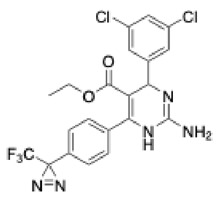

26b

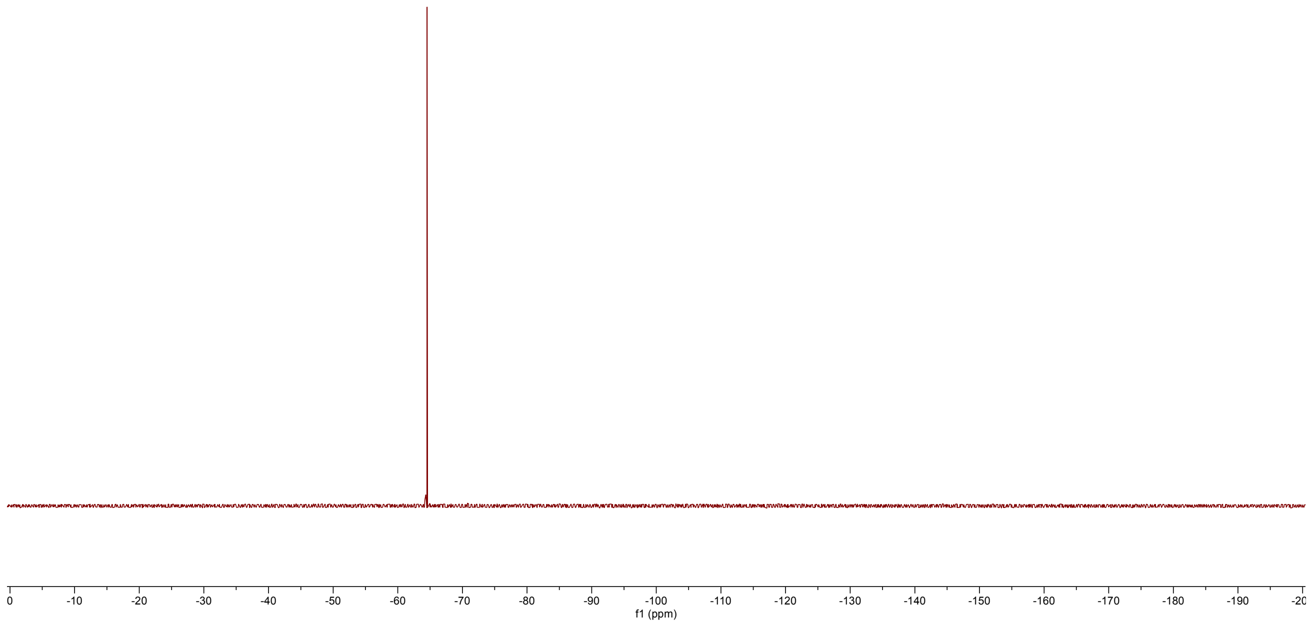

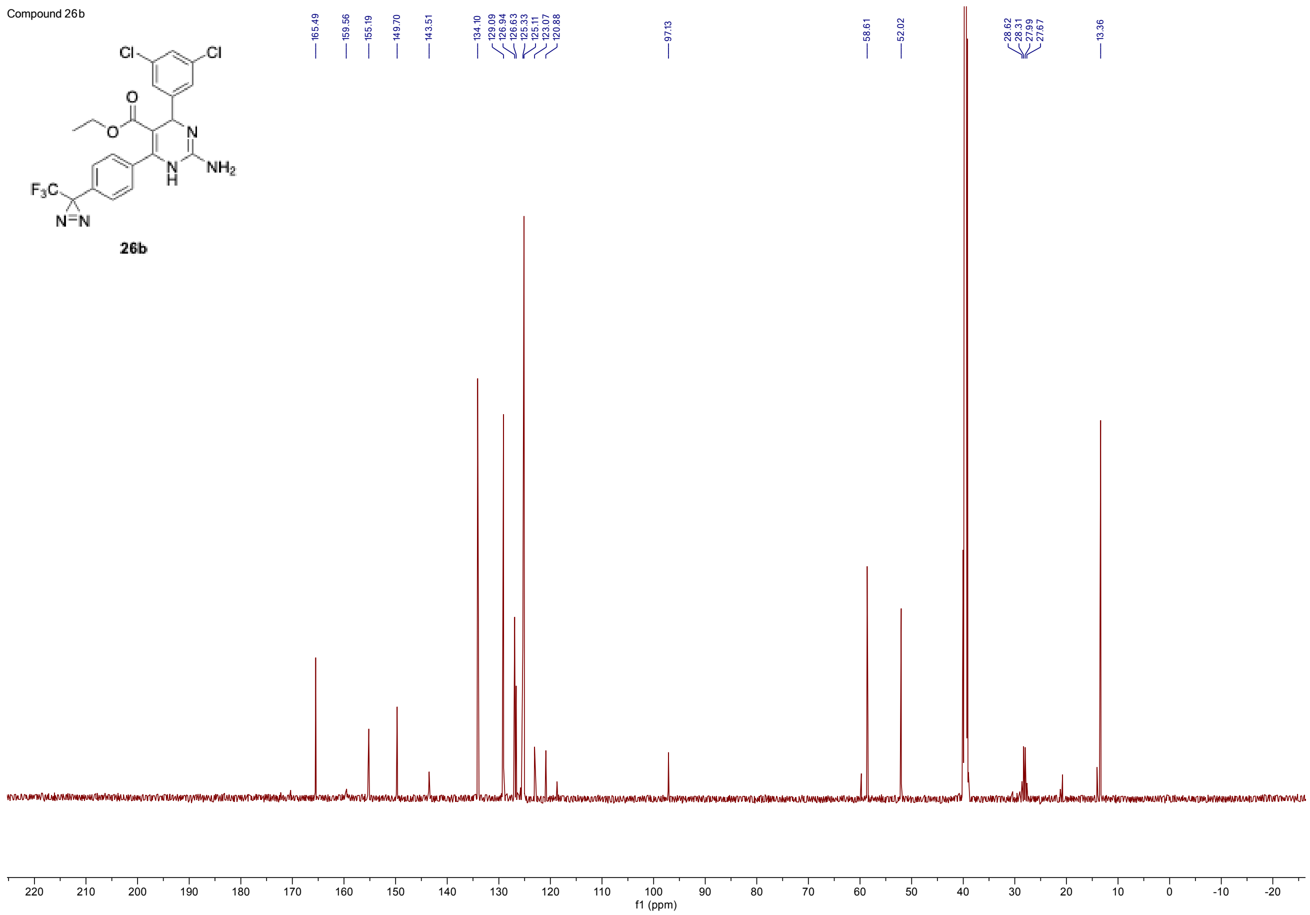
Compound 26b
26b
165.49
159.56
155.19
149.70
143.51
134.10
129.09
126.94
126.63
125.33
125.11
123.07
120.88
97.13
58.61
52.02
28.62
28.31
27.99
27.67
13.36
f1 (ppm)

2120

Compound 26c

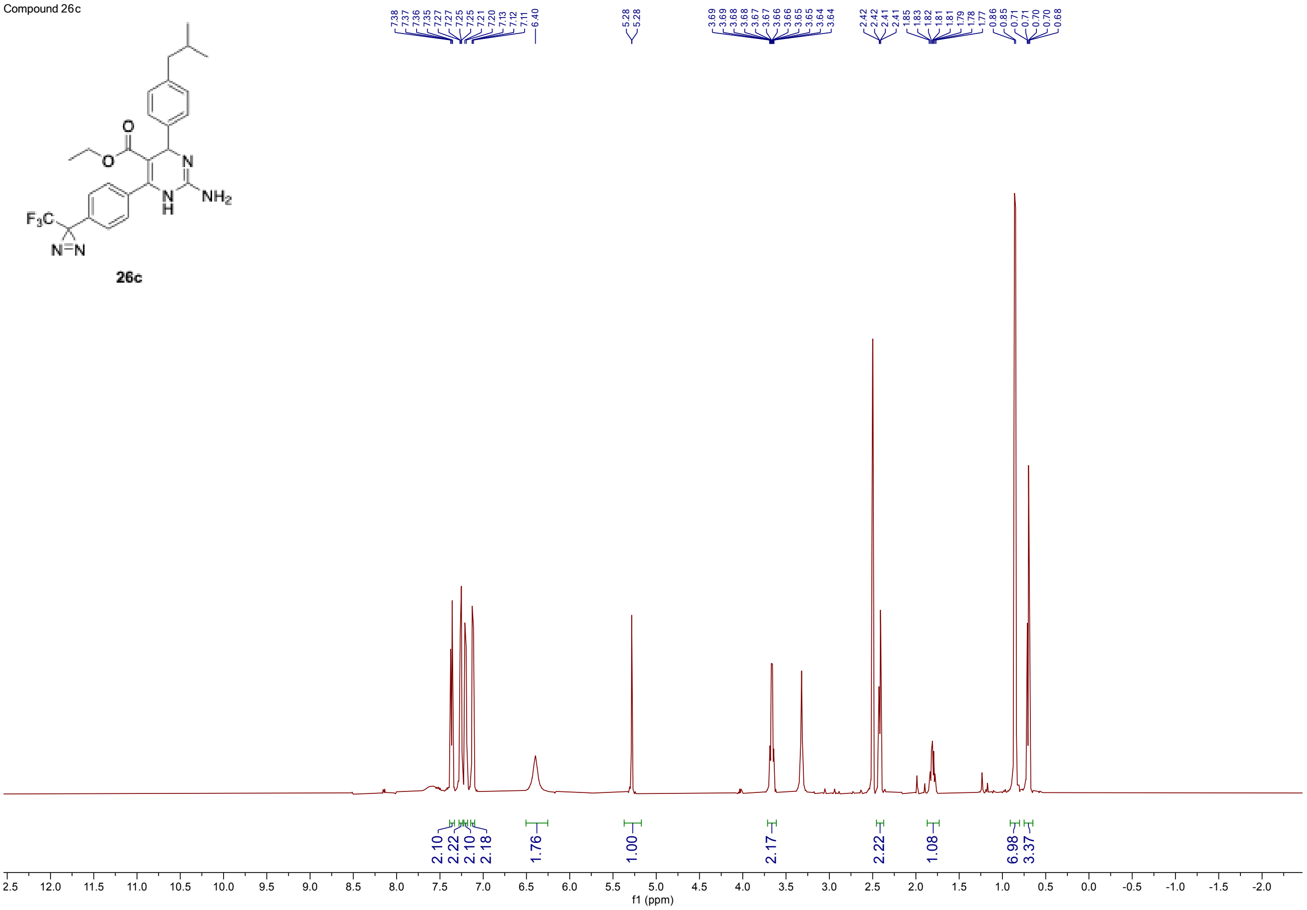

Compound 26c

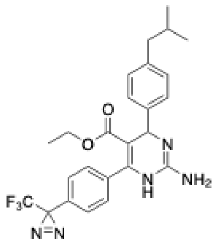

26c

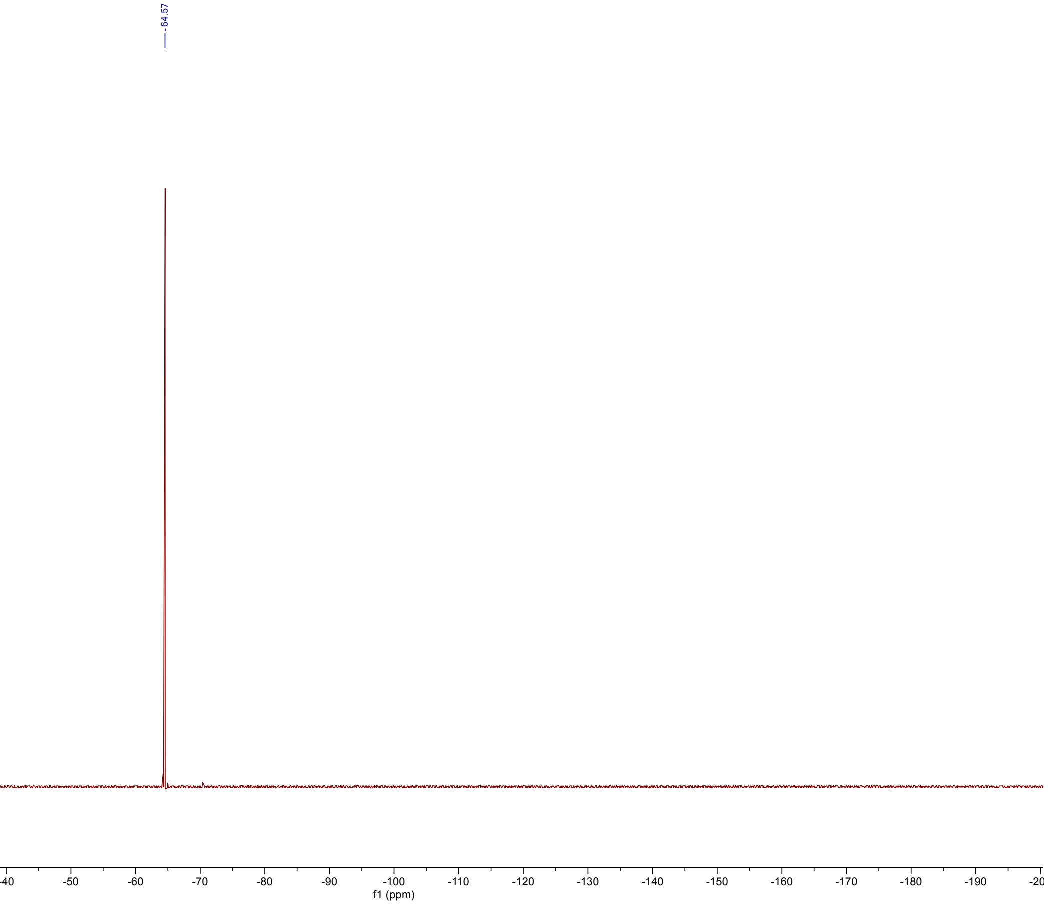

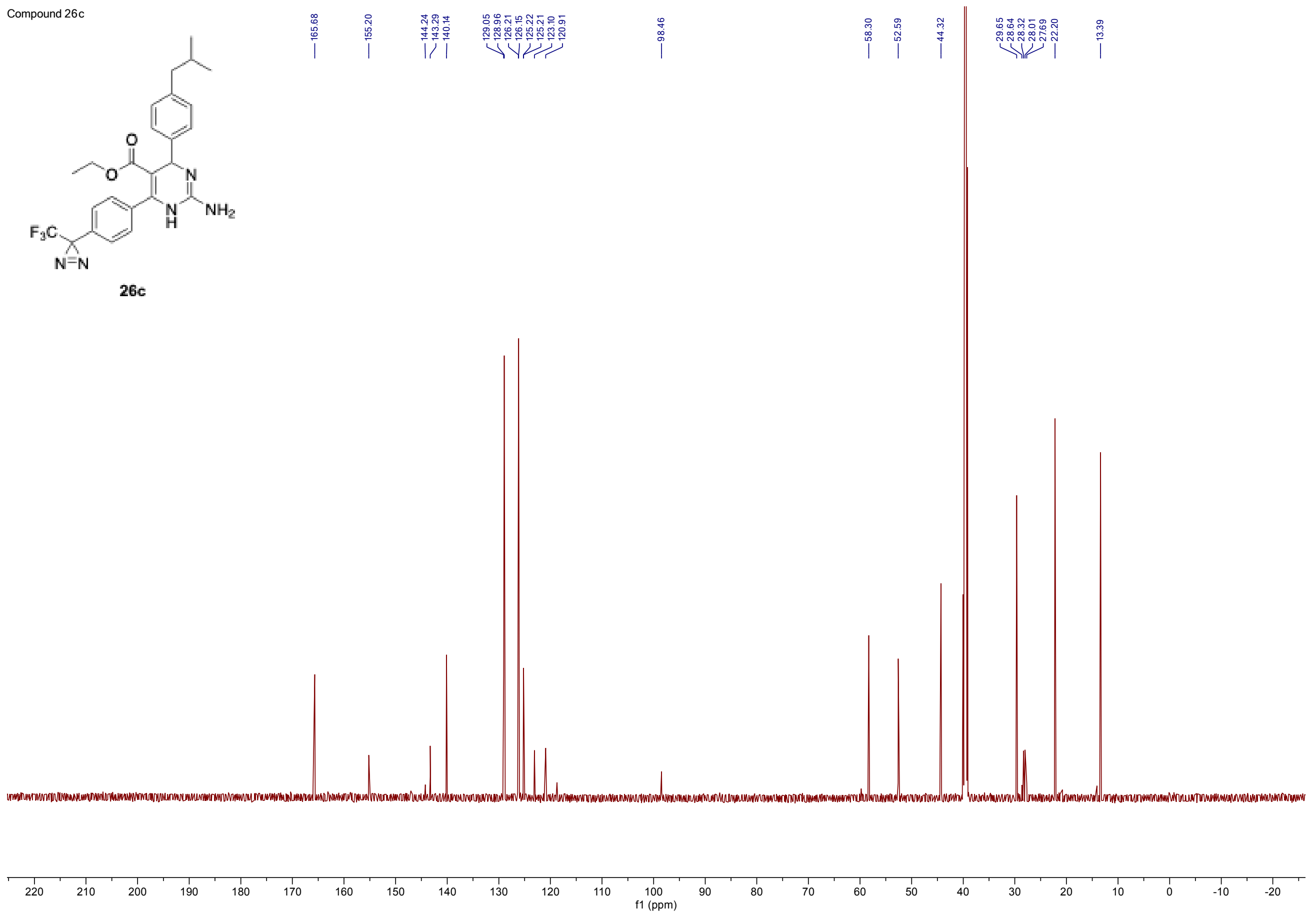
Compound 26c
26c
165.68
155.20
144.24
143.29
140.14
129.05
128.96
126.21
126.15
125.22
125.21
123.10
120.91
98.46
58.30
52.59
44.32
29.65
28.64
28.32
28.01
27.69
22.20
13.39
f1 (ppm)

Compound 26d

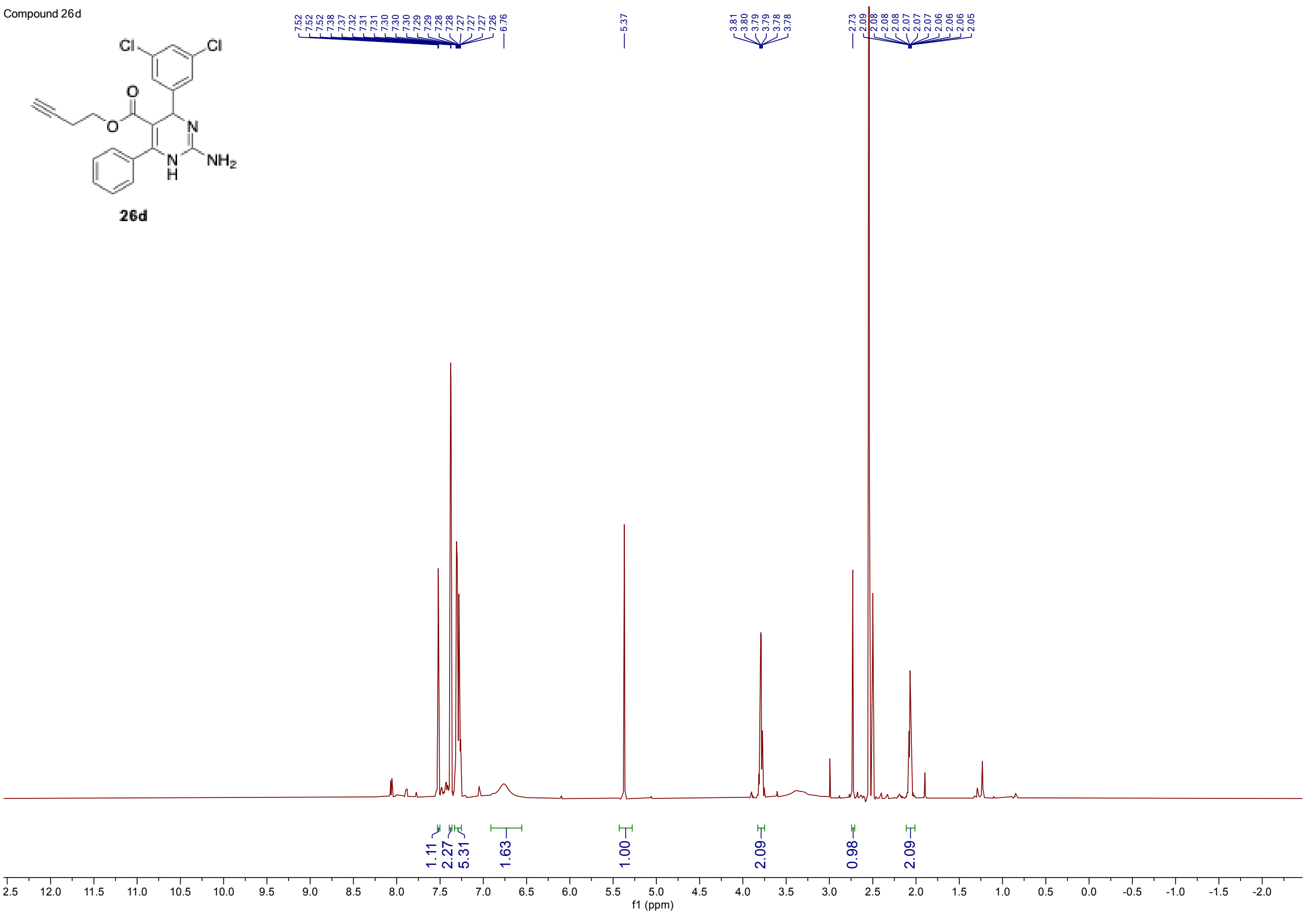

Compound 26d

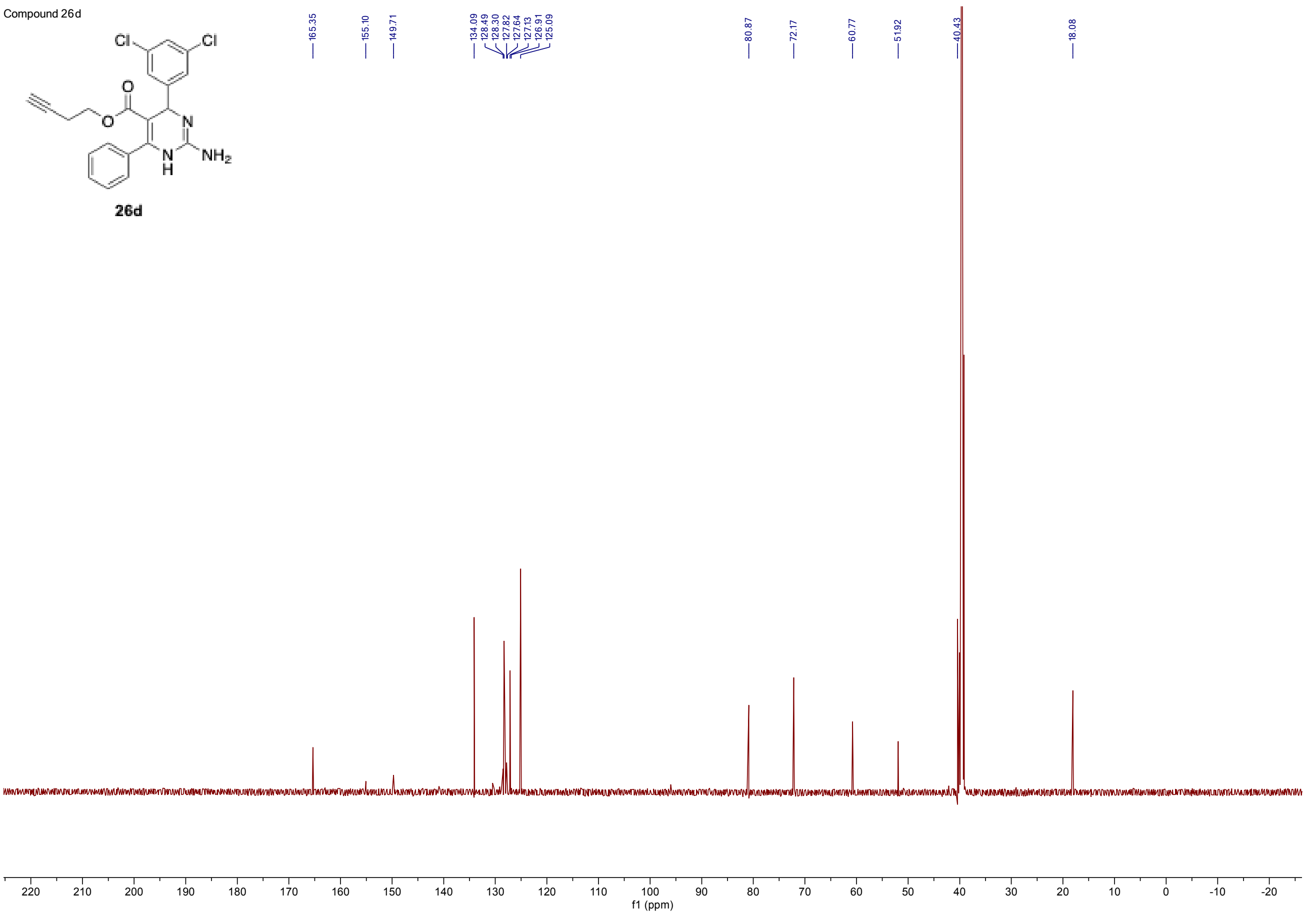

2130

Compound 26e

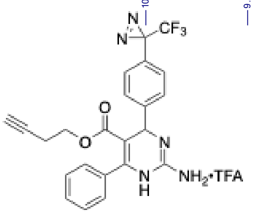

26e

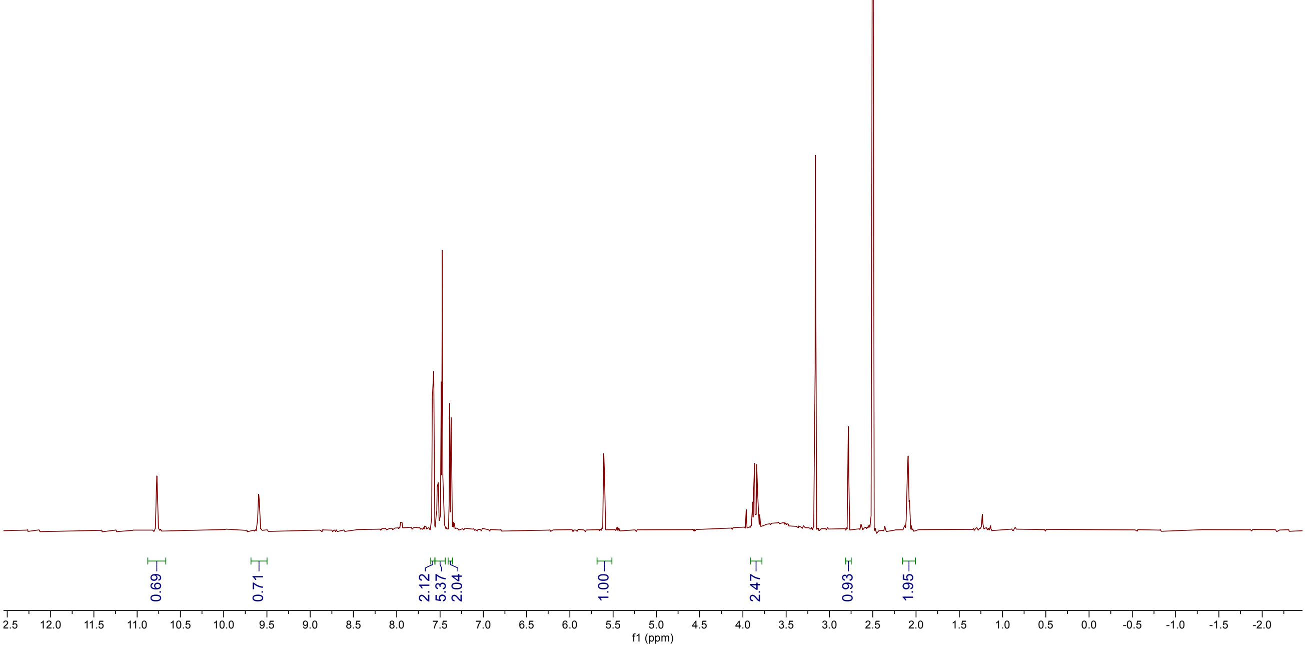

Compound 26e

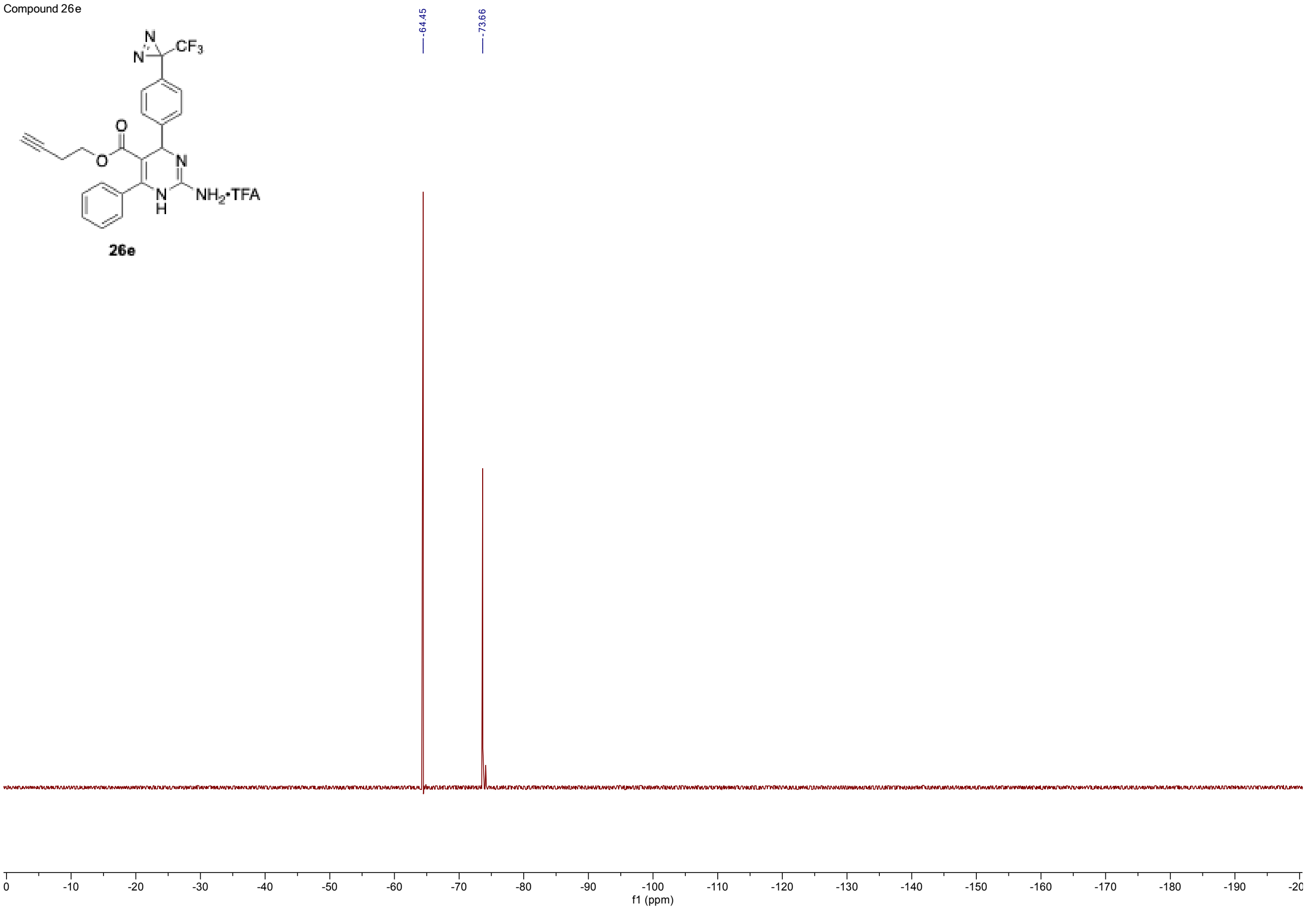

Compound 26e

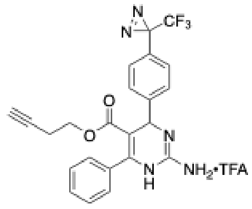

26e

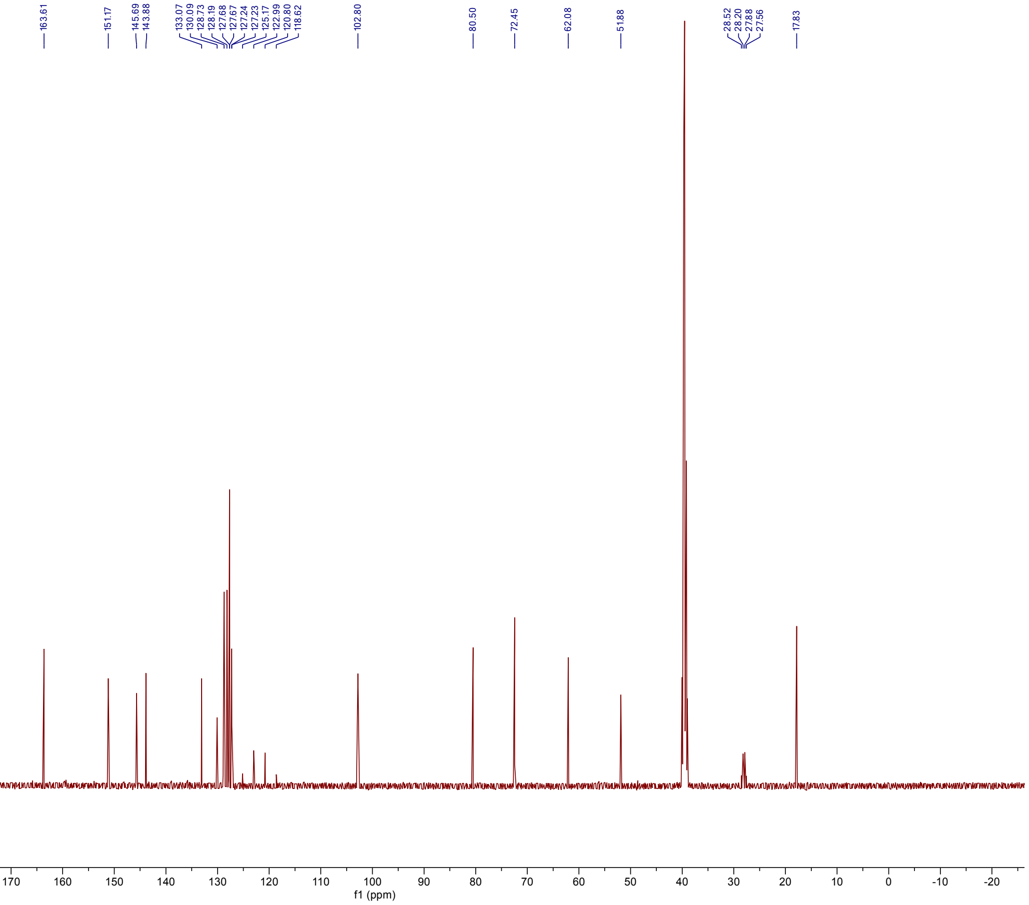

2135

Compound 26f

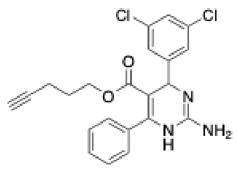

26f

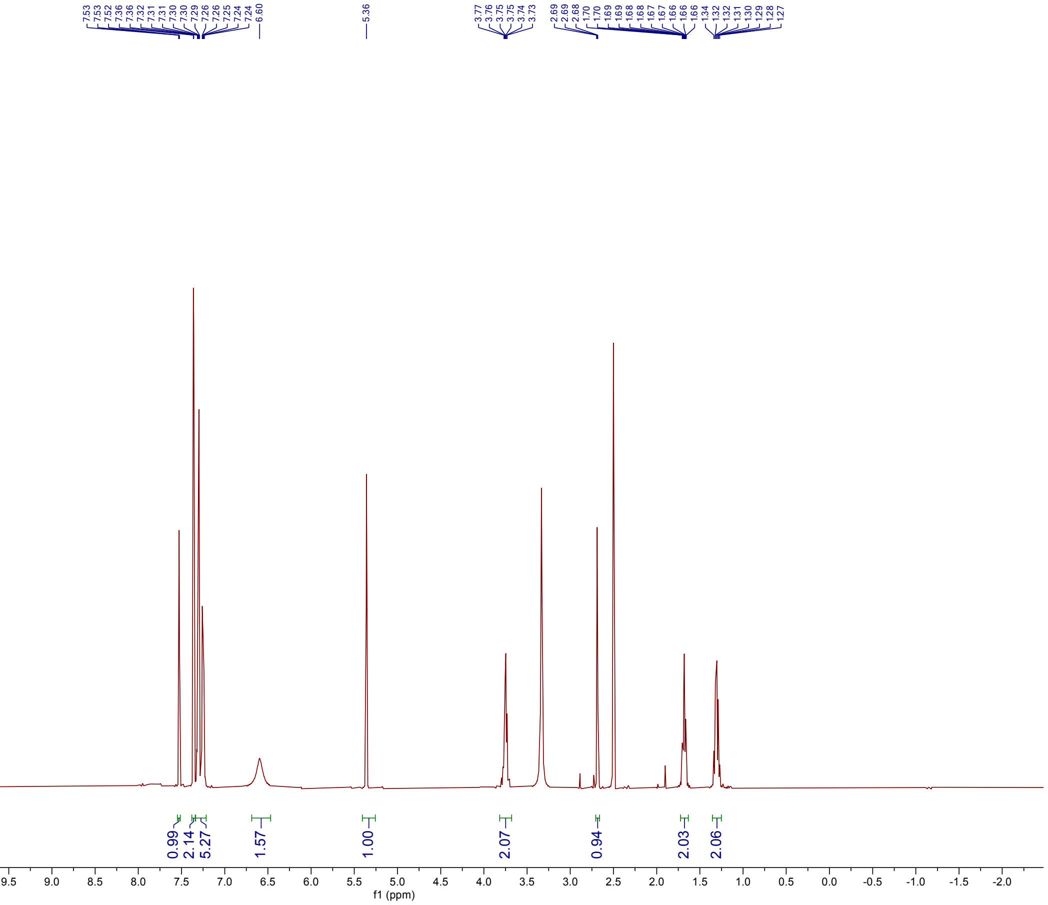

Compound 26f

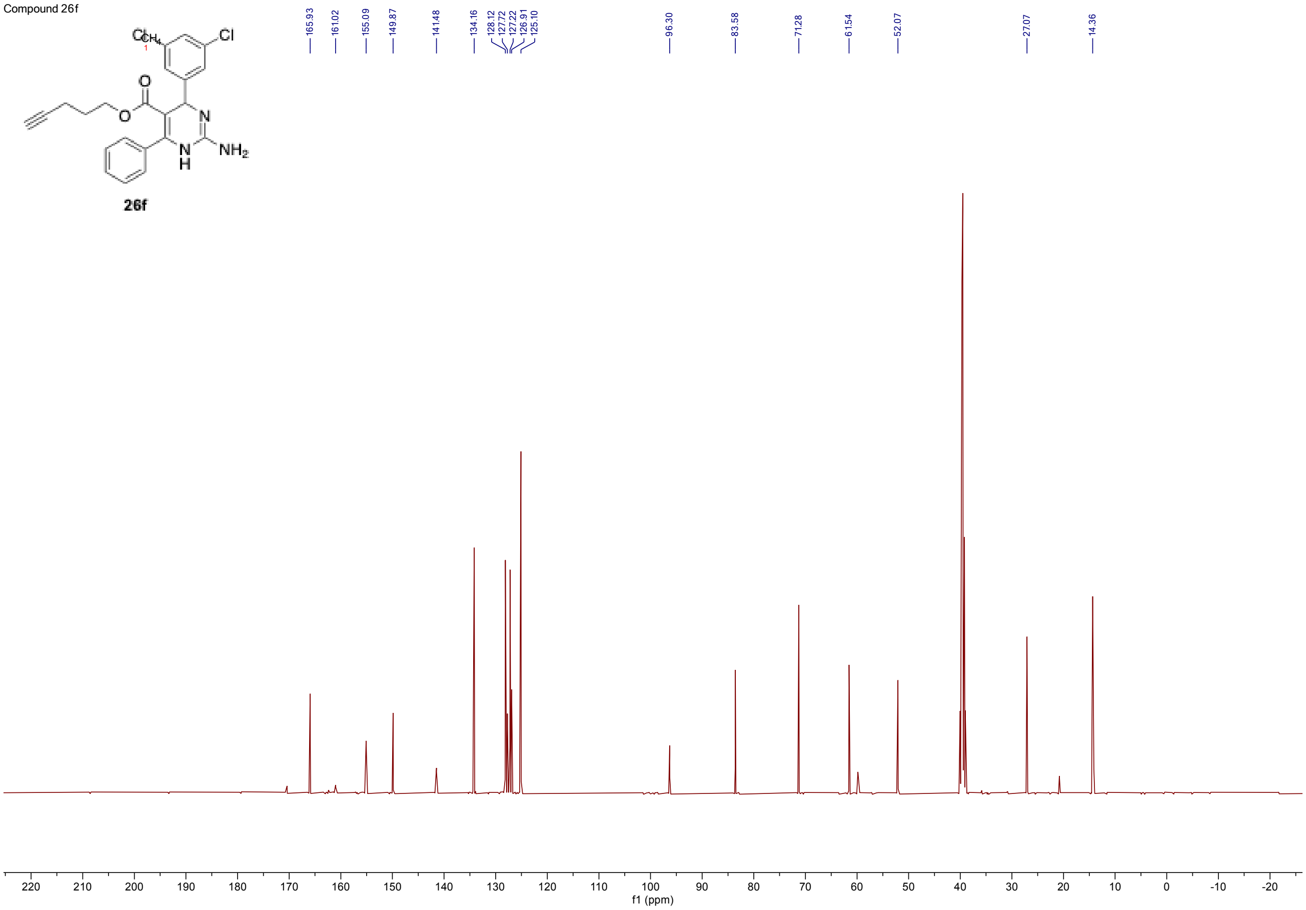

2140

Compound 26g

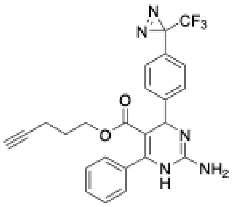

26g

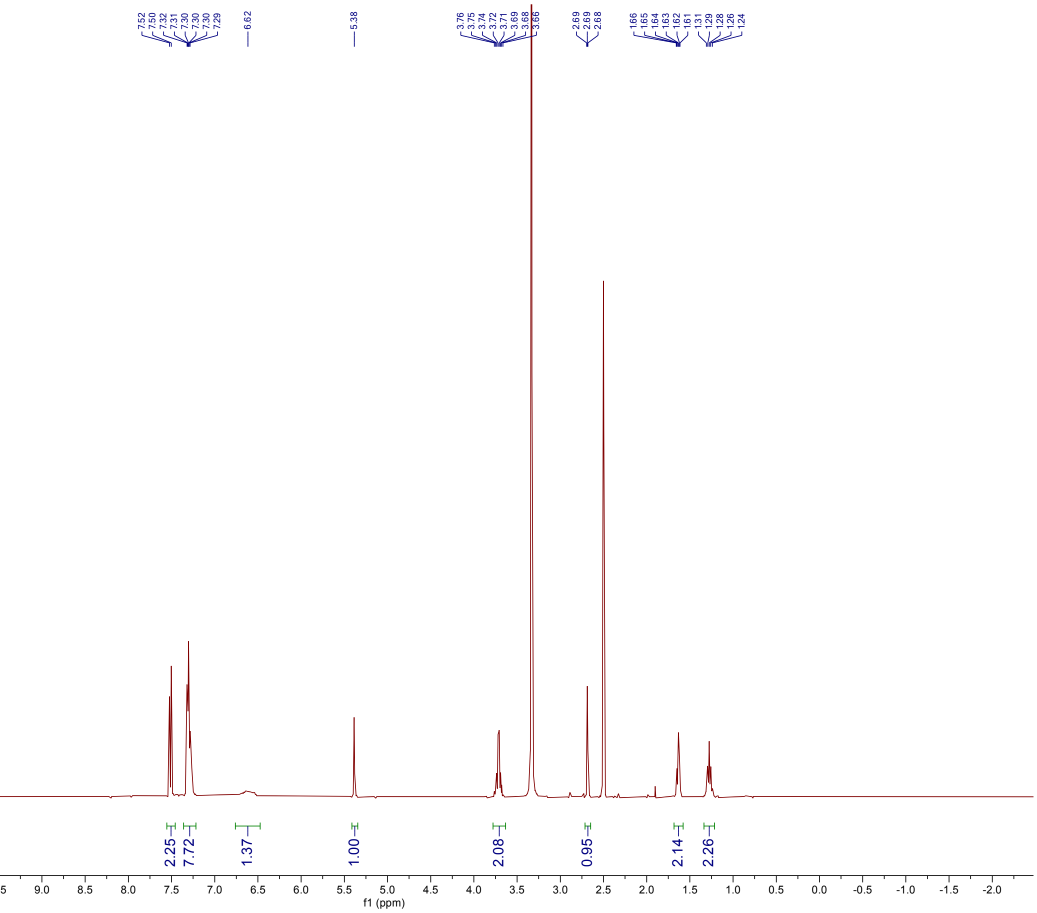

Compound 26g

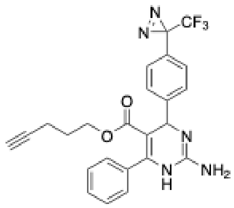

26g

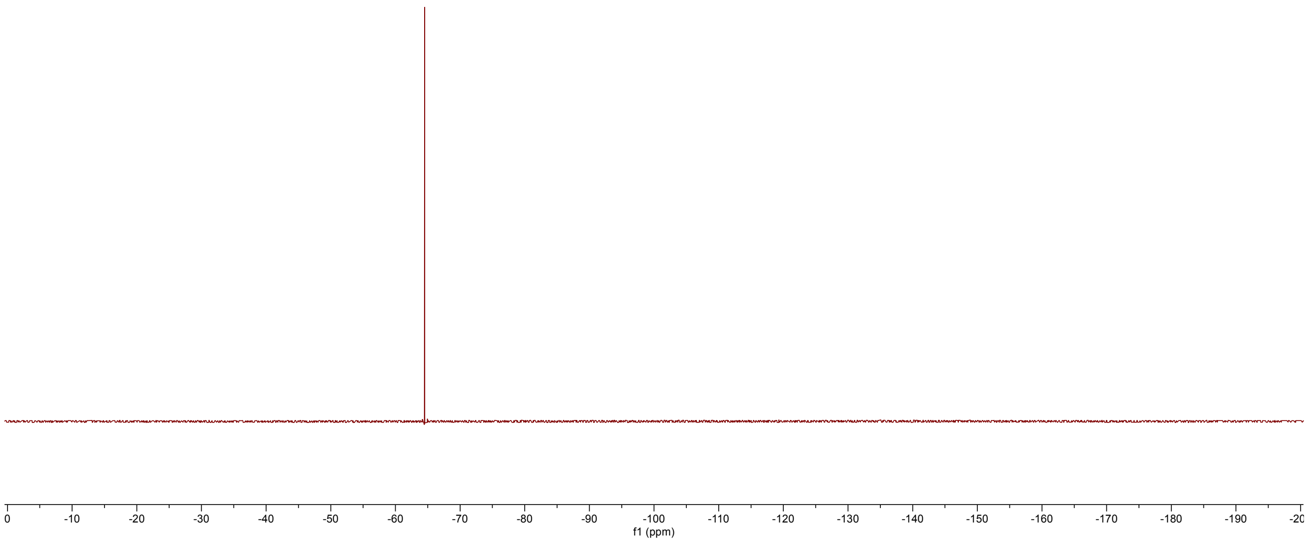

Compound 26g

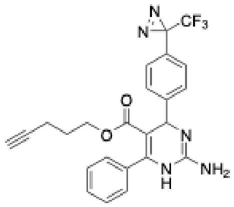

26g

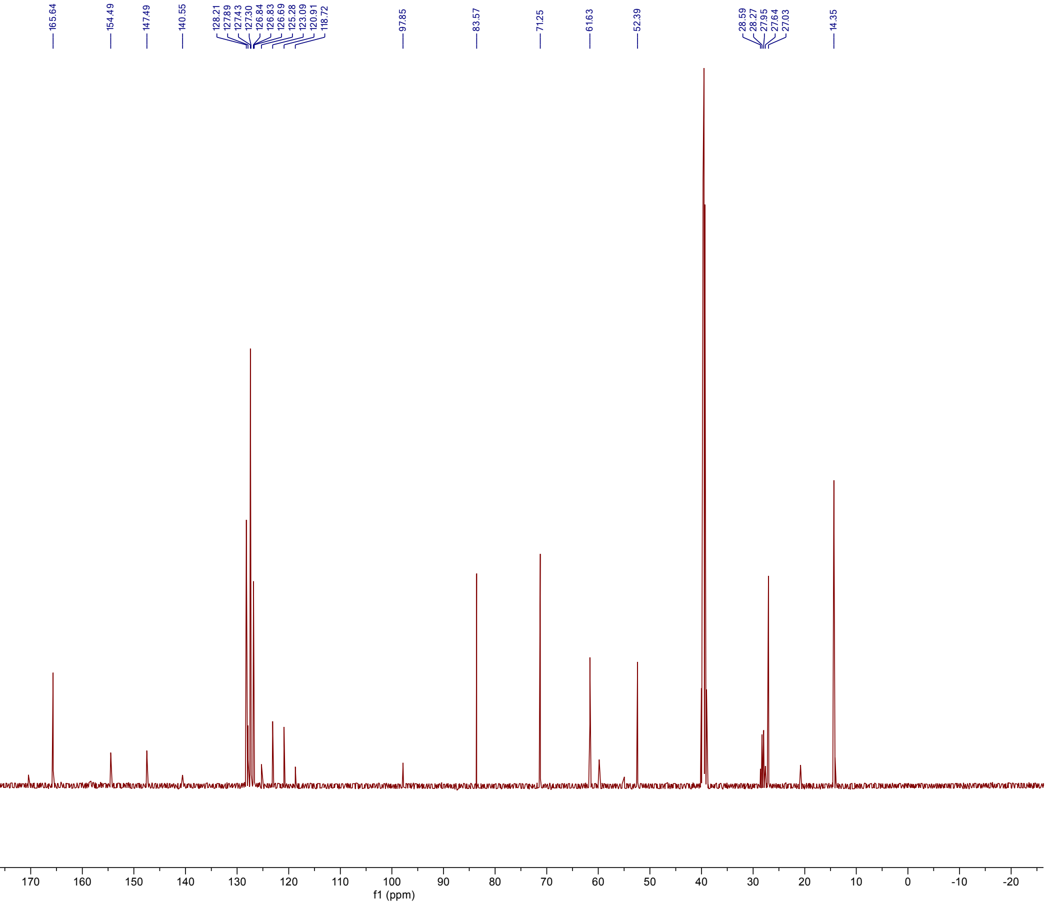

2145

Compound 26h

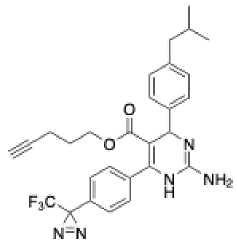

26h

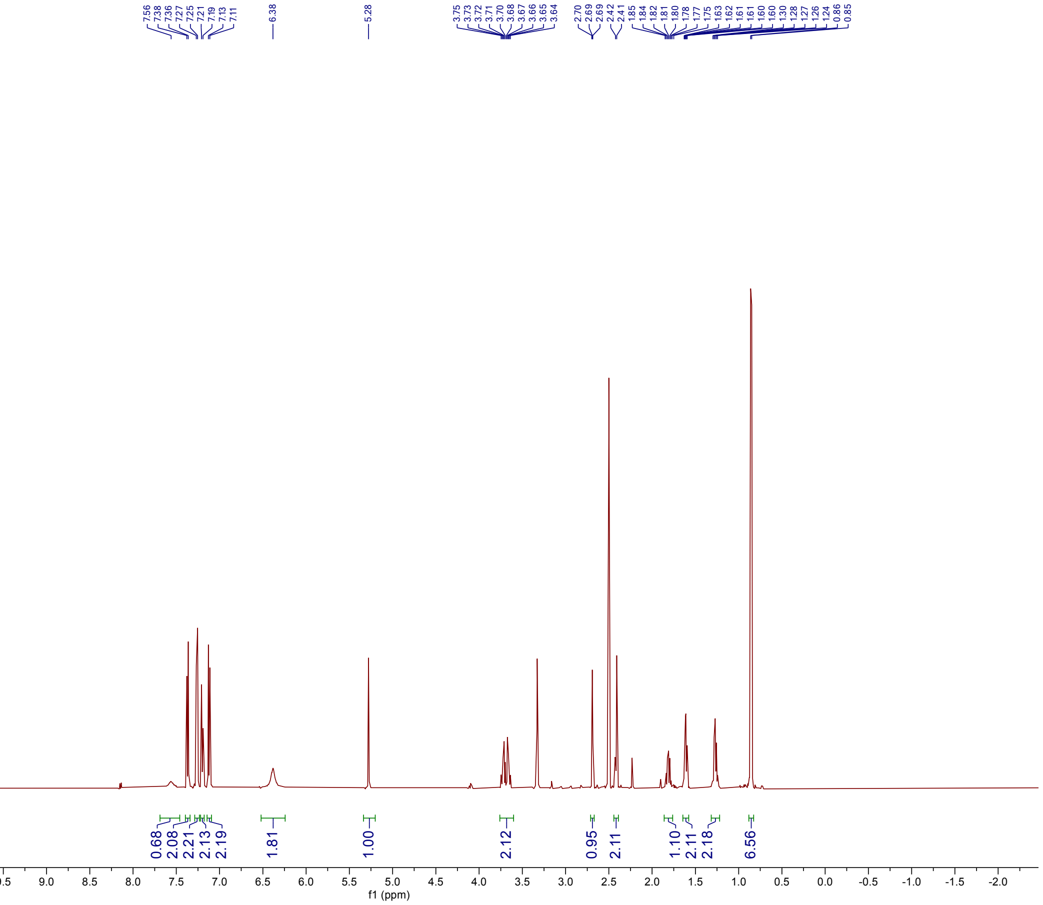

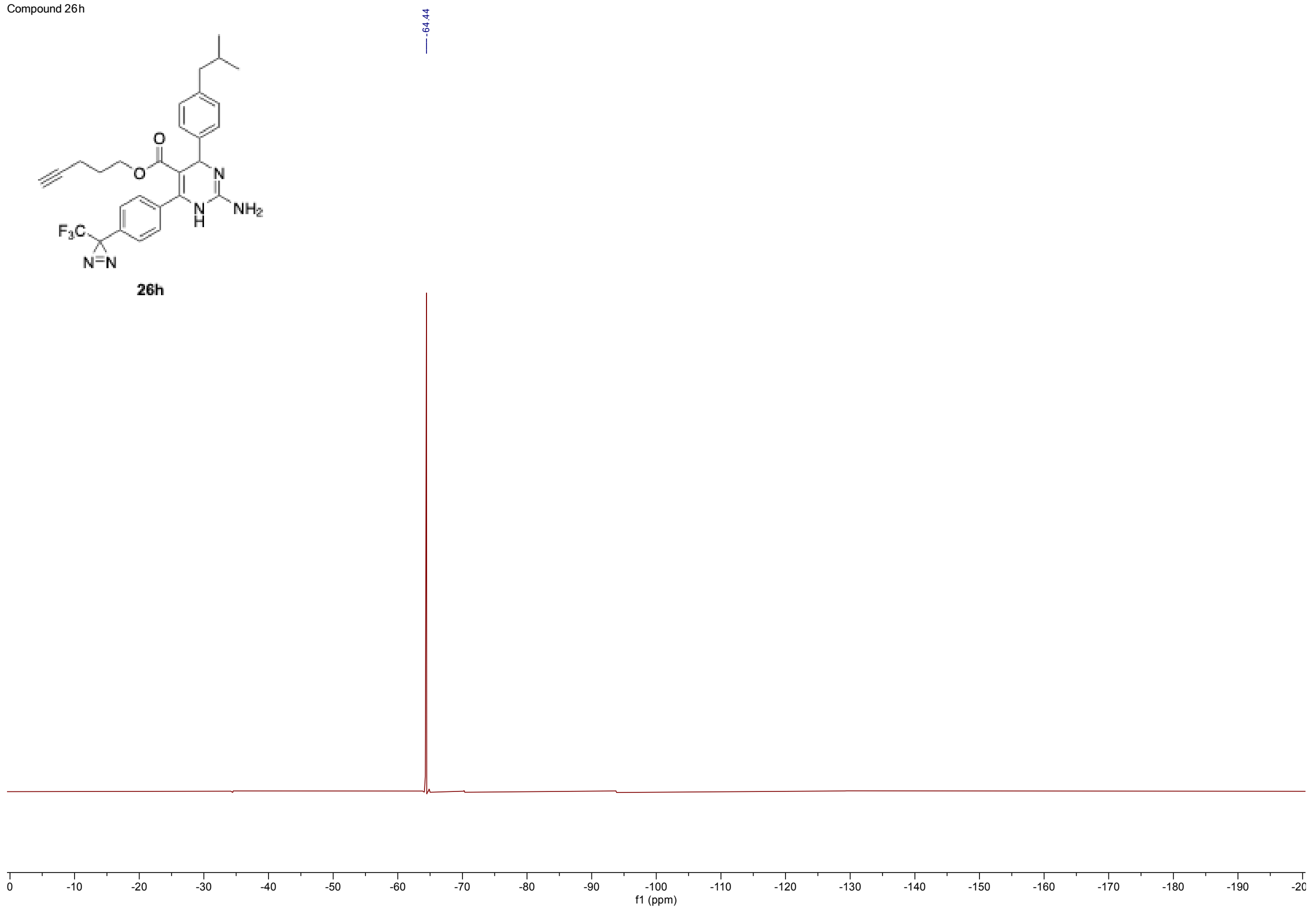
Compound 26h
-64.44
O
O
N
NH2
N
H
F3C
N=N
26h
0 -10 -20 -30 -40 -50 -60 -70 -80 -90 -100 -110 -120 -130 -140 -150 -160 -170 -180 -190 -20
f1 (ppm)

2150

Compound 26h

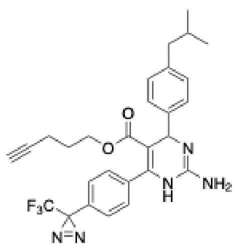

26h

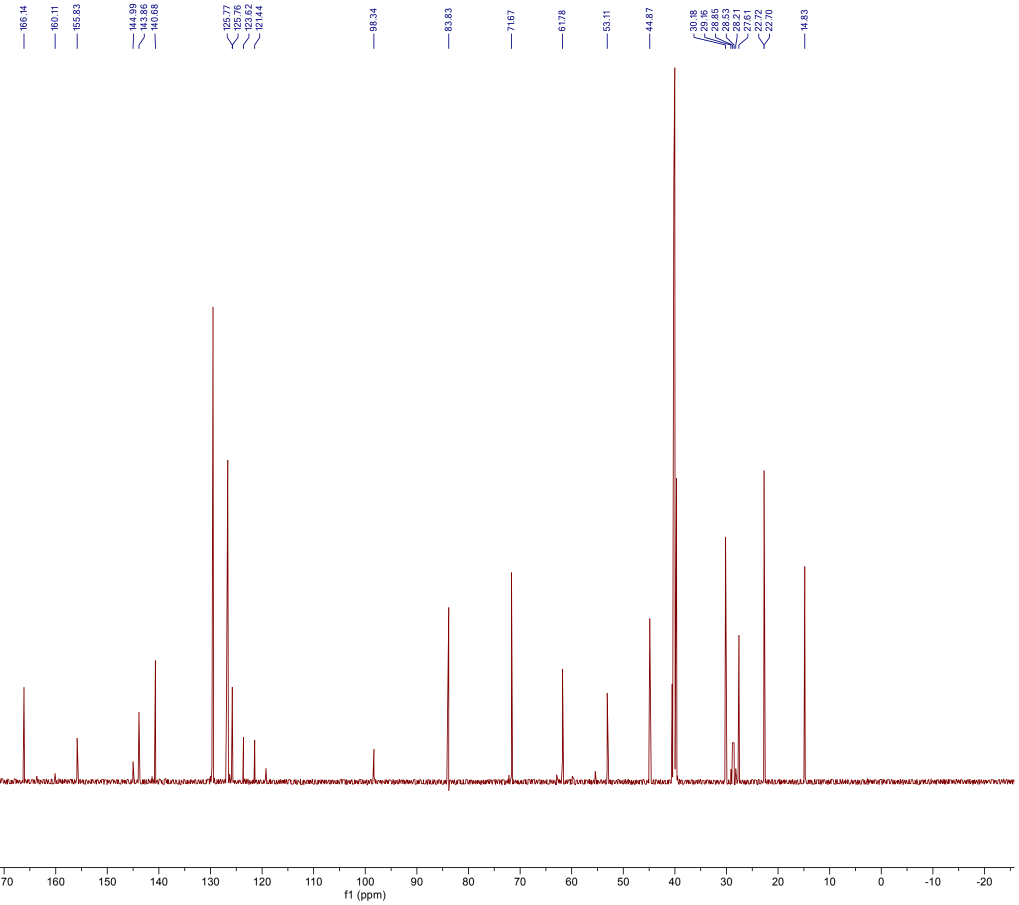

References:

(1) Boyer, Z. W.; Kessler, H.; Brosman, H.; Ruud, K. J.; Falkowski, A. F.; Viollet, C.; Bourne, C. R.; O'Reilly, M. C. Synthesis and Characterization of Functionalized Amino Dihydropyrimidines Toward the Analysis of Their Antibacterial Structure–Activity Relationships and Mechanism of Action. *ACS Omega* **2022**, *7* (42), 37907–37916. https://doi.org/10.1021/acsomega.2c05071.

(2) Smith, C. C.; Hollenstein, M.; Leumann, C. J. The Synthesis and Application of a Diazirine-Modified Uridine Analogue for Investigating RNA–Protein Interactions. *RSC Adv.* **2014**, *4* (89), 48228–48235. https://doi.org/10.1039/C4RA08682A.

(3) Stolze, S. C.; Liu, N.; Wijdeven, R. H.; Tuin, A. W.; Nieuwendijk, A. M. C. H. van den; Florea, B. I.; Stelt, M. van der; Marel, G. A. van der; Neefjes, J. J.; Overkleeft, H. S. Photo-Crosslinking of Clinically Relevant Kinases Using H89-Derived Photo-Affinity Probes. *Mol. Biosyst.* **2016**, *12* (6), 1809–1817. https://doi.org/10.1039/C6MB00257A.

(4) Casás-Selves, M.; Zhang, A. X.; Dowling, J. E.; Hallén, S.; Kawatkar, A.; Pace, N. J.; Denz, C. R.; Pontz, T.; Garahdaghi, F.; Cao, Q.; Sabirsh, A.; Thakur, K.; O'Connell, N.; Hu, J.; Cornella-Taracido, I.; Weerapana, E.; Zinda, M.; Goodnow Jr., R. A.; Castaldi, M. P. Target Deconvolution Efforts on Wnt Pathway Screen Reveal Dual Modulation of Oxidative Phosphorylation and SERCA2. *ChemMedChem* **2017**, *12* (12), 917–924. https://doi.org/10.1002/cmdc.201700028.
